# Supplementary material for: Selective androgen receptor degrader (SARD) to overcome antiandrogen resistance in castration-resistant prostate cancer
Source: eLife. 2023 Jan 19;12:e70700. doi: 10.7554/eLife.70700 (PMC9901937; doi:10.7554/eLife.70700)
Supplement: Supplementary file 1. — (a) The chemical structures of 80 candidate compounds. (b) Primer sequences for qRT-PCR. (c) The spectroscopic data of the Z15 derivatives. (d) Decreased protein profiles of LNCaP cells treated with ARV-110 plus DHT compared to DHT detected by 4D-lael free proteomics. (e) Decreased protein profiles of LNCaP cells treated with Z15 plus DHT compared to DHT detected by 4D-lael free proteomics [file elife-70700-supp1.doc]

**Supplementary File 1**

**Supplementary file 1a: The chemical structures of 80 candidate compounds**

| Z1 | Z2 | Z3 |
| --- | --- | --- |
| Z4 | Z5 | Z6 |
| Z7 | Z8 | Z9 |
| Z10 | Z11 | Z12 |
| Z13 | Z14 | Z15 |
| Z16 | Z17 | Z18 |
| Z19 | Z20 | Z21 |
| Z22 | Z23 | Z24 |
| Z25 | Z26 | Z27 |
| Z28 | Z29 | Z30 |
| Z31 | Z32 | Z33 |
| Z34 | Z35 | Z36 |
| Z37 | Z38 | Z39 |
| Z40 | Z41 | Z42 |
| Z43 | Z44 | Z45 |
| Z46 | Z47 | Z48 |
| Z49 | Z50 | Z51 |
| Z52 | Z53 | Z54 |
| Z55 | Z56 | Z57 |
| Z58 | Z59 | Z60 |
| Z61 | Z62 | Z63 |
| Z64 | Z65 | Z66 |
| Z67 | Z68 | Z69 |
| Z70 | Z71 | Z72 |
| Z73 | Z74 | Z75 |
| Z76 | Z77 | Z78 |
| Z79 | Z80 |  |

**Supplementary file 1b: Primer sequences for qRT-PCR**

| Segments | Primer sequences |
| --- | --- |
| GAPDH | up: 5’-GGT ATC GTG GAA GGA CTC ATG AC-3’ |
|  | down: 5’-ATG CCA GTG AGC TTC CCG TTC AG-3’ |
| PSA | up: 5’-GGT GAC CAA GTT CAT GCT GTG-3’ |
|  | down: 5’-GTG TCC TTG ATC CAC TTC CG-3’ |
| AR  TMPRSS2  PMEPA1 | up: 5’- ACATCAAGGAACTCGATCGTATCATTGC-3’ |
|  | down: 5’- TTGGGCACTTGCACAGAGAT-3’  up: 5’-CAGGAGTGTACGGGAATGTGATGGT-3’  down: 5’- GATTAGCCGTCTGCCCTCATTTGT-3’  up: 5’- GTGCAACTGCAAACGCTCTT-3’  down: 5’- AGCTTGTAGTGGCTCAGCAG-3’ |

**Supplementary file 1c: The spectroscopic data of the Z15 derivatives**

| **Compound ID** | **Vendors (ID)** | **Test parameter** |
| --- | --- | --- |
| ZL-1 | Life chemicals lnc. (F0473-0018) | TOF MS(ESI), m/z [M + H]+: Calc’d, 344.46, found, 345.2, HPLC(Retention Time:1.226 min; Purity:100.0%) |
| ZL-2 | Enamine  (Z28306927) | TOF MS(ESI), m/z [M + H]+: Calc’d, 425.53, found, 426.0, HPLC(Retention Time:0.690 min; Purity:98.4%) |
| ZL-3 | Enamine  (Z30198298) | TOF MS(ESI), m/z [M + H]+: Calc’d, 385.49, found, 386.0, HPLC(Retention Time:0.710 min; Purity:97.6%) |
| ZL-4 | Enamine  (Z30198298) | TOF MS(ESI), m/z [M + H]+: Calc’d, 379.43, found, 380.2, HPLC(Retention Time:1.388 min; Purity:100.0%) |
| ZL-5 | Enamine  (Z26660731) | TOF MS(ESI), m/z [M + H]+: Calc’d, 436.54, found, 437.3, HPLC(Retention Time:0.751 min; Purity:91.8%) |
| ZL-6 | Enamine  (PB228330606) | TOF MS(ESI), m/z [M + H]+: Calc’d, 454.59, found, 455.2, HPLC(Retention Time:1.070 min; Purity:100.0%) |
| ZL-7 | Enamine  (PB228374000) | TOF MS(ESI), m/z [M + H]+: Calc’d, 397.49, found, 398.2, HPLC(Retention Time:1.534 min; Purity:95.2%) |

**Supplementary file 1d: Decreased protein profiles of LNCaP cells treated with ARV-110 plus DHT compared to DHT detected by 4D-lael free proteomics**

| **Type** | **Protein accession** | **Gene name** | **Fold change** | **Fold P value** | **MW [kDa]** | **Coverage [%]** | **Unique peptides** |
| --- | --- | --- | --- | --- | --- | --- | --- |
| 1 | O00488 | ZNF593 | 0.592 | 0.024403 | 15.199 | 32.1 | 3 |
|  | O14965 | AURKA | 0.551 | 0.008807 | 45.809 | 14.6 | 4 |
|  | O15270 | SPTLC2 | 0.65 | 0.034038 | 62.924 | 14.4 | 5 |
|  | O15393 | TMPRSS2 | 0.661 | 0.000387 | 53.859 | 19.7 | 7 |
|  | O95235 | KIF20A | 0.568 | 0.000187 | 100.28 | 26.6 | 18 |
|  | P07288 | KLK3 | 0.573 | 2.95E-05 | 28.741 | 58.2 | 8 |
|  | P10275 | AR | 0.205 | 1.39E-07 | 99.187 | 20.7 | 13 |
|  | P18440 | NAT1 | 0.584 | 0.001592 | 33.898 | 23.4 | 6 |
|  | P31350 | RRM2 | 0.646 | 8.28E-06 | 44.877 | 52.4 | 14 |
|  | P36956 | SREBF1 | 0.64 | 0.033547 | 121.67 | 6.5 | 5 |
|  | P41567 | EIF1 | 0.611 | 0.009745 | 12.732 | 77.9 | 3 |
|  | Q12834 | CDC20 | 0.474 | 0.0003 | 54.722 | 11.4 | 4 |
|  | Q12899 | TRIM26 | 0.562 | 0.003625 | 62.165 | 17.1 | 7 |
|  | Q15014 | MORF4L2 | 0.607 | 2.59E-05 | 32.307 | 37.5 | 7 |
|  | Q8TF72 | SHROOM3 | 0.344 | 0.017201 | 216.85 | 12.2 | 15 |
|  | Q9BW19 | KIFC1 | 0.616 | 0.000833 | 73.747 | 23 | 11 |
|  | Q9BZQ6 | EDEM3 | 0.507 | 3.04E-06 | 104.66 | 11.8 | 8 |
|  | Q9NVP2 | ASF1B | 0.635 | 0.016002 | 22.433 | 45 | 5 |
|  | Q9Y4U1 | MMACHC | 0.556 | 0.026613 | 31.728 | 24.8 | 5 |
| 2 | P30519 | HMOX2 | 0.594 | 0.000398 | 36.032 | 56.6 | 12 |
|  | P48200 | IREB2 | 0.27 | 0.001134 | 105.06 | 10.8 | 6 |
|  | P48729 | CSNK1A1 | 0.612 | 2.51E-05 | 38.914 | 47.5 | 13 |
|  | Q0VGL1 | LAMTOR4 | 0.537 | 3.07E-05 | 10.741 | 28.3 | 2 |
|  | Q13433 | SLC39A6 | 0.446 | 0.033499 | 85.046 | 2.9 | 2 |
|  | Q6PIJ6 | FBXO38 | 0.583 | 0.01519 | 133.94 | 6.7 | 6 |
|  | Q8IWF2 | FOXRED2 | 0.664 | 0.012947 | 77.79 | 7.7 | 4 |
|  | Q92597 | NDRG1 | 0.559 | 0.000245 | 42.835 | 47.2 | 11 |
|  | Q96QD8 | SLC38A2 | 0.504 | 0.000862 | 56.025 | 7.3 | 2 |
|  | Q99618 | CDCA3 | 0.558 | 0.006232 | 28.998 | 15.3 | 2 |
|  | Q99801 | NKX3-1 | 0.534 | 0.001737 | 26.35 | 22.2 | 3 |
|  | Q9H0U9 | TSPYL1 | 0.638 | 0.001687 | 49.192 | 20.8 | 7 |
|  | Q9H967 | WDR76 | 0.648 | 0.003089 | 69.768 | 6.5 | 3 |
|  | Q9NV92 | NDFIP2 | 0.572 | 0.044804 | 36.389 | 7.4 | 2 |
|  | Q9NYZ3 | GTSE1 | 0.656 | 0.016275 | 76.644 | 14.7 | 6 |

Note: Type 1 means proteins down-regulated by both ARV-110 and Z15, type 2 means proteins down-regulated only by ARV-110.

**Supplementary file 1e: Decreased protein profiles of LNCaP cells treated with Z15 plus DHT compared to DHT detected by 4D-lael free proteomics**

| **Type** | **Protein accession** | **Gene name** | **Fold change** | **Fold P value** | **MW [kDa]** | **Coverage [%]** | **Unique peptides** |
| --- | --- | --- | --- | --- | --- | --- | --- |
| 1 | O00488 | ZNF593 | 0.494 | 0.010869 | 15.199 | 32.1 | 3 |
|  | O14965 | AURKA | 0.583 | 0.04144 | 45.809 | 14.6 | 4 |
|  | O15270 | SPTLC2 | 0.557 | 0.001984 | 62.924 | 14.4 | 5 |
|  | O15393 | TMPRSS2 | 0.571 | 5.28E-06 | 53.859 | 19.7 | 7 |
|  | O95235 | KIF20A | 0.463 | 4.53E-05 | 100.28 | 26.6 | 18 |
|  | P07288 | KLK3 | 0.435 | 9.16E-06 | 28.741 | 58.2 | 8 |
|  | P10275 | AR | 0.368 | 2.07E-06 | 99.187 | 20.7 | 13 |
|  | P18440 | NAT1 | 0.548 | 0.008395 | 33.898 | 23.4 | 6 |
|  | P31350 | RRM2 | 0.528 | 1.33E-06 | 44.877 | 52.4 | 14 |
|  | P36956 | SREBF1 | 0.649 | 0.030734 | 121.67 | 6.5 | 5 |
|  | P41567 | EIF1 | 0.639 | 0.004154 | 12.732 | 77.9 | 3 |
|  | Q12834 | CDC20 | 0.428 | 0.000863 | 54.722 | 11.4 | 4 |
|  | Q12899 | TRIM26 | 0.65 | 0.001774 | 62.165 | 17.1 | 7 |
|  | Q15014 | MORF4L2 | 0.546 | 9.42E-06 | 32.307 | 37.5 | 7 |
|  | Q8TF72 | SHROOM3 | 0.342 | 0.027503 | 216.85 | 12.2 | 15 |
|  | Q9BW19 | KIFC1 | 0.498 | 0.007692 | 73.747 | 23 | 11 |
|  | Q9BZQ6 | EDEM3 | 0.574 | 0.002238 | 104.66 | 11.8 | 8 |
|  | Q9NVP2 | ASF1B | 0.481 | 0.00011 | 22.433 | 45 | 5 |
|  | Q9Y4U1 | MMACHC | 0.335 | 0.003536 | 31.728 | 24.8 | 5 |
| 2 | A0A096LP01 | SMIM26 | 0.417 | 0.002326 | 10.908 | 25.3 | 2 |
|  | O00762 | UBE2C | 0.619 | 0.030896 | 19.652 | 41.3 | 6 |
|  | O14757 | CHEK1 | 0.567 | 0.000388 | 54.433 | 11.3 | 4 |
|  | O15379 | HDAC3 | 0.623 | 0.00414 | 48.847 | 16.4 | 4 |
|  | O43663 | PRC1 | 0.666 | 0.006486 | 71.606 | 10 | 5 |
|  | O60427 | FADS1 | 0.635 | 0.029512 | 51.964 | 23.6 | 7 |
|  | O95070 | YIF1A | 0.551 | 0.032217 | 32.011 | 19.8 | 4 |
|  | O95297 | MPZL1 | 0.661 | 0.001021 | 29.082 | 27.1 | 5 |
|  | O95864 | FADS2 | 0.479 | 5.18E-06 | 52.259 | 27.3 | 12 |
|  | P07948 | LYN | 0.615 | 0.013084 | 58.573 | 14.5 | 5 |
|  | P15104 | GLUL | 0.629 | 3.01E-05 | 42.064 | 22 | 7 |
|  | P36873 | PPP1CC | 0.48 | 0.009247 | 36.983 | 47.7 | 2 |
|  | P43357 | MAGEA3 | 0.555 | 0.00011 | 34.747 | 18.2 | 3 |
|  | P50579 | METAP2 | 0.535 | 1.52E-05 | 52.891 | 34.1 | 12 |
|  | P53350 | PLK1 | 0.636 | 0.009834 | 68.254 | 18.2 | 8 |
|  | P62487 | POLR2G | 0.636 | 0.014137 | 19.294 | 52.9 | 6 |
|  | P62841 | RPS15 | 0.654 | 0.000221 | 17.04 | 41.4 | 7 |
|  | P98179 | RBM3 | 0.623 | 0.000656 | 17.17 | 24.2 | 3 |
|  | Q01581 | HMGCS1 | 0.575 | 0.000427 | 57.293 | 50.6 | 18 |
|  | Q03111 | MLLT1 | 0.619 | 0.044851 | 62.055 | 12.7 | 6 |
|  | Q12983 | BNIP3 | 0.631 | 0.001668 | 27.832 | 13.5 | 3 |
|  | Q13426 | XRCC4 | 0.64 | 0.014215 | 38.286 | 29.2 | 7 |
|  | Q14493 | SLBP | 0.602 | 0.009447 | 31.285 | 15.2 | 4 |
|  | Q14807 | KIF22 | 0.59 | 0.000252 | 73.261 | 24.8 | 14 |
|  | Q15036 | SNX17 | 0.641 | 0.000578 | 52.901 | 20 | 6 |
|  | Q16763 | UBE2S | 0.648 | 6.68E-06 | 23.845 | 36.9 | 5 |
|  | Q3ZCM7 | TUBB8 | 0.626 | 0.003213 | 49.775 | 24.5 | 2 |
|  | Q53GT1 | KLHL22 | 0.585 | 0.038277 | 71.666 | 11 | 6 |
|  | Q58WW2 | DCAF6 | 0.464 | 0.04465 | 96.291 | 8.1 | 5 |
|  | Q6NUK4 | REEP3 | 0.659 | 0.002021 | 29.264 | 18.8 | 4 |
|  | Q6PL18 | ATAD2 | 0.599 | 8.61E-06 | 158.55 | 28.9 | 28 |
|  | Q71UM5 | RPS27L | 0.527 | 0.01344 | 9.4771 | 39.3 | 2 |
|  | Q86TB9 | PATL1 | 0.539 | 0.004696 | 86.849 | 10.5 | 5 |
|  | Q86Y07 | VRK2 | 0.663 | 0.032701 | 58.14 | 15 | 5 |
|  | Q8WXA9 | SREK1 | 0.441 | 0.000522 | 59.38 | 9.4 | 3 |
|  | Q92995 | USP13 | 0.616 | 0.001548 | 97.326 | 15.3 | 8 |
|  | Q96F63 | CCDC97 | 0.491 | 0.010815 | 38.946 | 22.7 | 4 |
|  | Q96T88 | UHRF1 | 0.655 | 0.000815 | 89.813 | 30 | 16 |
|  | Q9BPZ3 | PAIP2 | 0.482 | 0.004626 | 14.984 | 26 | 2 |
|  | Q9BV36 | MLPH | 0.636 | 0.003083 | 65.948 | 13.2 | 5 |
|  | Q9BX46 | RBM24 | 0.642 | 0.000484 | 24.776 | 24.6 | 4 |
|  | Q9GZY8 | MFF | 0.657 | 0.002856 | 38.464 | 28.4 | 7 |
|  | Q9HA47 | UCK1 | 0.599 | 0.024105 | 31.434 | 19.1 | 3 |
|  | Q9NPD8 | UBE2T | 0.594 | 4.02E-06 | 22.521 | 48.2 | 7 |
|  | Q9NRZ9 | HELLS | 0.505 | 0.004828 | 97.073 | 6.8 | 5 |
|  | Q9NUQ2 | AGPAT5 | 0.547 | 0.00176 | 42.072 | 19.8 | 5 |
|  | Q9NXV2 | KCTD5 | 0.595 | 0.002275 | 26.092 | 20.9 | 3 |
|  | Q9ULR0 | ISY1 | 0.581 | 0.016586 | 32.992 | 16.1 | 3 |
|  | Q9Y2D8 | SSX2IP | 0.631 | 0.025709 | 71.235 | 9.3 | 5 |
|  | Q9Y4C2 | TCAF1 | 0.367 | 0.000328 | 102.12 | 19.5 | 15 |

Note: Type 1 means proteins down-regulated by both ARV-110 and Z15, type 2 means proteins down-regulated only by Z15.
