# Supplementary material for: Selective androgen receptor degrader (SARD) to overcome antiandrogen resistance in castration-resistant prostate cancer
Source: eLife. 2023 Jan 19;12:e70700. doi: 10.7554/eLife.70700 (PMC9901937; doi:10.7554/eLife.70700)

Figure 2

Figure 2D

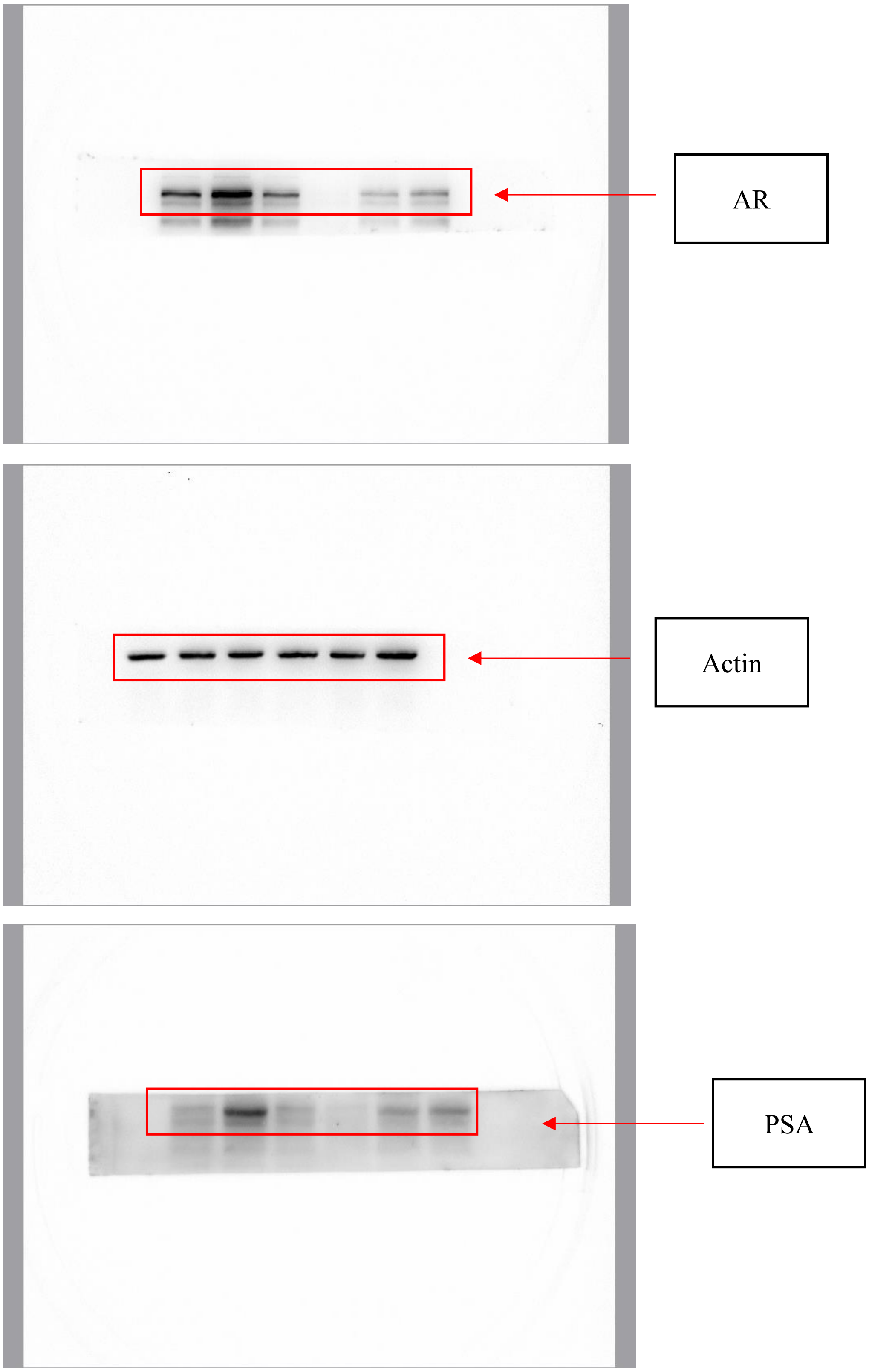

Figure 2E

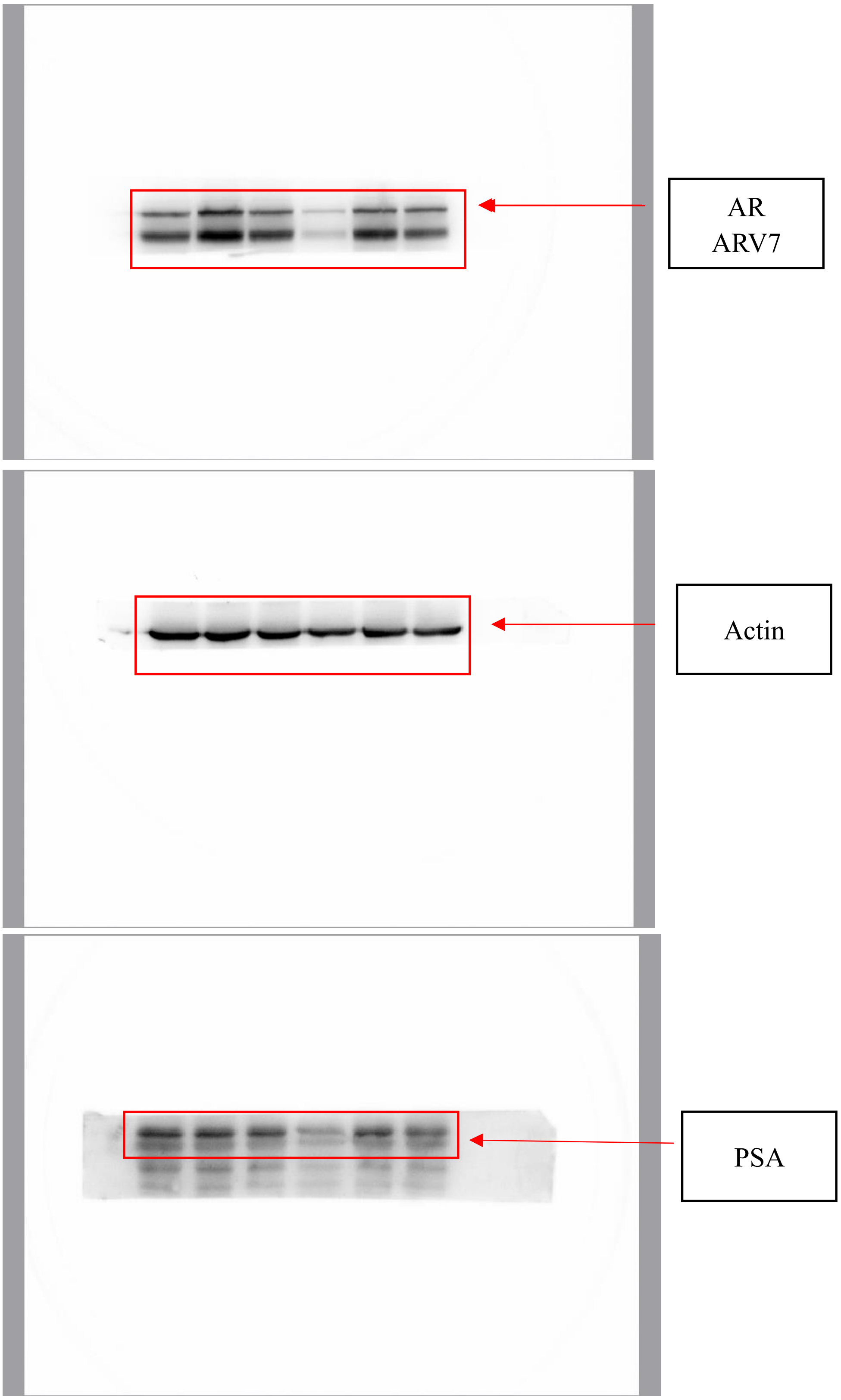

Figure 2F

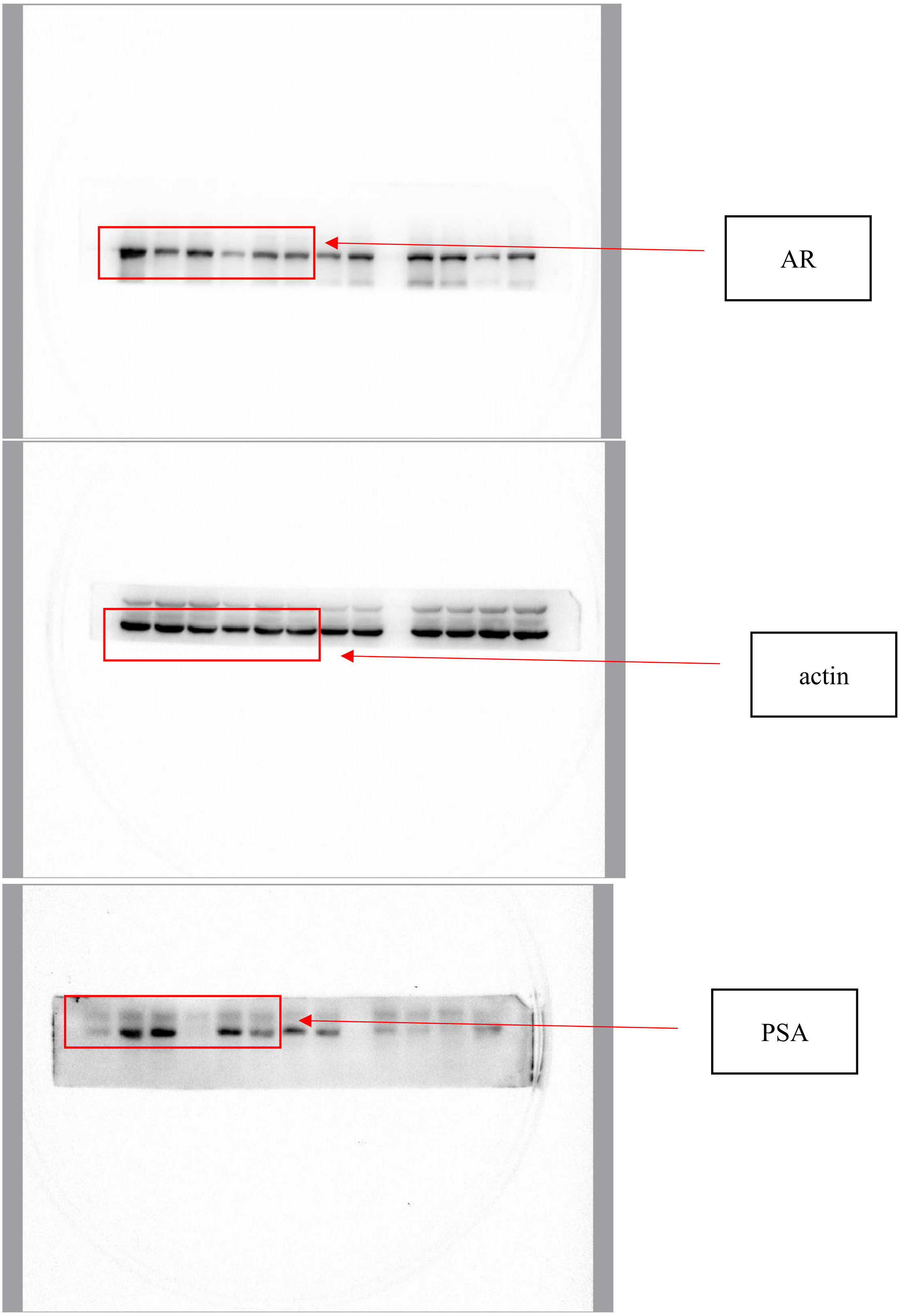

Figure 2G

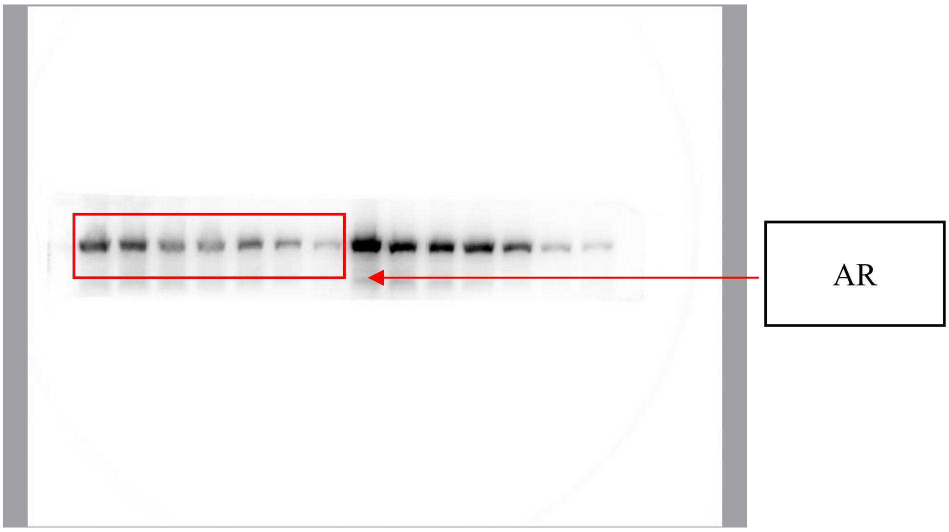

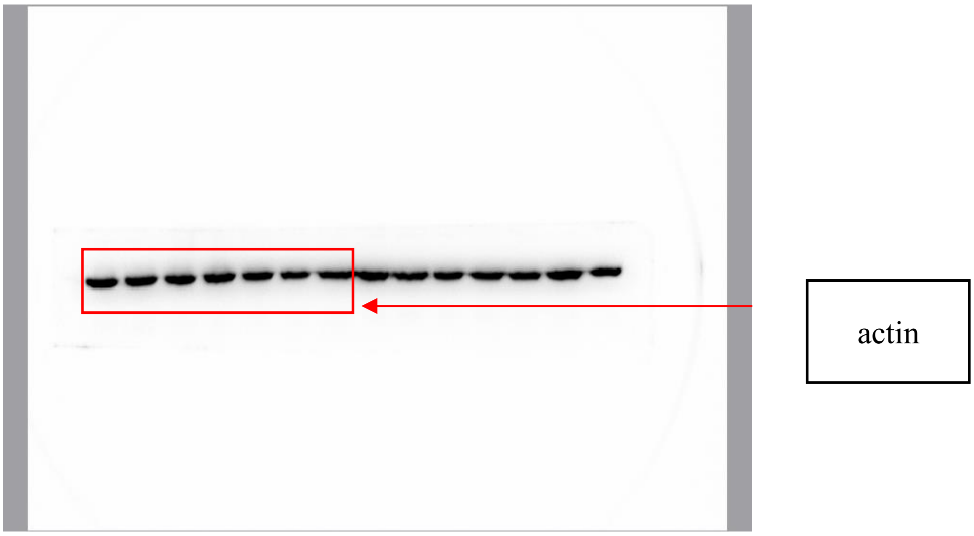

Figure 2H

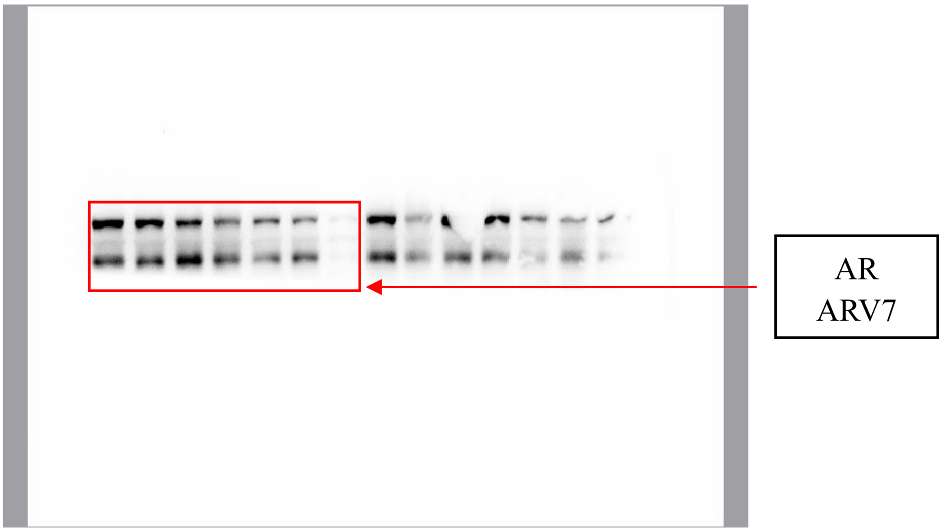

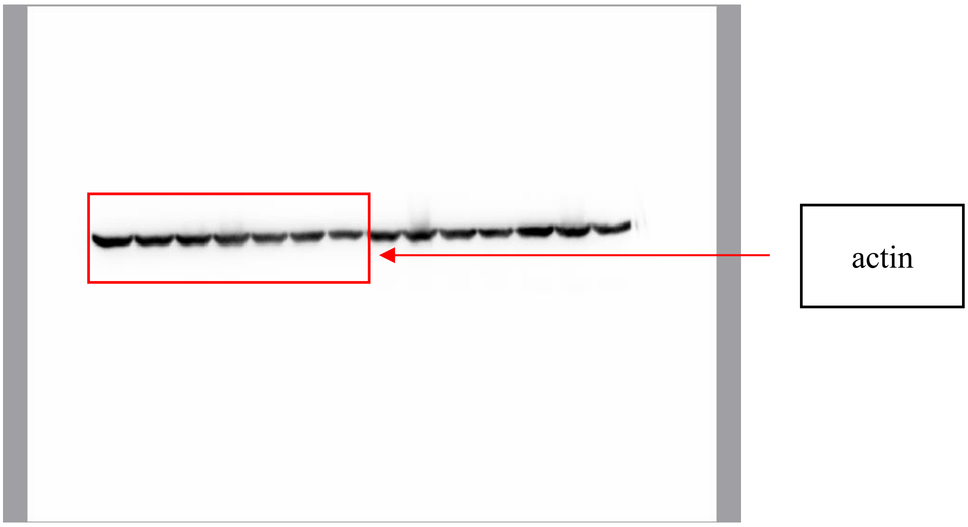
actin

Figure 5

Figure 5A

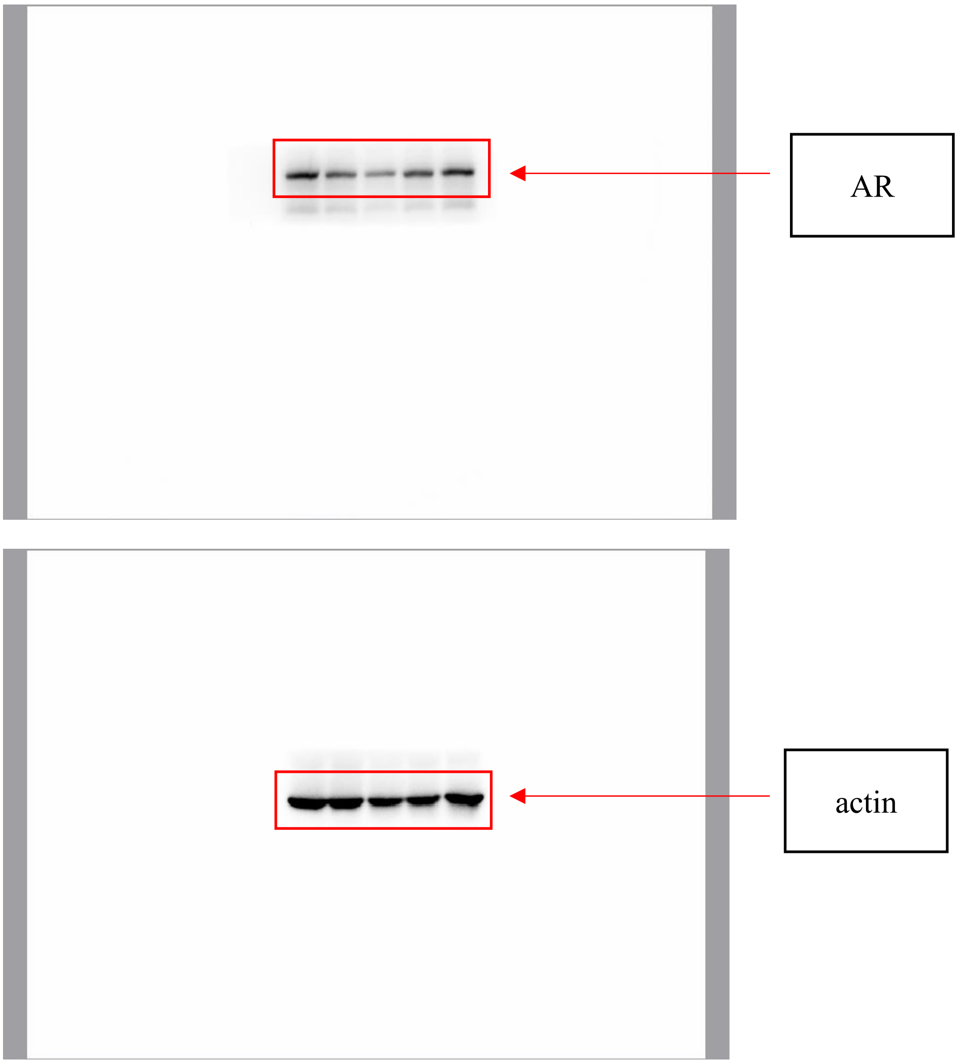

Figure 5B

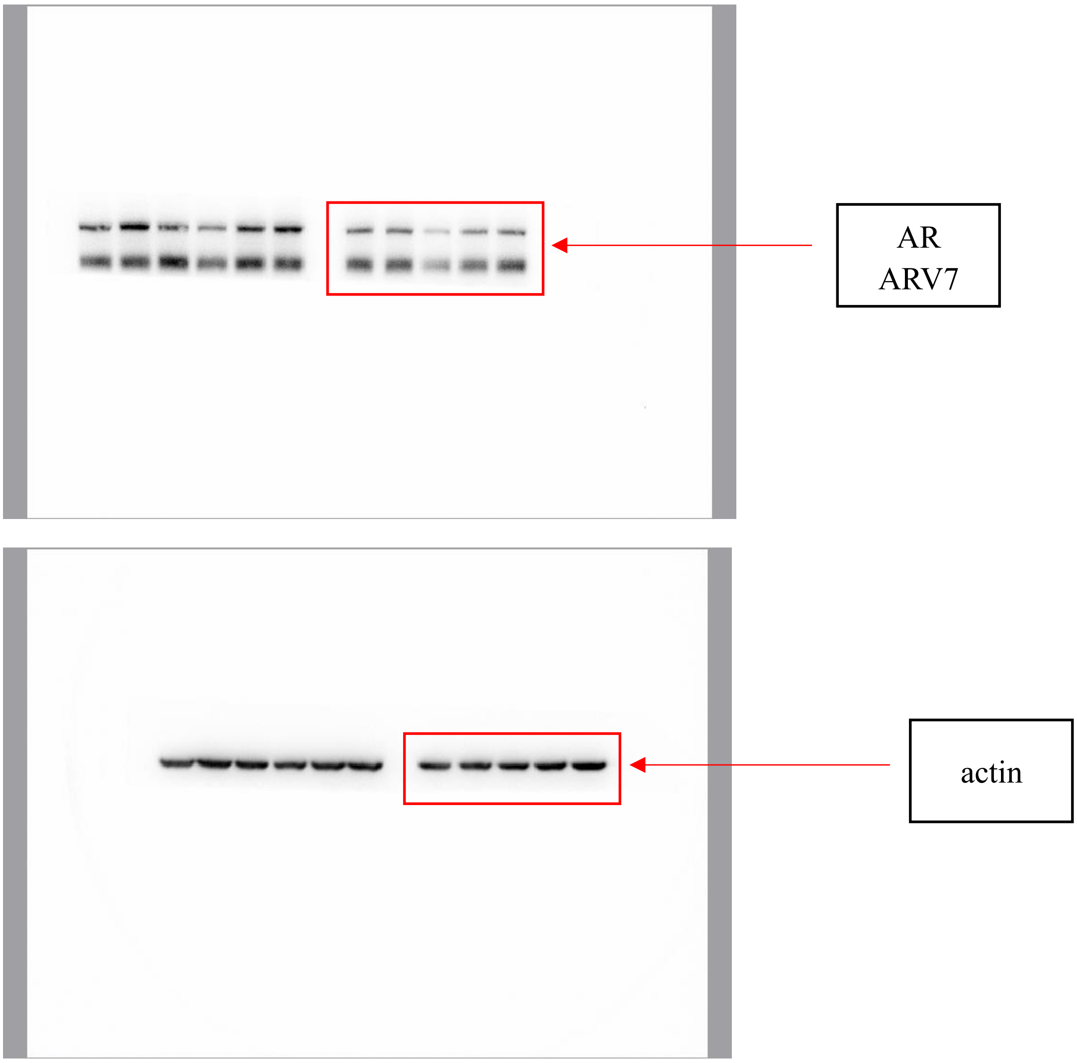

Figure 5C

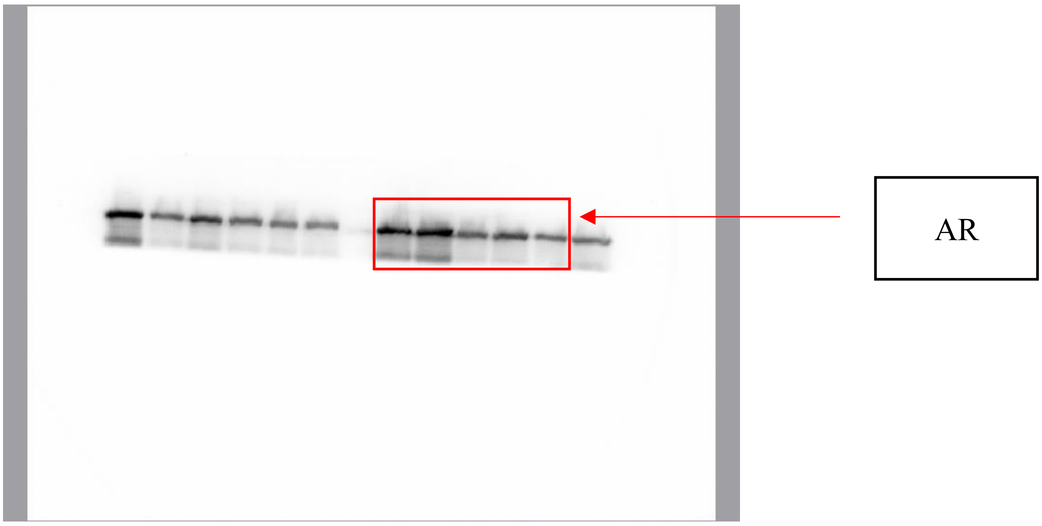

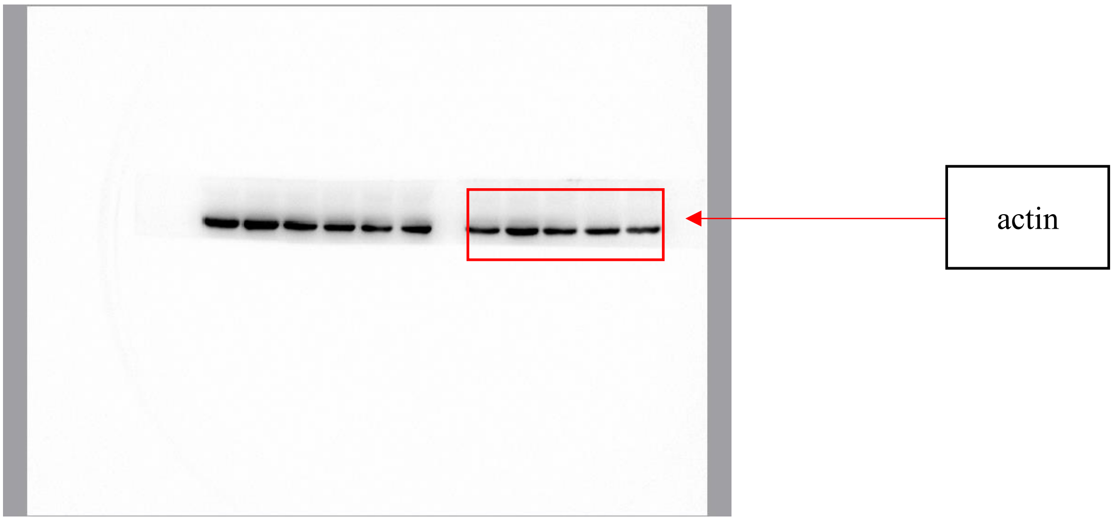

Figure 5D

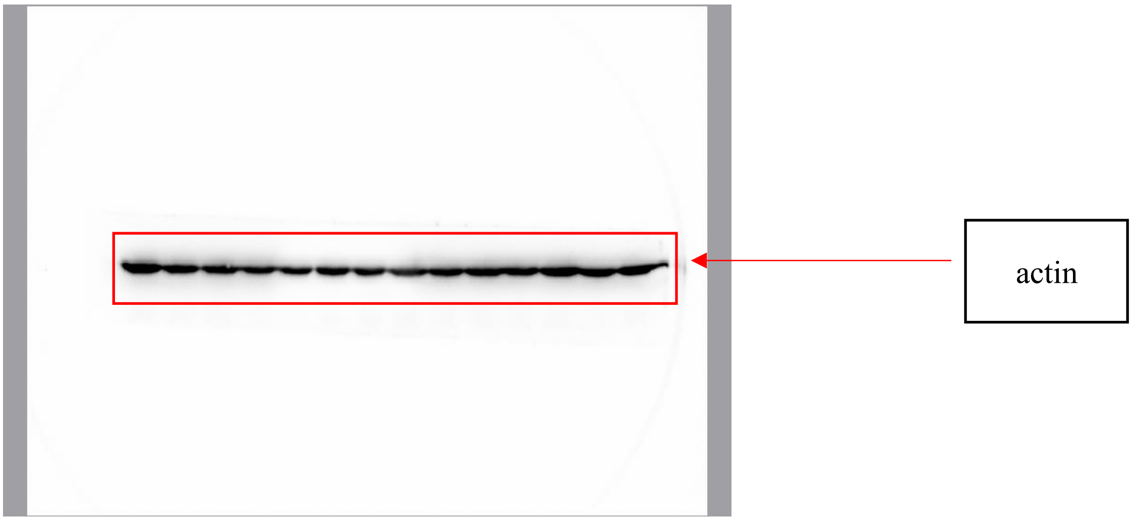

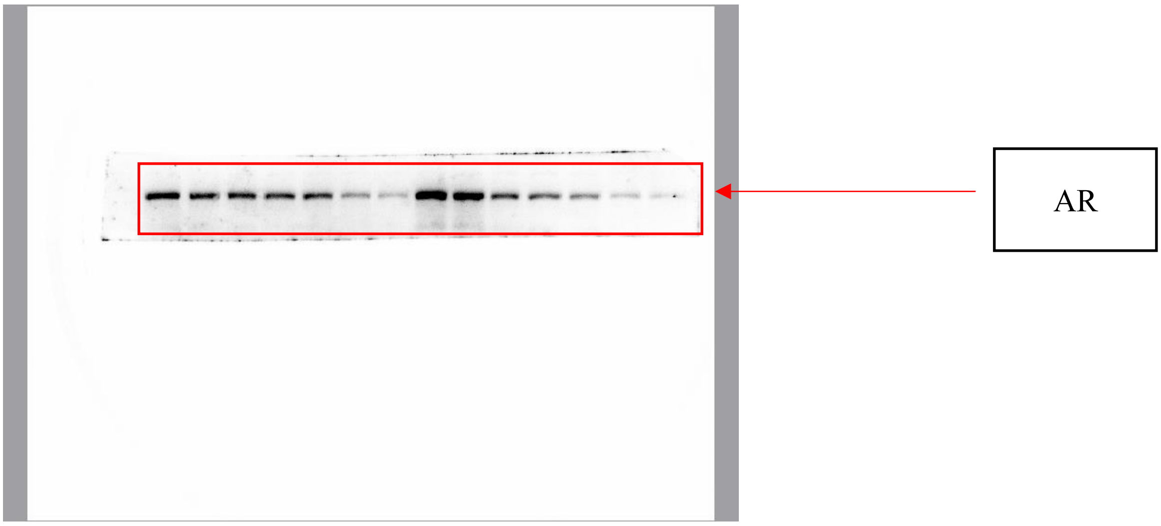

Figure 5E

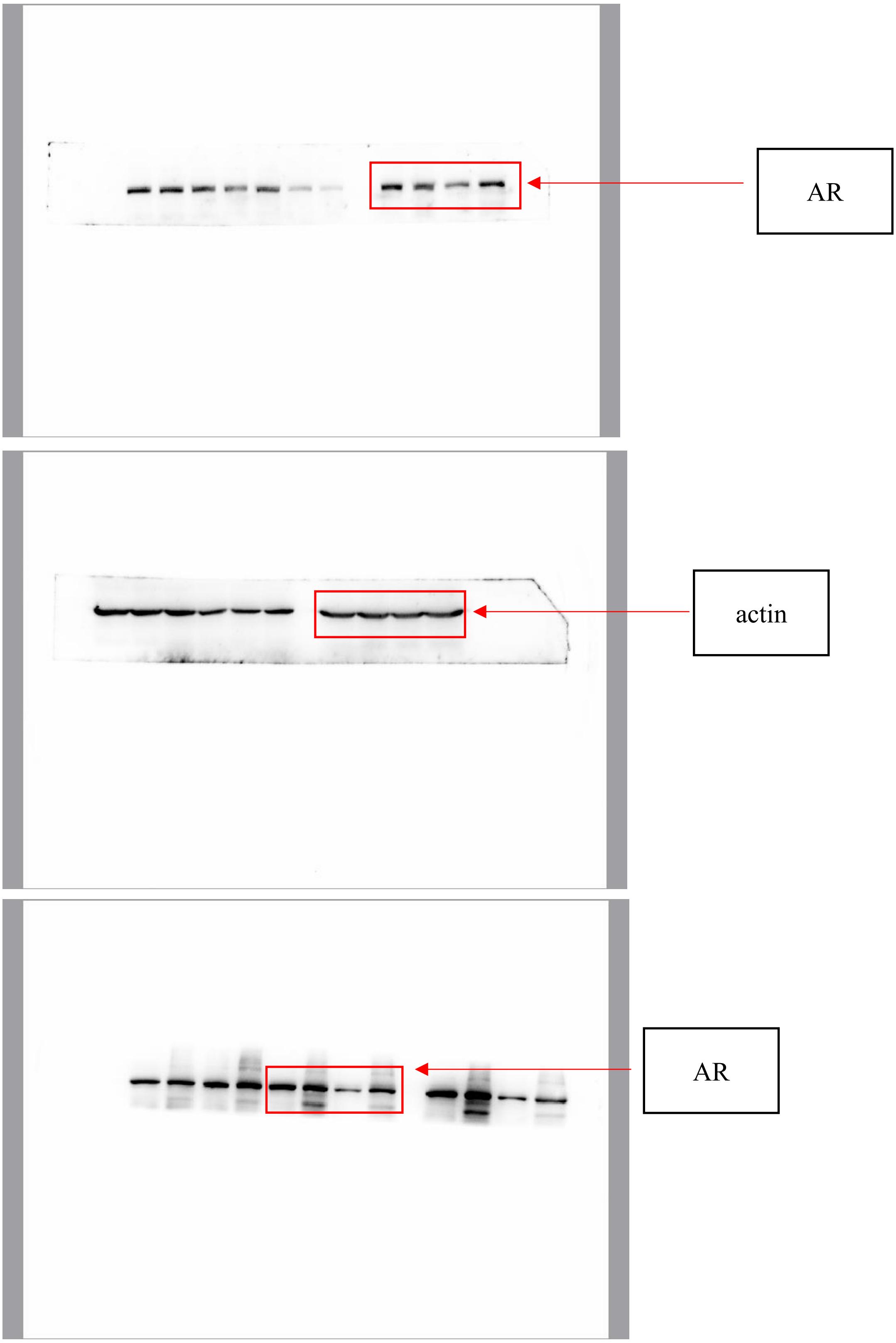

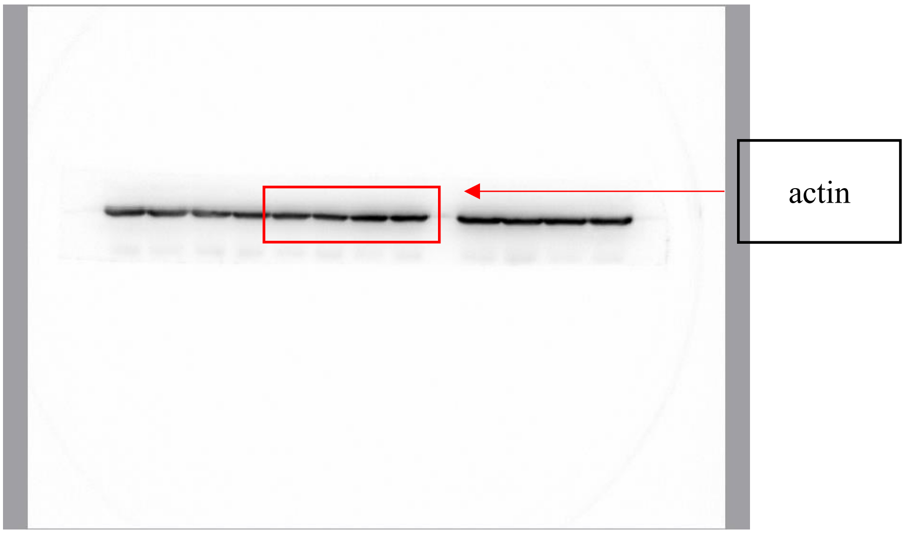

Figure 5F

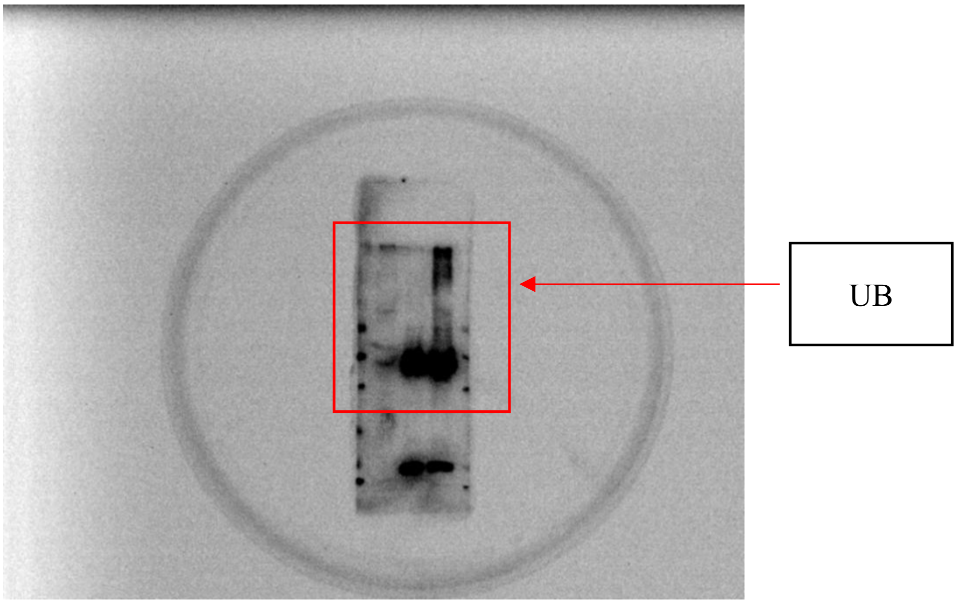

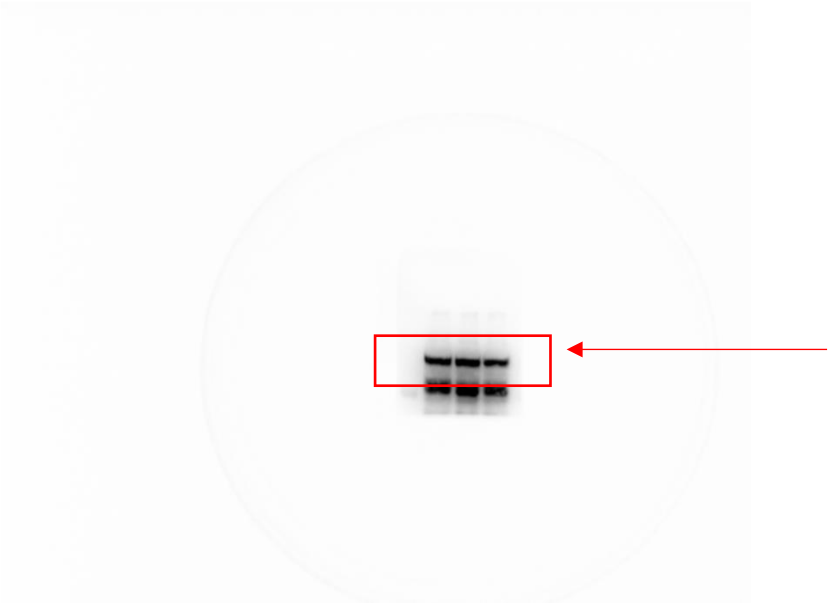

AR

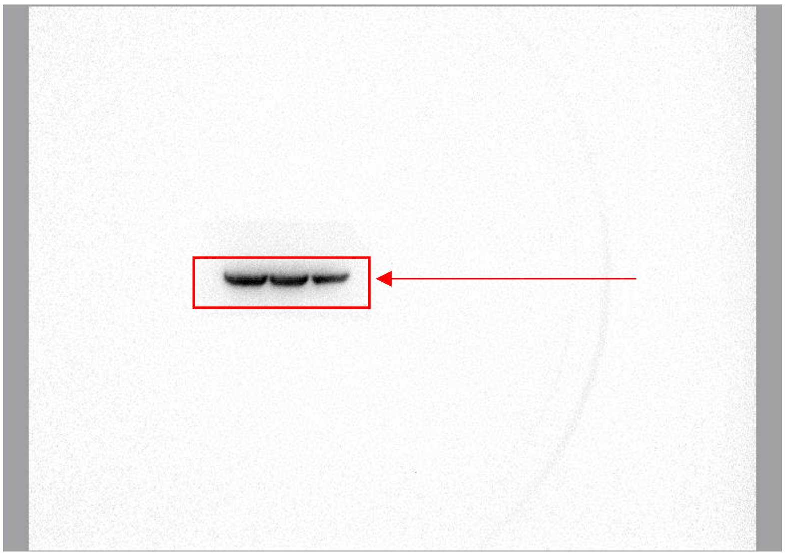

actin

Figure 6

Figure 6D

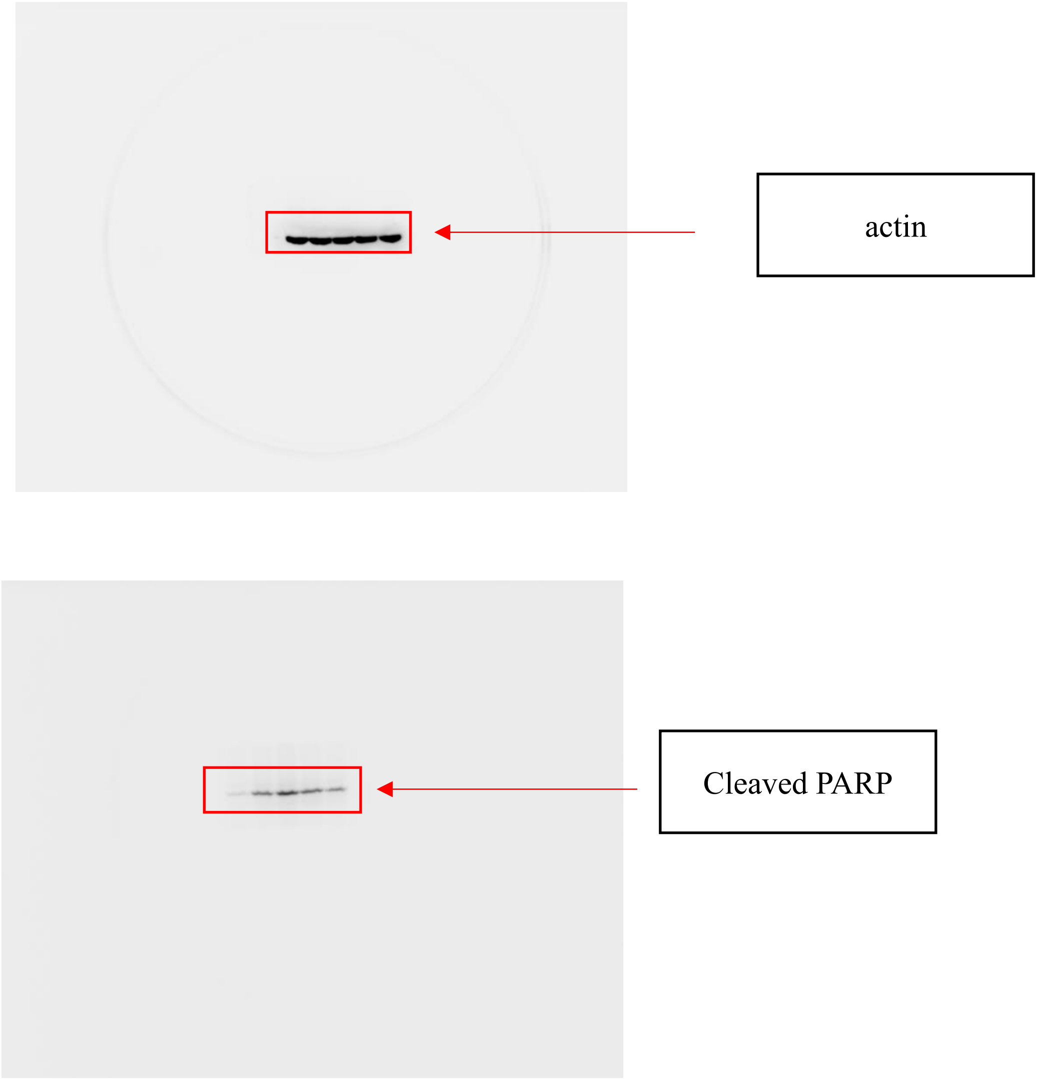

Figure 6E

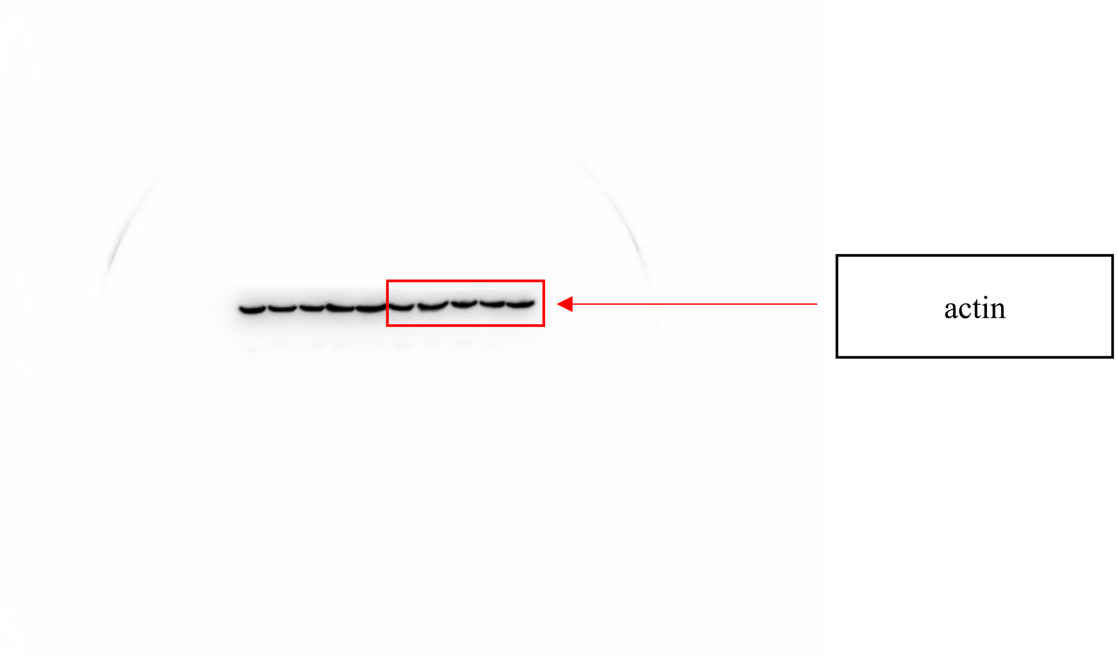

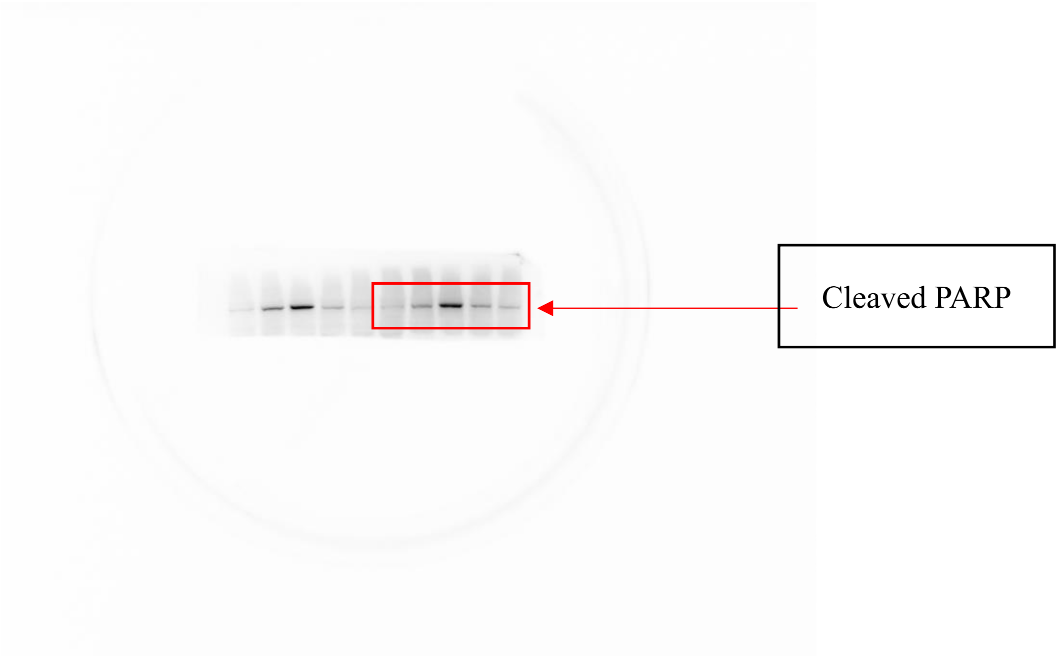

Figure 7

Figure 7D

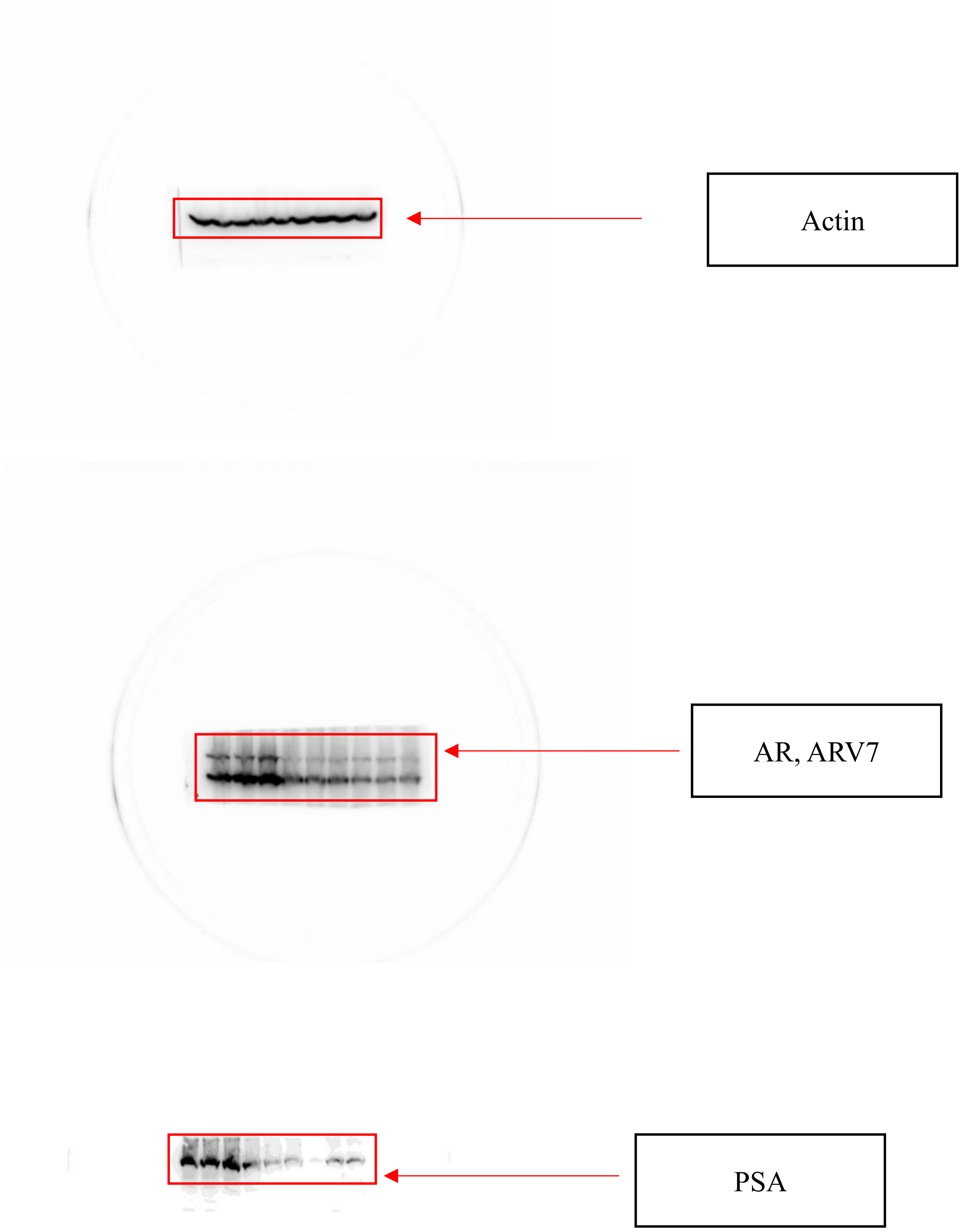

Figure 8

Figure 8C

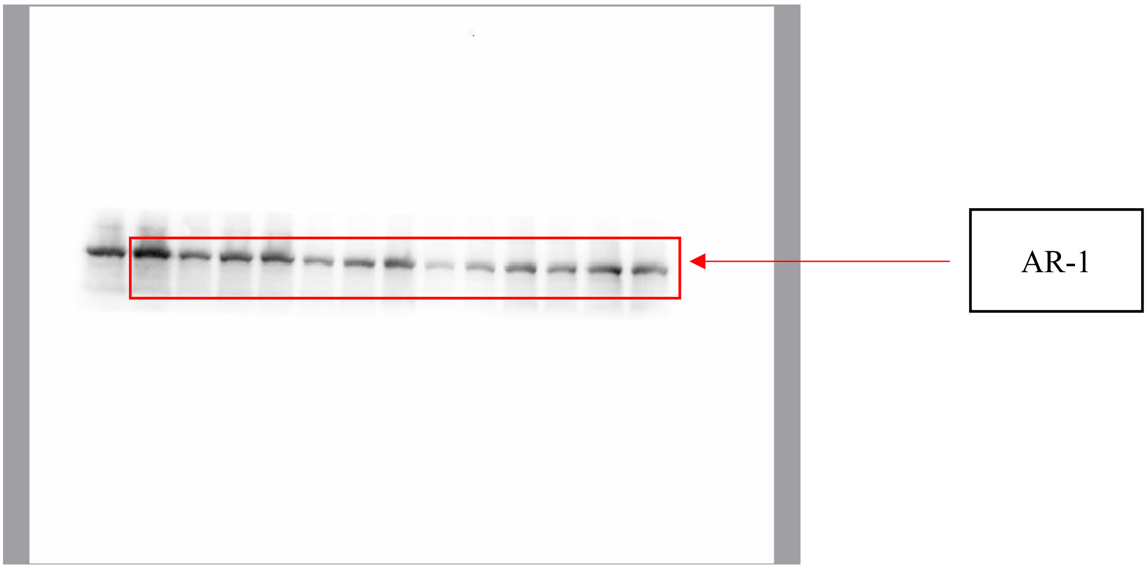

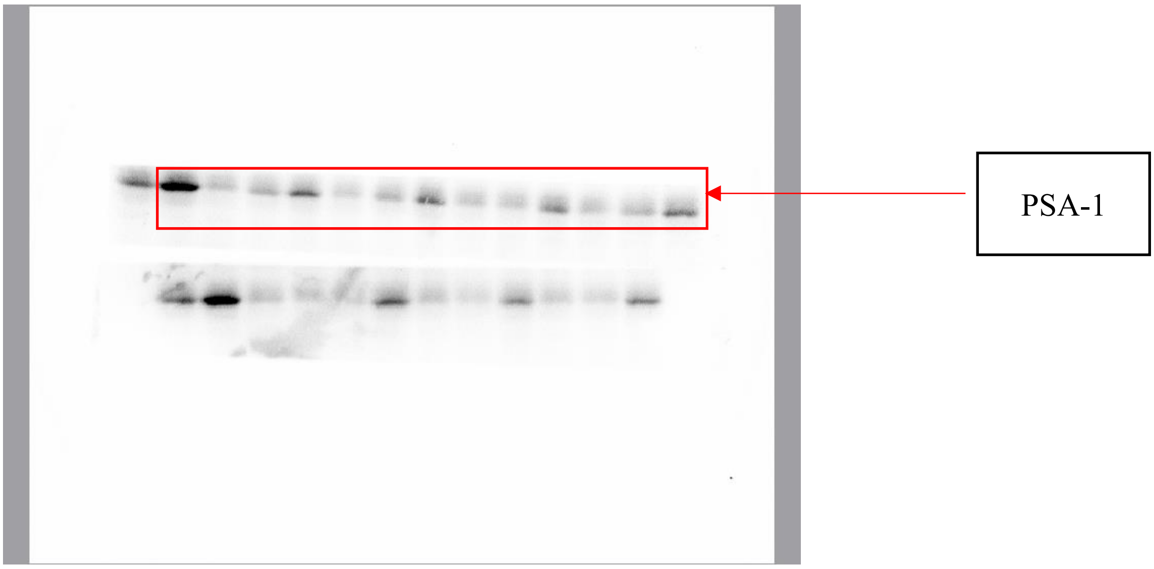

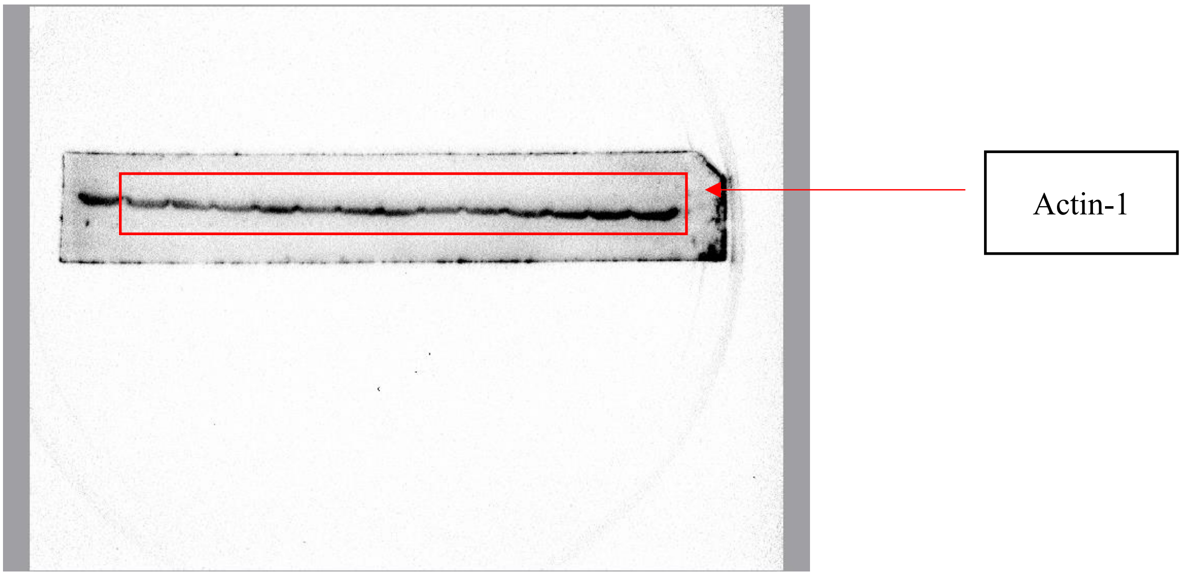
Actin-1

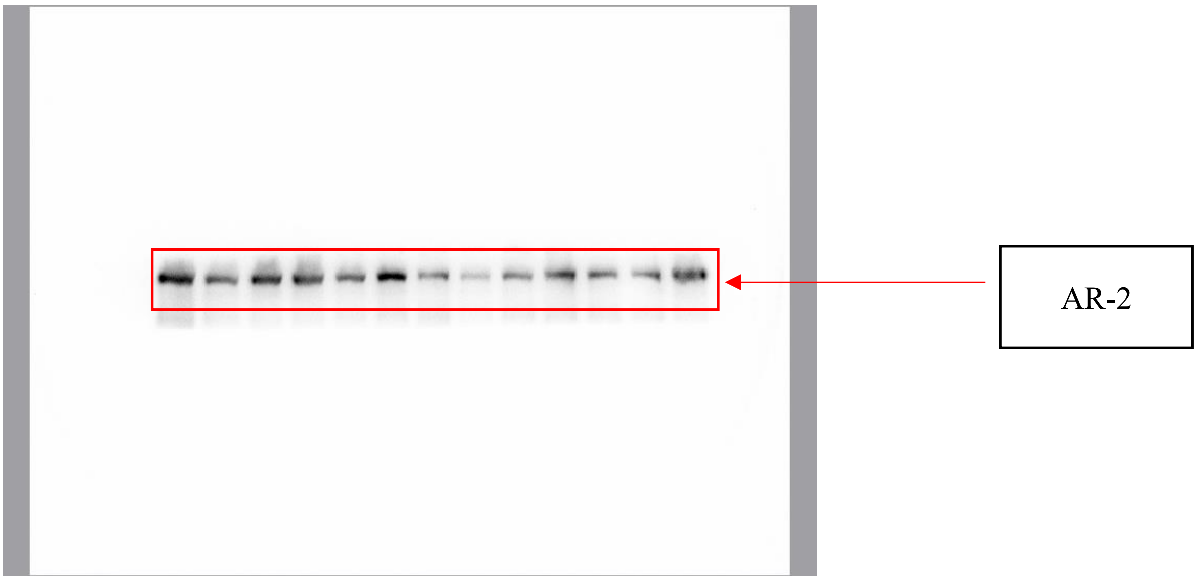
AR-2

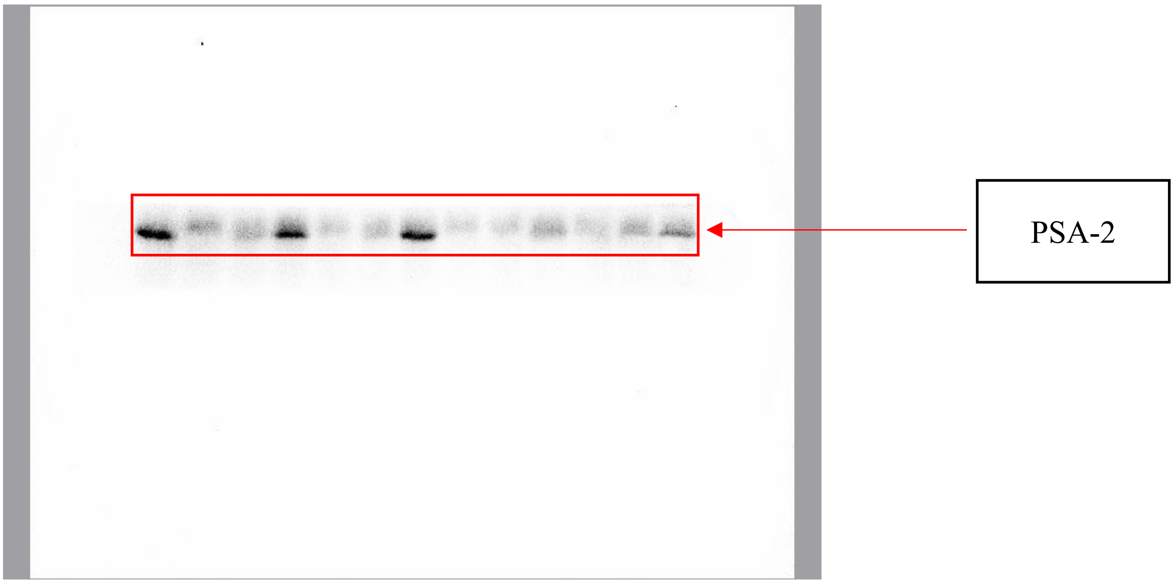
PSA-2

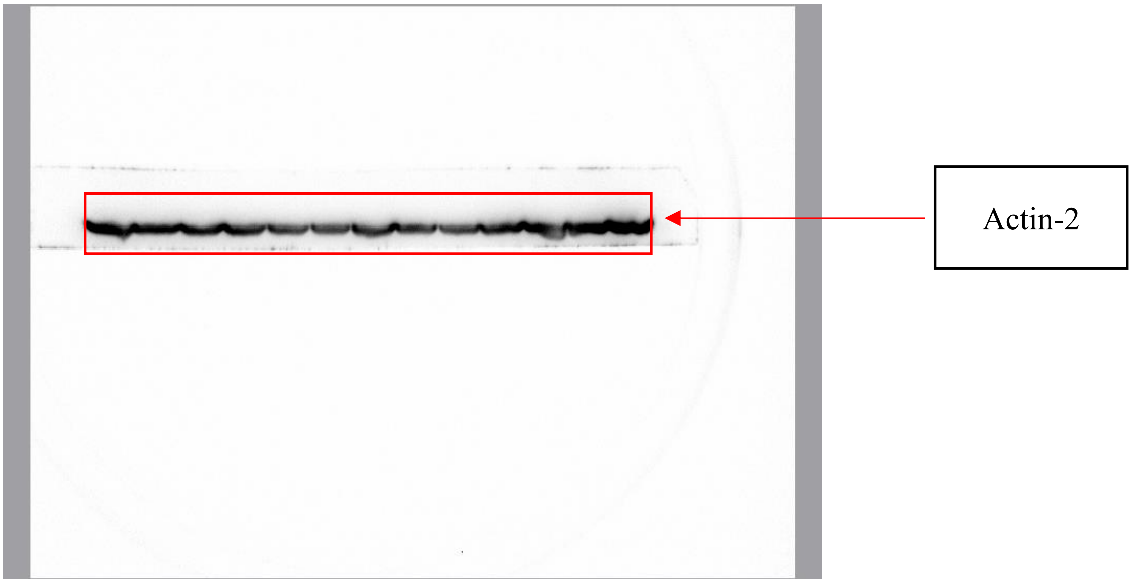
Actin-2

Figure 2-figure supplement 3

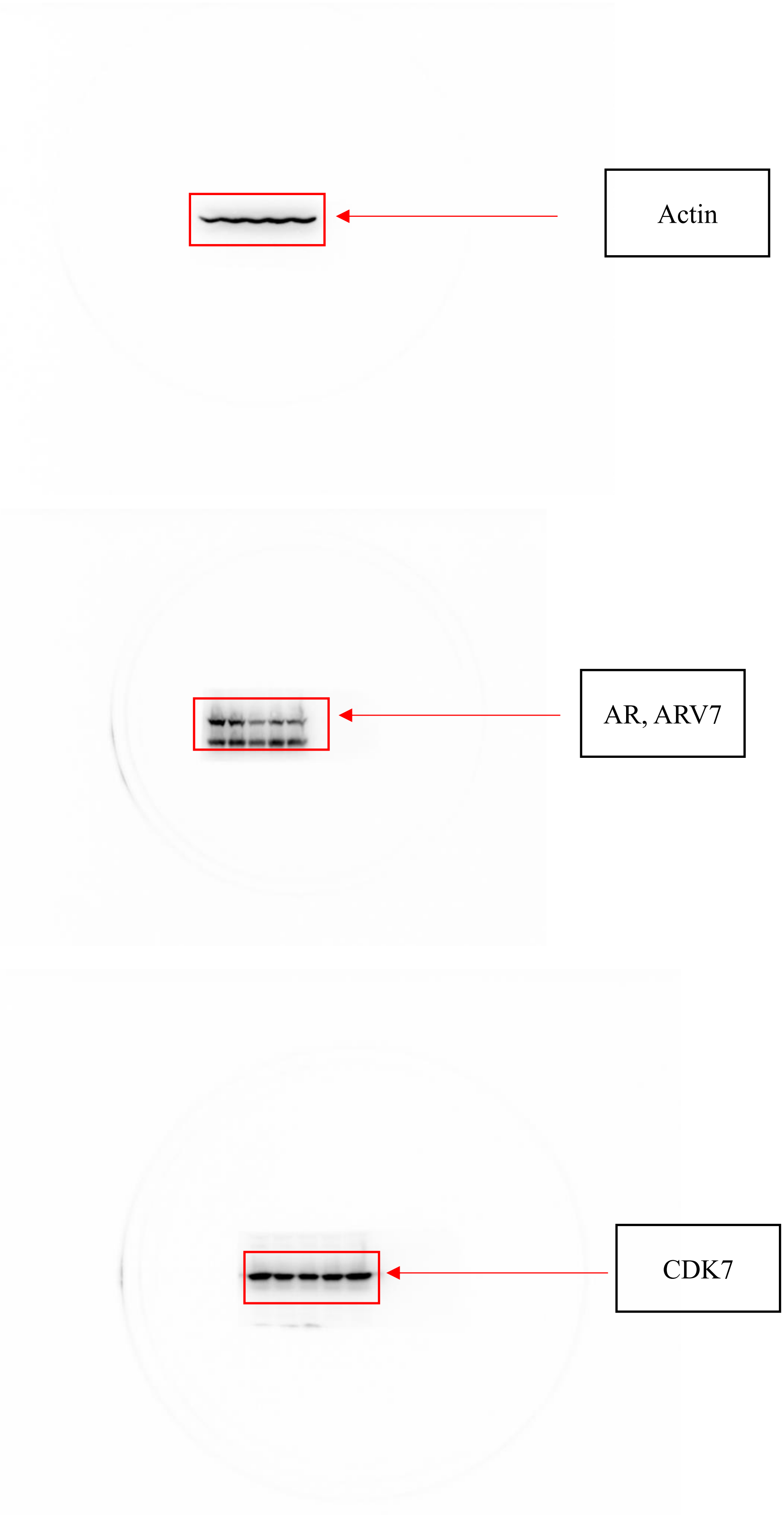

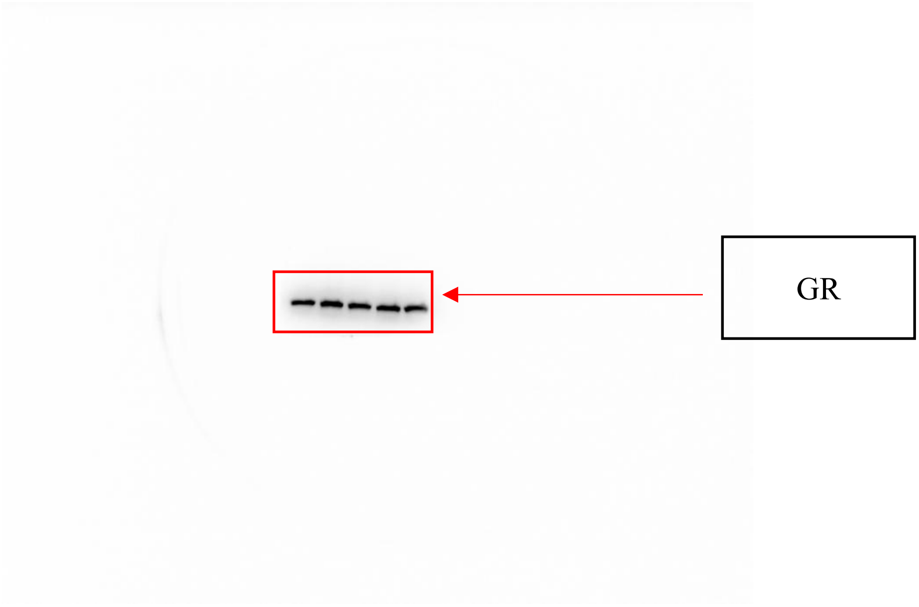
GR

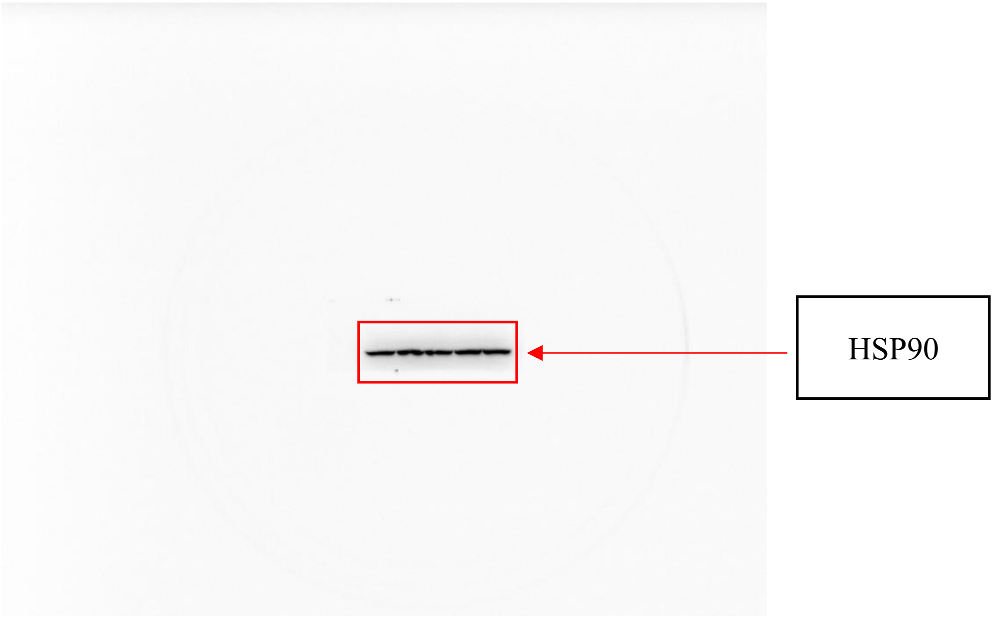
HSP90

Figure 4-figure supplement 2

Figure 4-figure supplement 2A

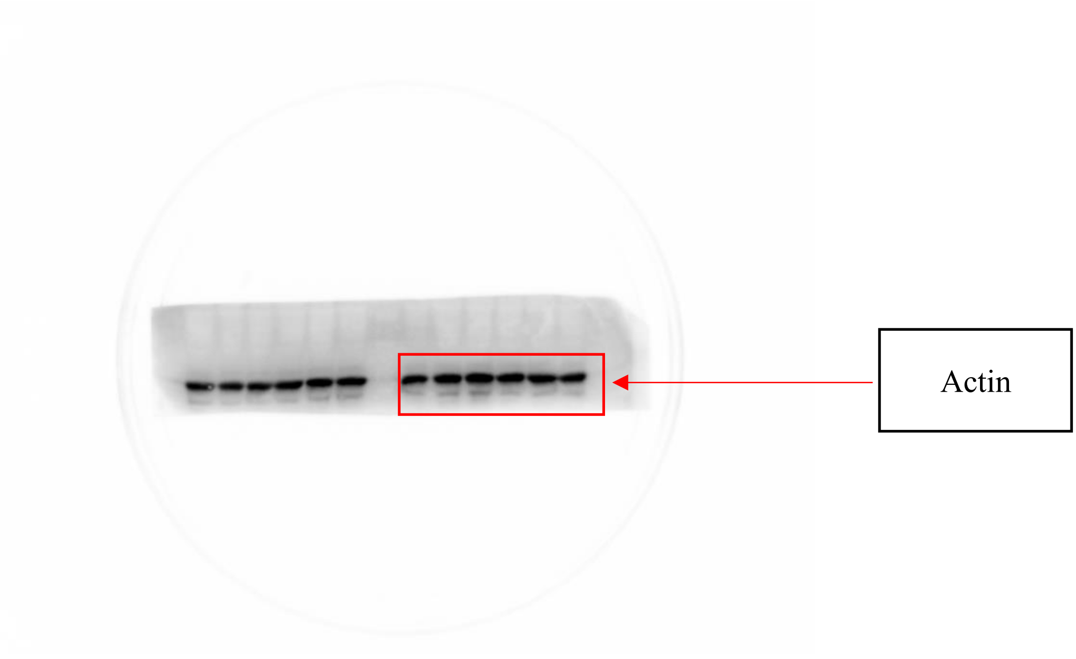

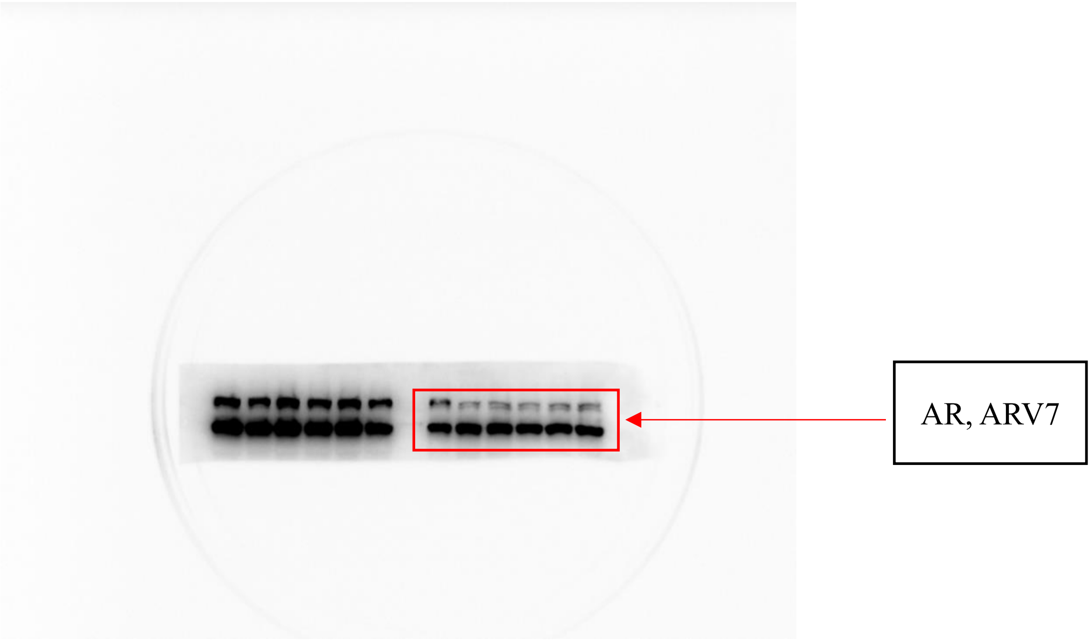

Figure 4-figure supplement 2D

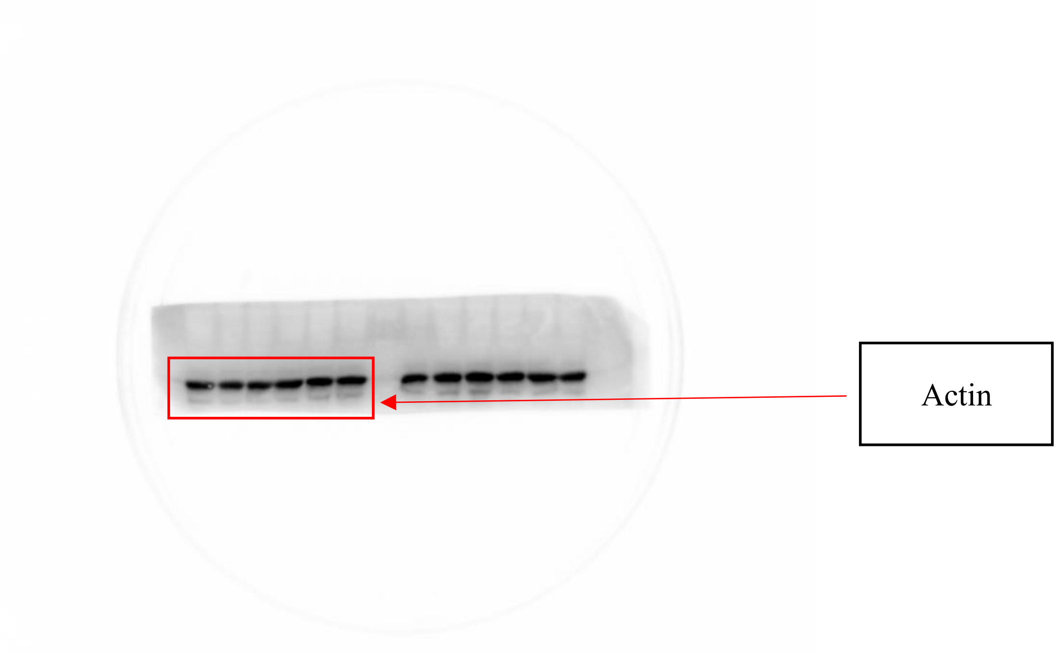

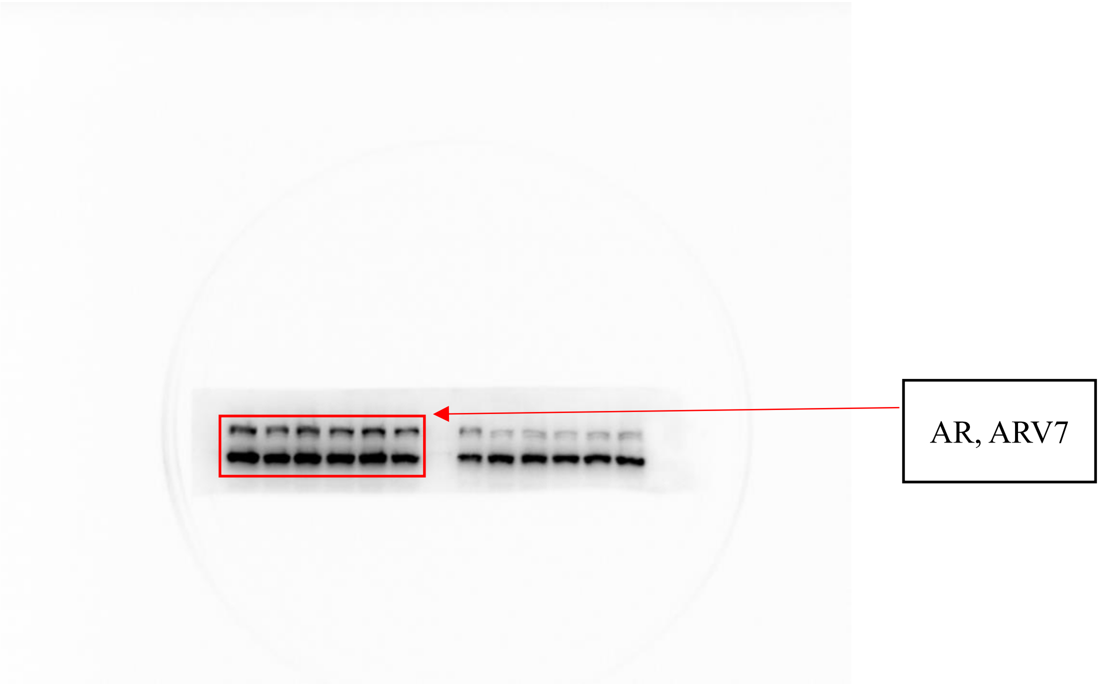

Figure 6-figure supplement 1

Figure 6-figure supplement 1A

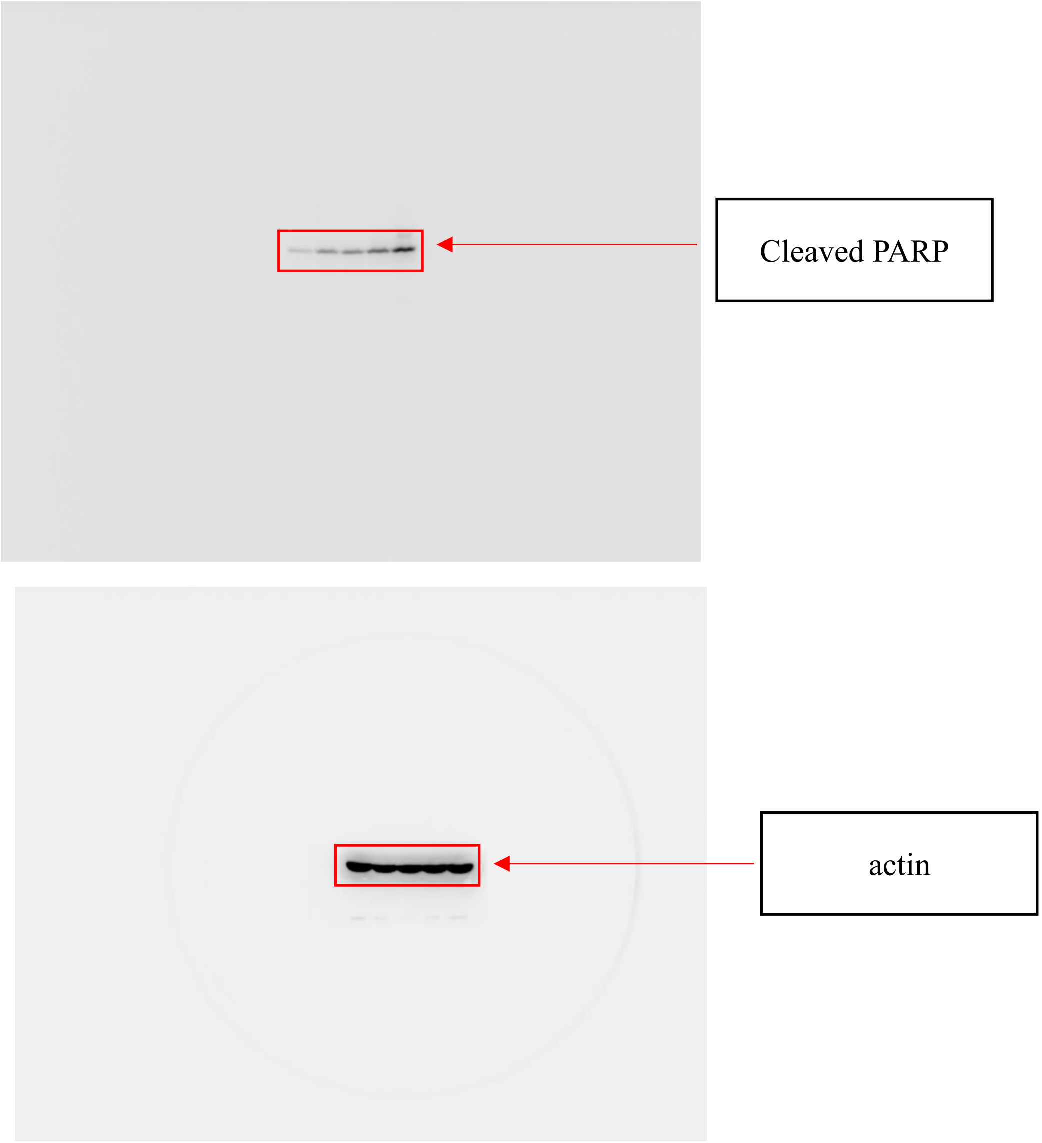

Figure 6-figure supplement 1B

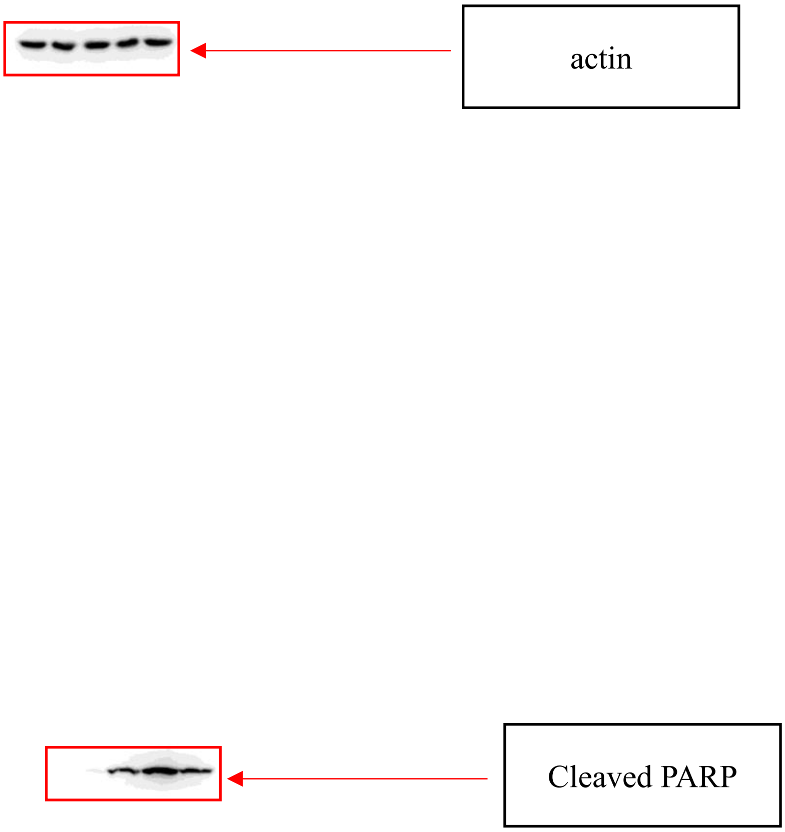

Figure 6-figure supplement 1E

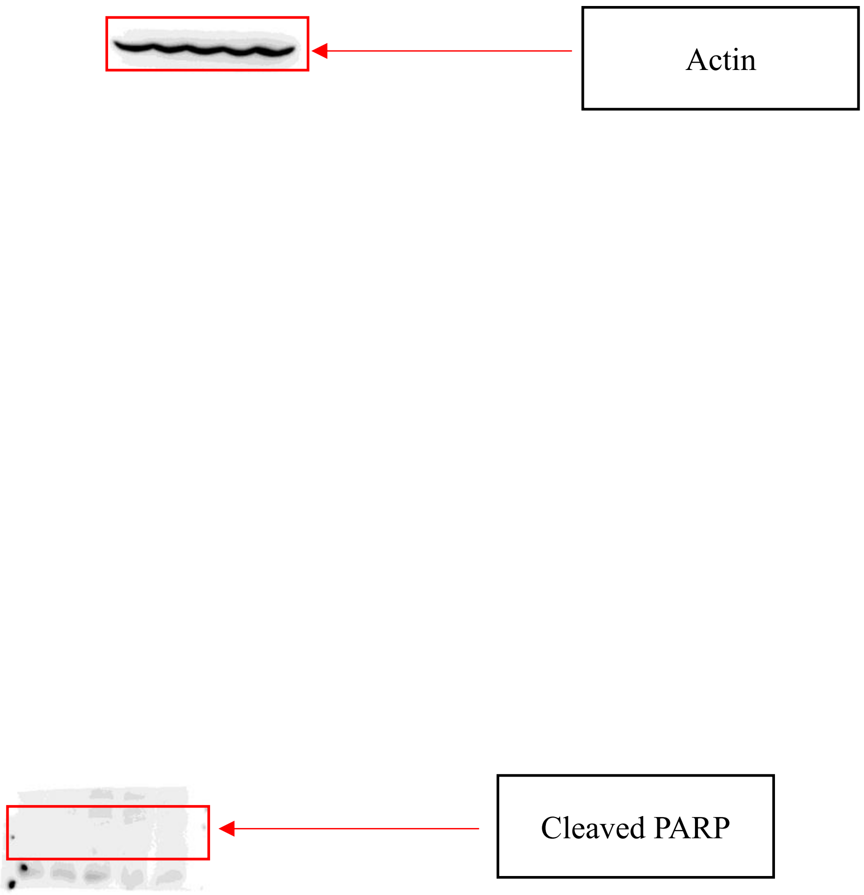

Figure 8-figure supplement 2

Figure 8-figure supplement 2B

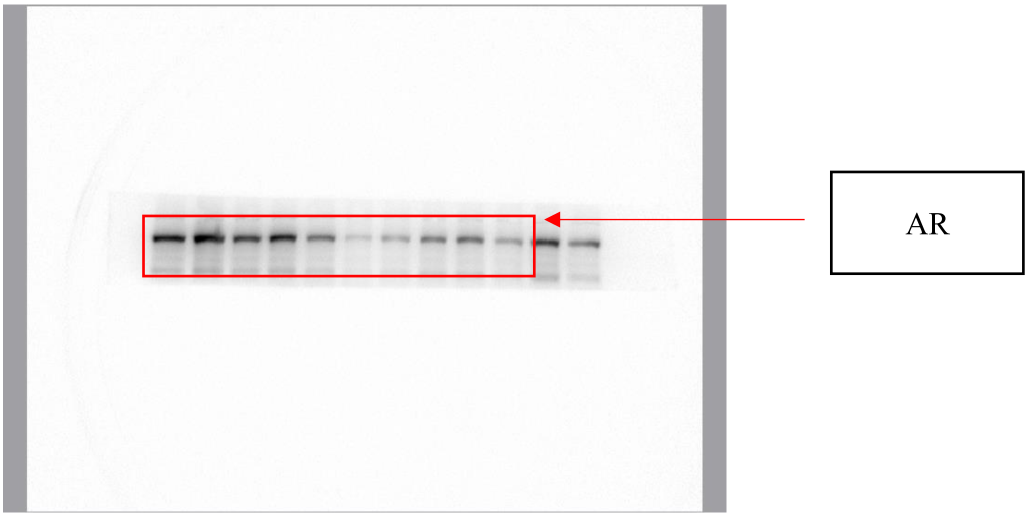

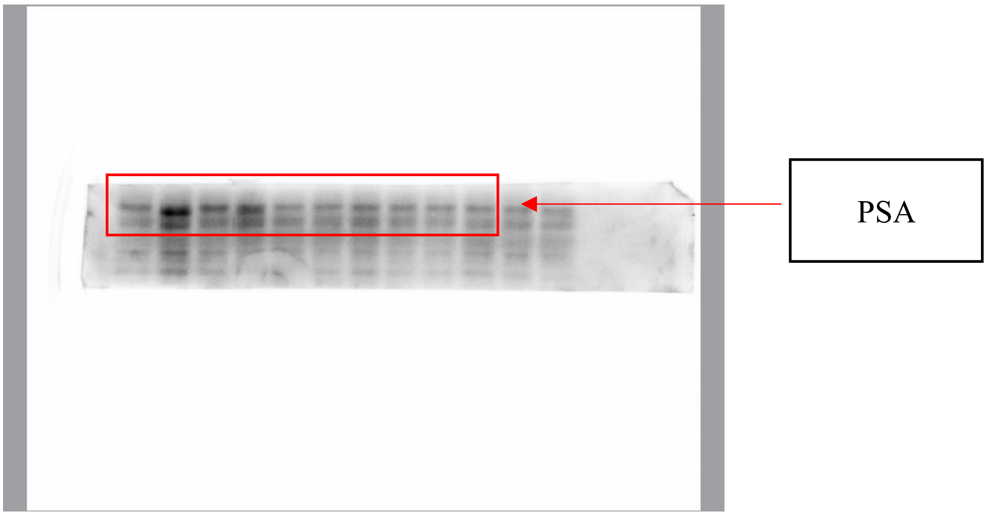

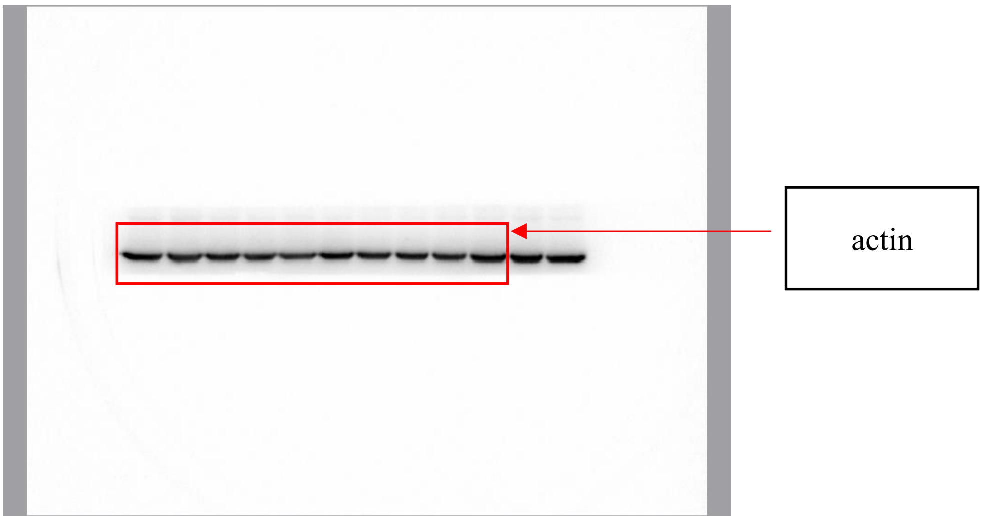

Supplement: Source data 1. [file elife-70700-data1.pdf]
