# Supplementary material for: Selective androgen receptor degrader (SARD) to overcome antiandrogen resistance in castration-resistant prostate cancer
Source: eLife. 2023 Jan 19;12:e70700. doi: 10.7554/eLife.70700 (PMC9901937; doi:10.7554/eLife.70700)

Sample Name: Z1908 OK Injection Date 2004-Nov-13
Acquisition Method: SB15_3PN.M Location P2-B-06

Raw data file name: D:\MYWORK~1\VRABOT~1\11_12_08\SAMPL014.D
The method for the Gradient Sample using short rapid resolution HT Cartridge ZORBAX SB-C18 4.6x15 mm (p/n 821975-932). For testing purity of syntez.

#	Time	Area %
1	0.722	94.62
2	0.768	5.38

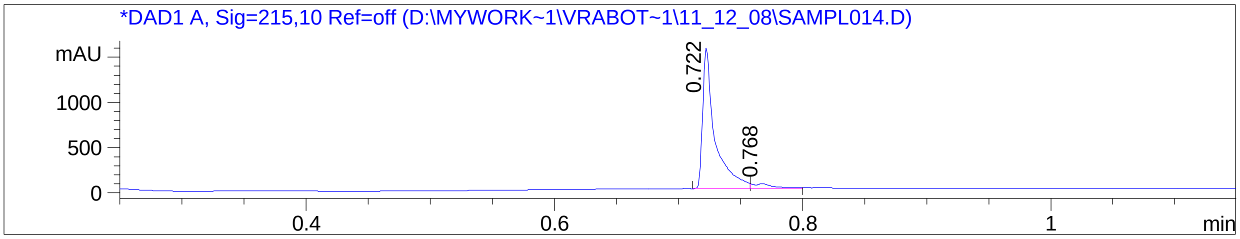

#	Time	Area %
1	0.764	100.00

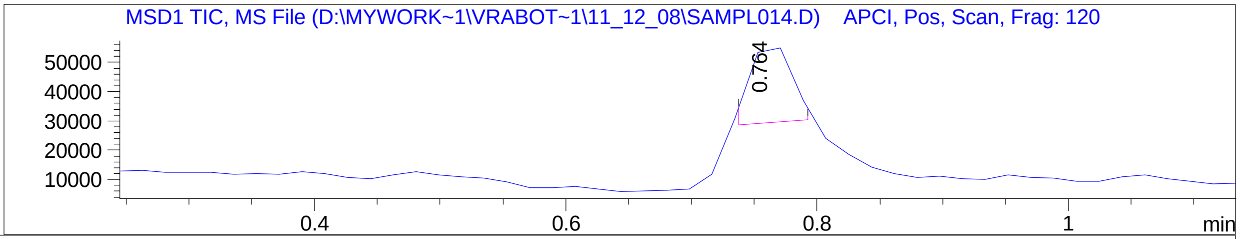

Ret. Time 0.764

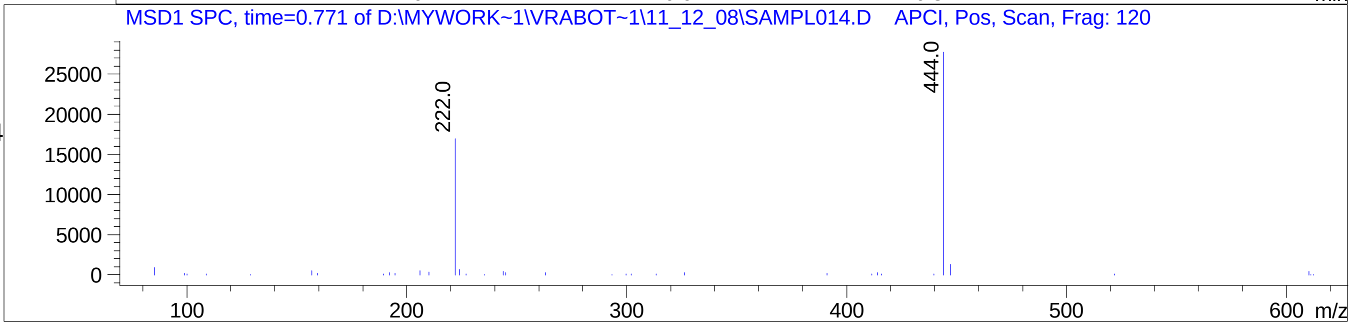

Supplement: Source data 2. [file elife-70700-data2.zip › Supplementary Material_source_data/Figure 1-figure supplement 1 & Supplementary1a-source/Z11.PDF]
