# Supplementary material for: Selective androgen receptor degrader (SARD) to overcome antiandrogen resistance in castration-resistant prostate cancer
Source: eLife. 2023 Jan 19;12:e70700. doi: 10.7554/eLife.70700 (PMC9901937; doi:10.7554/eLife.70700)
Supplement: Source data 2. [file elife-70700-data2.zip › Supplementary Material_source_data/Figure 1-figure supplement 1 & Supplementary1a-source/Z12.PDF]

MaxPeak: 100.00%
Ret_Time: 0.711 min

1968818	OK

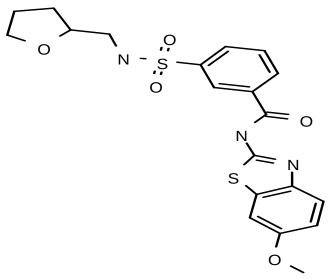

mw = 447.54

DAD1 A, Sig=215,10 Ref=off (D:\DATE\11_14\11_13_07\SAMPL050.D)

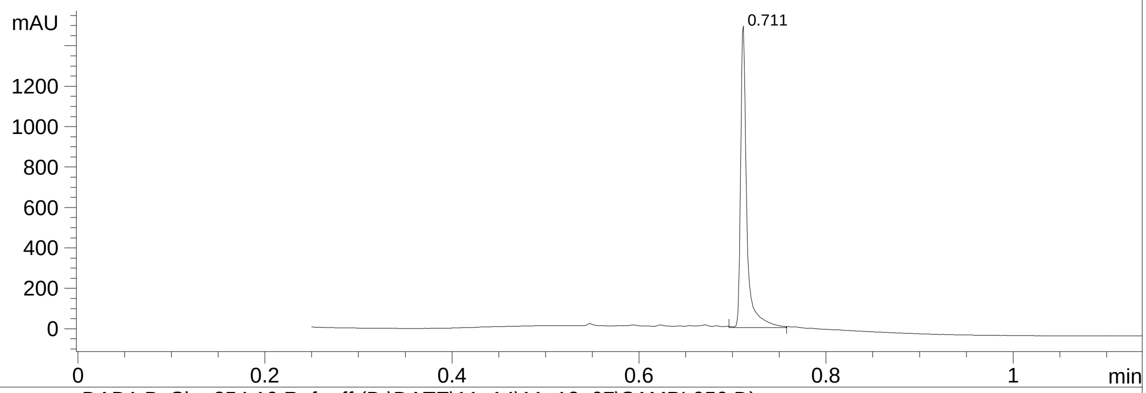

#	Time	Area%
1	0.711	100.00

DAD1 B, Sig=254,10 Ref=off (D:\DATE\11_14\11_13_07\SAMPL050.D)

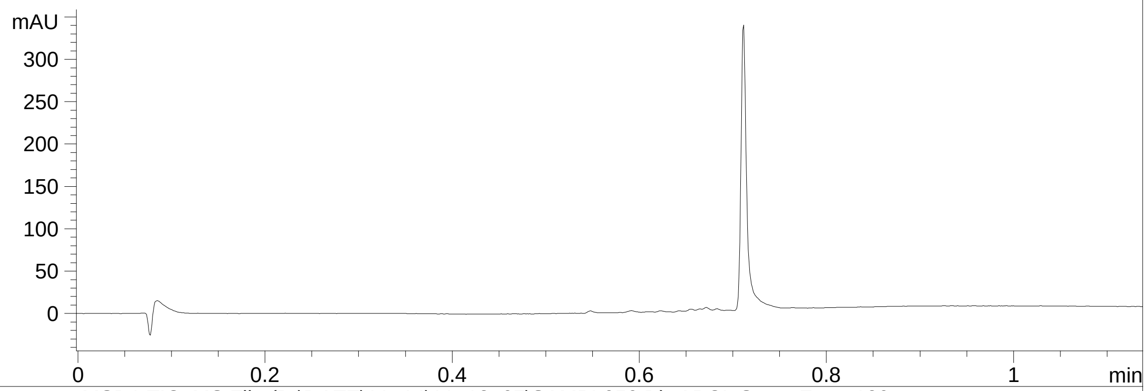

MSD1 TIC, MS File (D:\DATE\11_14\11_13_07\SAMPL050.D) APCI, Scan, Frag: 120

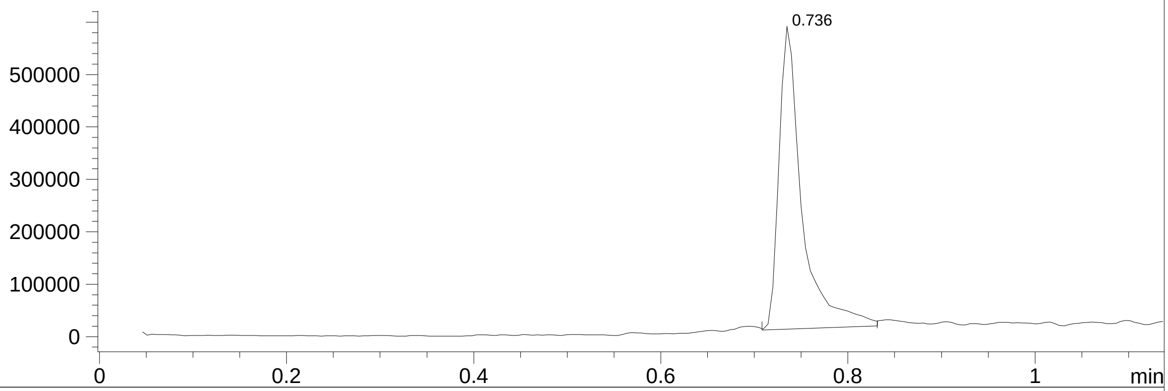

*MSD1 SPC, time=0.735 of D:\DATE\11_14\11_13_07\SAMPL050.D APCI, Scan, Frag: 120

RT 0.736

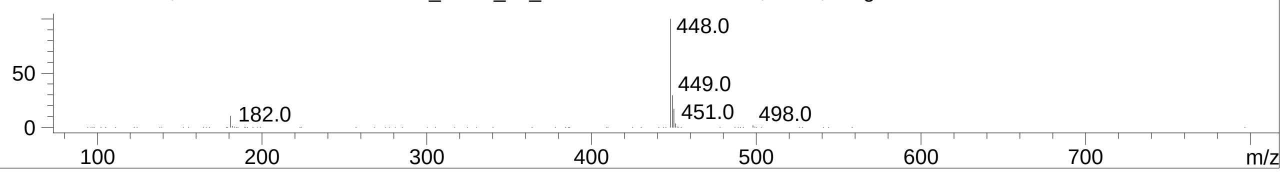

Inj.Date 11/14/2007 E P2-F-06 B03075 Acq. Method C:\HPCHEM\->
