# Supplementary material for: Selective androgen receptor degrader (SARD) to overcome antiandrogen resistance in castration-resistant prostate cancer
Source: eLife. 2023 Jan 19;12:e70700. doi: 10.7554/eLife.70700 (PMC9901937; doi:10.7554/eLife.70700)
Supplement: Source data 2. [file elife-70700-data2.zip › Supplementary Material_source_data/Figure 1-figure supplement 1 & Supplementary1a-source/Z13.PDF]

MaxPeak: 99.39%
Ret_Time: 0.543 min

1347021 OK

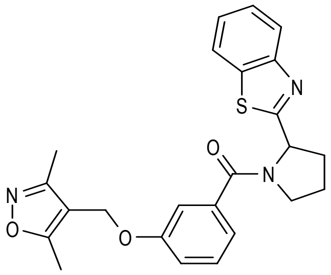

mw = 433.53

#	Time	Area%
1	0.445	0.61
2	0.543	99.39

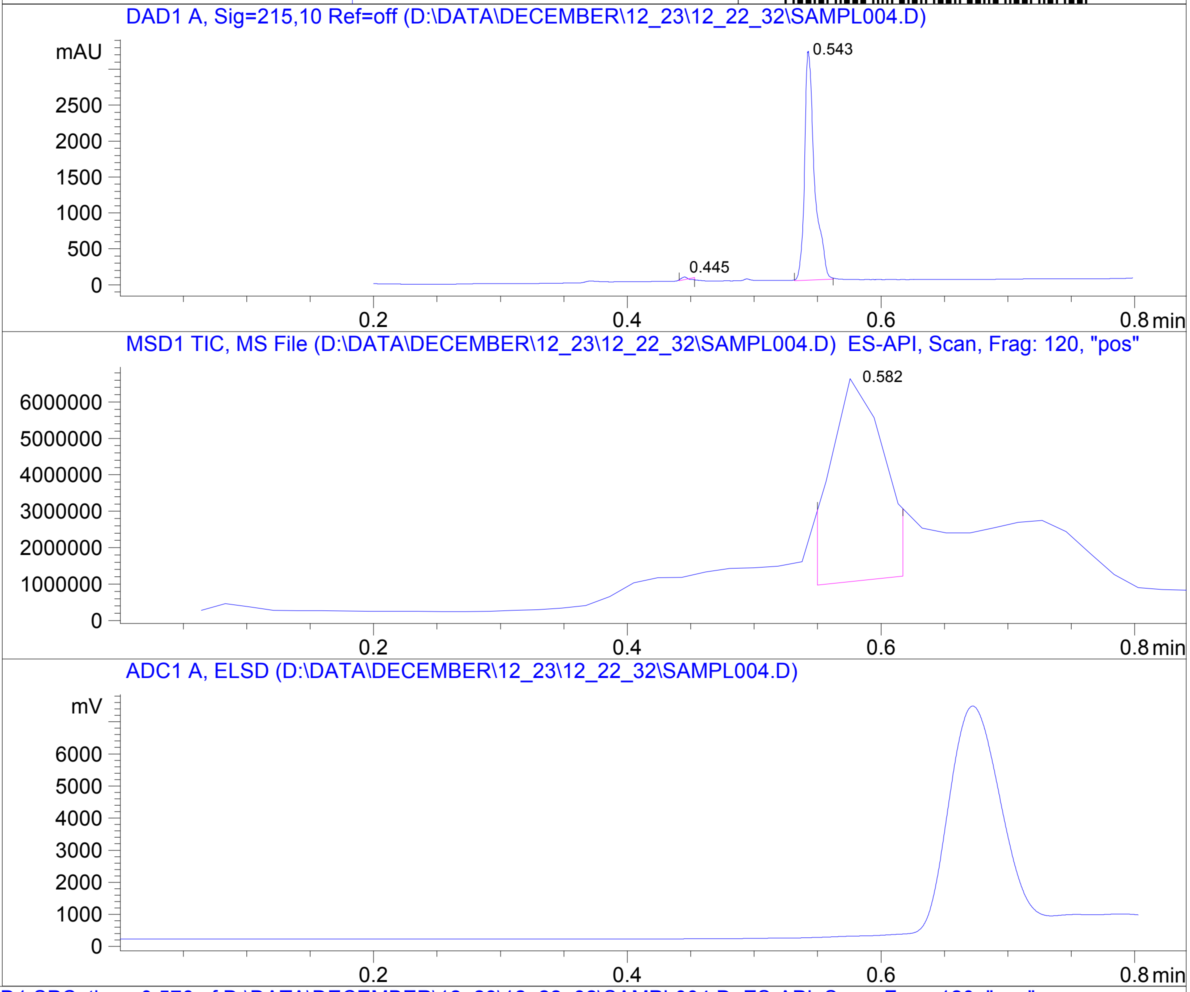

RT 0.582

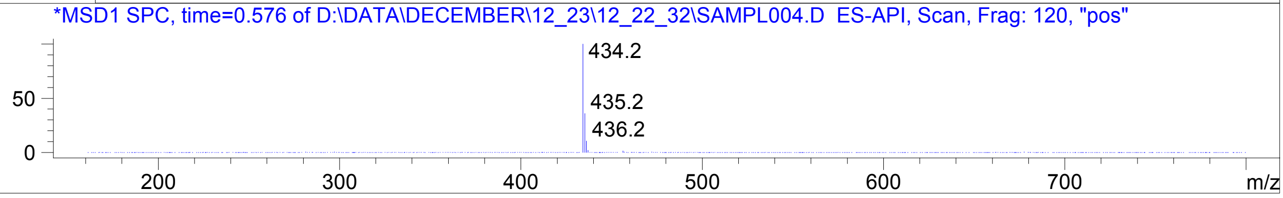

Inj.Date 12/23/2006 L P1-A-05 Acq. Method C:\Chem32\->
