# Supplementary material for: Selective androgen receptor degrader (SARD) to overcome antiandrogen resistance in castration-resistant prostate cancer
Source: eLife. 2023 Jan 19;12:e70700. doi: 10.7554/eLife.70700 (PMC9901937; doi:10.7554/eLife.70700)
Supplement: Source data 2. [file elife-70700-data2.zip › Supplementary Material_source_data/Figure 1-figure supplement 1 & Supplementary1a-source/Z14.PDF]

MaxPeak: 94.41%
Ret_Time: 0.771 min

1892367	OK

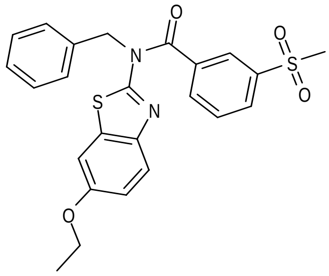

mw = 466.58

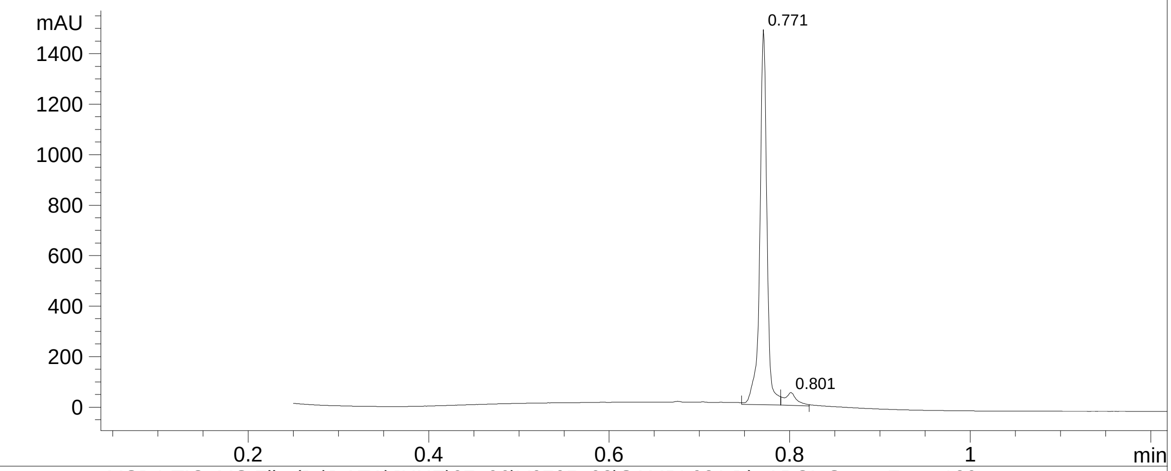

#	Time	Area%
1	0.771	94.41
2	0.801	5.59

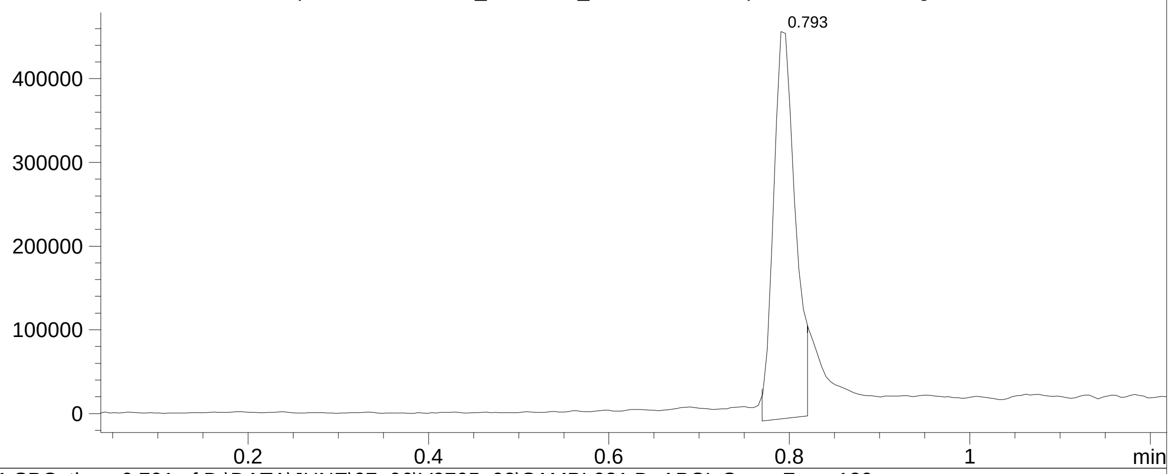

RT 0.793

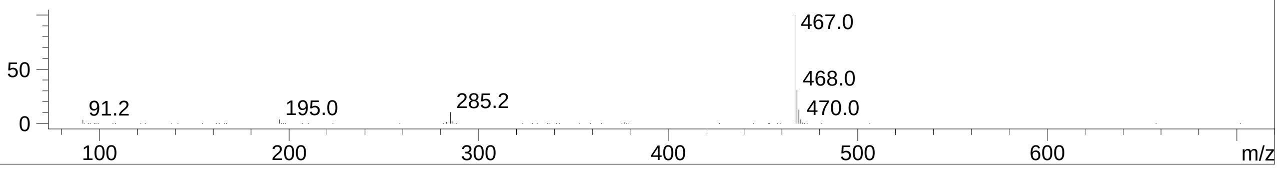

Inj.Date 7/6/2007 L P2-D-05 B03075 Acq. Method C:\HPCHEM\->
