# Supplementary material for: Selective androgen receptor degrader (SARD) to overcome antiandrogen resistance in castration-resistant prostate cancer
Source: eLife. 2023 Jan 19;12:e70700. doi: 10.7554/eLife.70700 (PMC9901937; doi:10.7554/eLife.70700)
Supplement: Source data 2. [file elife-70700-data2.zip › Supplementary Material_source_data/Figure 1-figure supplement 1 & Supplementary1a-source/Z15.PDF]

Sample Name: 1229024   OK

DFN: D:\DATA\MARCH\03_27\03_24_10\SAMPL014.D

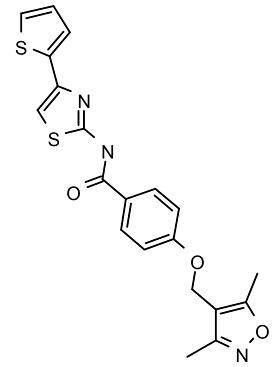

mw = 411,5

MaxPeak: 97.55%   Ret_Time: 0.705 min

The method for the Gradient Sample using short rapid resolution HT Cartridge ZORBAX SB-C18 4.6x15 mm (p/n 821975-932). For testing purity of syntez.

#	Time	Area%
1	0.522	2.45
2	0.705	97.55

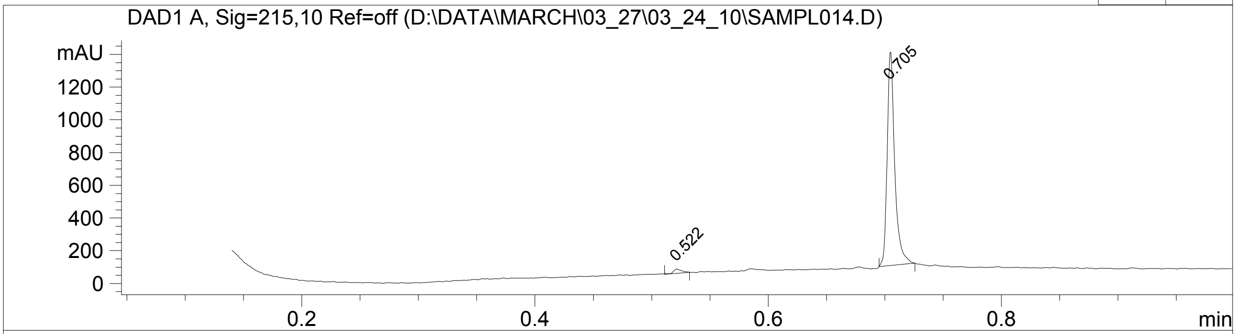

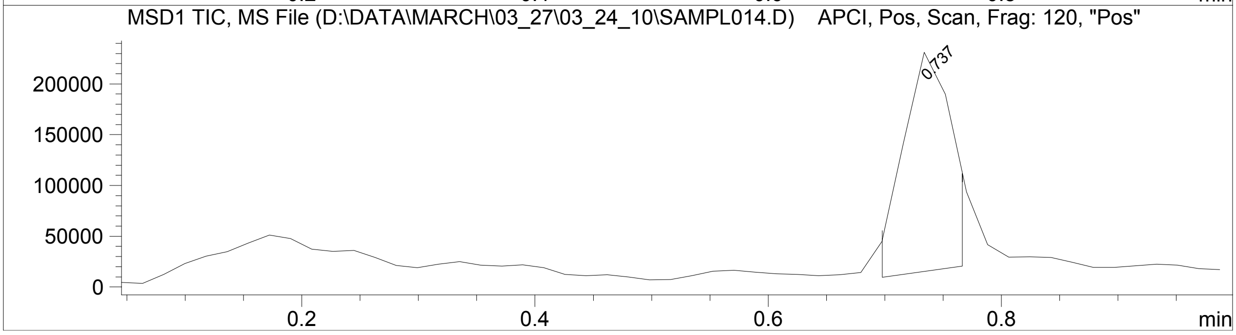

RT 0.737

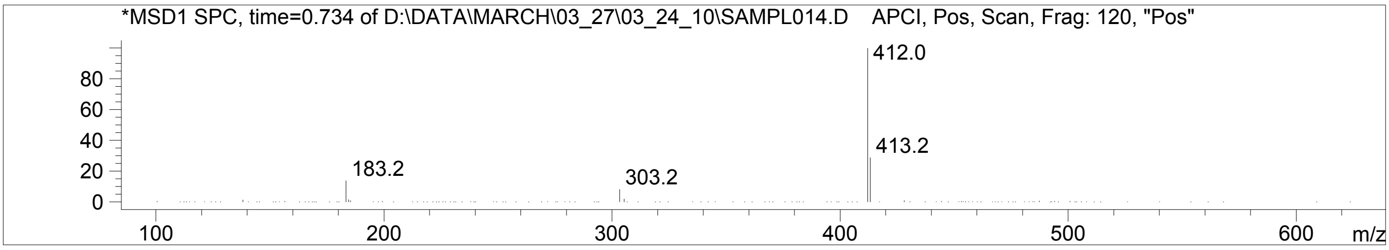

Inj.Date 25.03.2006   L   P2-B-06   Acq. Method SB15_3PN.M
