# Supplementary material for: Selective androgen receptor degrader (SARD) to overcome antiandrogen resistance in castration-resistant prostate cancer
Source: eLife. 2023 Jan 19;12:e70700. doi: 10.7554/eLife.70700 (PMC9901937; doi:10.7554/eLife.70700)
Supplement: Source data 2. [file elife-70700-data2.zip › Supplementary Material_source_data/Figure 1-figure supplement 1 & Supplementary1a-source/Z18.PDF]

Sample Name: 1133741 OK

DFN: D:\DATATY~1\OLD\04\V04_04\V0404_04\
SAMPL026.D

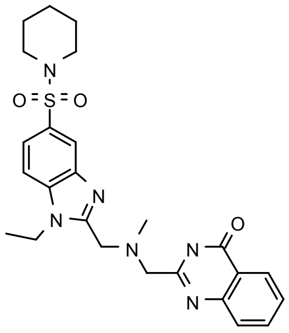

mw = 494,62

MaxPeak: 97.13% Ret_Time: 0.645 min

The method for the Gradient Sample using short rapid resolution HT Cartridge ZORBAX SB-C18 4.6x15 mm (p/n 821975-932). For testing purity of syntez.

#	Time	Area%
1	0.600	0.80
2	0.645	97.13
3	0.707	2.07

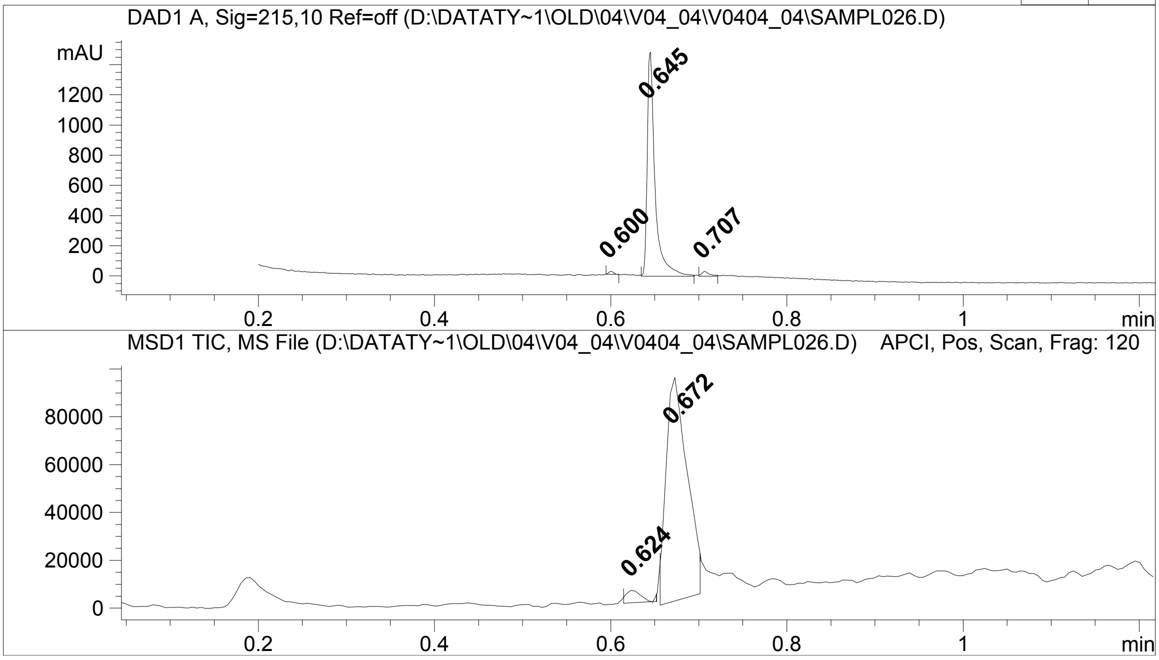

RT 0.624

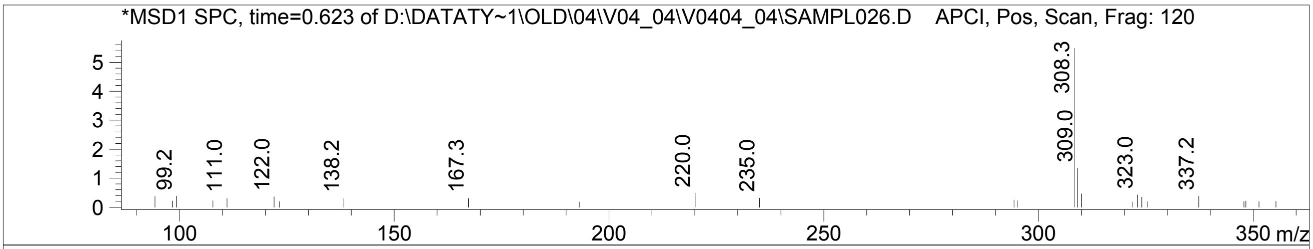

RT 0.672

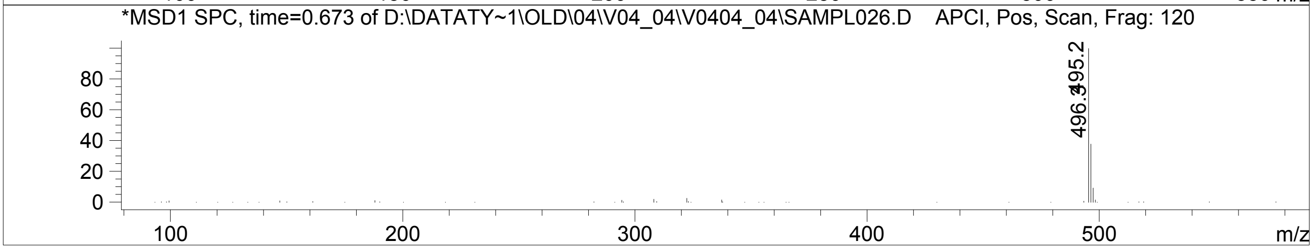

Inj.Date 05.04.2006 O P2-D-03 Acq. Method SB15TFA3.M
