# Supplementary material for: Selective androgen receptor degrader (SARD) to overcome antiandrogen resistance in castration-resistant prostate cancer
Source: eLife. 2023 Jan 19;12:e70700. doi: 10.7554/eLife.70700 (PMC9901937; doi:10.7554/eLife.70700)

Acquisition Time (sec)	2.9884	Comment	2009765-112207-D			Date	Jul 22 2016
Date Stamp	Jul 22 2016	File Name	D:\20160720-KG\112207-D_20160722_01\112207-D_PSLABEL_01.FID\FID				
Frequency (MHz)	499.91	Nucleus	1H	Number of Transients	16	Original Points Count	32768
Points Count	32768	Pulse Sequence	s2pul	Receiver Gain	46.00	Solvent	DMSO-d6
Spectrum Offset (Hz)	4504.6592	Spectrum Type	STANDARD	Sweep Width (Hz)	10964.91	Temperature (degree C)	25.000

^{1}H NMR (500 MHz, DMSO-d_6) δ ppm 10.18 (s, 1 H) 9.37 (br. s., 1 H) 9.28 (d, *J*=1.67 Hz, 1 H) 8.98 (s, 1 H) 8.69 (dd, *J*=4.69, 1.67 Hz, 1 H) 8.52 (d, *J*=5.02 Hz, 1 H) 8.48 (dt, *J*=8.03, 1.84 Hz, 1 H) 8.08 (d, *J*=2.01 Hz, 1 H) 7.95 (d, *J*=8.03 Hz, 2 H) 7.53 (dd, *J*=8.03, 4.68 Hz, 1 H) 7.47 - 7.51 (m, 2 H) 7.46 (br. s., 1 H) 7.43 (d, *J*=5.02 Hz, 1 H) 7.21 (d, *J*=8.37 Hz, 1 H) 3.65 (br. s., 2 H) 3.40 (br. s., 2 H) 3.04 (br. s., 2 H) 2.93 (d, *J*=10.71 Hz, 2 H) 2.79 (br. s., 3 H) 2.25 - 2.44 (m, 5 H) 2.23 (s, 3 H)

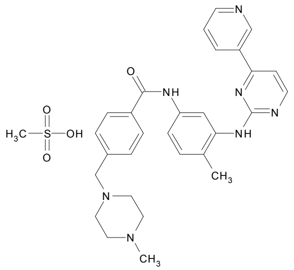

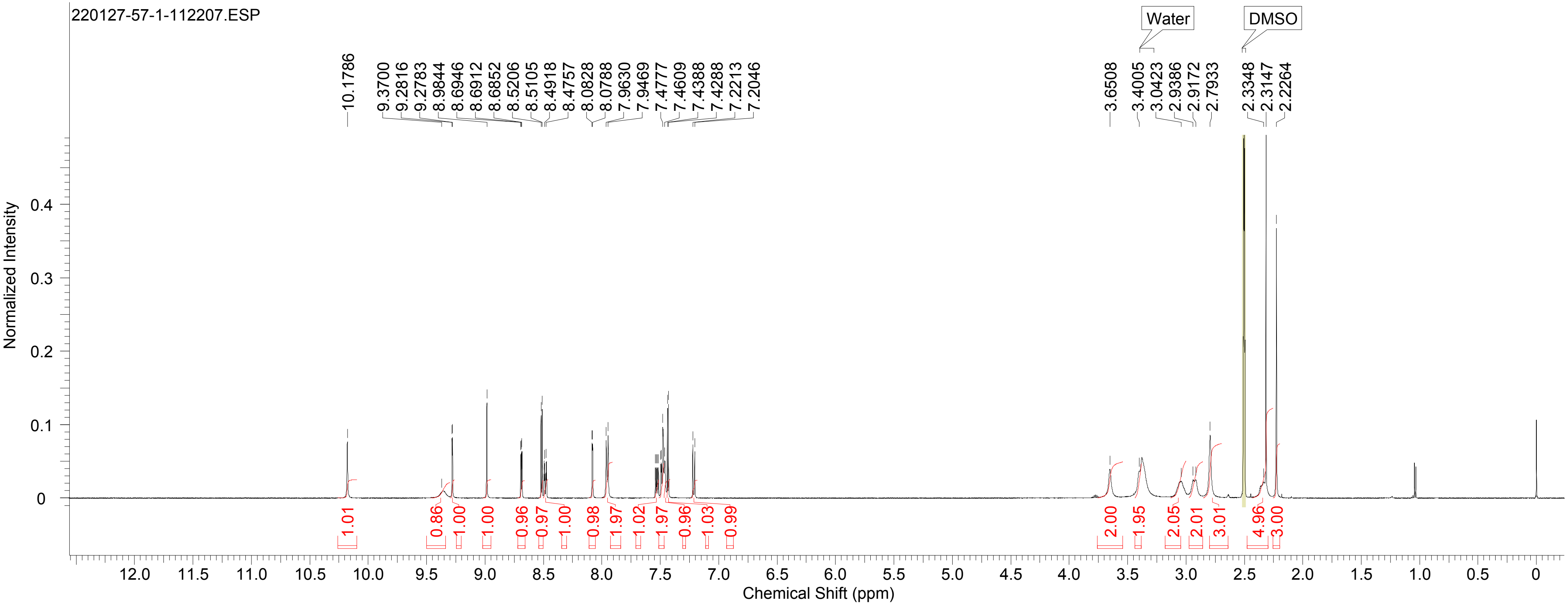

Supplement: Source data 2. [file elife-70700-data2.zip › Supplementary Material_source_data/Figure 1-figure supplement 1 & Supplementary1a-source/Z1-HNMR.pdf]
