# Supplementary material for: Selective androgen receptor degrader (SARD) to overcome antiandrogen resistance in castration-resistant prostate cancer
Source: eLife. 2023 Jan 19;12:e70700. doi: 10.7554/eLife.70700 (PMC9901937; doi:10.7554/eLife.70700)
Supplement: Source data 2. [file elife-70700-data2.zip › Supplementary Material_source_data/Figure 1-figure supplement 1 & Supplementary1a-source/Z1-HPLC.pdf]

SAMPLE INFORMATION

Sample Name:	220127-57-1 Imatinib Mesylate	Acquired By:	System
Sample Type:	Unknown		
Vial:	1	Acq. Method Set:	YZR
Injection #:	16		
Injection Volume:	20.00 ul	Processing Method	270
Run Time:	30.0 Minutes	Channel Name:	2487Channel 1
Sample Set Name:		Proc. Chnl. Descr.:	270

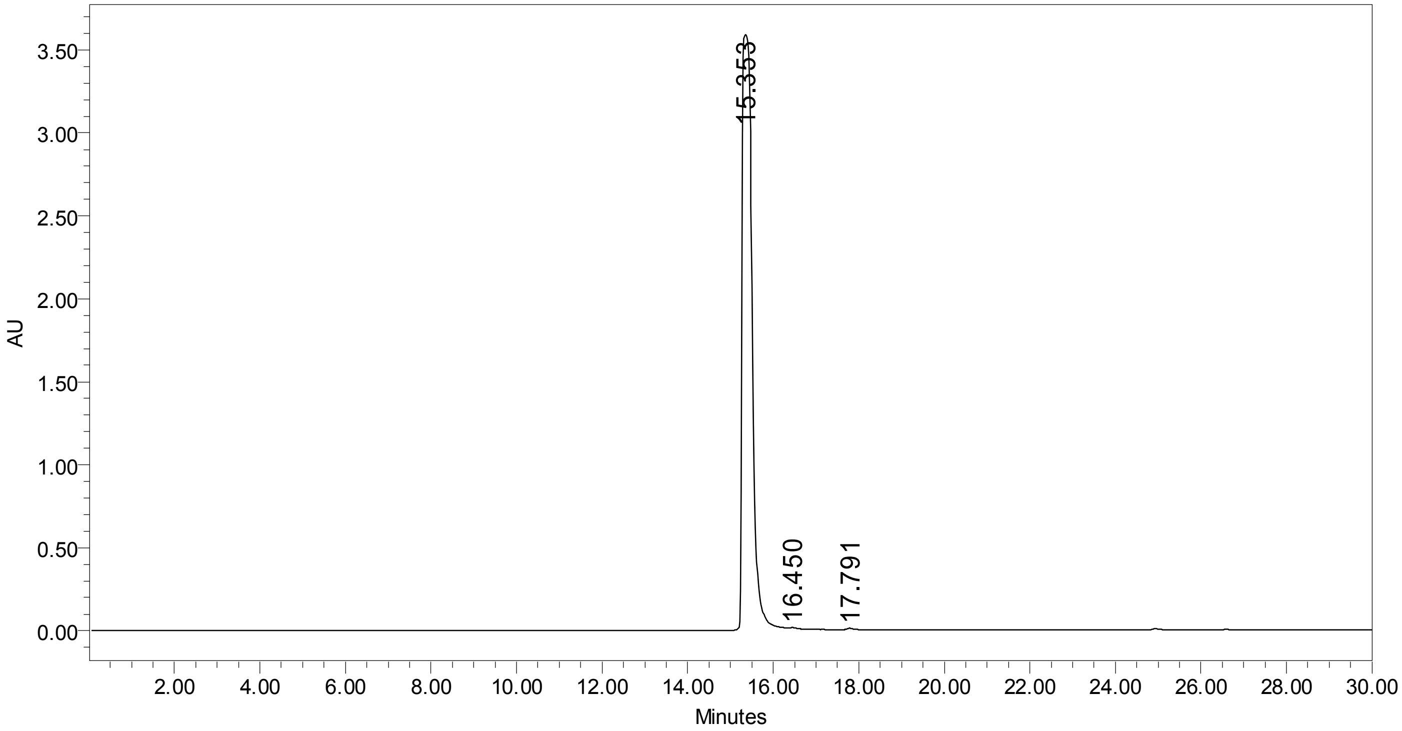

	RT	Area	% Area	Height
1	15.353	57856600	99.61	3589812
2	16.450	131789	0.23	11224
3	17.791	95690	0.16	8588
