# Supplementary material for: Selective androgen receptor degrader (SARD) to overcome antiandrogen resistance in castration-resistant prostate cancer
Source: eLife. 2023 Jan 19;12:e70700. doi: 10.7554/eLife.70700 (PMC9901937; doi:10.7554/eLife.70700)
Supplement: Source data 2. [file elife-70700-data2.zip › Supplementary Material_source_data/Figure 1-figure supplement 1 & Supplementary1a-source/Z21.PDF]

Sample Name: 659627   OK

DFN: D:\DATA~1\APRIL\04_27\04_26_06\SAMPL043.
D

MaxPeak: 96.75%   Ret_Time: 0.616 min

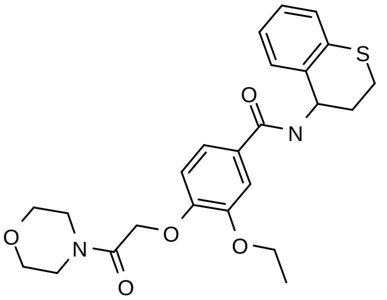

mw = 456,57

The method for the Gradient Sample using
short rapid resolution HT Cartridge ZORBAX
SB-C18 4.6x15 mm (p/n 821975-932). For
testing purity of syntez.

#	Time	Area%
1	0.616	96.75
2	0.691	3.25

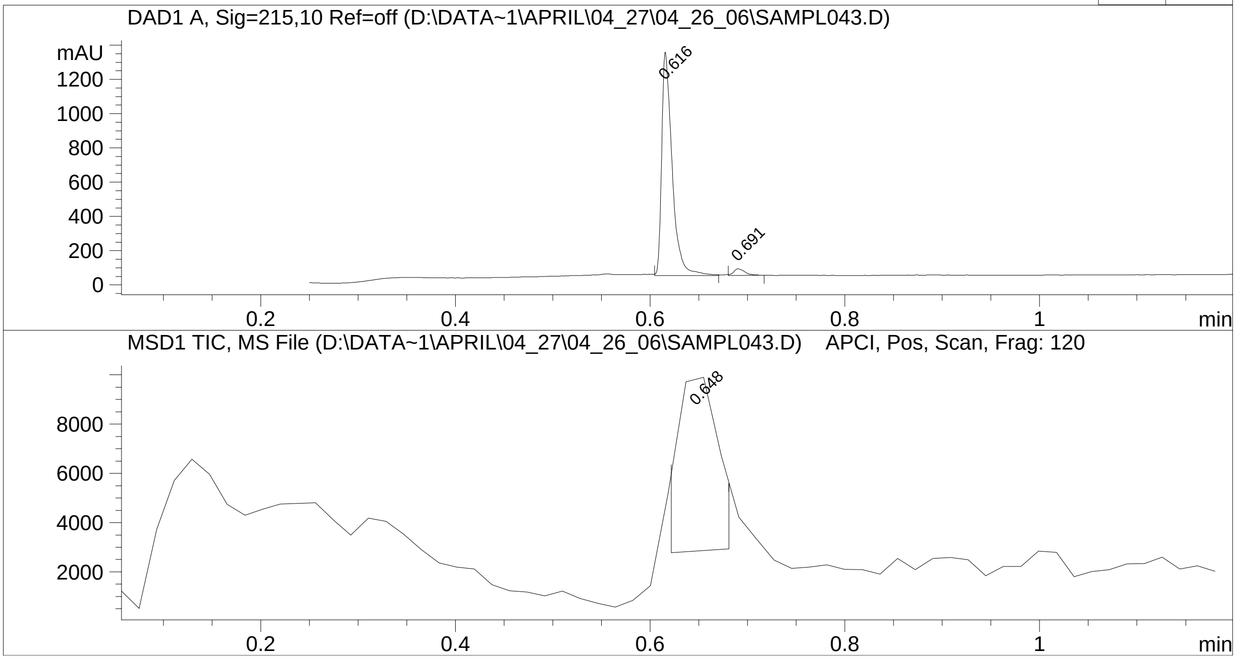

RT 0.648

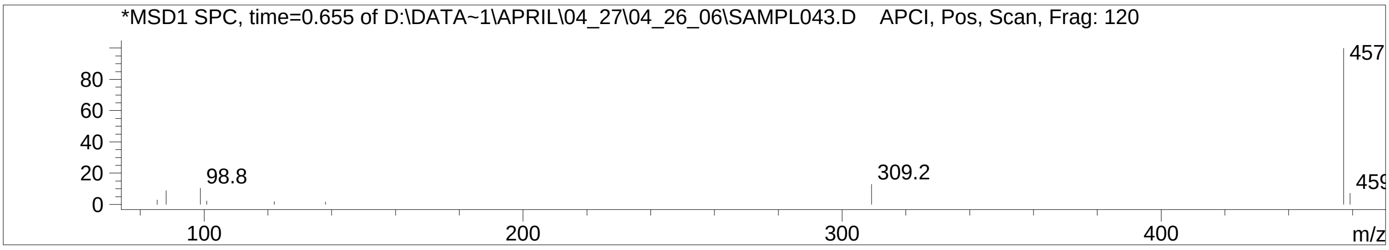

Inj.Date 26.04.2005   L   P2-F-01   Acq. Method SB15_3PN.M
