# Supplementary material for: Selective androgen receptor degrader (SARD) to overcome antiandrogen resistance in castration-resistant prostate cancer
Source: eLife. 2023 Jan 19;12:e70700. doi: 10.7554/eLife.70700 (PMC9901937; doi:10.7554/eLife.70700)
Supplement: Source data 2. [file elife-70700-data2.zip › Supplementary Material_source_data/Figure 1-figure supplement 1 & Supplementary1a-source/Z23.PDF]

MaxPeak:  90.07%
Ret_Time: 1.174 min

3123513$2

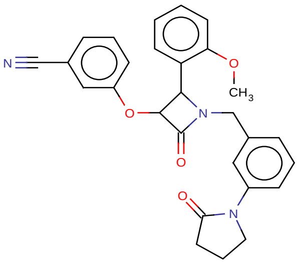


**Mol Wt** 467.516
**Exact Mass** 467.21

| # | Time | Area% |
|---|---|---|
| 1 | 1.174 | 90.07 |
| 2 | 1.200 | 9.93 |

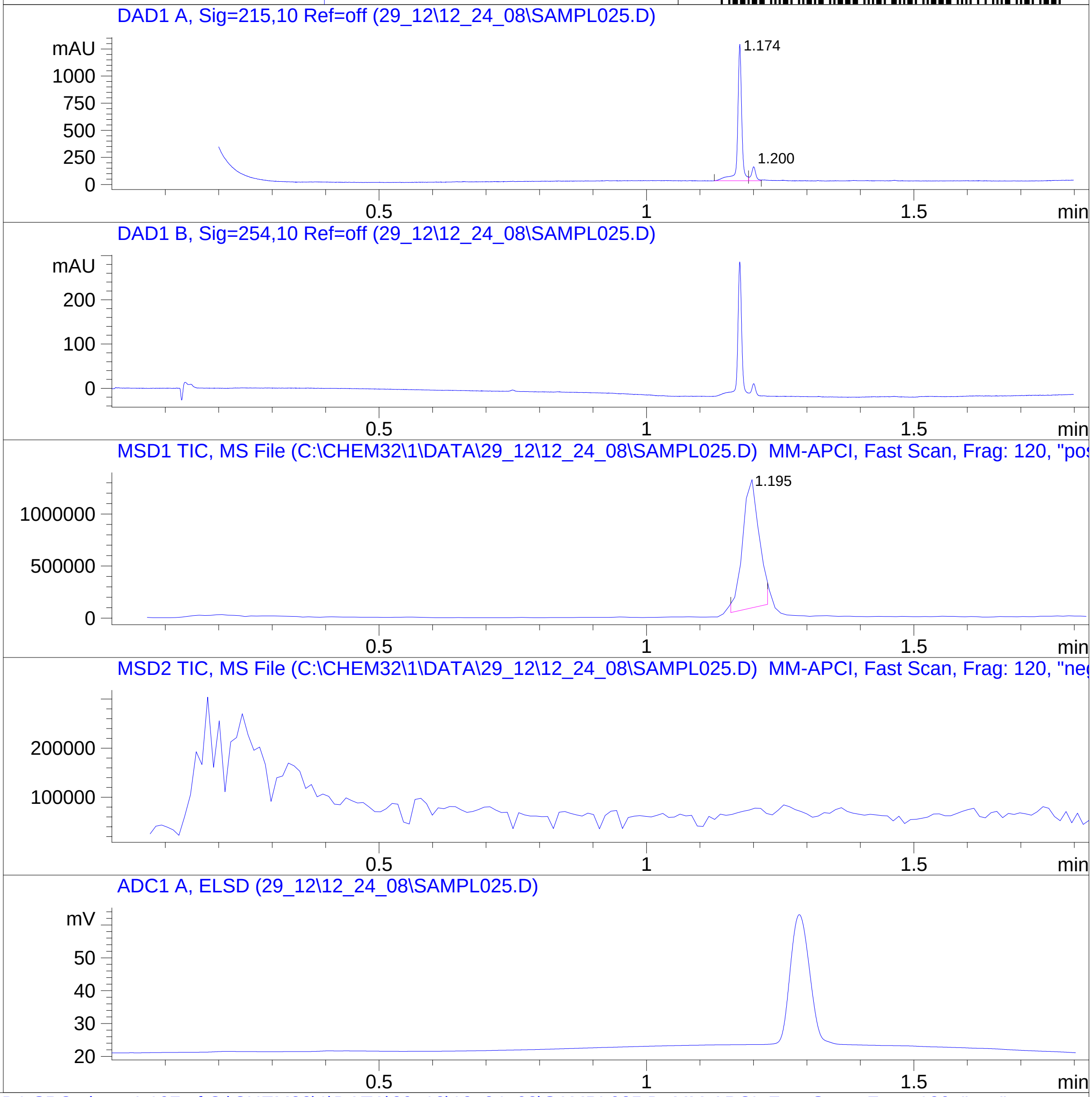


RT 1.195

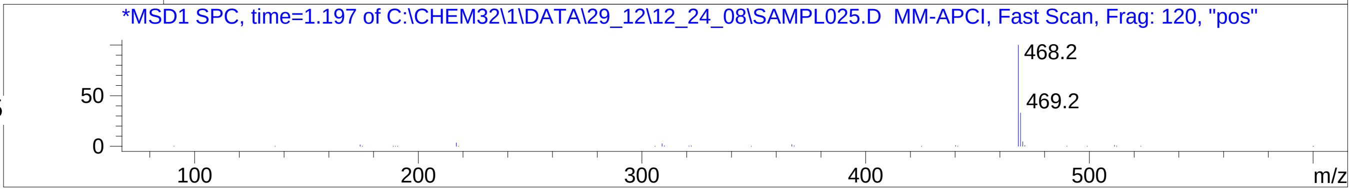


Inj.Date 12/26/2009 Link P2-C-07 RR Acq. Method C:\CHEM32\-> ->
