# Supplementary material for: Selective androgen receptor degrader (SARD) to overcome antiandrogen resistance in castration-resistant prostate cancer
Source: eLife. 2023 Jan 19;12:e70700. doi: 10.7554/eLife.70700 (PMC9901937; doi:10.7554/eLife.70700)
Supplement: Source data 2. [file elife-70700-data2.zip › Supplementary Material_source_data/Figure 1-figure supplement 1 & Supplementary1a-source/Z24.PDF]

Sample Name: 825821 OK

DFN: E:\DATATY~1\NEW\10\10_11\10_11_02\
SAMPL078.D

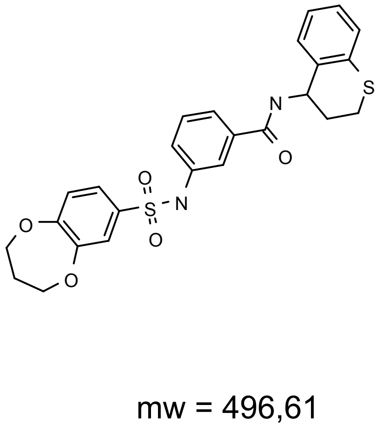

MaxPeak: 100.00% Ret_Time: 0.676 min

Could not execute user defined Macro ->

The method for the Gradient Sample using short rapid resolution HT Cartridge ZORBAX SB-C18 4.6x15 mm (p/n 821975-932). For testing purity of syntez.

#	Time	Area%
1	0.676	100.00

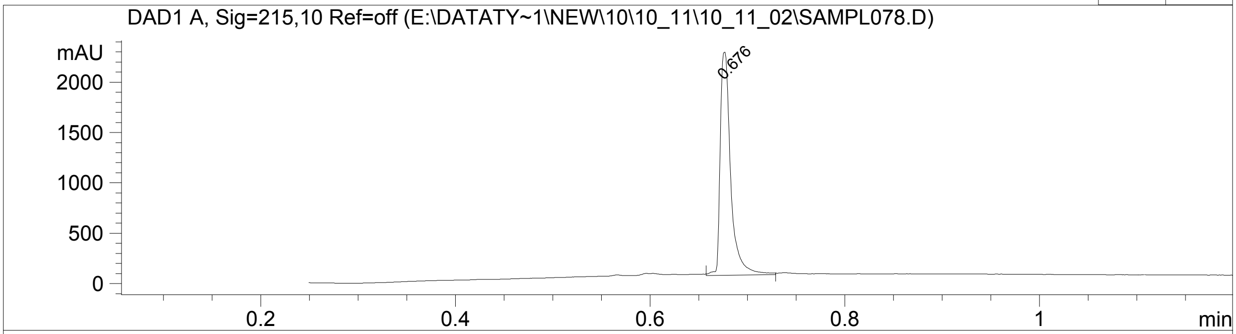

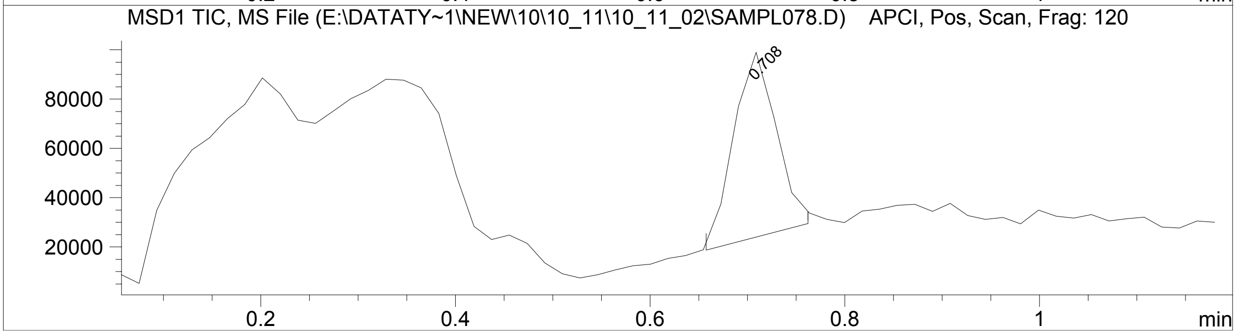

RT 0.708

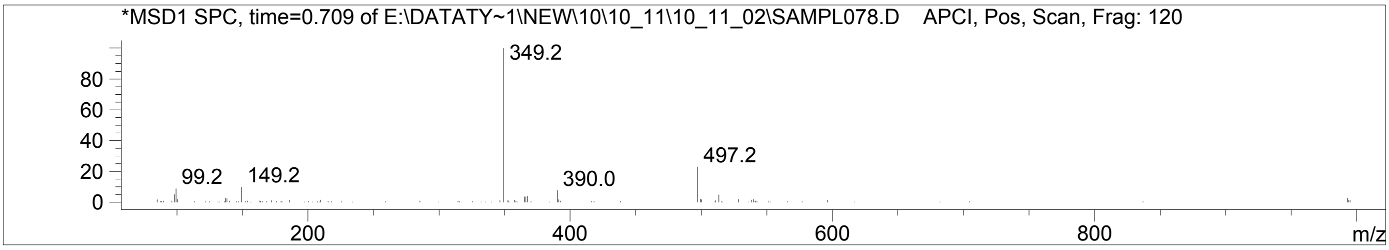

Inj.Date 11.10.2005 O P2-E-01 Acq. Method SB15_3PN.M
