# Supplementary material for: Selective androgen receptor degrader (SARD) to overcome antiandrogen resistance in castration-resistant prostate cancer
Source: eLife. 2023 Jan 19;12:e70700. doi: 10.7554/eLife.70700 (PMC9901937; doi:10.7554/eLife.70700)
Supplement: Source data 2. [file elife-70700-data2.zip › Supplementary Material_source_data/Figure 1-figure supplement 1 & Supplementary1a-source/Z25.PDF]

Sample Name: 550247 OK

DFN: C:\HPCHEM\1\DATA\OLD\V04_20\V0420_09\
SAMPL028.D

MaxPeak: 91.45% Ret_Time: 0.708 min

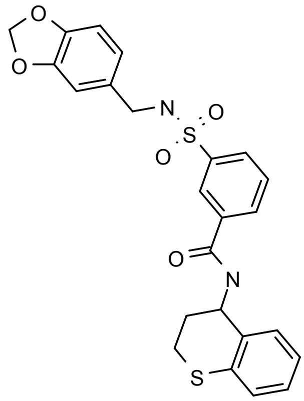

mw = 482,58

The method for the Gradient Sample using short rapid resolution HT Cartridge ZORBAX SB-C18 4.6x15 mm (p/n 821975-932). For testing purity of syntez.

#	Time	Area%
1	0.469	1.14
2	0.535	2.62
3	0.708	91.45
4	0.763	4.79

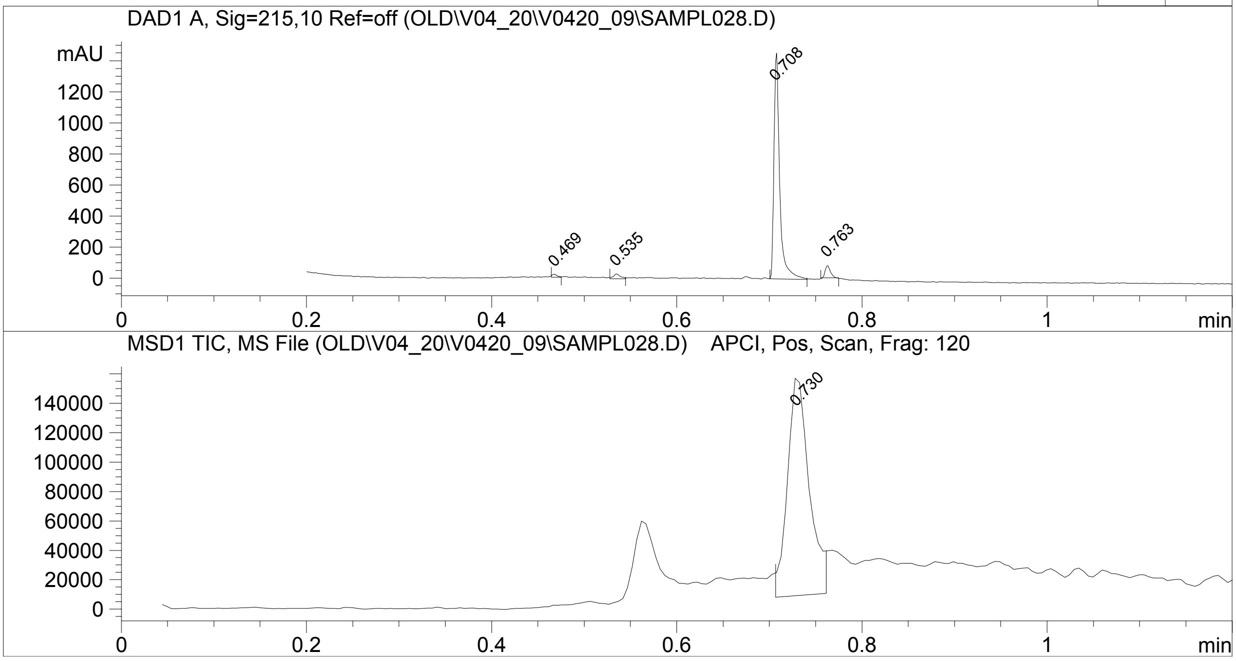

RT 0.730

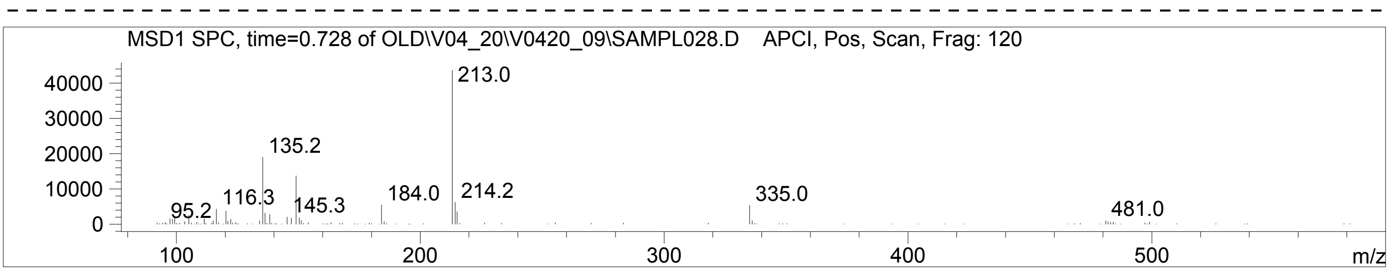

Inj.Date 21.04.2005 T P2-D-02 Acq. Method SB15TFA3.M
