# Supplementary material for: Selective androgen receptor degrader (SARD) to overcome antiandrogen resistance in castration-resistant prostate cancer
Source: eLife. 2023 Jan 19;12:e70700. doi: 10.7554/eLife.70700 (PMC9901937; doi:10.7554/eLife.70700)
Supplement: Source data 2. [file elife-70700-data2.zip › Supplementary Material_source_data/Figure 1-figure supplement 1 & Supplementary1a-source/Z28.PDF]

MaxPeak: 100.00%
Ret_Time: 0.717 min

1910060	OK

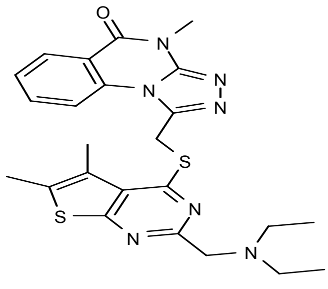

mw = 493.66

#	Time	Area%
1	0.717	100.00

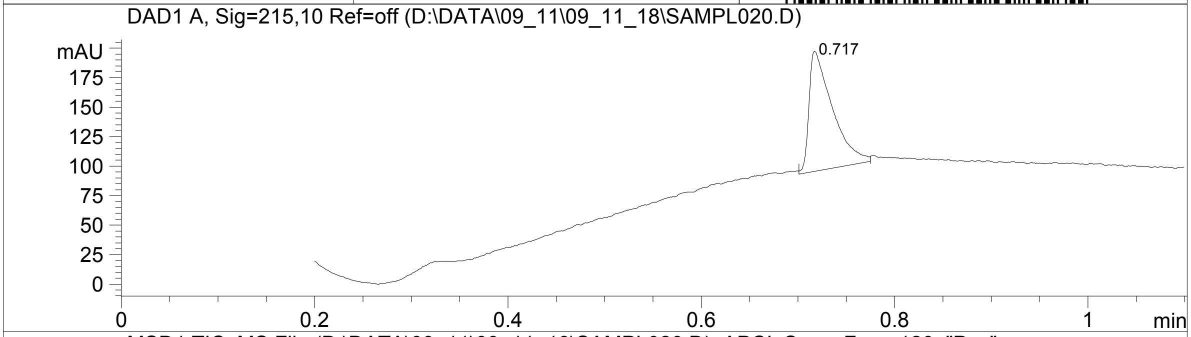

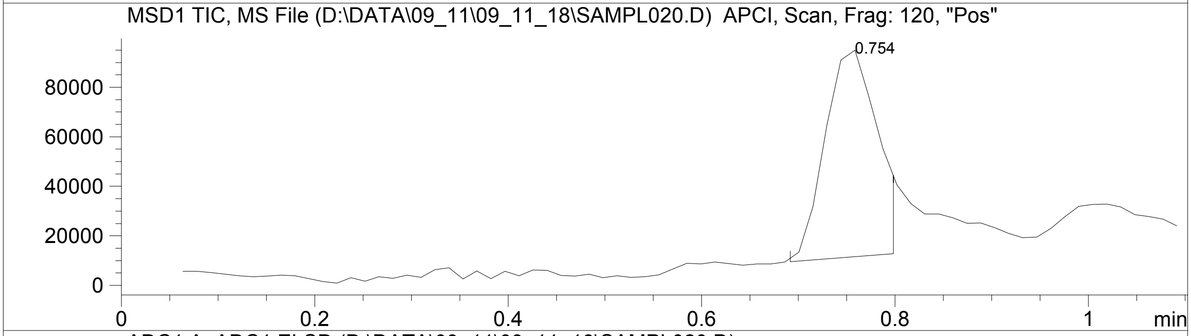

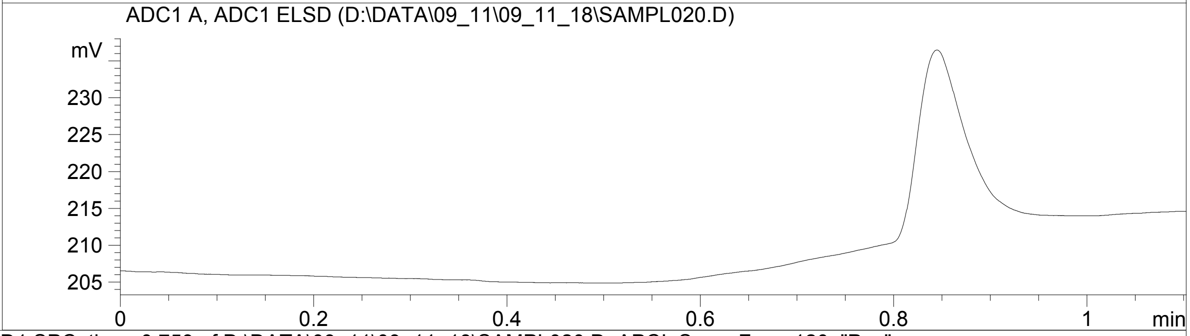

RT 0.754

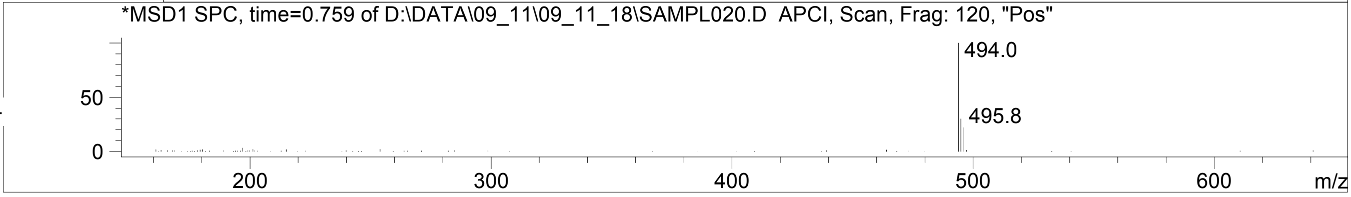

Inj.Date 9/12/2007 O P2-C-03 B05017 Acq. Method C:\HPCHEM\->
