# Supplementary material for: Selective androgen receptor degrader (SARD) to overcome antiandrogen resistance in castration-resistant prostate cancer
Source: eLife. 2023 Jan 19;12:e70700. doi: 10.7554/eLife.70700 (PMC9901937; doi:10.7554/eLife.70700)
Supplement: Source data 2. [file elife-70700-data2.zip › Supplementary Material_source_data/Figure 1-figure supplement 1 & Supplementary1a-source/Z31.PDF]

MaxPeak: 95.15%
Ret_Time: 0.960 min

3612172

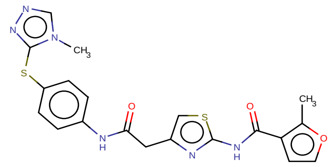

Mol Wt 454.525
Exact Mass 454.09

#	Time	Area%
1	0.825	3.13
2	0.960	95.15
3	1.023	1.73

DAD1 A, Sig=215,16 Ref=off (05_21\05_19_40\SAMPL053.D)

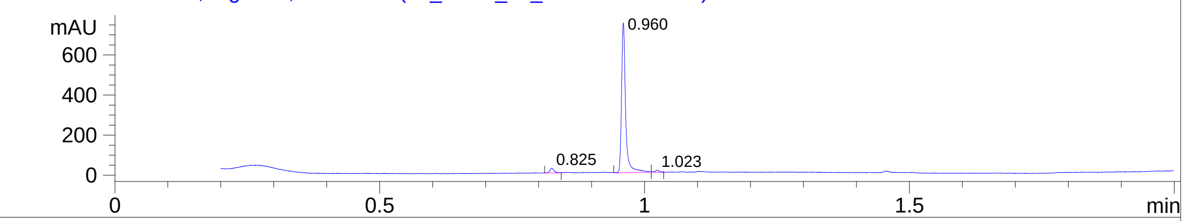

DAD1 B, Sig=254,16 Ref=off (05_21\05_19_40\SAMPL053.D)

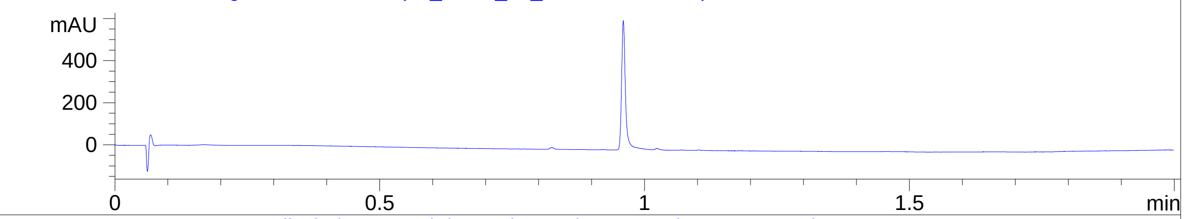

MSD1 TIC, MS File (C:\CHEM32\1\DATA\05_21\05_19_40\SAMPL053.D) ES-API, Scan, Frag: 100, "POS"

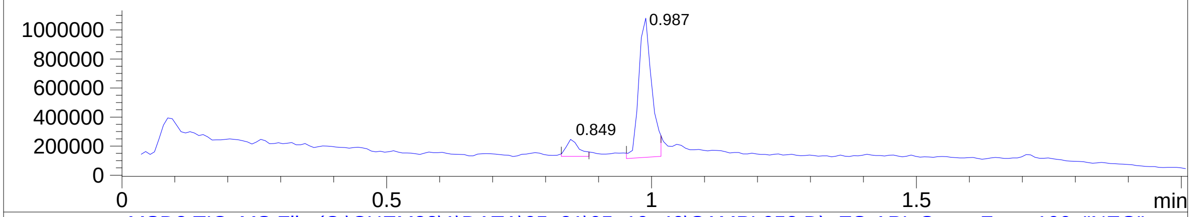

MSD2 TIC, MS File (C:\CHEM32\1\DATA\05_21\05_19_40\SAMPL053.D) ES-API, Scan, Frag: 100, "NEG"

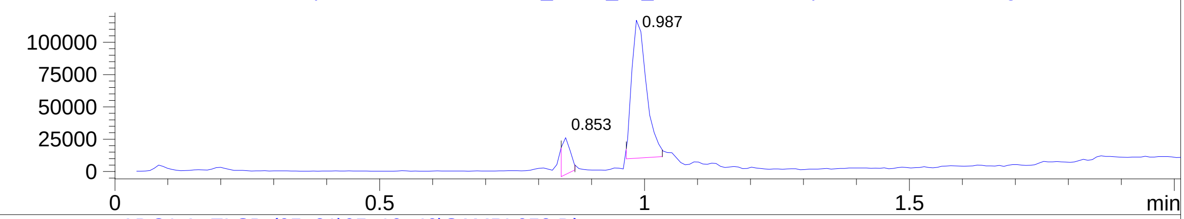

ADC1 A, ELSD (05_21\05_19_40\SAMPL053.D)

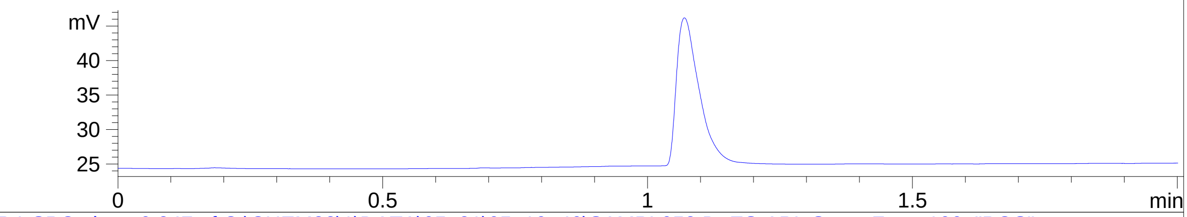

*MSD1 SPC, time=0.847 of C:\CHEM32\1\DATA\05_21\05_19_40\SAMPL053.D ES-API, Scan, Frag: 100, "POS"

RT 0.849

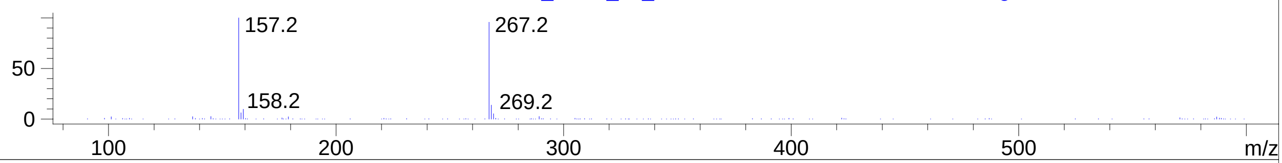

*MSD1 SPC, time=0.989 of C:\CHEM32\1\DATA\05_21\05_19_40\SAMPL053.D ES-API, Scan, Frag: 100, "POS"

RT 0.987

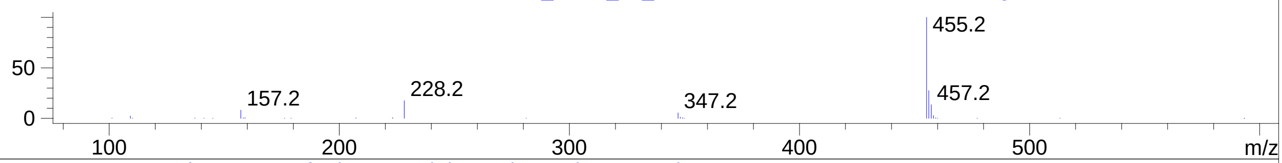

*MSD2 SPC, time=0.851 of C:\CHEM32\1\DATA\05_21\05_19_40\SAMPL053.D ES-API, Scan, Frag: 100, "NEG"

RT 0.853

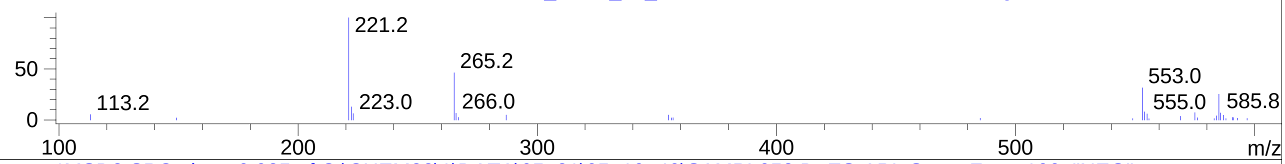

*MSD2 SPC, time=0.985 of C:\CHEM32\1\DATA\05_21\05_19_40\SAMPL053.D ES-API, Scan, Frag: 100, "NEG"

RT 0.987

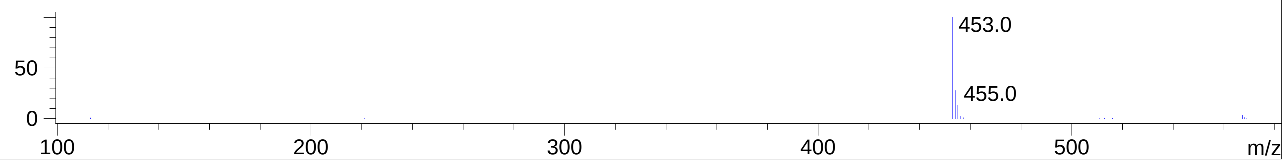

Inj.Date 5/20/2010   Link P2-F-09 '4'   Acq. Method C:\CHEM32\-> ->
