# Supplementary material for: Selective androgen receptor degrader (SARD) to overcome antiandrogen resistance in castration-resistant prostate cancer
Source: eLife. 2023 Jan 19;12:e70700. doi: 10.7554/eLife.70700 (PMC9901937; doi:10.7554/eLife.70700)
Supplement: Source data 2. [file elife-70700-data2.zip › Supplementary Material_source_data/Figure 1-figure supplement 1 & Supplementary1a-source/Z37.PDF]

/nmru vt02274 ivx07891

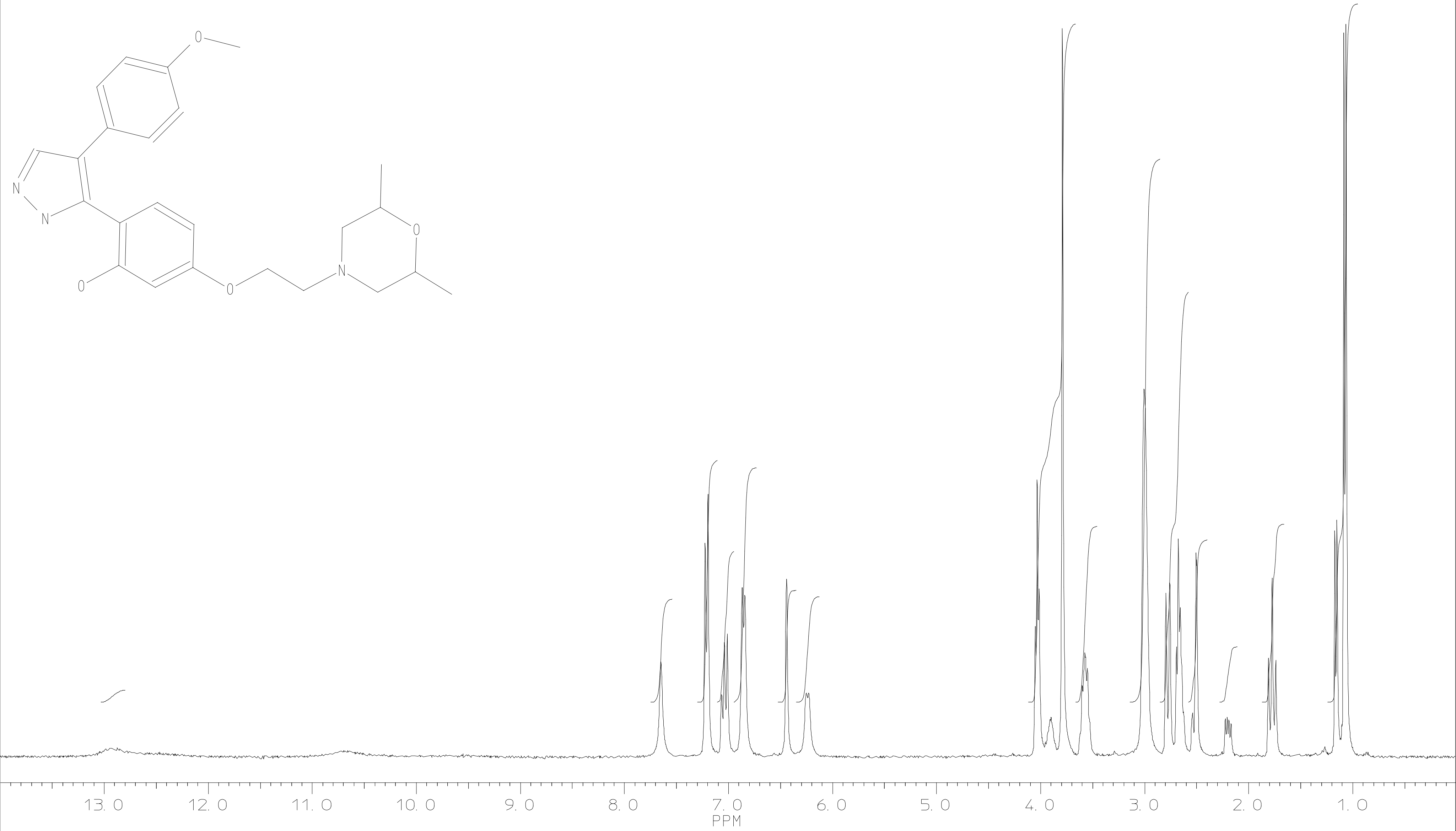

/nmru vt02274 ivx07891	AC-300 SF=300.13 MHz	SI=16K, SW=5376.34, PW=5.0	AQ=1.333, RD=3.00, NS=16	SR=4858.26, TE=318K	
	Moscow, 20 June 2014	Opr: STEPANOV A.V.;	Solv: DMSO+CCl4;	Prep: VT-63;	
