# Supplementary material for: Selective androgen receptor degrader (SARD) to overcome antiandrogen resistance in castration-resistant prostate cancer
Source: eLife. 2023 Jan 19;12:e70700. doi: 10.7554/eLife.70700 (PMC9901937; doi:10.7554/eLife.70700)
Supplement: Source data 2. [file elife-70700-data2.zip › Supplementary Material_source_data/Figure 1-figure supplement 1 & Supplementary1a-source/Z40.PDF]

Sample: 191 | Date: 09-Jan-2003 | Page 1.
File: 5850_91 | Time: 12:30:59 | AMRI code: ALB-H01862192
Vial: C/12 | Description: 695442 | Vial label: M1822242AMS0001

DAD: 254

max. intensity: 2.9E6

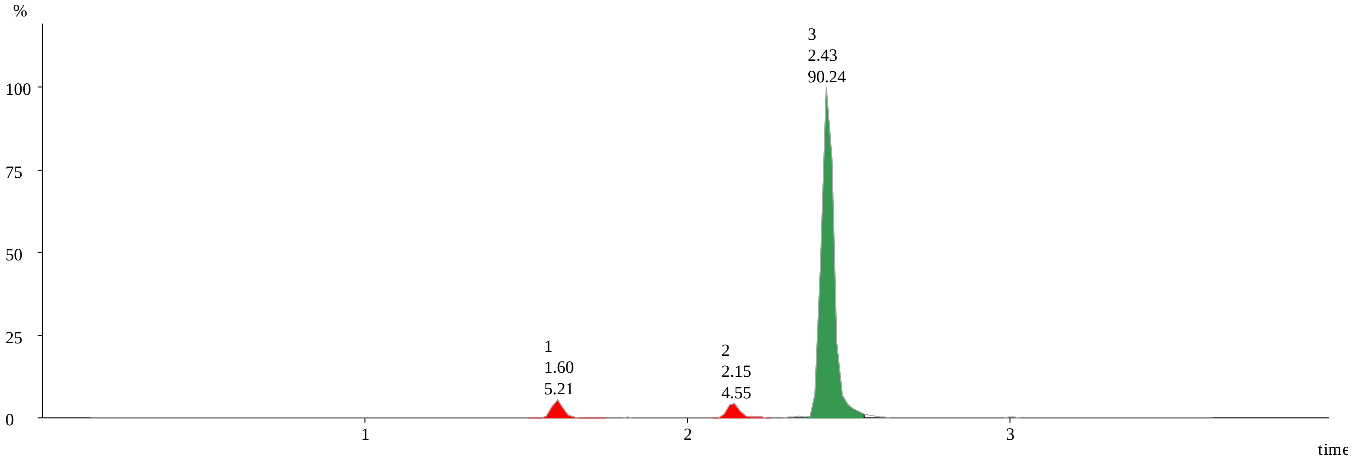

Peak_ID	Peak	Area	Area%	Height	Time	Mass Found
1	1.51 1.75	7.E3	5.21	2.E5	1.60	
2	2.08 2.26	6.E3	4.55	1.E5	2.15	
3	2.37 2.55	1.E5	90.24	3.E6	2.43	496.21

MS ES+ :497.21

max. intensity: 2.5E4

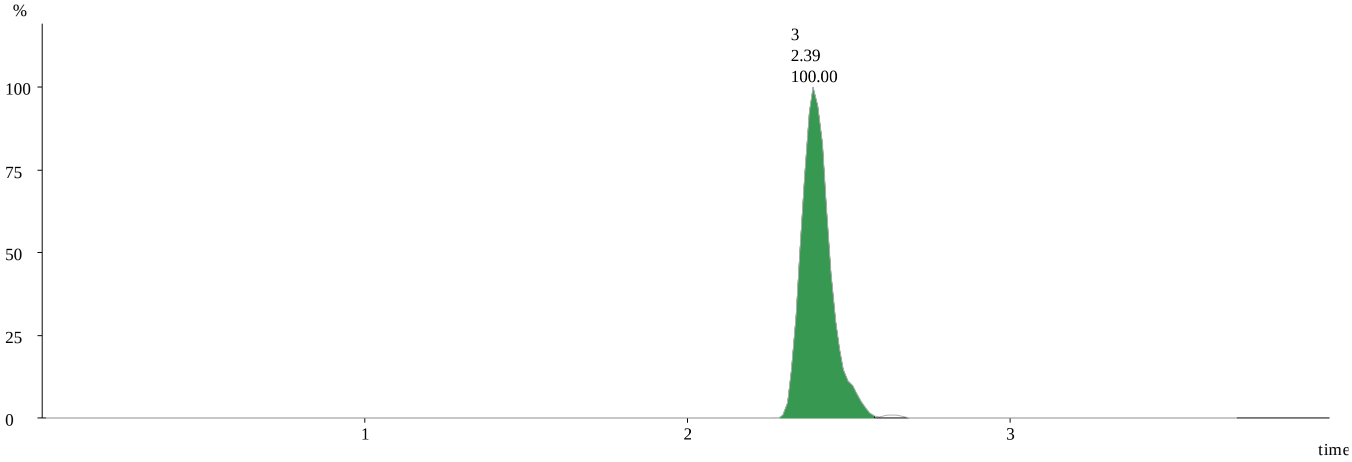

Peak_ID	Peak	Area	Area%	Height	Time	Mass Found
3	2.27 2.58	3.E3	100	2.E4	2.39	496.21

Sample: 191	Date: 09-Jan-2003	Page 2.
File: 5850_91	Time: 12:30:59	AMRI code: ALB-H01862192
Vial: C/12	Description: 695442	Vial label: M1822242AMS0001

MS ES+ :TIC

max. intensity: 3E4

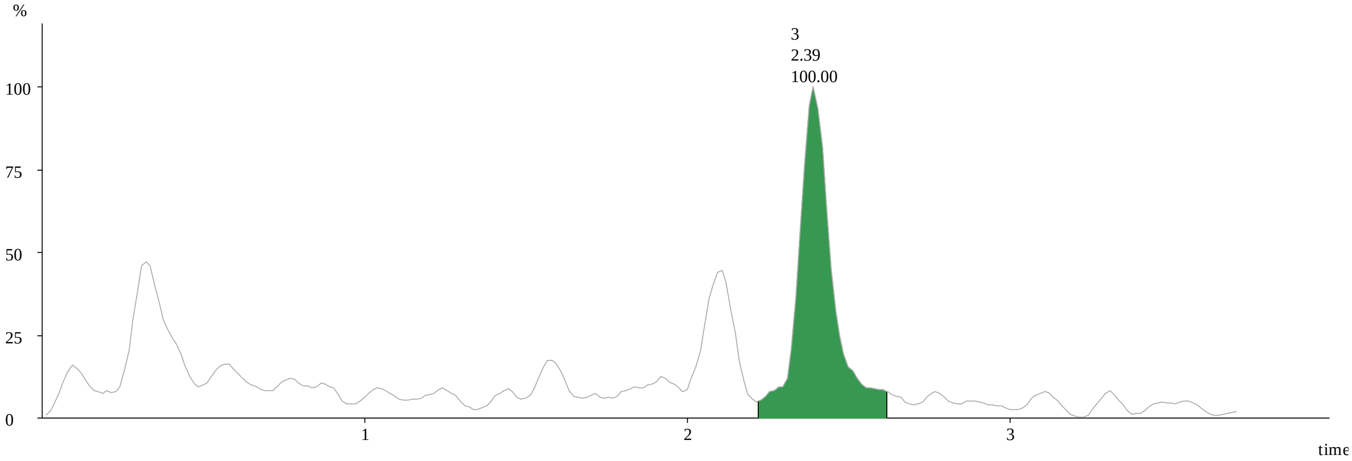

Peak_ID	Peak	Area	Area%	Height	Time	Mass Found
3	2.22 2.62	3.E3	100	3.E4	2.39	496.21

MS: ES+

Combine (117:119-(107:109+132:135))

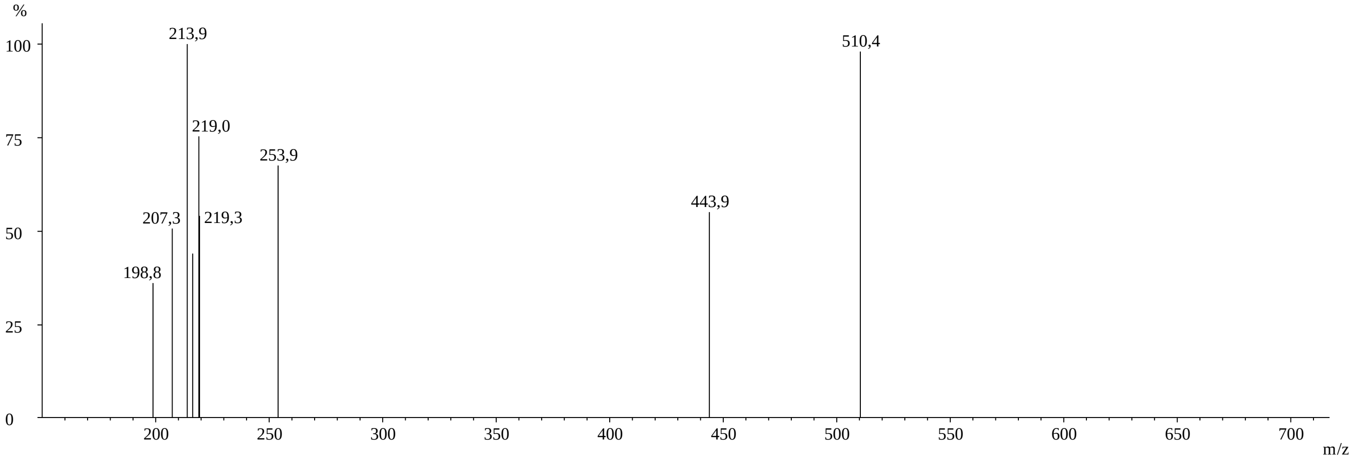

Peak_ID	Compound	Time	Mass found
1		1.60	

MS: ES+

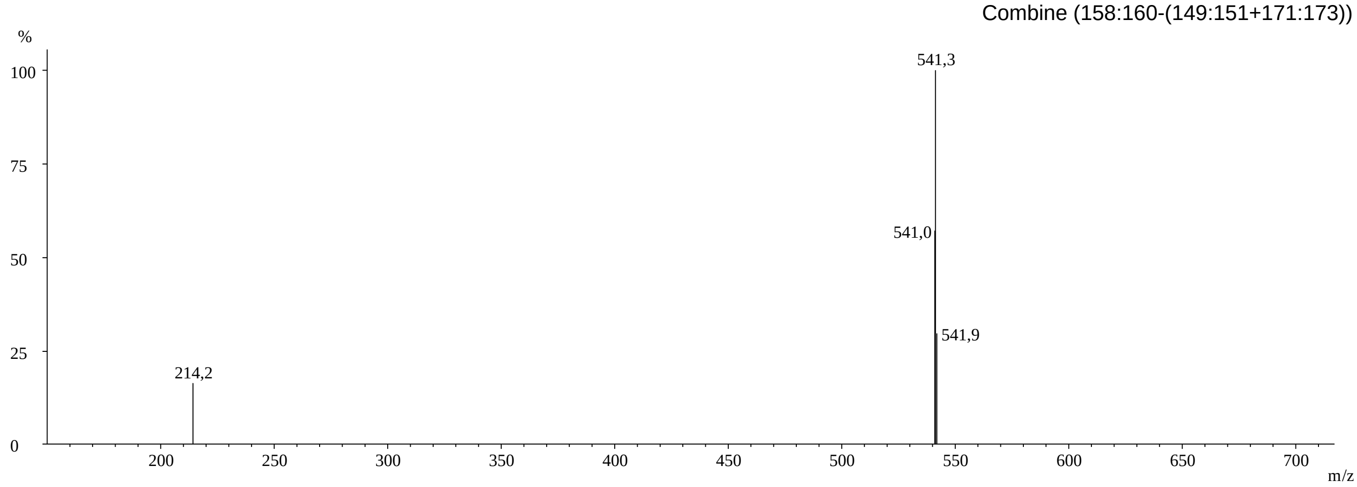

Peak_ID	Compound	Time	Mass found
2		2.15	

MS: ES+

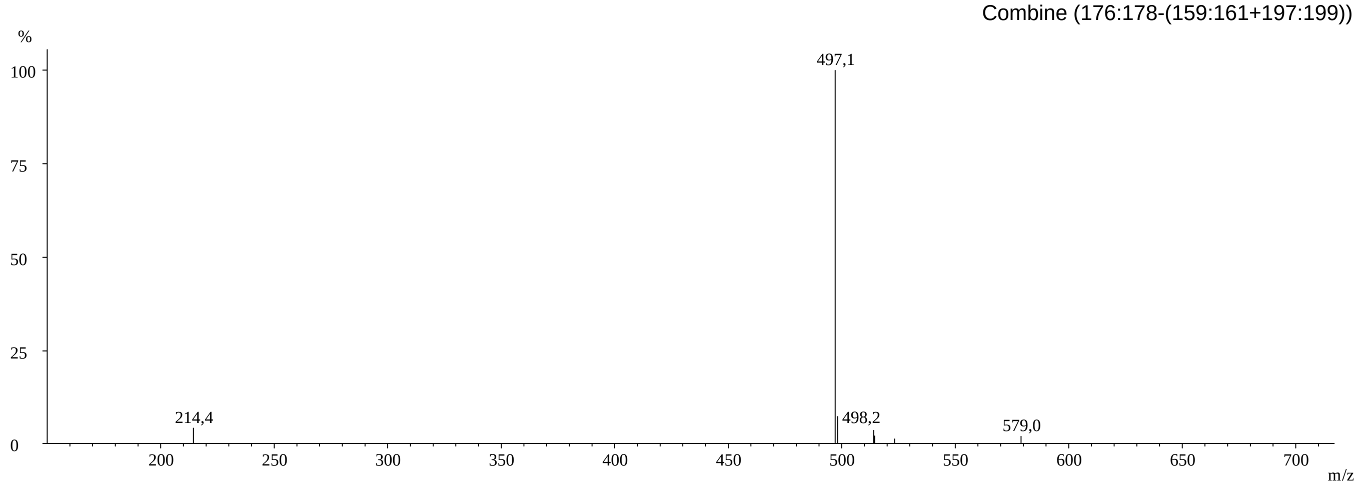

Peak_ID	Compound	Time	Mass found
3	Found	2.39	496.2100
