# Supplementary material for: Selective androgen receptor degrader (SARD) to overcome antiandrogen resistance in castration-resistant prostate cancer
Source: eLife. 2023 Jan 19;12:e70700. doi: 10.7554/eLife.70700 (PMC9901937; doi:10.7554/eLife.70700)
Supplement: Source data 2. [file elife-70700-data2.zip › Supplementary Material_source_data/Figure 1-figure supplement 1 & Supplementary1a-source/Z66.PDF]

Sample: 15 | Date: 27-Feb-2004 | Page 1.
File: 8323_05 | Time: 12:53:09 | AMRI code: ALB-H05180684
Vial: E/1 | Description: 909561 | Vial label: M5173041AMP0009

DAD: 220

max. intensity: 2.8E6

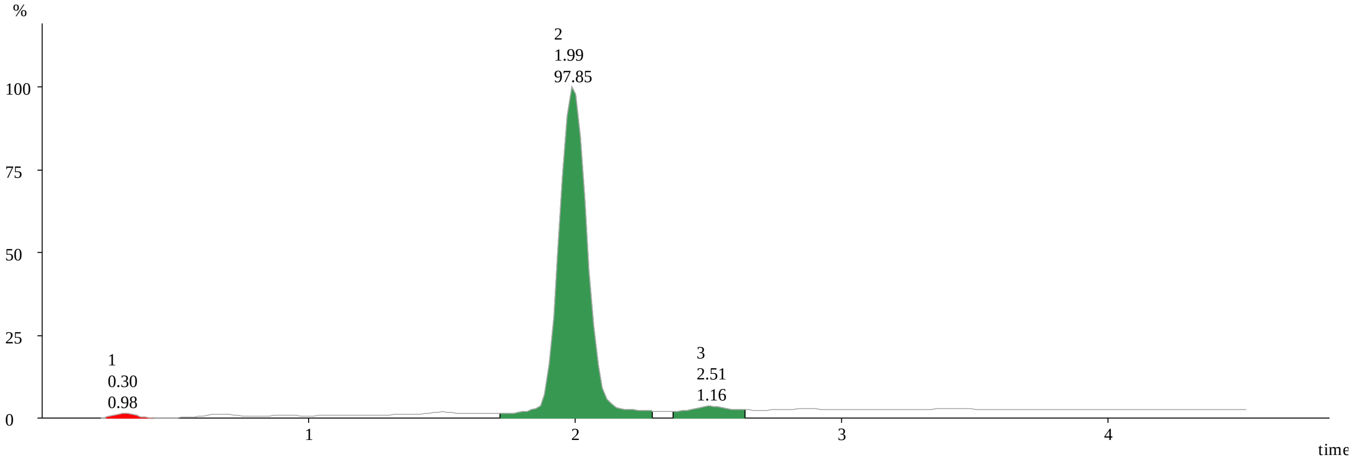

Peak_ID	Peak	Area	Area%	Height	Time	Mass Found
1	0.24 0.42	3.E3	0.98	4.E4	0.30	
2	1.72 2.29	3.E5	97.85	3.E6	1.99	455.26
3	2.37 2.64	4.E3	1.16	4.E4	2.51	455.26

MS ES+ :473.26+456.26

max. intensity: 9.3E4

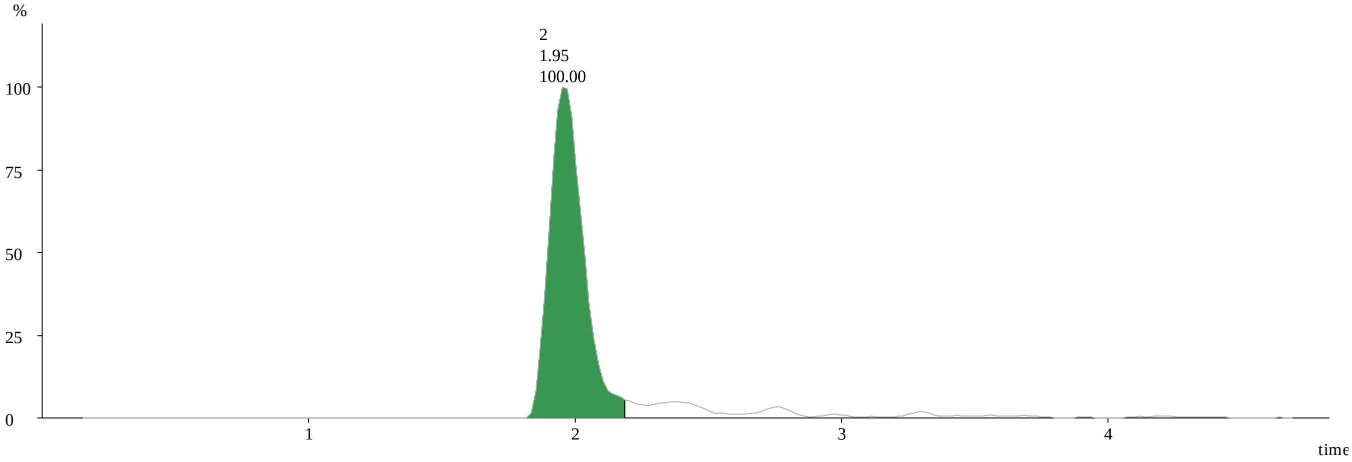

Peak_ID	Peak	Area	Area%	Height	Time	Mass Found
2	1.82 2.19	1.E4	100	9.E4	1.95	455.26

Sample: 15	Date: 27-Feb-2004	Page 2.
File: 8323_05	Time: 12:53:09	AMRI code: ALB-H05180684
Vial: E/1	Description: 909561	Vial label: M5173041AMP0009

MS ES+ :TIC

max. intensity: 1.2E5

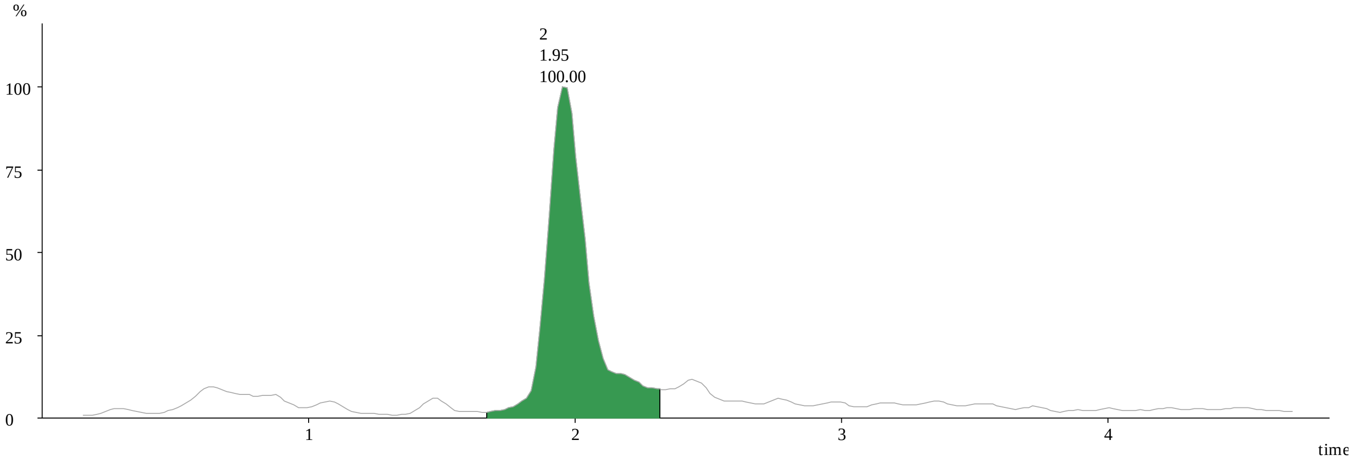

Peak_ID	Peak	Area	Area%	Height	Time	Mass Found
2	1.67 2.32	2.E4	100	1.E5	1.95	455.26

MS: ES+

Combine (116:118-(96:98+141:143))

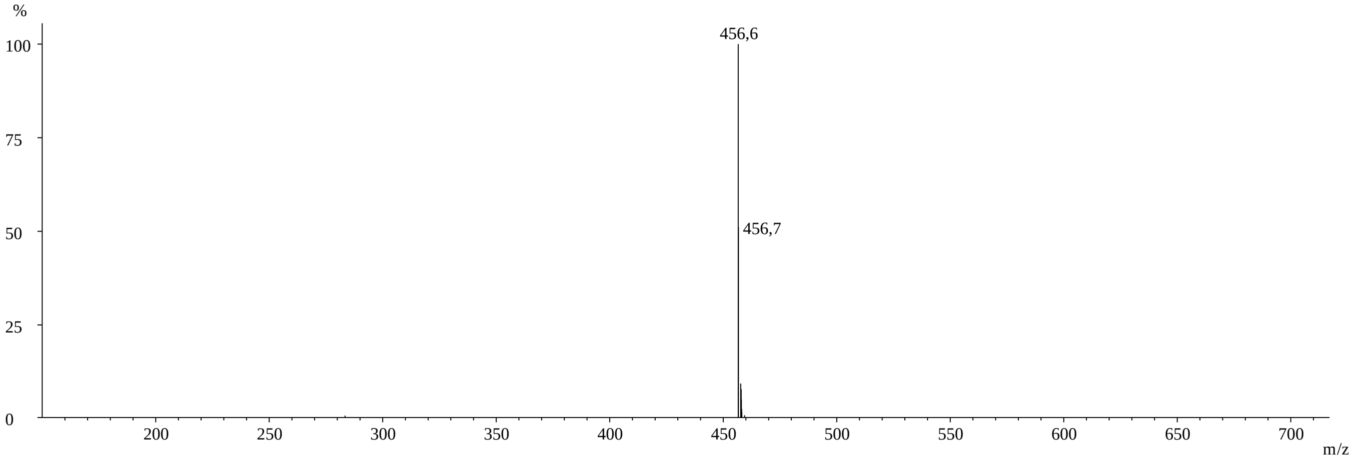

Peak_ID	Compound	Time	Mass found
2	Found	1.95	455.2600

MS: ES+

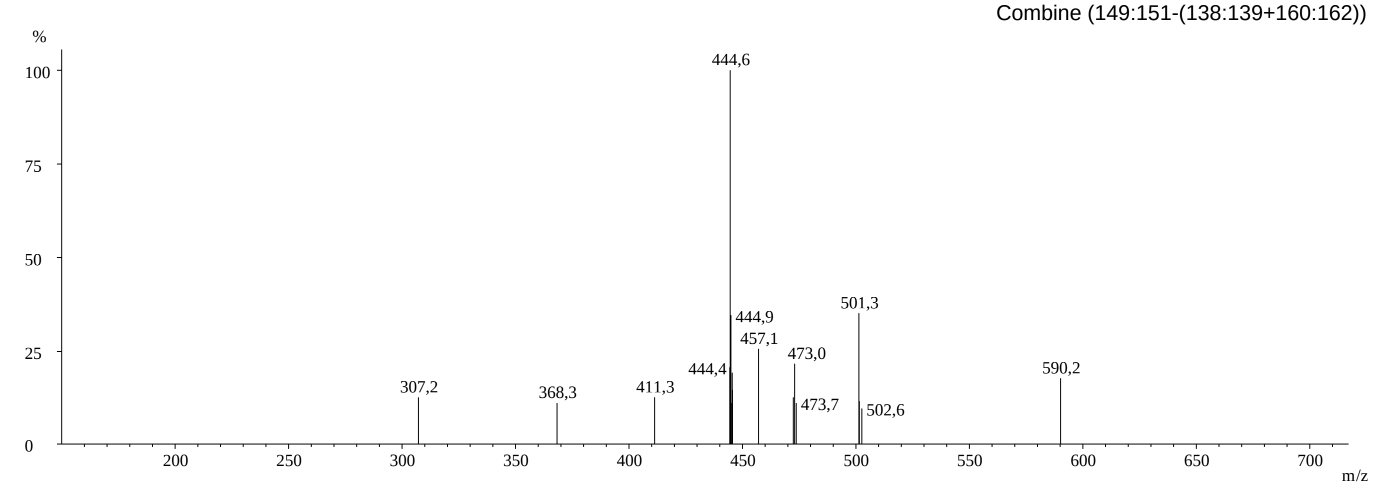

Peak_ID	Compound	Time	Mass found
3	Tentative	2.51	455.2600
