# Supplementary material for: Selective androgen receptor degrader (SARD) to overcome antiandrogen resistance in castration-resistant prostate cancer
Source: eLife. 2023 Jan 19;12:e70700. doi: 10.7554/eLife.70700 (PMC9901937; doi:10.7554/eLife.70700)
Supplement: Source data 2. [file elife-70700-data2.zip › Supplementary Material_source_data/Figure 1-figure supplement 1 & Supplementary1a-source/Z7.PDF]

MaxPeak: 96.59%
Ret_Time: 1.269 min

# 5466878

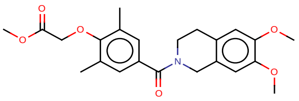

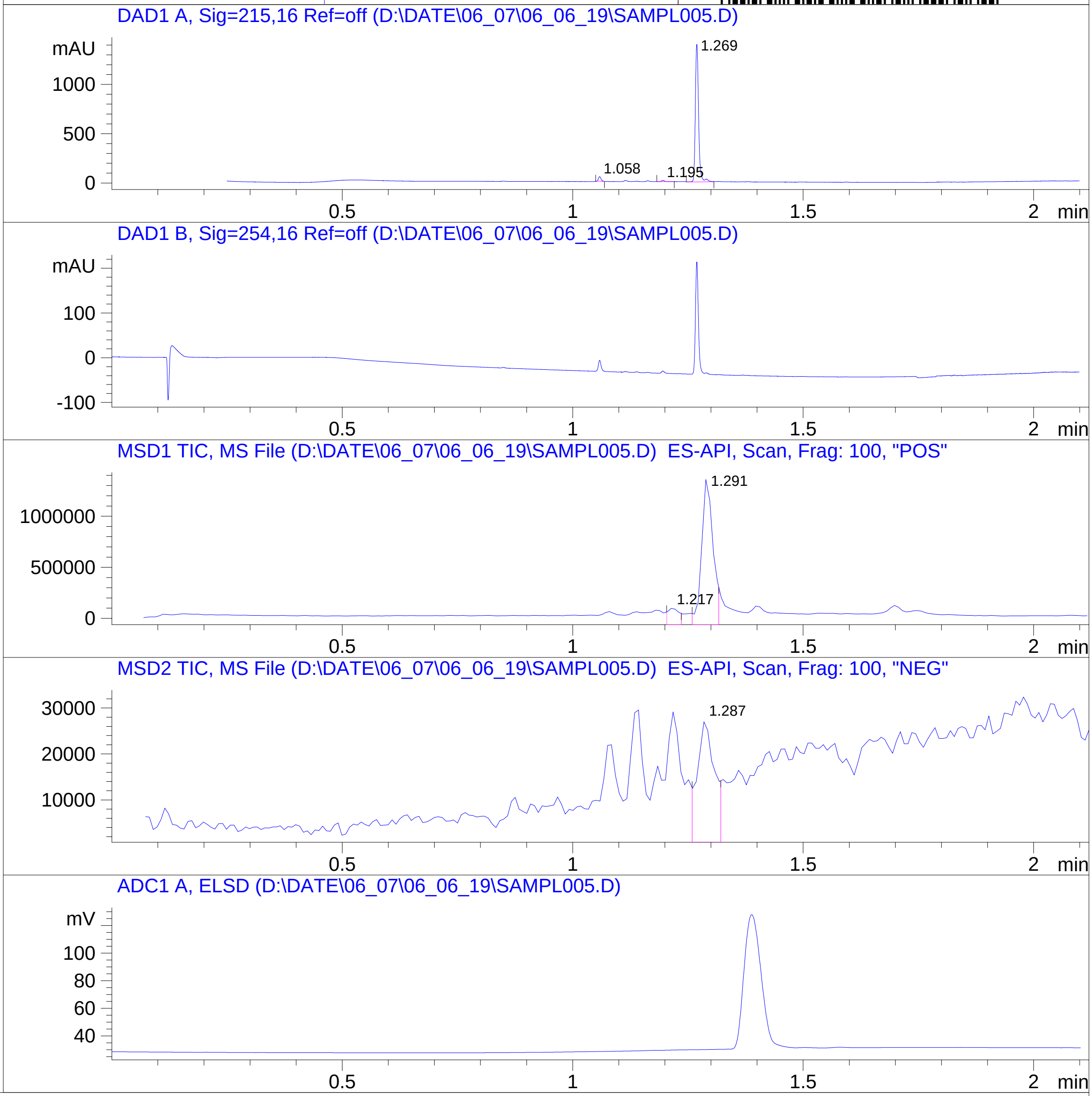


| Mol Wt | 413.46 |
|---|---|
| Exact Mass | 413.21 |

| # | Time | Area% |
|---|---|---|
| 1 | 1.058 | 2.53 |
| 2 | 1.195 | 0.88 |
| 3 | 1.269 | 96.59 |

RT 1.217

RT 1.291

RT 1.287

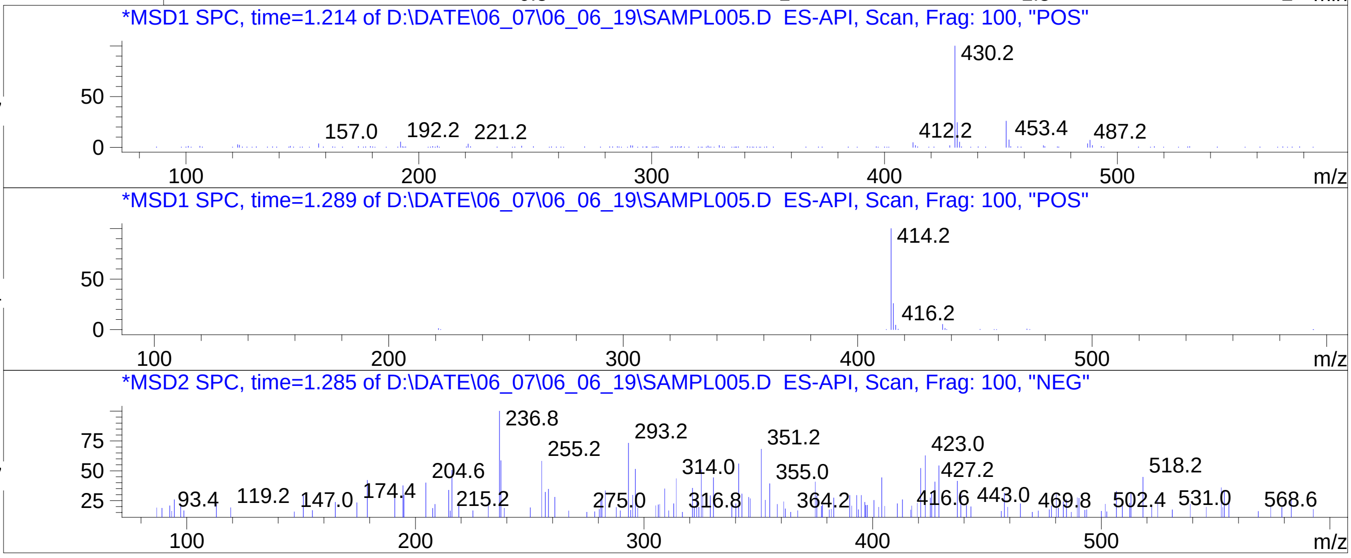


Inj.Date 6/7/2012 L P2-A-05 '4' Acq. Method C:\CHEM32\-> ->
