# Supplementary material for: Selective androgen receptor degrader (SARD) to overcome antiandrogen resistance in castration-resistant prostate cancer
Source: eLife. 2023 Jan 19;12:e70700. doi: 10.7554/eLife.70700 (PMC9901937; doi:10.7554/eLife.70700)
Supplement: Source data 2. [file elife-70700-data2.zip › Supplementary Material_source_data/Figure 1-figure supplement 1 & Supplementary1a-source/Z70.PDF]

Sample: 157 | Date: 10-Apr-2008 | Page 1.
File: Ar23321_57 | Time: 22:11:45 | AMRI code: ALB-H00824478
Vial: A/8 | Description: 793942 | Vial label: 300000451785

DAD: 220

max. intensity: 1.1E6

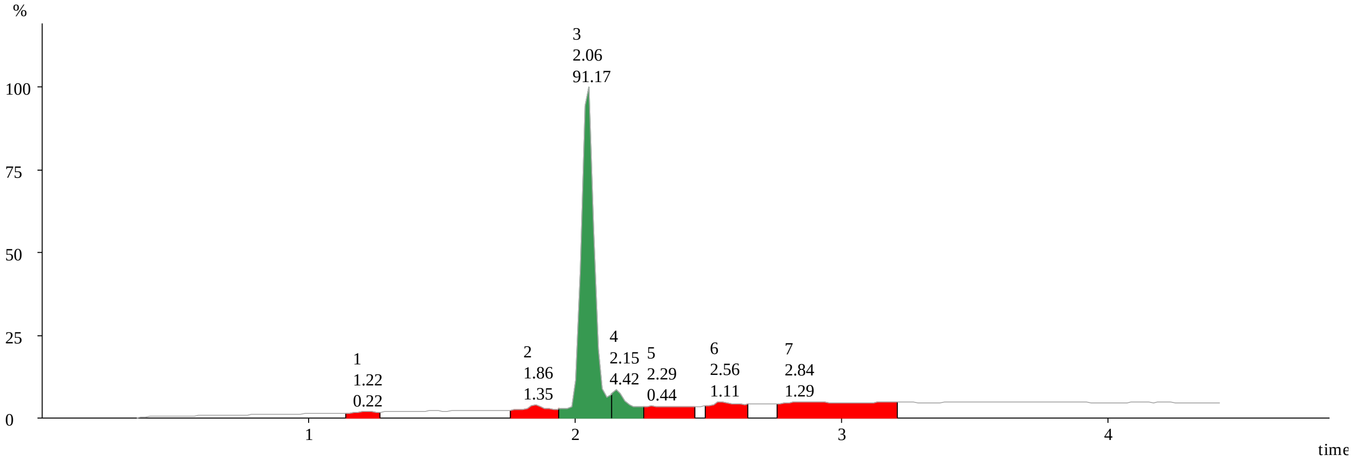

Peak_ID	Peak	Area	Area%	Height	Time	Mass Found
1	1.14 1.27	1.E2	0.22	2.E3	1.22	
2	1.76 1.94	9.E2	1.35	2.E4	1.86	
3	1.94 2.14	6.E4	91.17	1.E6	2.06	539.21
4	2.14 2.26	3.E3	4.42	6.E4	2.15	539.21
5	2.26 2.45	3.E2	0.44	4.E3	2.29	
6	2.49 2.65	7.E2	1.11	1.E4	2.56	
7	2.76 3.21	8.E2	1.29	5.E3	2.84	

MS ES+ :542.21+557.21+1079.42+540.21

max. intensity: 1.6E4

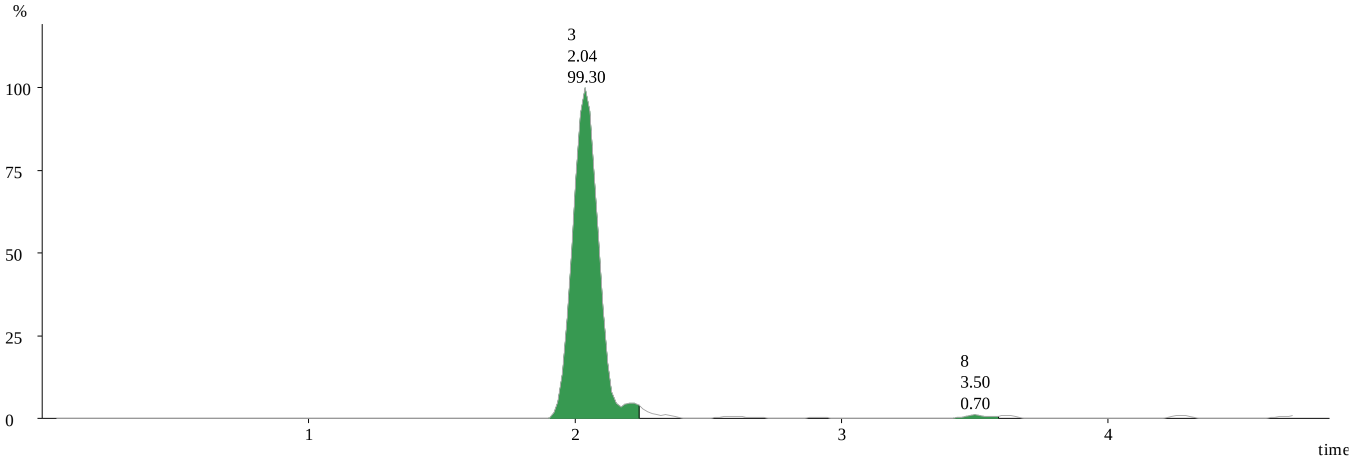

Peak_ID	Peak	Area	Area%	Height	Time	Mass Found
3	1.90 2.24	2.E3	99.3	2.E4	2.04	539.21
8	3.42 3.59	1.E1	0.7	2.E2	3.50	539.21

Sample: 157	Date: 10-Apr-2008	Page 2.
File: Ar23321_57	Time: 22:11:45	AMRI code: ALB-H00824478
Vial: A/8	Description: 793942	Vial label: 300000451785

MS ES+ :TIC

max. intensity: 2.1E4

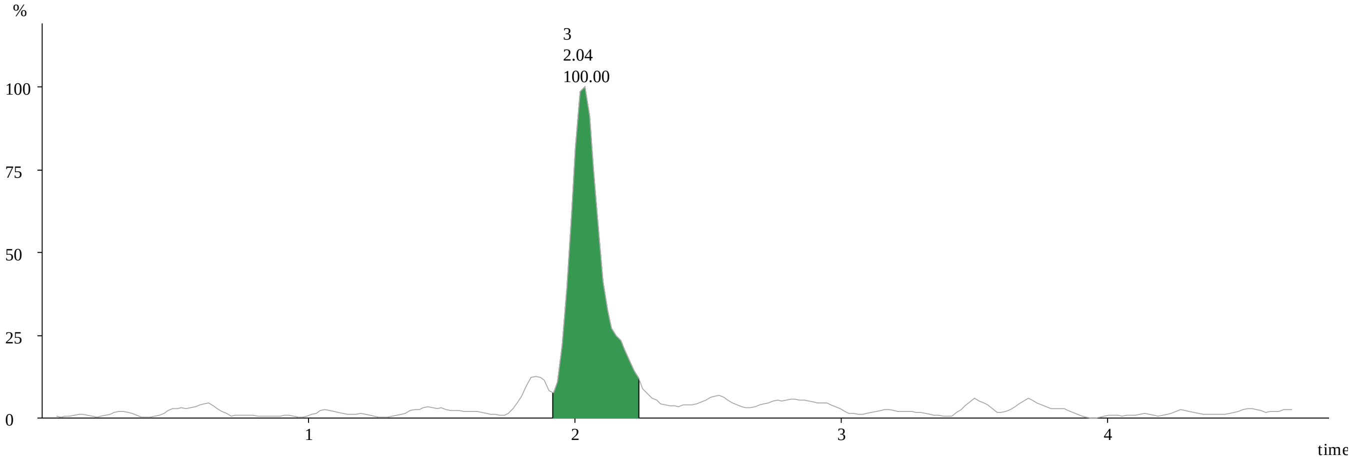

Peak_ID	Peak	Area	Area%	Height	Time	Mass Found
3	1.92 2.24	2.E3	100	2.E4	2.04	539.21

MS: ES+

Combine (110:112-(101:103+118:120))

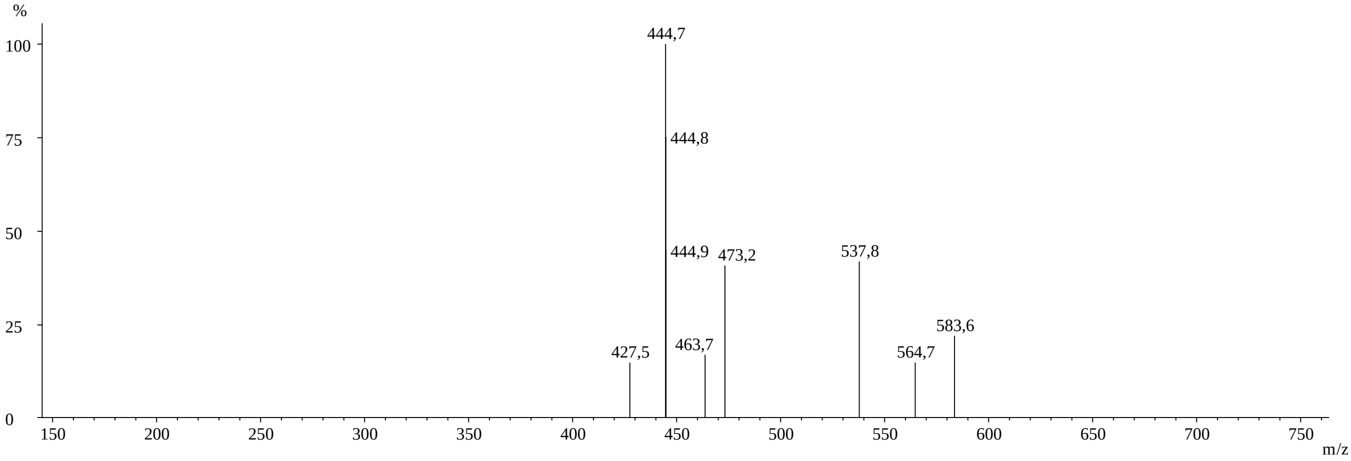

Peak_ID	Compound	Time	Mass found
2		1.86	

MS: ES+

Combine (121:123-(111:113+136:138))

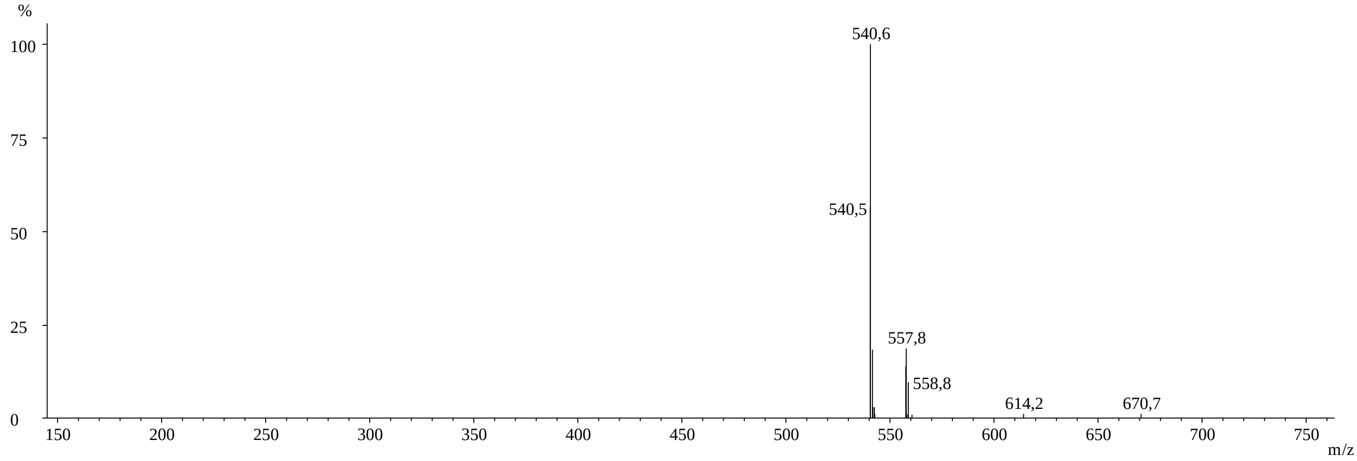

Peak_ID	Compound	Time	Mass found
3	Found	2.04	539.2100

MS: ES+

Combine (128:130-(124:126+137:139))

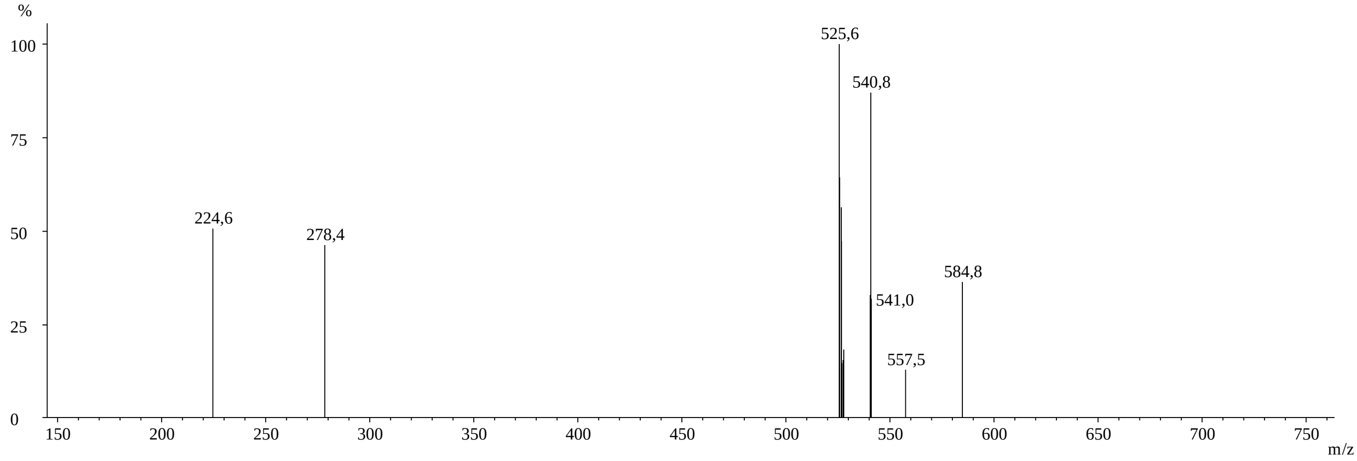

Peak_ID	Compound	Time	Mass found
4	Found	2.15	539.2100

MS: ES+

Combine (152:154-(145:146+161:163))

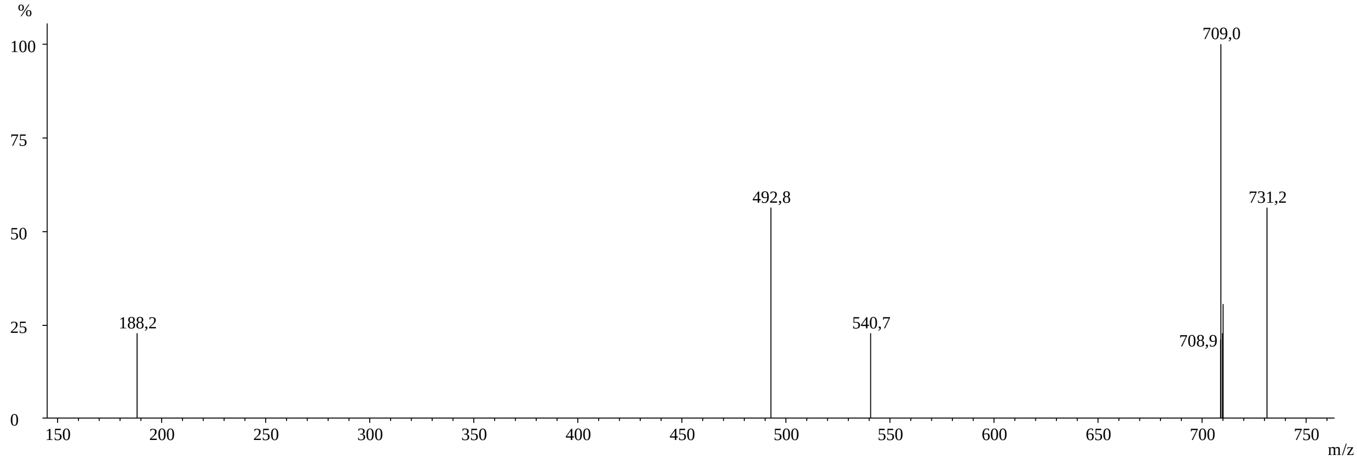

Peak_ID	Compound	Time	Mass found
6		2.56	

MS: ES+

Combine (169:170-(160:162+194:196))

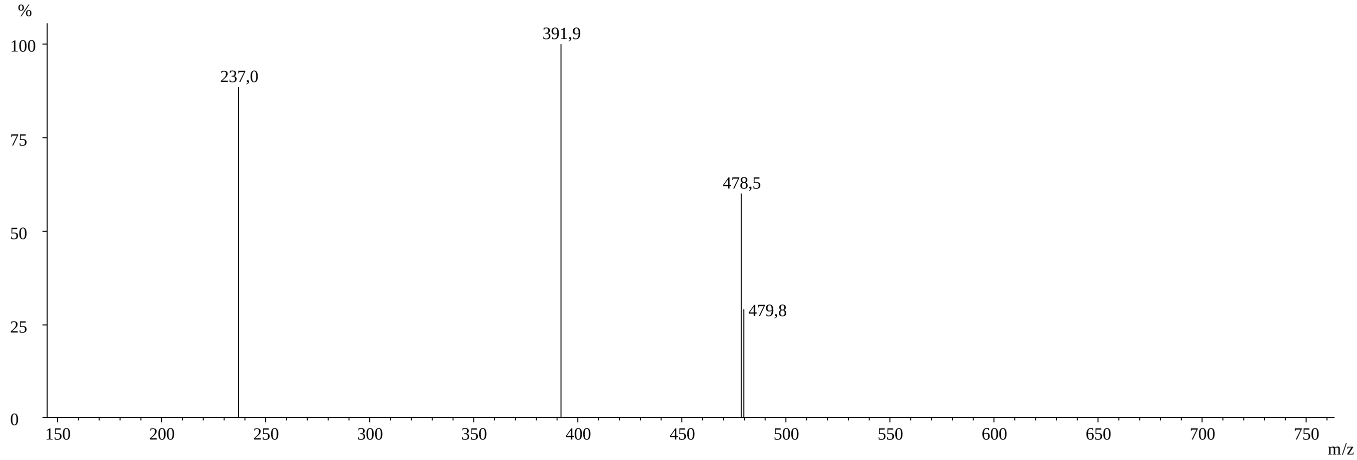

Peak_ID	Compound	Time	Mass found
7		2.84	
