# Supplementary material for: Selective androgen receptor degrader (SARD) to overcome antiandrogen resistance in castration-resistant prostate cancer
Source: eLife. 2023 Jan 19;12:e70700. doi: 10.7554/eLife.70700 (PMC9901937; doi:10.7554/eLife.70700)
Supplement: Source data 2. [file elife-70700-data2.zip › Supplementary Material_source_data/Figure 1-figure supplement 1 & Supplementary1a-source/Z71.PDF]

Sample: 119
File: Ar20357_32
Vial: C/3

Date: 20-Jun-2007
Time: 06:22:29
Description: 20583426

AMRI code: ALB-H10713478
Vial label: 222991-1

DAD: 220

max. intensity: 1.2E6

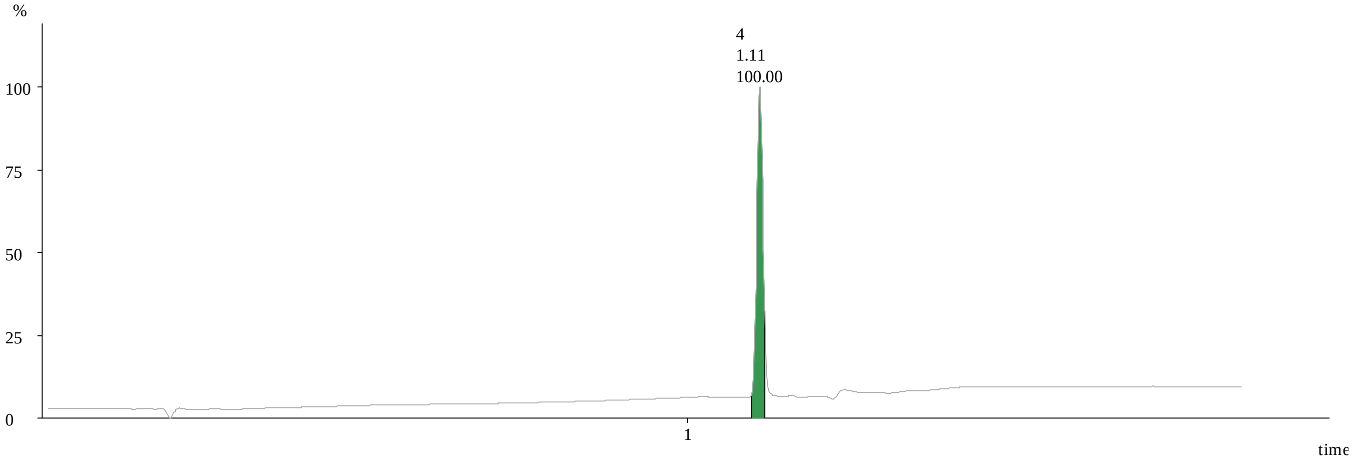

Peak_ID	Peak	Area	Area%	Height	Time	Mass Found
4	1.10 1.12	1.E4	100	1.E6	1.11	540.24

MS ES+ :543.24+1081.48+558.24+541.24

max. intensity: 6.1E6

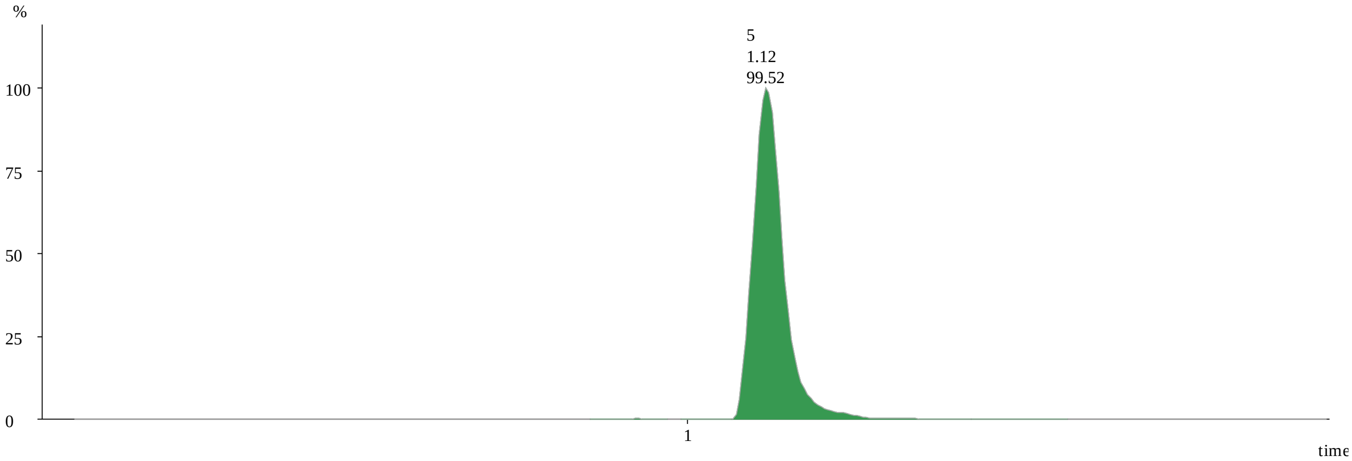

Peak_ID	Peak	Area	Area%	Height	Time	Mass Found
2	0.85 0.97	7.E2	0.21	2.E4	0.92	540.24
3	0.99 1.07	5.E2	0.14	1.E4	1.04	540.24
5	1.07 1.44	3.E5	99.52	6.E6	1.12	540.24
7	1.44 1.59	4.E2	0.11	5.E3	1.49	540.24
9	1.71 1.80	5.E1	0.02	1.E3	1.75	540.24

MS ES+ :TIC

max. intensity: 9.6E6

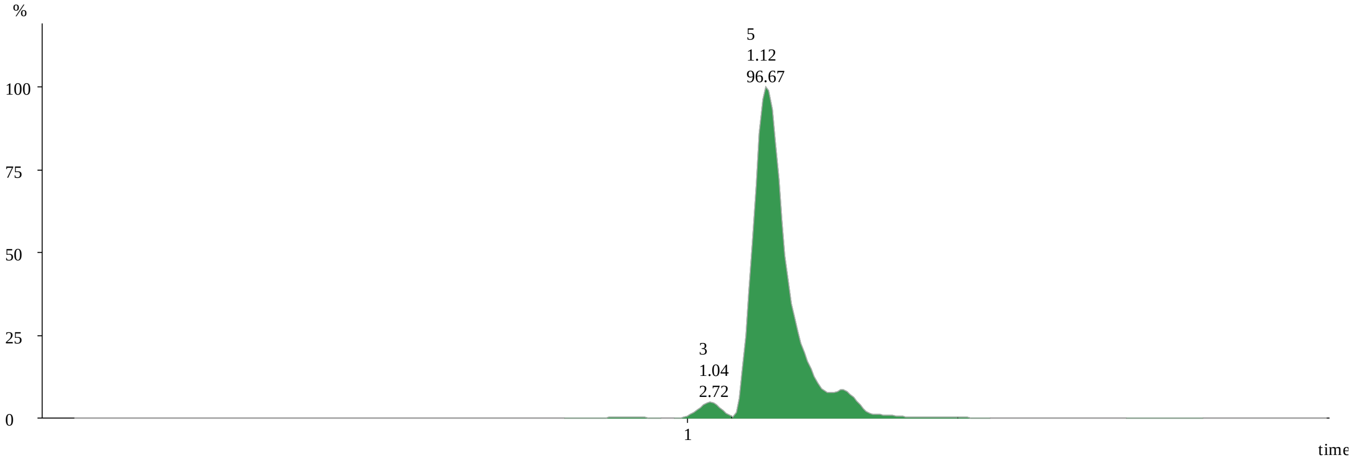

Peak_ID	Peak	Area	Area%	Height	Time	Mass Found
1	0.81 0.96	3.E3	0.47	5.E4	0.91	540.24
3	0.98 1.07	2.E4	2.72	4.E5	1.04	540.24
5	1.07 1.42	6.E5	96.67	1.E7	1.12	540.24
6	1.42 1.47	3.E2	0.05	1.E4	1.43	540.24
8	1.68 1.80	6.E2	0.1	1.E4	1.73	540.24

MS: ES+

Combine (217:223-(204:210+231:237))

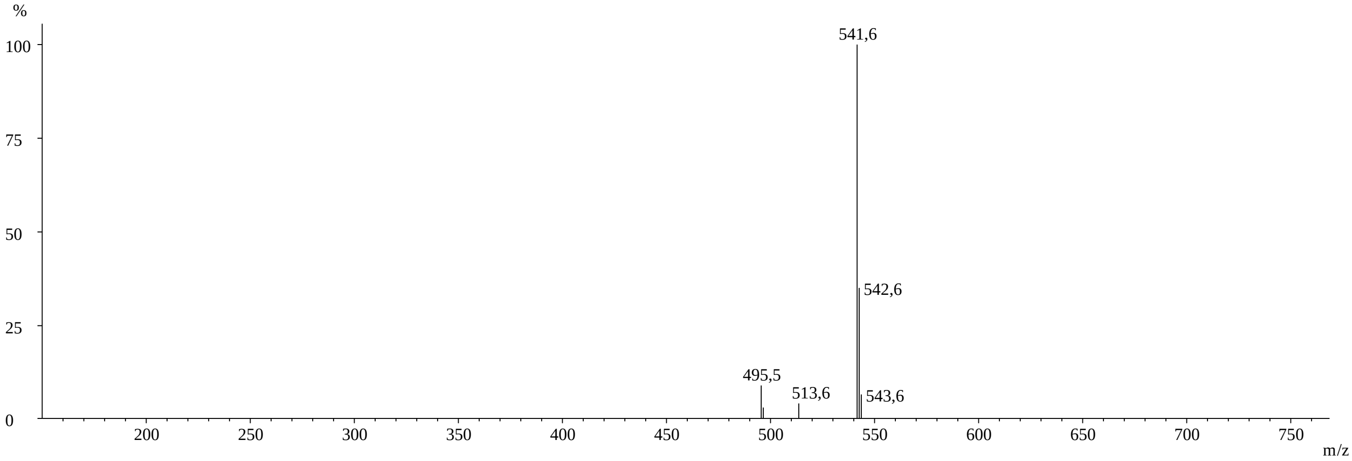

Peak_ID	Compound	Time	Mass found
4	Found	1.11	540.2400
