# Supplementary material for: Selective androgen receptor degrader (SARD) to overcome antiandrogen resistance in castration-resistant prostate cancer
Source: eLife. 2023 Jan 19;12:e70700. doi: 10.7554/eLife.70700 (PMC9901937; doi:10.7554/eLife.70700)
Supplement: Source data 2. [file elife-70700-data2.zip › Supplementary Material_source_data/Figure 1-figure supplement 1 & Supplementary1a-source/Z75.PDF]

Sample: 136	Date: 27-Feb-2004	Page 1.
File: 8324_36	Time: 16:39:52	AMRI code: ALB-H05204887
Vial: D/5	Description: 909640	Vial label: M5173042AMS0020

DAD: 220

max. intensity: 4E6

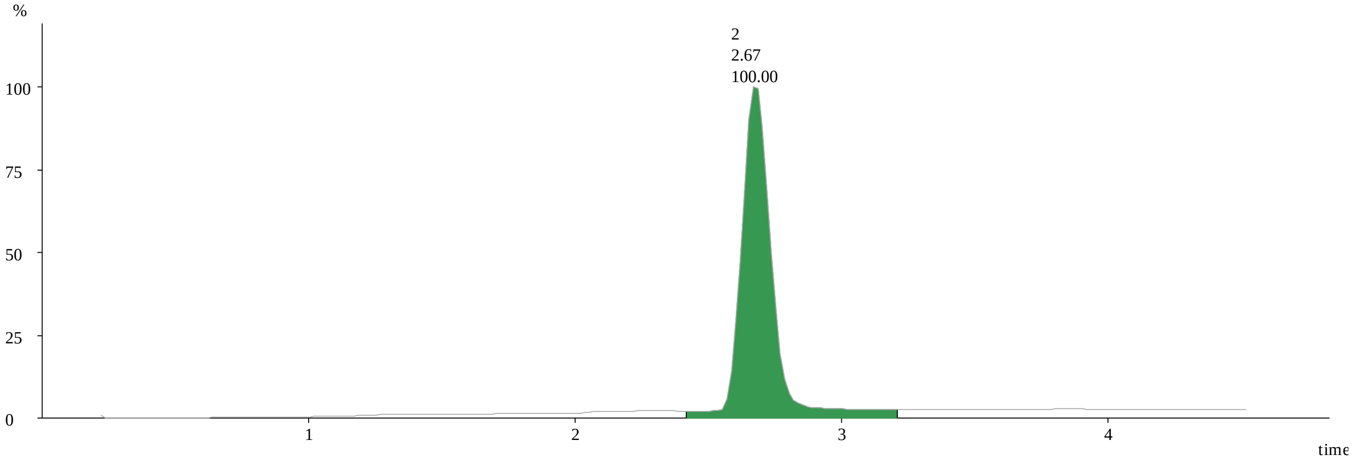

Peak_ID	Peak	Area	Area%	Height	Time	Mass Found
2	2.42 3.21	5.E5	100	4.E6	2.67	524.27

MS ES+ :542.27+525.27

max. intensity: 5.3E4

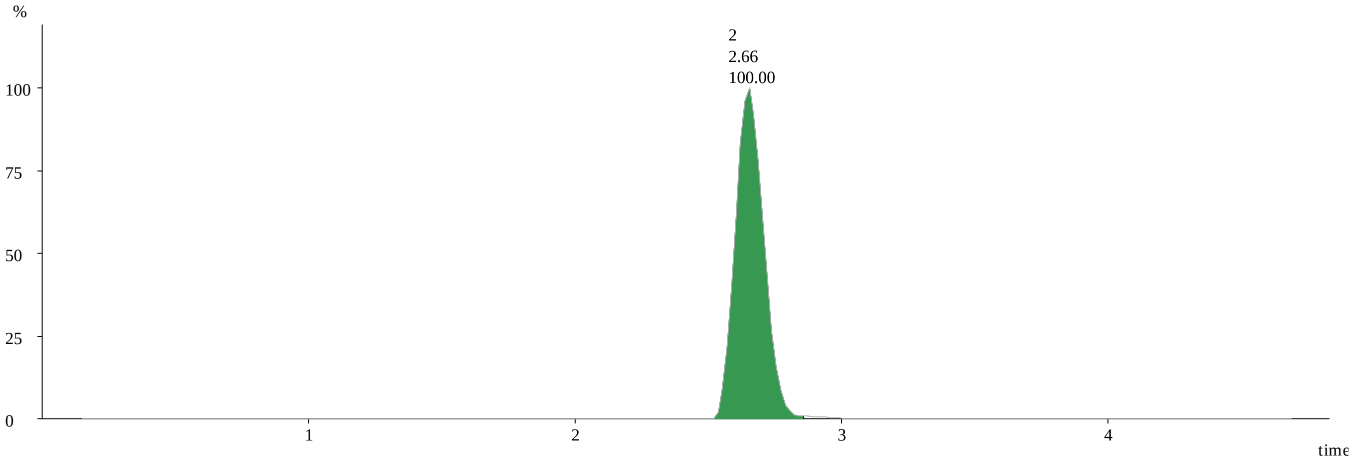

Peak_ID	Peak	Area	Area%	Height	Time	Mass Found
2	2.52 2.86	7.E3	100	5.E4	2.66	524.27

Sample: 136	Date: 27-Feb-2004	Page 2.
File: 8324_36	Time: 16:39:52	AMRI code: ALB-H05204887
Vial: D/5	Description: 909640	Vial label: M5173042AMS0020

MS ES+ :TIC

max. intensity: 7.1E4

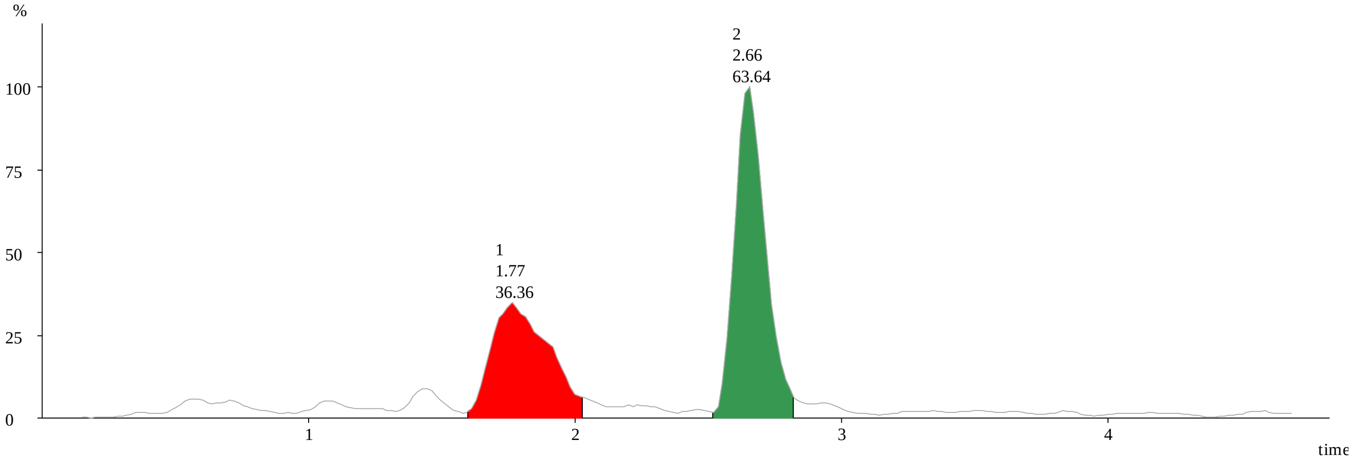

Peak_ID	Peak	Area	Area%	Height	Time	Mass Found
1	1.60 2.03	5.E3	36.36	2.E4	1.77	
2	2.52 2.82	9.E3	63.64	7.E4	2.66	524.27

MS: ES+

Combine (157:159-(146:148+172:174))

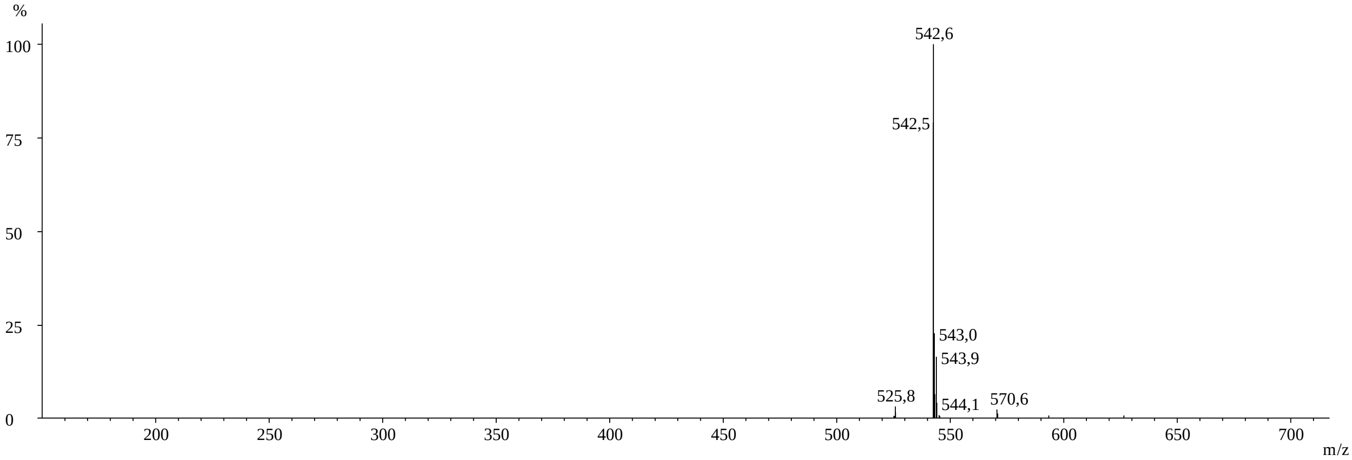

Peak_ID	Compound	Time	Mass found
2	Found	2.66	524.2700
