# Supplementary material for: Selective androgen receptor degrader (SARD) to overcome antiandrogen resistance in castration-resistant prostate cancer
Source: eLife. 2023 Jan 19;12:e70700. doi: 10.7554/eLife.70700 (PMC9901937; doi:10.7554/eLife.70700)
Supplement: Source data 2. [file elife-70700-data2.zip › Supplementary Material_source_data/Figure 1-figure supplement 1 & Supplementary1a-source/Z76.PDF]

Sample: 185
File: Ar23492_85
Vial: E/11

Date: 22-Apr-2008
Time: 02:50:28
Description: 10165737

AMRI code: ALB-H01332277
Vial label: M1305155ACC0031

(1) ELSD Signal

max. intensity: 6.3E5

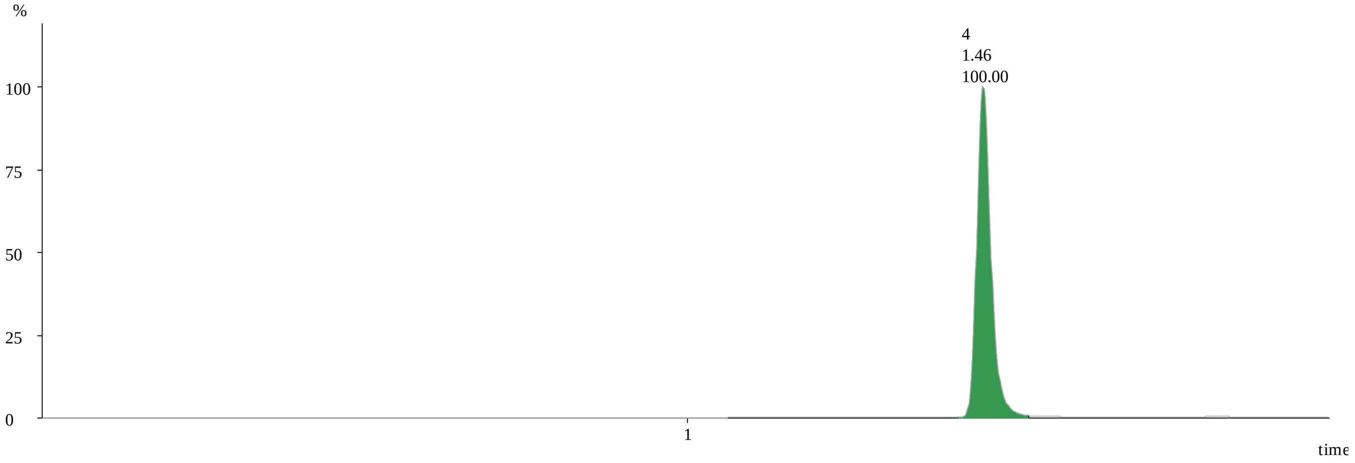

Peak_ID	Peak	Area	Area%	Height	Time	Mass Found
4	1.42 1.53	2.E4	100	6.E5	1.46	520.26

DAD: 220

max. intensity: 1.7E6

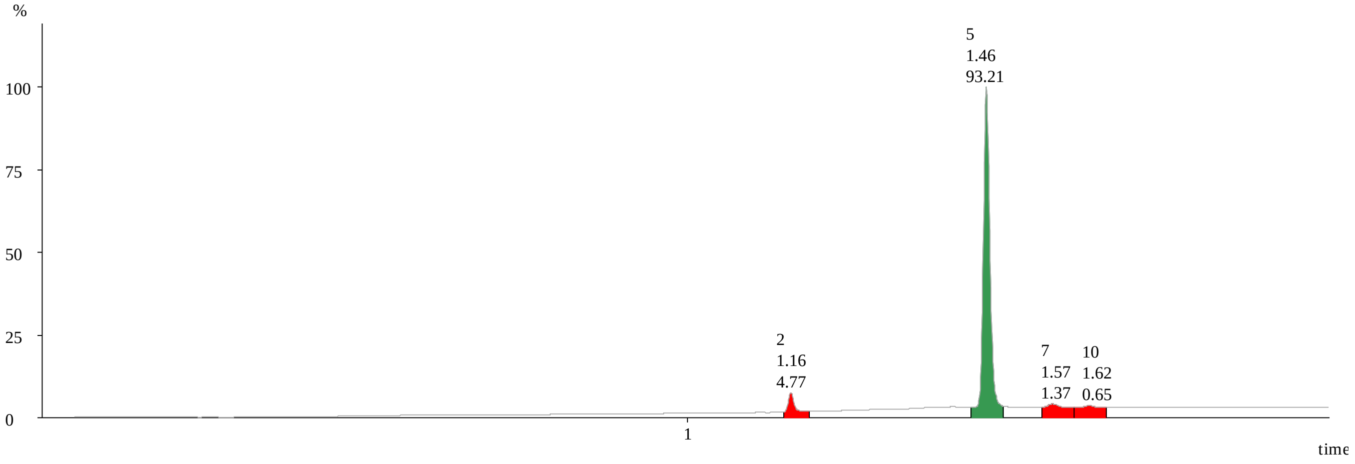

Peak_ID	Peak	Area	Area%	Height	Time	Mass Found
2	1.15 1.19	1.E3	4.77	1.E5	1.16	
5	1.44 1.49	2.E4	93.21	2.E6	1.46	520.26
7	1.55 1.60	3.E2	1.37	2.E4	1.57	
10	1.60 1.65	1.E2	0.65	1.E4	1.62	

MS AP+ :1041.52+538.26+521.26

max. intensity: 4.1E7

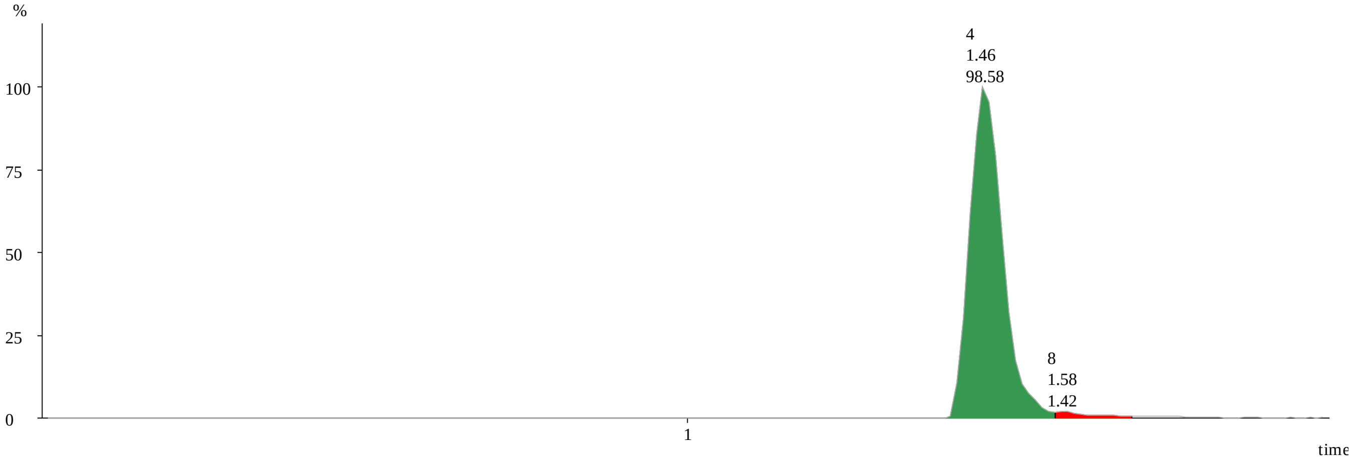

Peak_ID	Peak	Area	Area%	Height	Time	Mass Found
4	1.40 1.57	2.E6	98.58	4.E7	1.46	520.26
8	1.57 1.69	4.E4	1.42	7.E5	1.58	

MS AP+ :TIC

max. intensity: 9.9E7

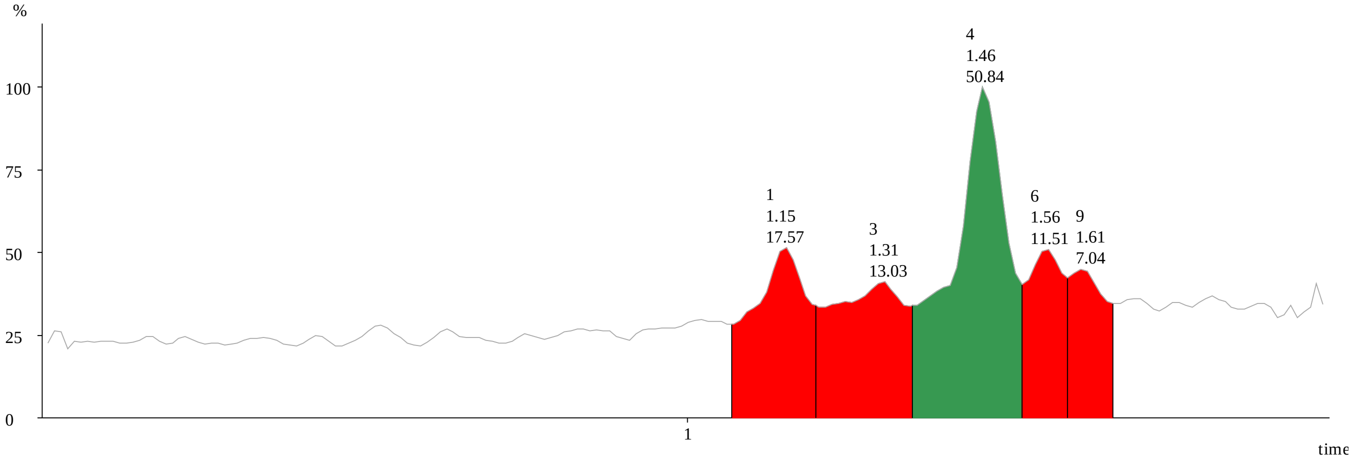

Peak_ID	Peak	Area	Area%	Height	Time	Mass Found
1	1.07 1.20	2.E6	17.57	2.E7	1.15	
3	1.20 1.35	1.E6	13.03	1.E7	1.31	
4	1.35 1.52	5.E6	50.84	7.E7	1.46	520.26
6	1.52 1.59	1.E6	11.51	2.E7	1.56	
9	1.59 1.66	7.E5	7.04	1.E7	1.61	

Sample: 185 | Date: 22-Apr-2008 | Page 3.
File: Ar23492_85 | Time: 02:50:28 | AMRI code: ALB-H01332277
Vial: E/11 | Description: 10165737 | Vial label: M1305155ACC0031

MS: AP+

Combine (113:116-(106:109+121:124))

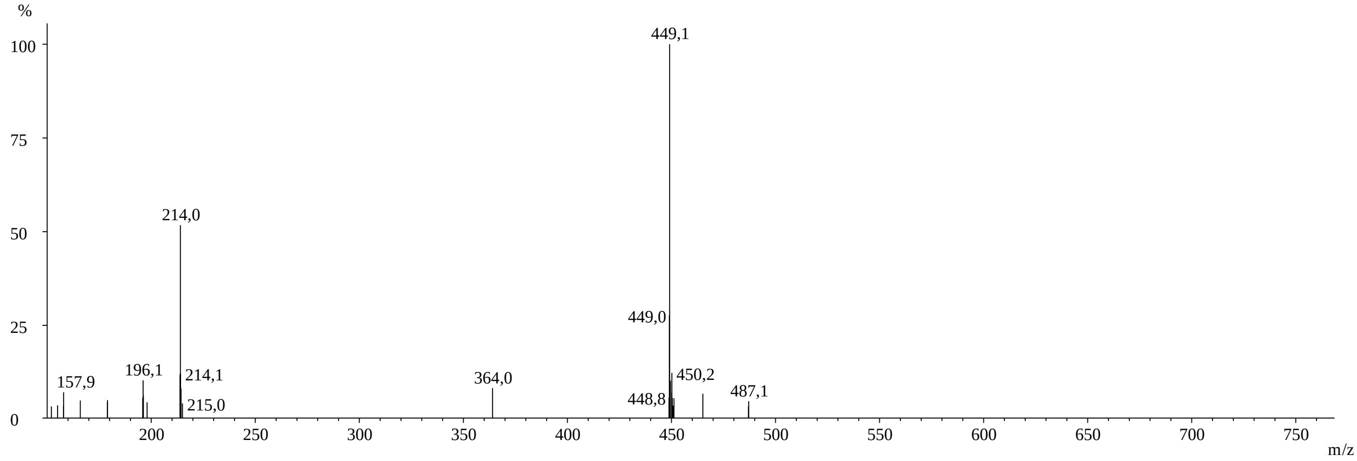

Peak_ID	Compound	Time	Mass found
2		1.16	

MS: AP+

Combine (143:146-(136:139+151:154))

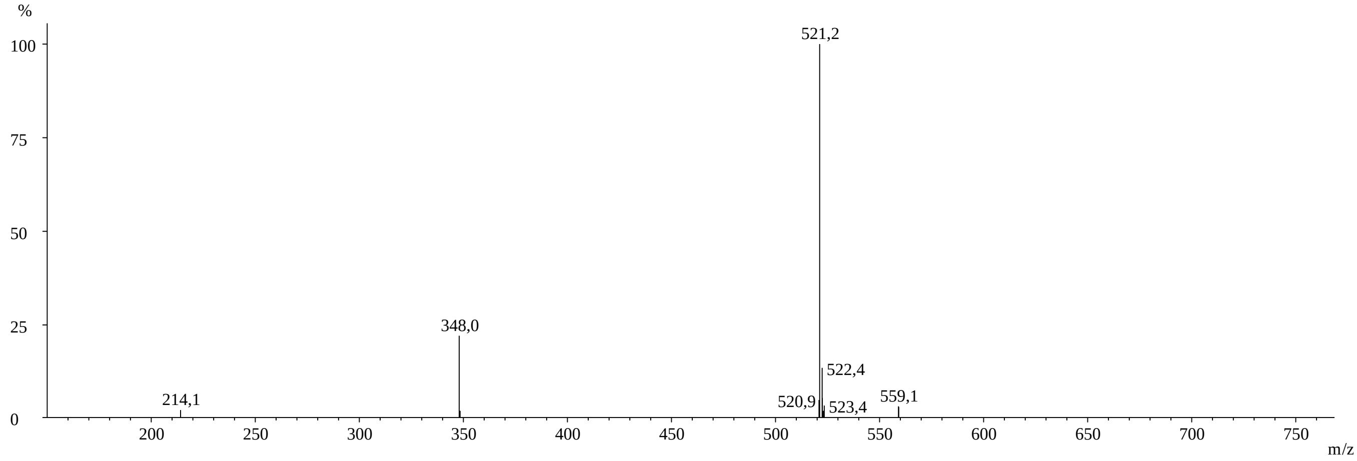

Peak_ID	Compound	Time	Mass found
5	Found	1.46	520.2600

MS: AP+

Combine (153:156-(146:149+162:165))

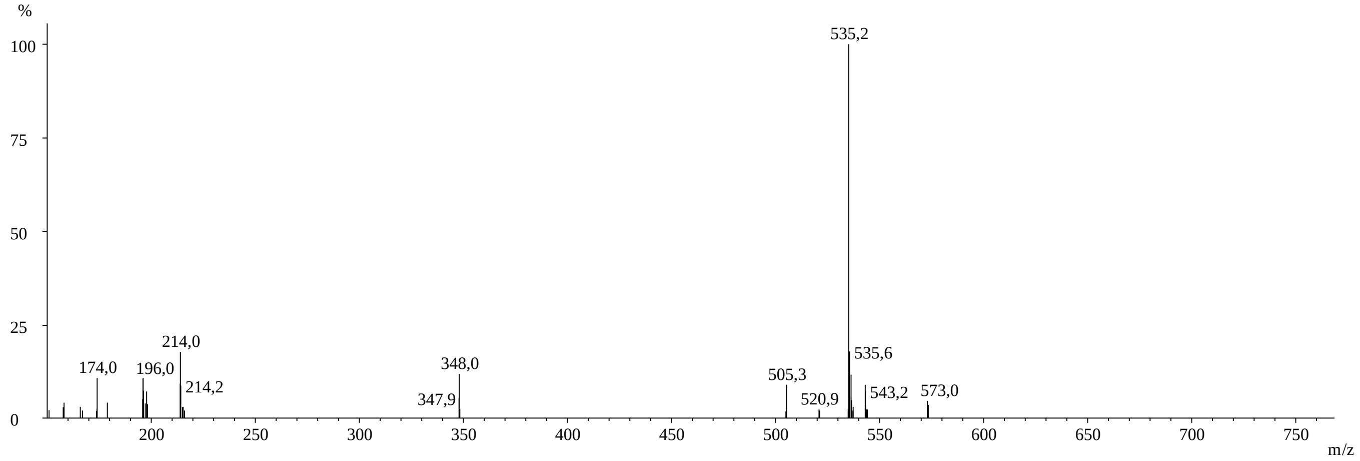

Peak_ID	Compound	Time	Mass found
7		1.57	
