# Supplementary material for: Selective androgen receptor degrader (SARD) to overcome antiandrogen resistance in castration-resistant prostate cancer
Source: eLife. 2023 Jan 19;12:e70700. doi: 10.7554/eLife.70700 (PMC9901937; doi:10.7554/eLife.70700)
Supplement: Source data 2. [file elife-70700-data2.zip › Supplementary Material_source_data/Figure 1-figure supplement 1 & Supplementary1a-source/Z79.PDF]

Sample: 16
File: Ar17704_68
Vial: F/1

Date: 10-Oct-2006
Time: 13:43:29
Description: 20370720

AMRI code: ALB-H11202313
Vial label: 300000194253

DAD: 220

max. intensity: 1.6E6

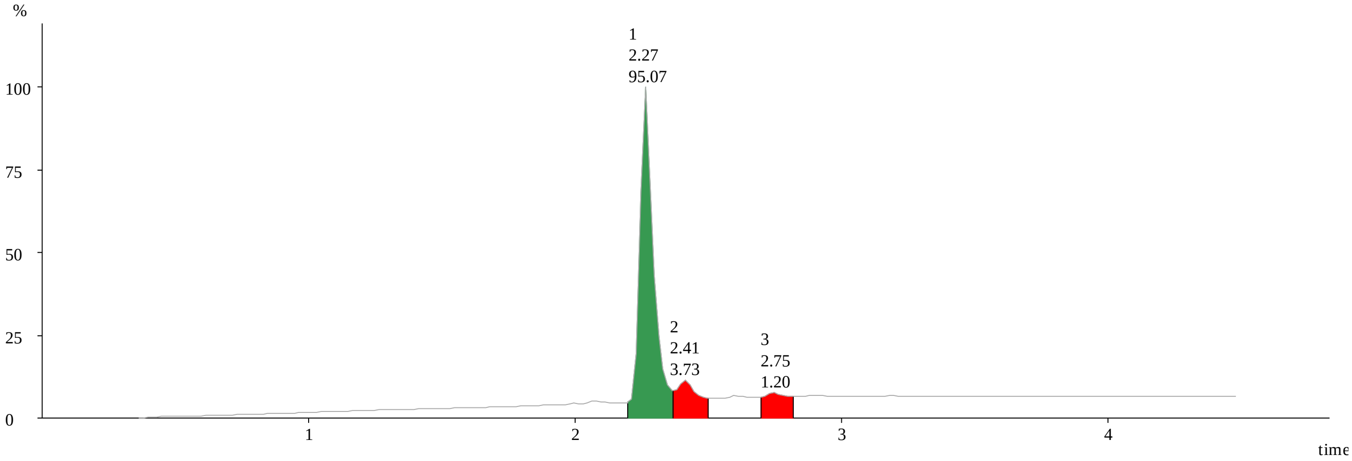

Peak_ID	Peak	Area	Area%	Height	Time	Mass Found
1	2.20 2.37	8.E4	95.07	2.E6	2.27	525.19
2	2.37 2.50	3.E3	3.73	7.E4	2.41	
3	2.70 2.82	1.E3	1.2	2.E4	2.75	

MS ES+ :543.19+1051.38+526.19

max. intensity: 5.7E4

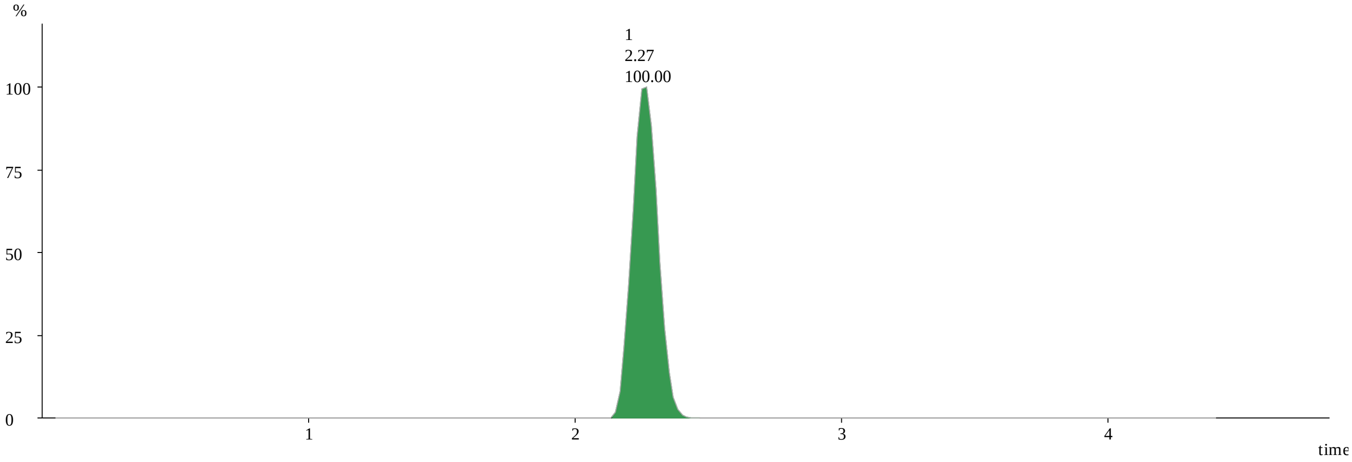

Peak_ID	Peak	Area	Area%	Height	Time	Mass Found
1	2.14 2.47	6.E3	100	6.E4	2.27	525.19

Sample: 16	Date: 10-Oct-2006	
File: Ar17704_68	Time: 13:43:29	AMRI code: ALB-H11202313
Vial: F/1	Description: 20370720	Vial label: 300000194253

MS ES+ :TIC

max. intensity: 7.5E4

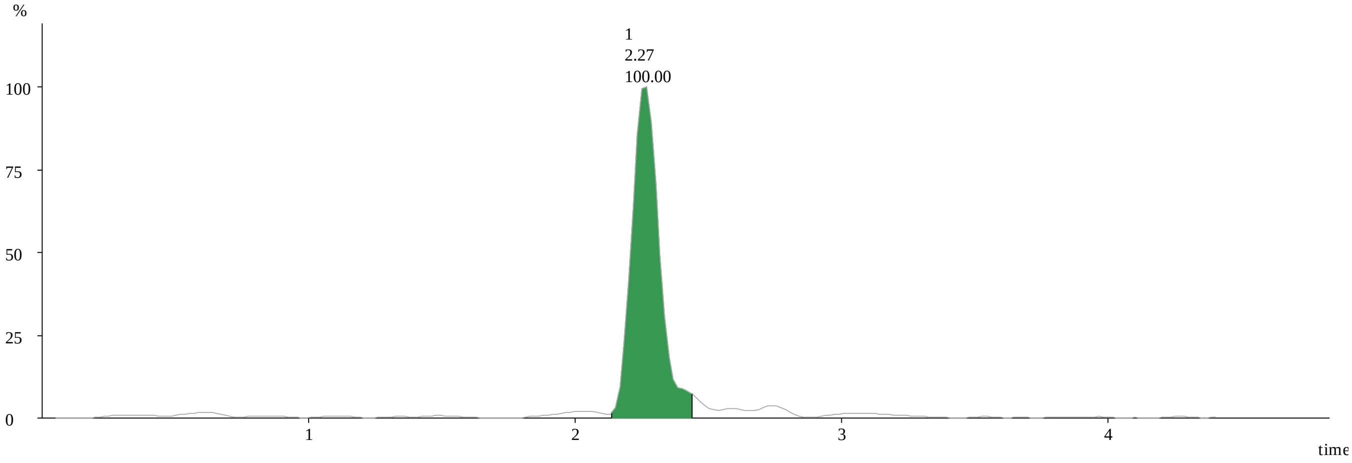

Peak_ID	Peak	Area	Area%	Height	Time	Mass Found
1	2.14 2.44	8.E3	100	7.E4	2.27	525.19

MS: ES+

Combine (134:136-(123:125+147:149))

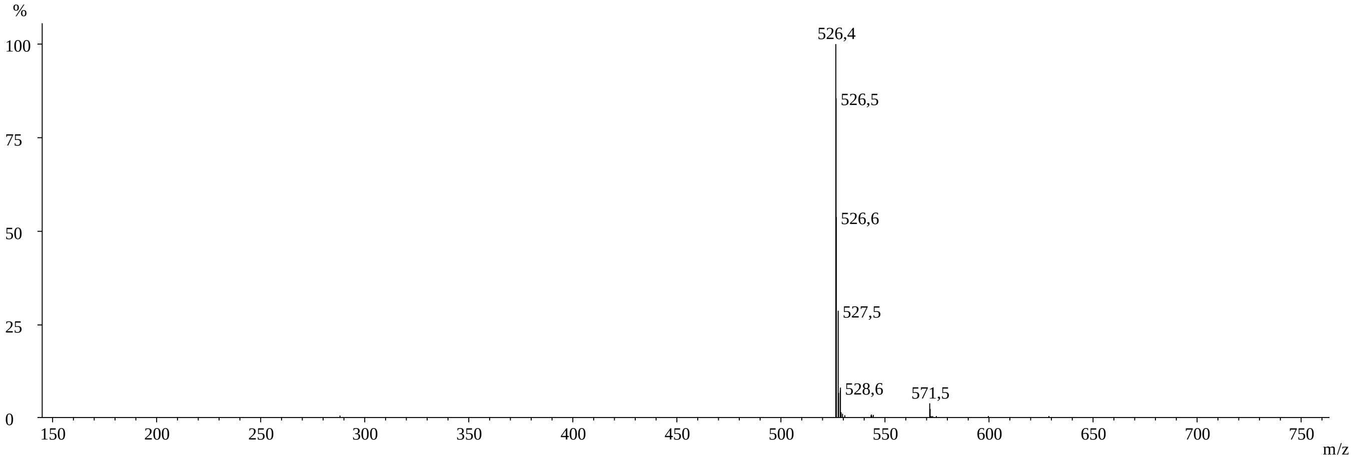

Peak_ID	Compound	Time	Mass found
1	Found	2.27	525.1900

MS: ES+

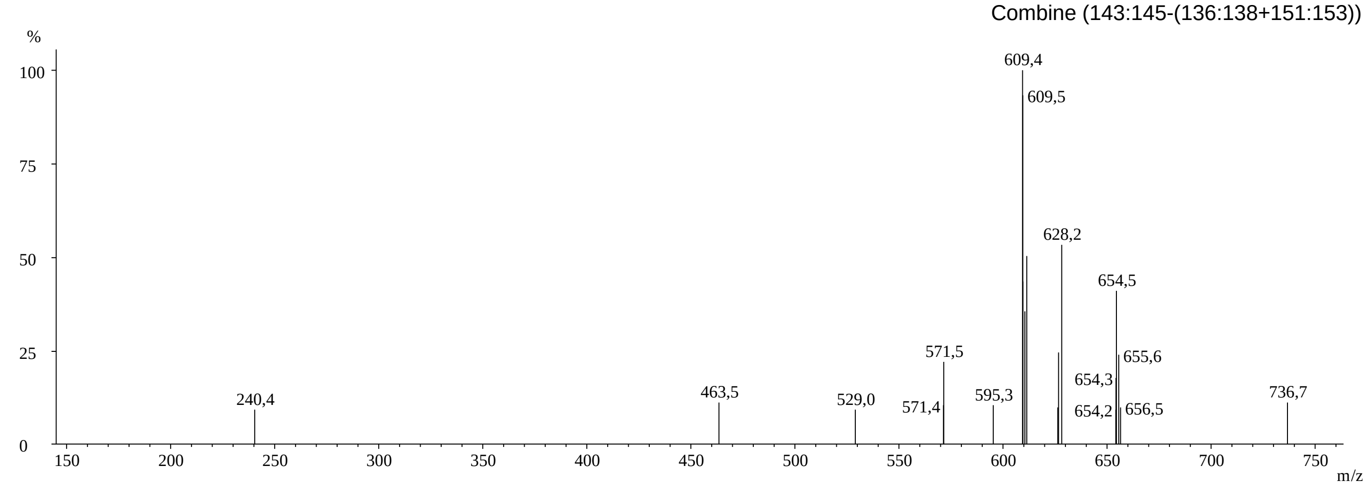

Peak_ID	Compound	Time	Mass found
2		2.41	

MS: ES+

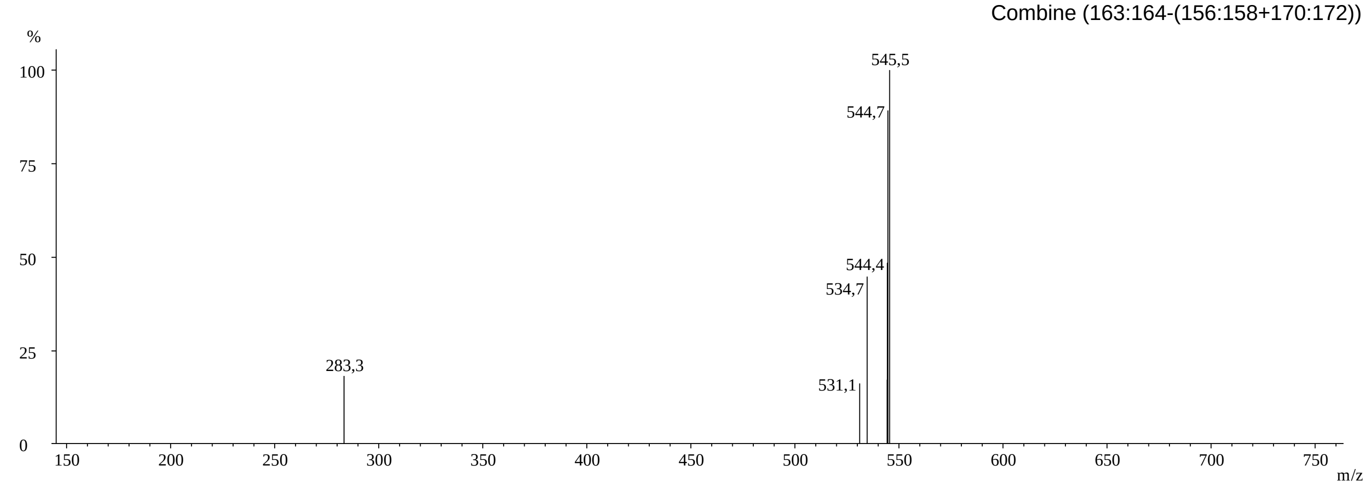

Peak_ID	Compound	Time	Mass found
3		2.75	
