# Supplementary material for: Selective androgen receptor degrader (SARD) to overcome antiandrogen resistance in castration-resistant prostate cancer
Source: eLife. 2023 Jan 19;12:e70700. doi: 10.7554/eLife.70700 (PMC9901937; doi:10.7554/eLife.70700)
Supplement: Source data 2. [file elife-70700-data2.zip › Supplementary Material_source_data/Figure 1-figure supplement 1 & Supplementary1a-source/Z8.PDF]

Sample Name: 1231624

DFN: D:\DATA\MAY\05_15\V0515_02\SAMPL044.D

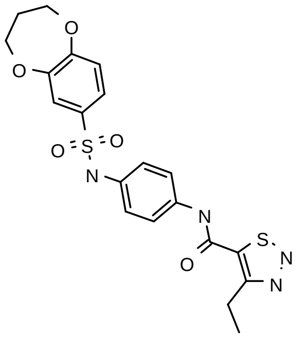

mw = 460,53

MaxPeak: 100.00%   Ret_Time: 0.700 min

The method for the Gradient Sample using short rapid resolution HT Cartridge ZORBAX SB-C18 4.6x15 mm (p/n 821975-932). For testing purity of syntez.

#	Time	Area%
1	0.700	100.00

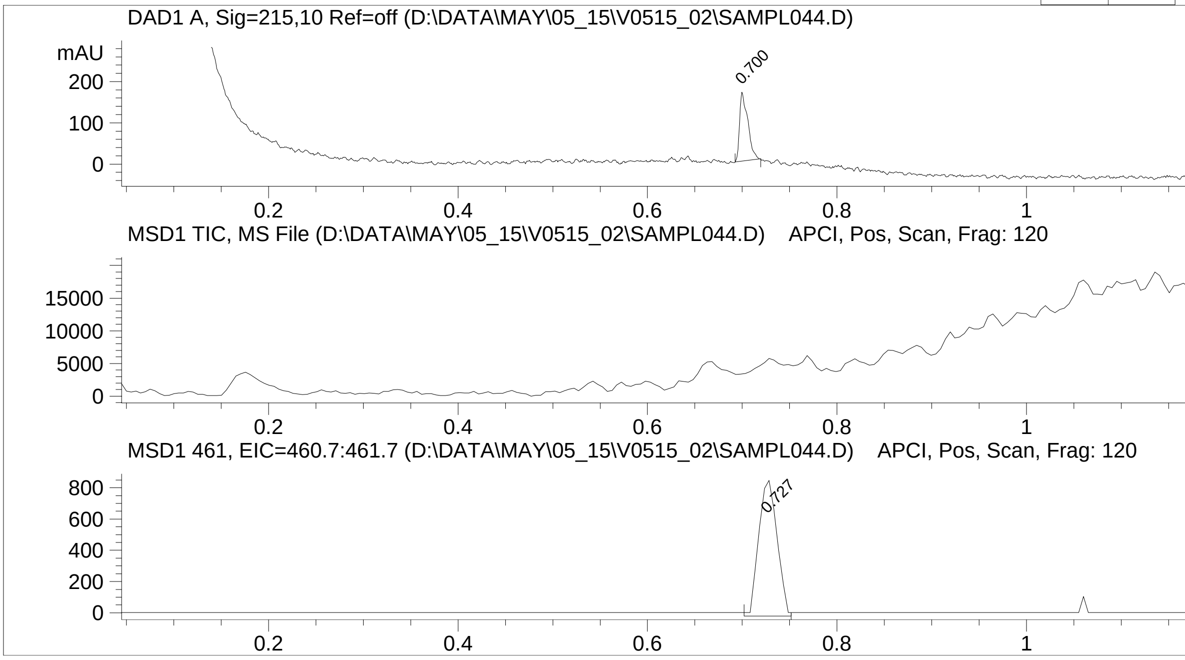

RT 0.727

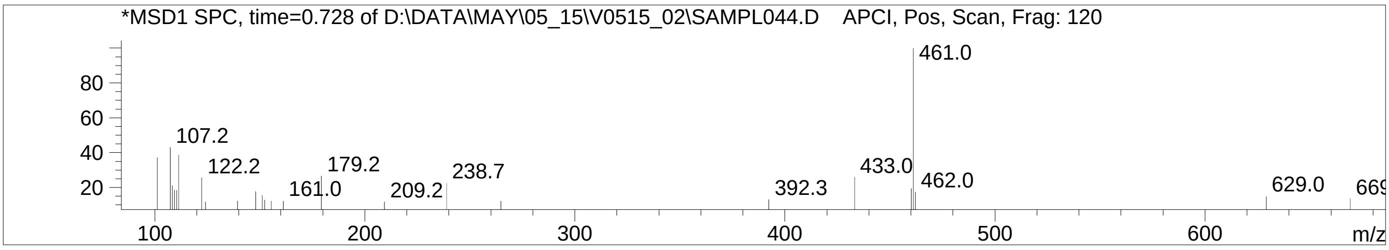

Inj.Date 15.05.2006   L   P2-E-08   Acq. Method SB15TFA3.M
