# Supplementary material for: Selective androgen receptor degrader (SARD) to overcome antiandrogen resistance in castration-resistant prostate cancer
Source: eLife. 2023 Jan 19;12:e70700. doi: 10.7554/eLife.70700 (PMC9901937; doi:10.7554/eLife.70700)
Supplement: Source data 2. [file elife-70700-data2.zip › Supplementary Material_source_data/Figure 1-figure supplement 1 & Supplementary1a-source/Z80.PDF]

Sample: 144	Date: 19-Jul-2007	Page 1.
File: Ar20781_44	Time: 05:20:51	AMRI code: ALB-H10738727
Vial: D/6	Description: 20588572	Vial label: 279138-1

DAD: 220

max. intensity: 2.6E6

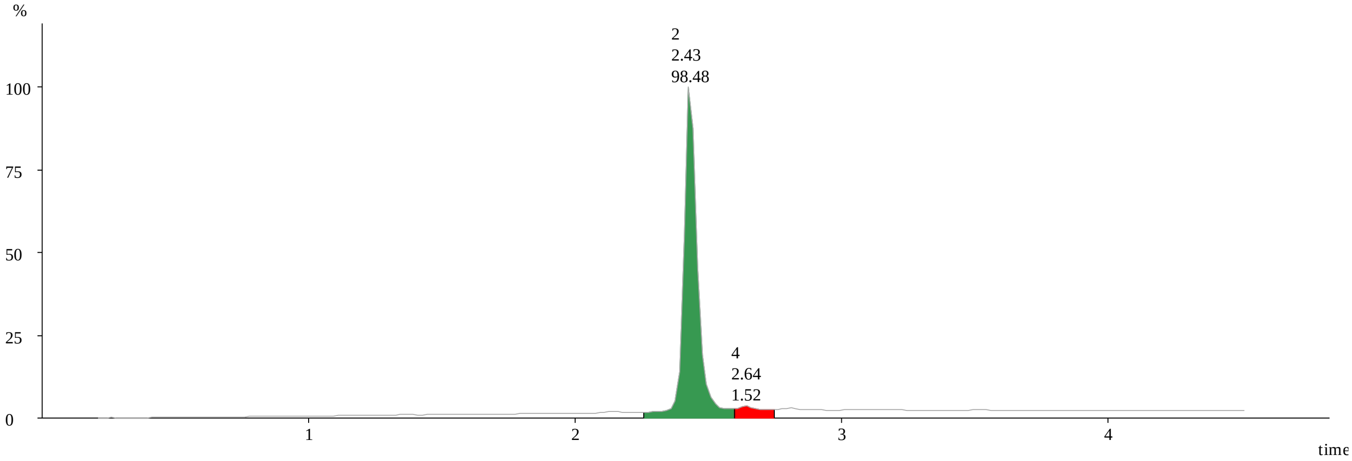

Peak_ID	Peak	Area	Area%	Height	Time	Mass Found
2	2.26 2.60	1.E5	98.48	3.E6	2.43	543.2
4	2.60 2.75	2.E3	1.52	3.E4	2.64	

MS ES+ :561.2+544.2

max. intensity: 1.5E3

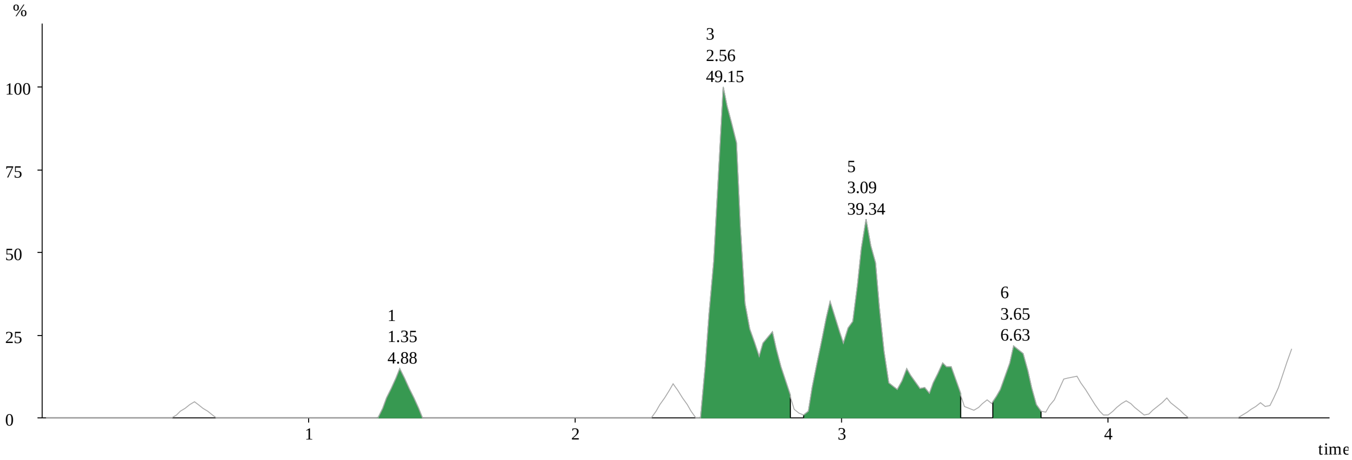

Peak_ID	Peak	Area	Area%	Height	Time	Mass Found
1	1.26 1.43	2.E1	4.88	2.E2	1.35	543.2
3	2.47 2.81	2.E2	49.15	1.E3	2.56	543.2
5	2.86 3.45	1.E2	39.34	8.E2	3.09	543.2
6	3.57 3.75	3.E1	6.63	3.E2	3.65	543.2

Sample: 144	Date: 19-Jul-2007	Page 2.
File: Ar20781_44	Time: 05:20:51	AMRI code: ALB-H10738727
Vial: D/6	Description: 20588572	Vial label: 279138-1

MS ES+ :TIC

max. intensity: 8.6E4

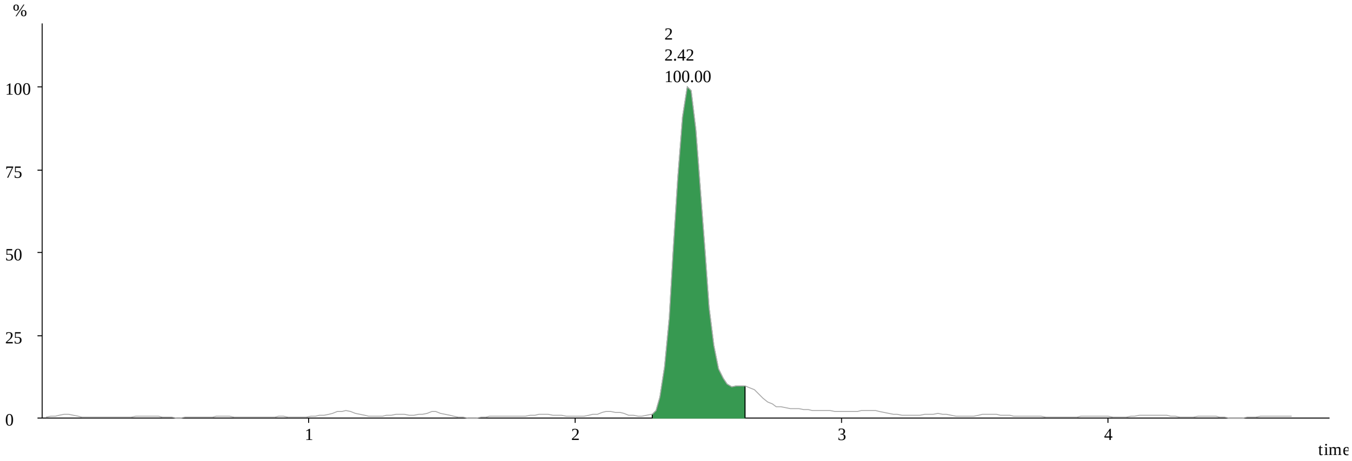

Peak_ID	Peak	Area	Area%	Height	Time	Mass Found
2	2.29 2.64	1.E4	100	8.E4	2.42	543.2

MS: ES+

Combine (143:145-(132:134+159:161))

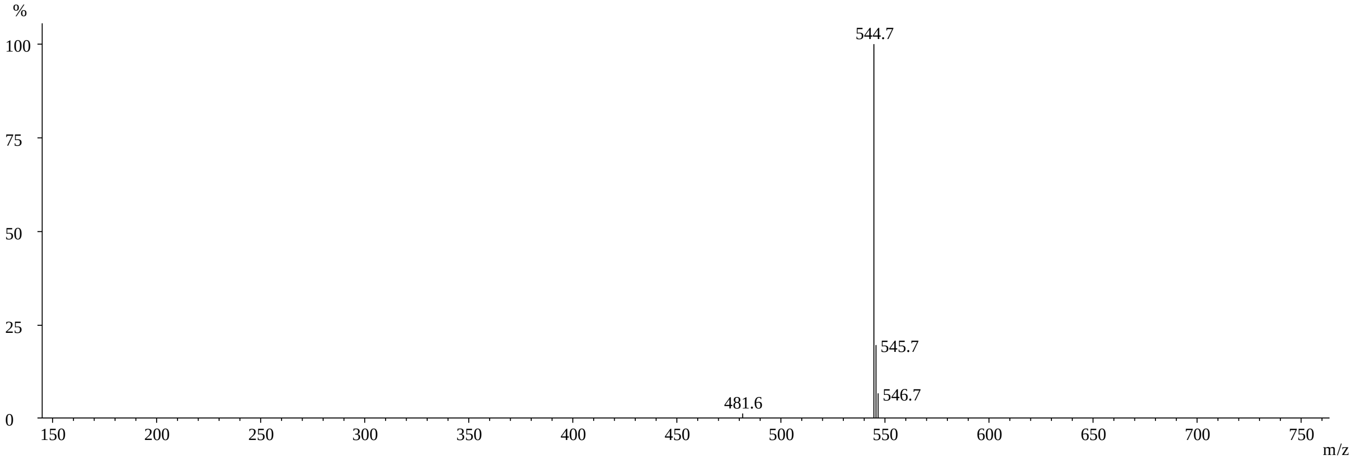

Peak_ID	Compound	Time	Mass found
2	Found	2.42	543.2000

Sample: 144	Date: 19-Jul-2007	Page 3.
File: Ar20781_44	Time: 05:20:51	AMRI code: ALB-H10738727
Vial: D/6	Description: 20588572	Vial label: 279138-1

MS: ES+

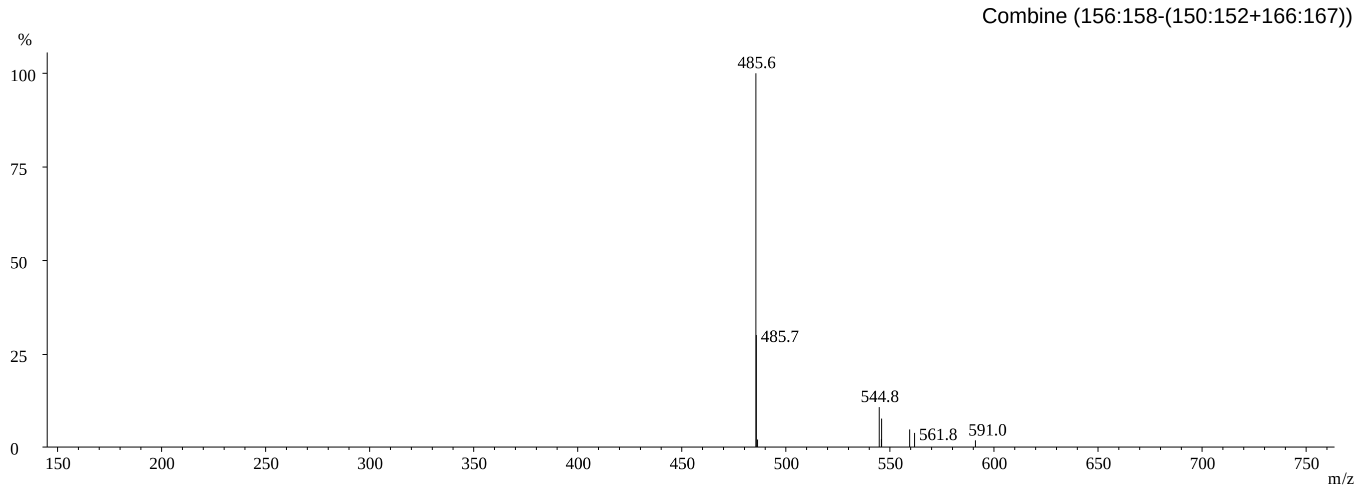

Peak_ID	Compound	Time	Mass found
4		2.64	
