# Supplementary material for: Selective androgen receptor degrader (SARD) to overcome antiandrogen resistance in castration-resistant prostate cancer
Source: eLife. 2023 Jan 19;12:e70700. doi: 10.7554/eLife.70700 (PMC9901937; doi:10.7554/eLife.70700)
Supplement: Source data 2. [file elife-70700-data2.zip › Supplementary Material_source_data/Figure 8-figure supplement 1 & Supplementary file 1c-source/ZL-1-F0473-0018-98.pdf]

-.o.-Syntez Purity Report -.o.-

Agilent 1100 LC/MSD SL
Diodearrey G1315B (DAD1A-215nm; DAD1B-254nm)
Mass Quad G1956B (MSD1-Pos, MSD2-Neg)
ELSD Altech 3300 (ADC1 A, ELSD)

Mobile Phase:A-H2O+0.1%HCOOH;B-MeCN+0.1HCOOH
Separation column:
Rapid Resolutionn HT Cartige 4.6x30mm,
1.8-Micron, Zorbx SB-C18

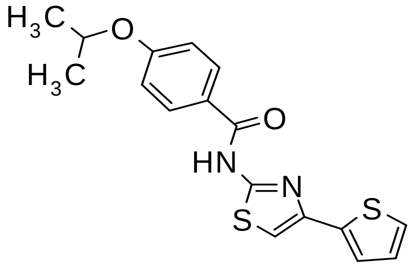

Mol.Weight: 344,46

F0473-0018

M11768 ->

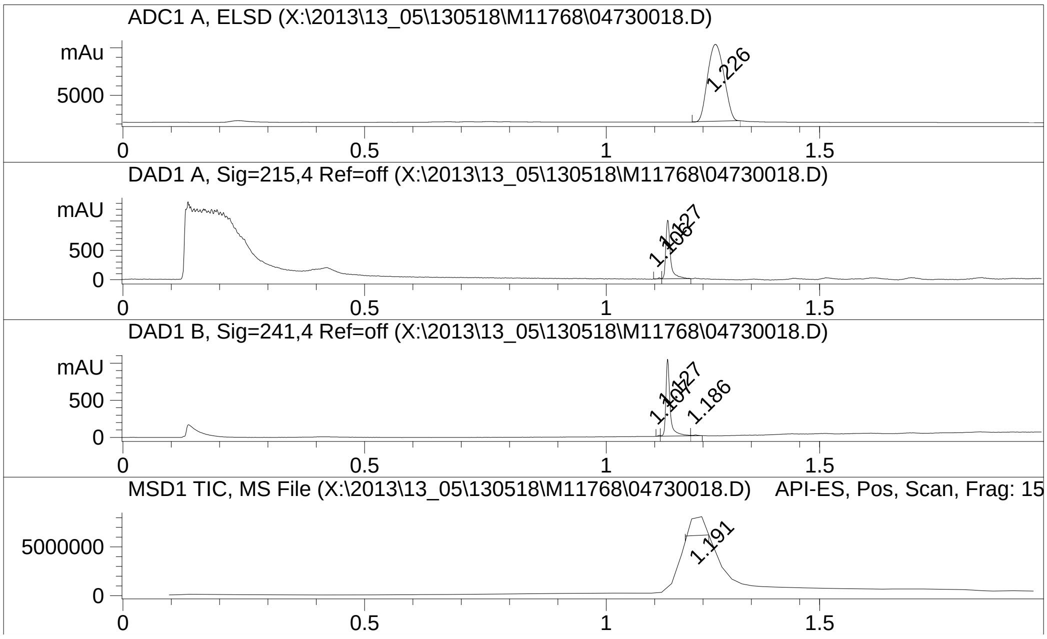

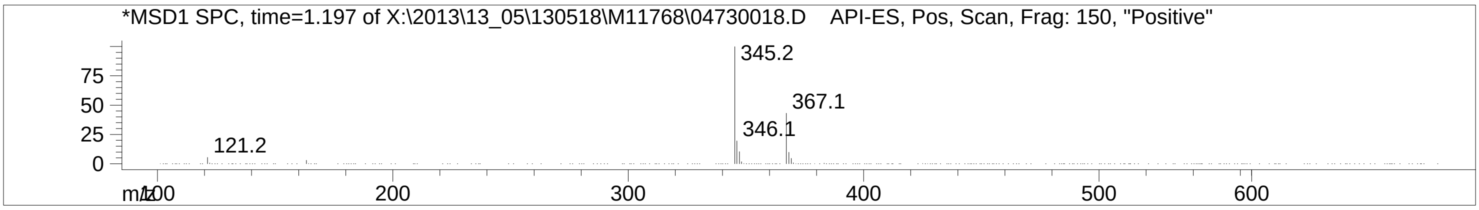

#	Signal	R.Time	Area %
1	ADC1 A, ELSD	1.226	100.000

#	Signal	R.Time	Area %
1	DAD1 A, Sig=215,4 Ref=off	1.106	1.095
2		1.127	98.905

#	Signal	R.Time	Area %
1	DAD1 B, Sig=241,4 Ref=off	1.107	0.697
2		1.127	97.841
3		1.186	1.462

#	Signal	R.Time	Area %
1	MSD1 TIC, MS File	1.191	100.000
