# Supplementary material for: Selective androgen receptor degrader (SARD) to overcome antiandrogen resistance in castration-resistant prostate cancer
Source: eLife. 2023 Jan 19;12:e70700. doi: 10.7554/eLife.70700 (PMC9901937; doi:10.7554/eLife.70700)
Supplement: Source data 2. [file elife-70700-data2.zip › Supplementary Material_source_data/Figure 8-figure supplement 1 & Supplementary file 1c-source/ZL-2-Z28306927.PDF]

Sample Name: 1188094   OK

DFN: C:\HPCHEM\1\DATA\02_28\02_27_05\
SAMPL000.D

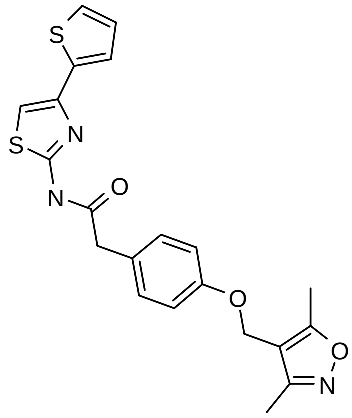

mw = 425,53

MaxPeak: 98.44%   Ret_Time: 0.690 min

The method for the Gradient Sample using short rapid resolution HT Cartridge ZORBAX SB-C18 4.6x15 mm (p/n 821975-932). For testing purity of syntez.

#	Time	Area%
1	0.521	1.56
2	0.690	98.44

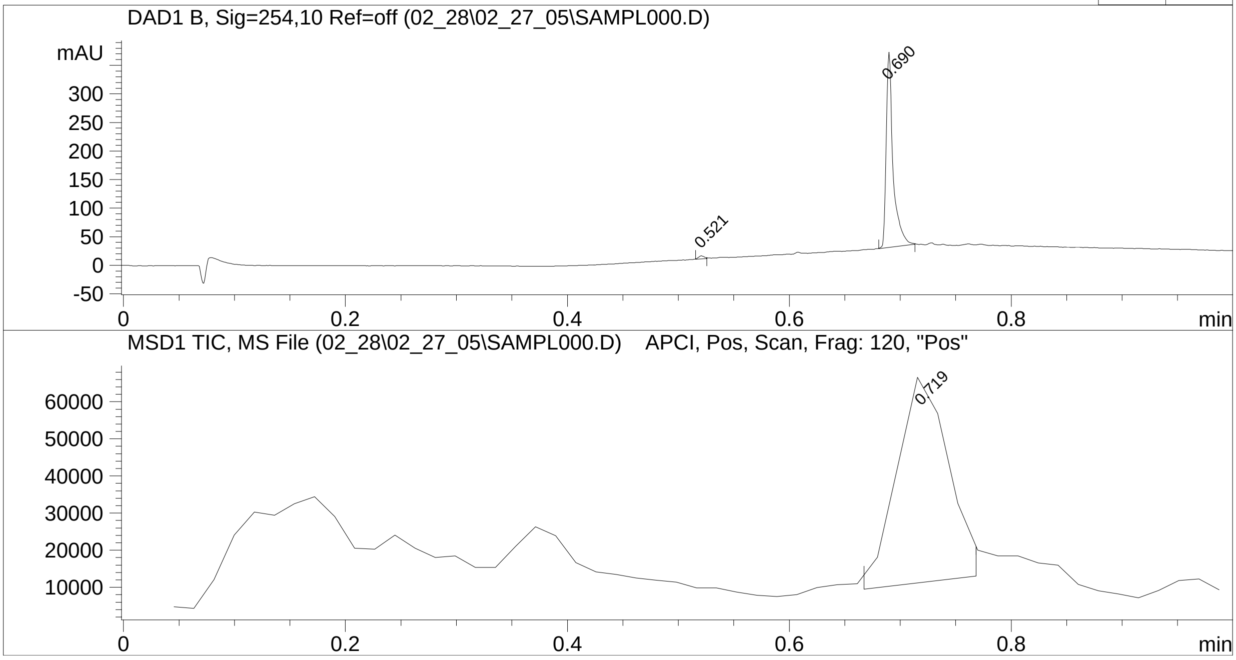

RT 0.719

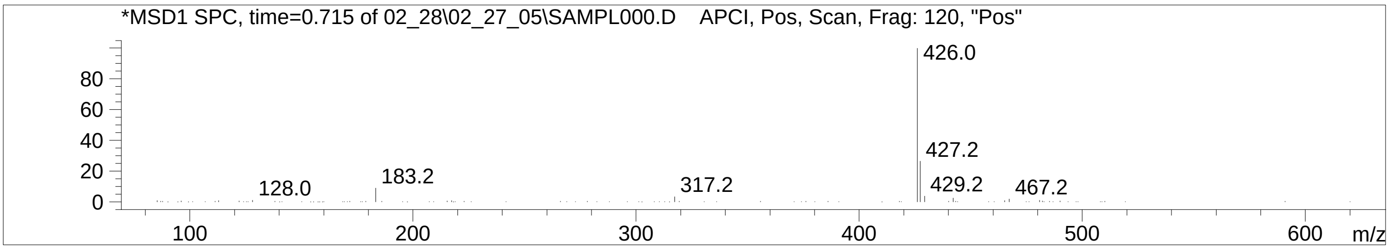

Inj.Date 27.02.2006   T   P2-A-01   Acq. Method SB15_3PN.M
