# Supplementary material for: Selective androgen receptor degrader (SARD) to overcome antiandrogen resistance in castration-resistant prostate cancer
Source: eLife. 2023 Jan 19;12:e70700. doi: 10.7554/eLife.70700 (PMC9901937; doi:10.7554/eLife.70700)
Supplement: Source data 2. [file elife-70700-data2.zip › Supplementary Material_source_data/Figure 8-figure supplement 1 & Supplementary file 1c-source/ZL-3-Z29951011.PDF]

Sample Name: 512197 OK

DFN: D:\DATA\2005\NEW\MARCH\03_15\03_15_06\
SAMPL032.D

MaxPeak: 90.77% Ret_Time: 0.710 min

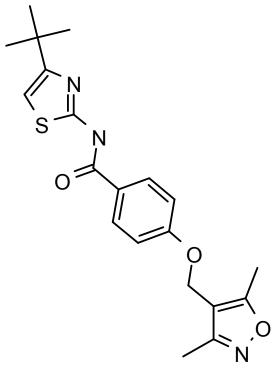

mw = 385,49

The method for the Gradient Sample using short rapid resolution HT Cartridge ZORBAX SB-C18 4.6x15 mm (p/n 821975-932). For testing purity of syntez.

#	Time	Area%
1	0.582	2.45
2	0.697	6.78
3	0.710	90.77

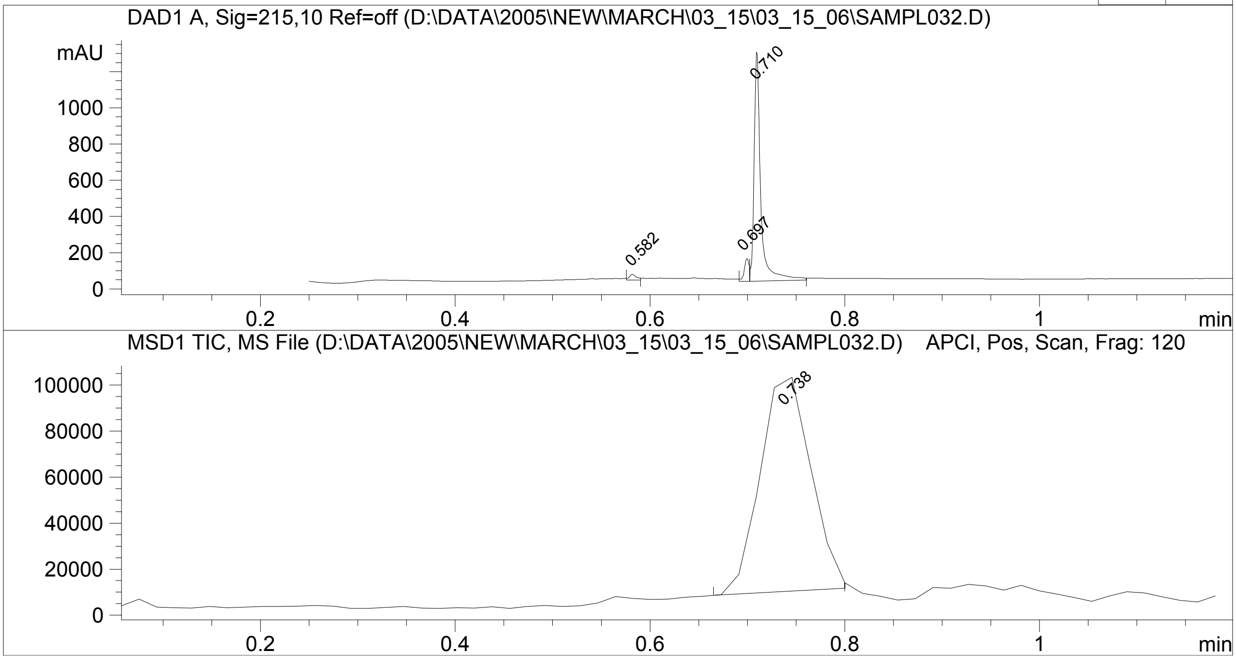

RT 0.738

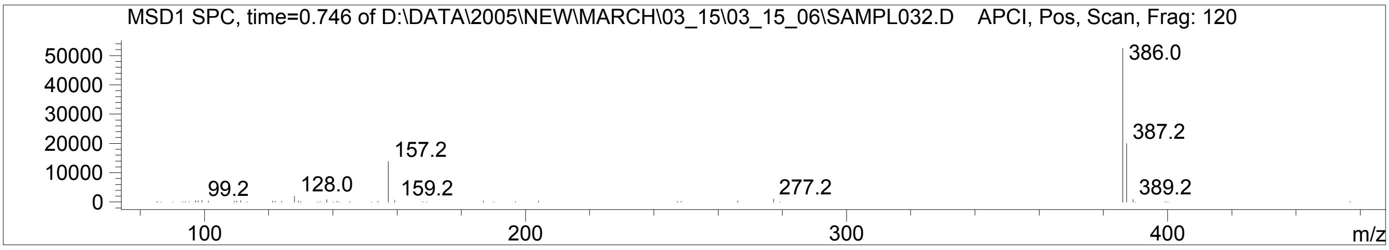

Inj.Date 16.03.2005 O P2-D-07 Acq. Method SB15_3PN.M
