# Supplementary material for: Selective androgen receptor degrader (SARD) to overcome antiandrogen resistance in castration-resistant prostate cancer
Source: eLife. 2023 Jan 19;12:e70700. doi: 10.7554/eLife.70700 (PMC9901937; doi:10.7554/eLife.70700)
Supplement: Source data 2. [file elife-70700-data2.zip › Supplementary Material_source_data/Figure 8-figure supplement 1 & Supplementary file 1c-source/ZL-5-Z26660731.PDF]

Sample Name: 1228156   OK

DFN: D:\DATA\APRIL\04_03\V0403_01\SAMPL021.D

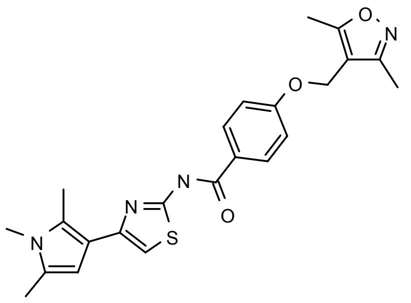

mw = 436,54

MaxPeak: 91.76%   Ret_Time: 0.751 min

The method for the Gradient Sample using short rapid resolution HT Cartridge ZORBAX SB-C18 4.6x15 mm (p/n 821975-932). For testing purity of syntez.

#	Time	Area%
1	0.638	5.77
2	0.751	91.76
3	0.804	2.47

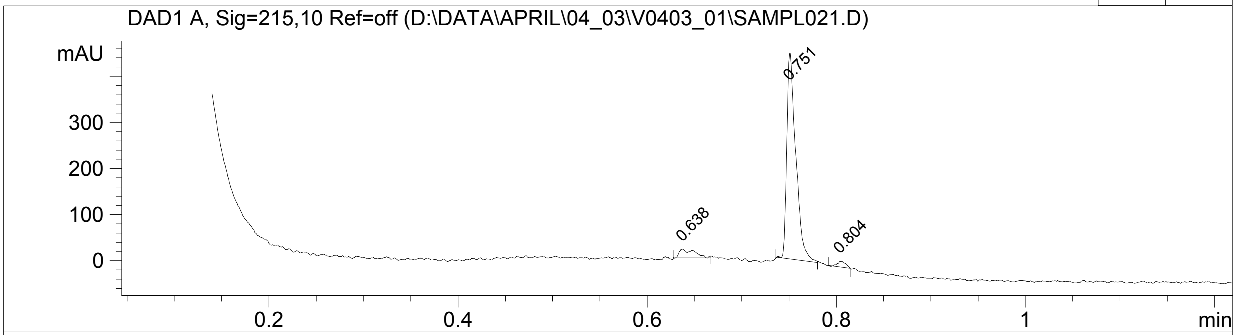

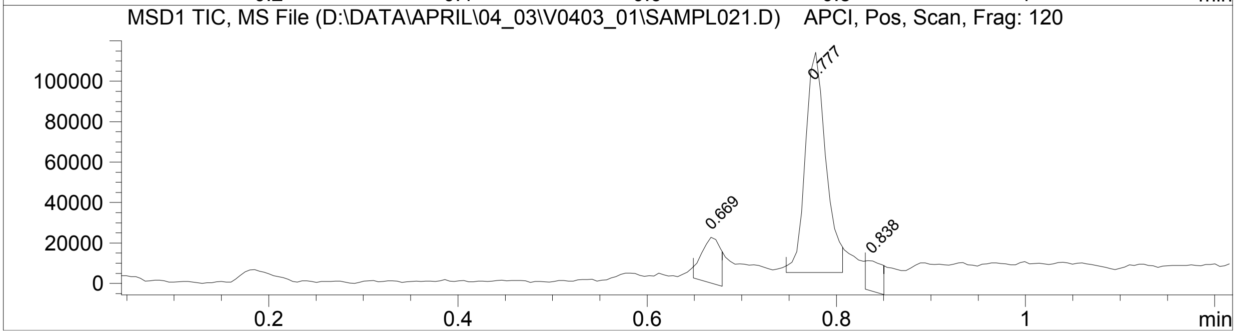

RT 0.669

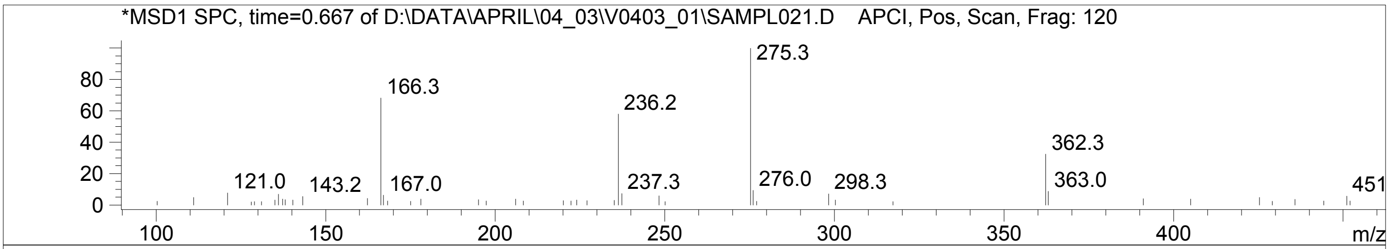

RT 0.777

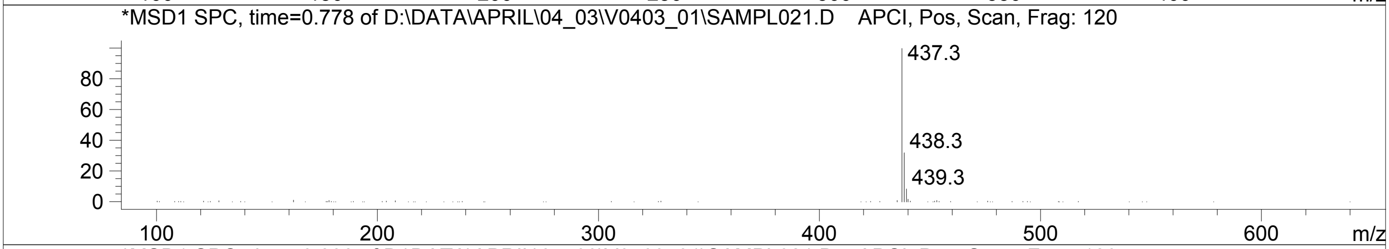

RT 0.838

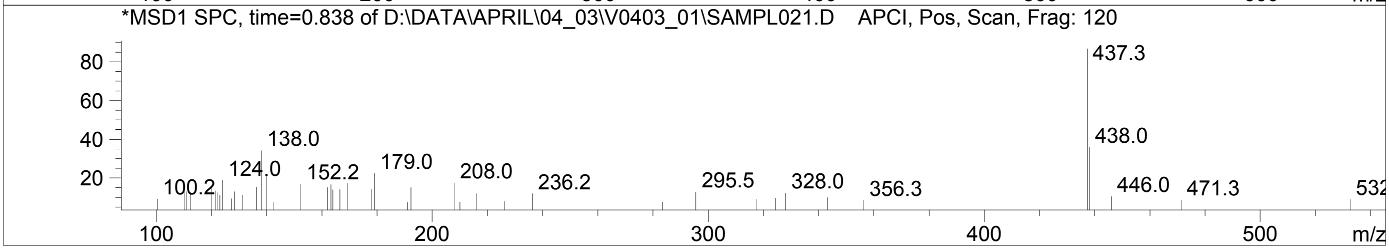

Inj.Date 03.04.2006   L   P2-C-04   Acq. Method SB15TFA3.M
