# Supplementary figures and images for: Selective androgen receptor degrader (SARD) to overcome antiandrogen resistance in castration-resistant prostate cancer (part 1 of 2)
Source: eLife. 2023 Jan 19;12:e70700. doi: 10.7554/eLife.70700 (PMC9901937; doi:10.7554/eLife.70700)

MaxPeak: 98.38%  
Ret\_Time: 0.926 min

2604918

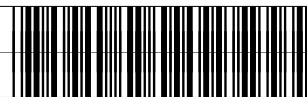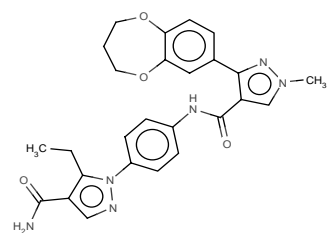

Mol Wt 486.523  
Exact Mass 486.22

| # | Time  | Area% |
|---|-------|-------|
| 1 | 0.926 | 98.38 |
| 2 | 1.004 | 1.62  |

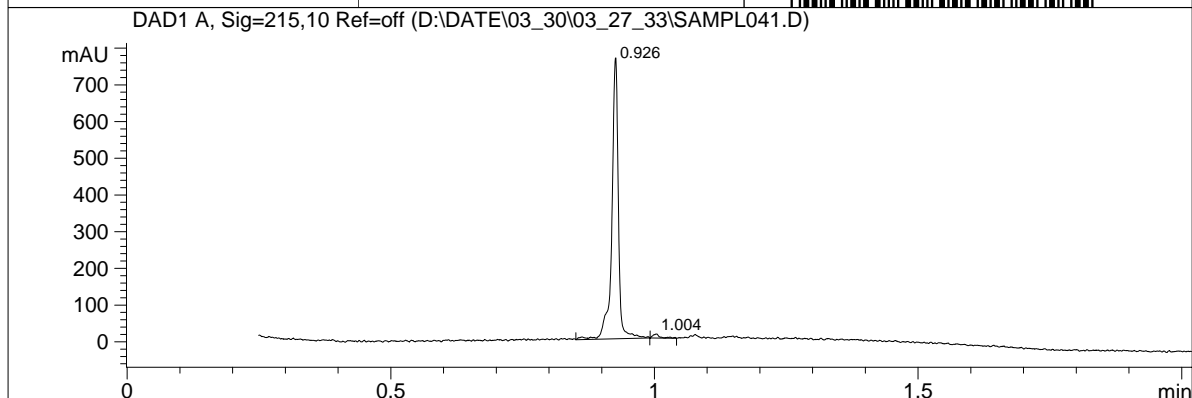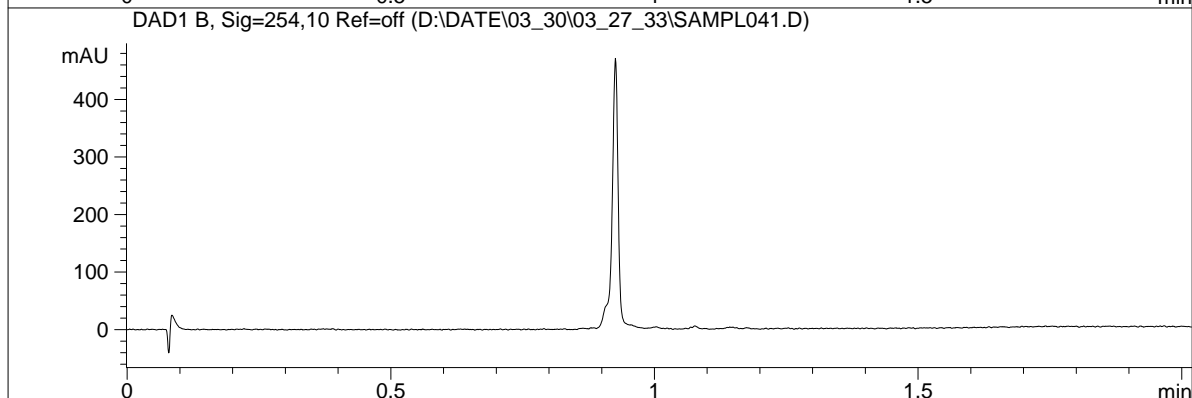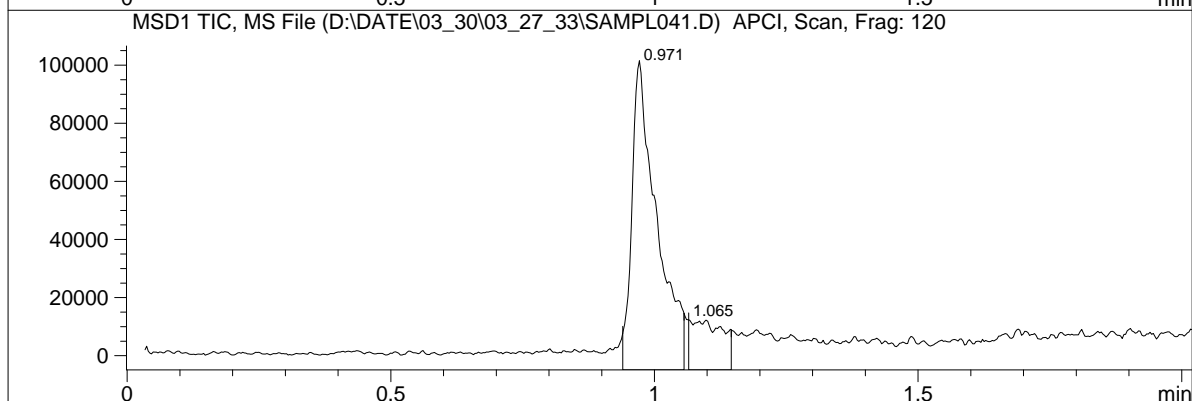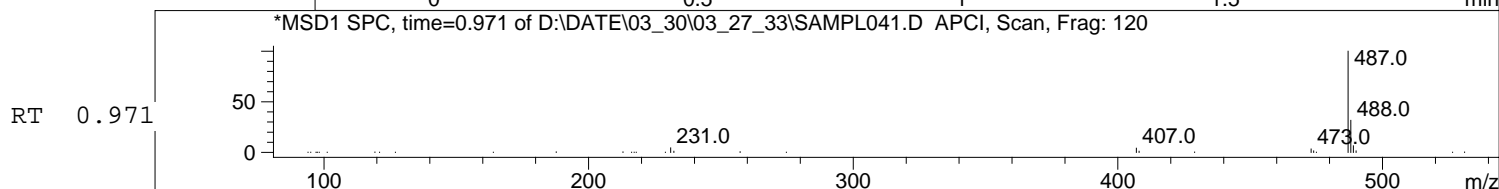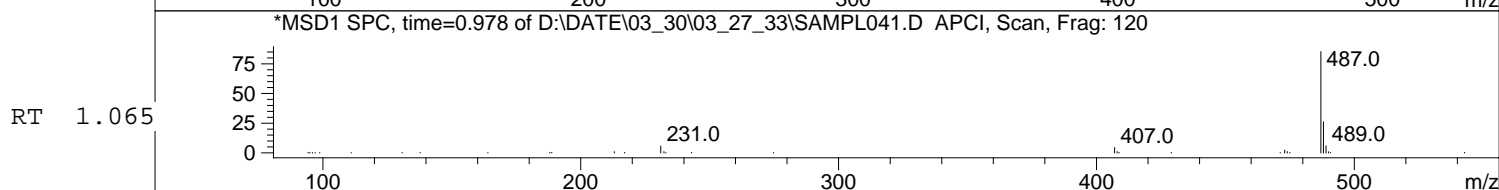

Supplement: Source data 2. [file elife-70700-data2.zip › Supplementary Material_source_data/Figure 1-figure supplement 1 & Supplementary1a-source/Z16.PDF]

MaxPeak: 100.00%  
Ret\_Time: 0.980 min

2681079

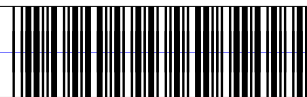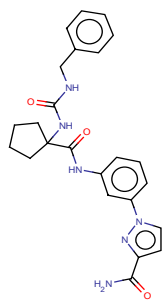

Mol Wt 446.502  
Exact Mass 446.23

| # | Time  | Area%  |
|---|-------|--------|
| 1 | 0.980 | 100.00 |

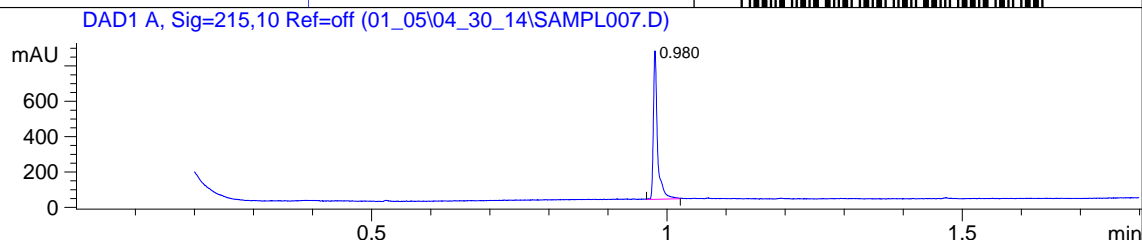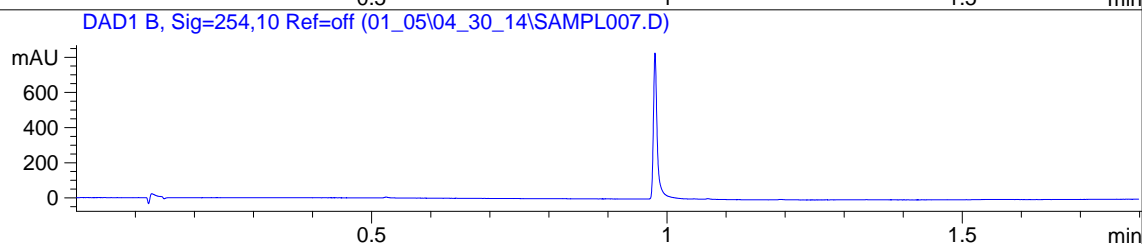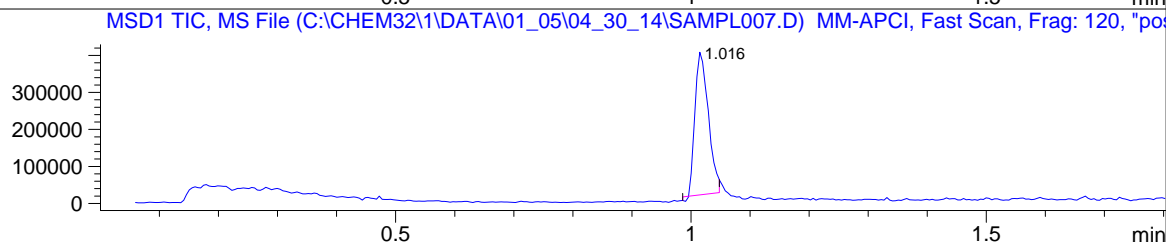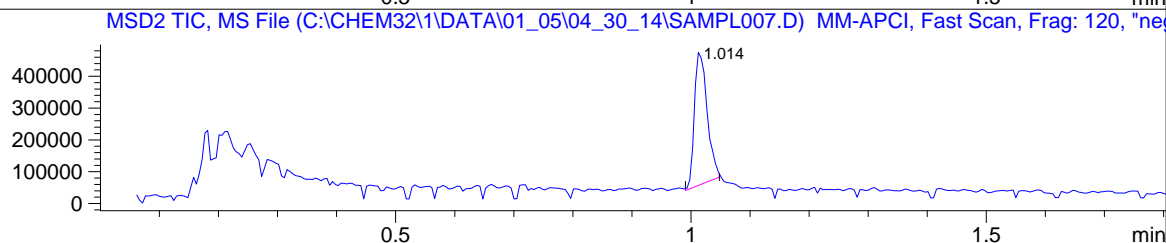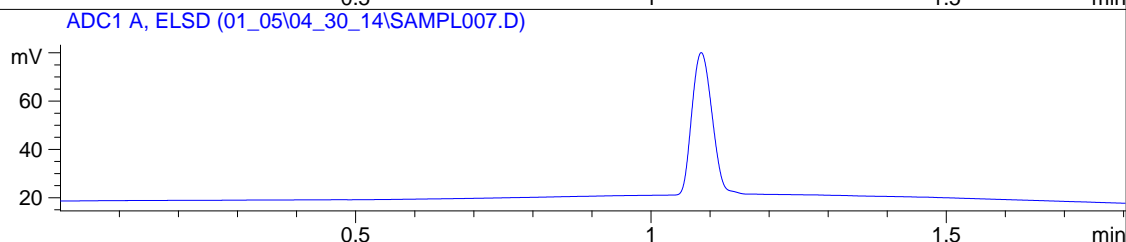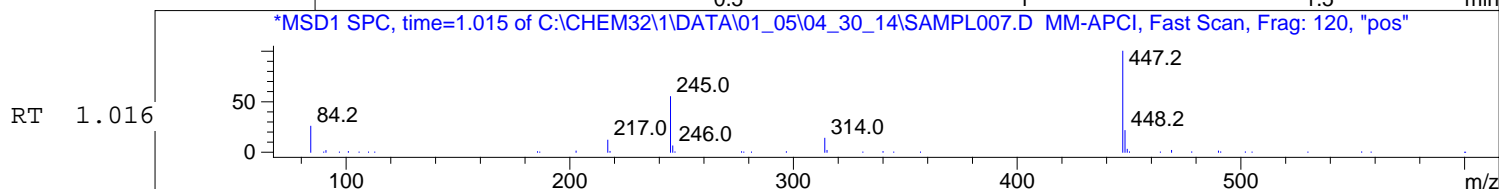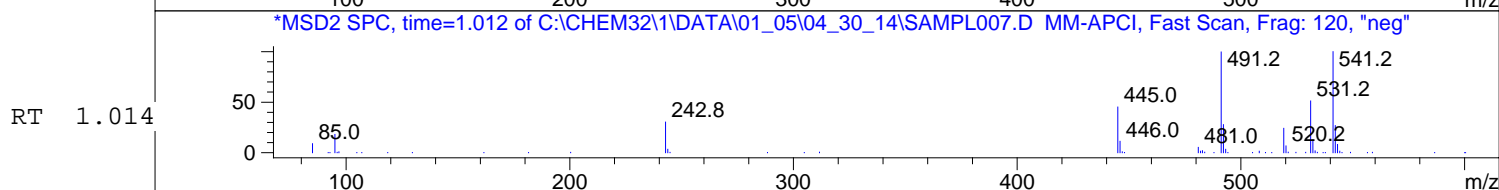

Supplement: Source data 2. [file elife-70700-data2.zip › Supplementary Material_source_data/Figure 1-figure supplement 1 & Supplementary1a-source/Z17.PDF]

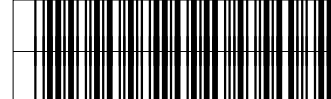

MaxPeak: 100.00%  
Ret\_Time: 0.607 min

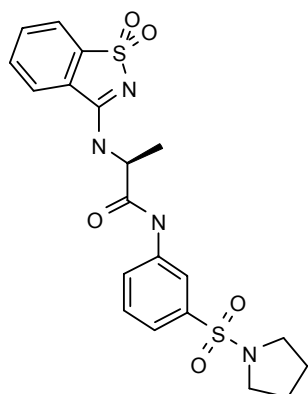

mw = 462,55

| # | Time  | Area%  |
|---|-------|--------|
| 1 | 0.607 | 100.00 |

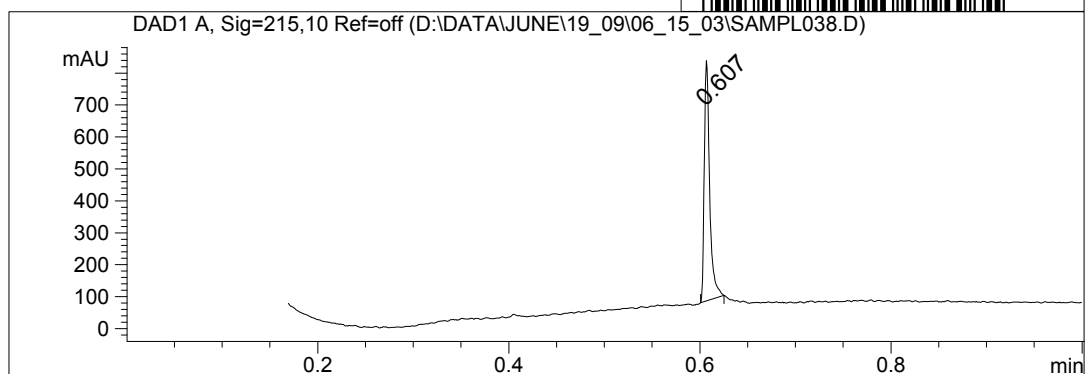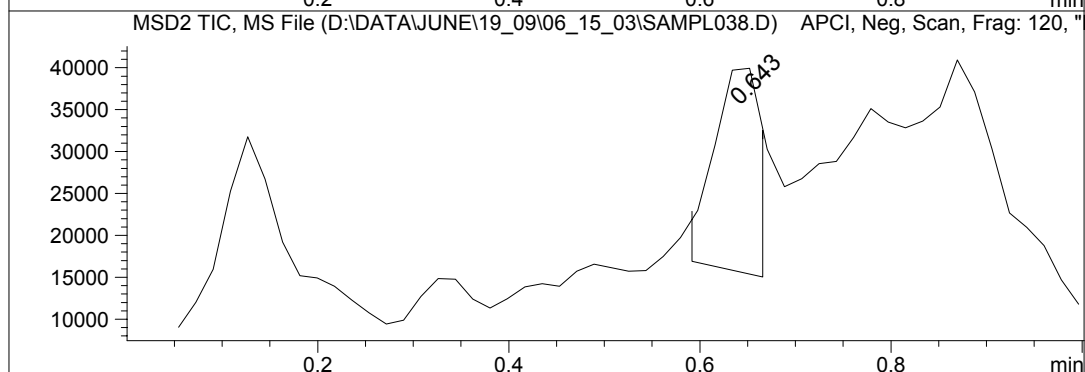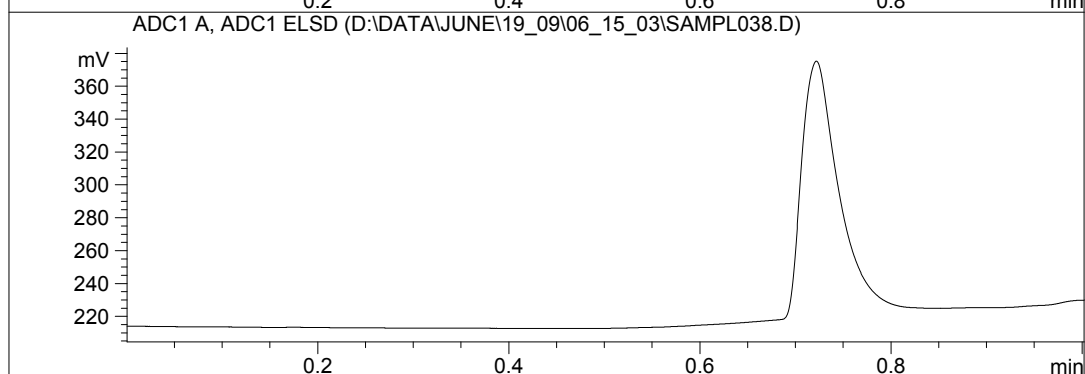

RT 0.643

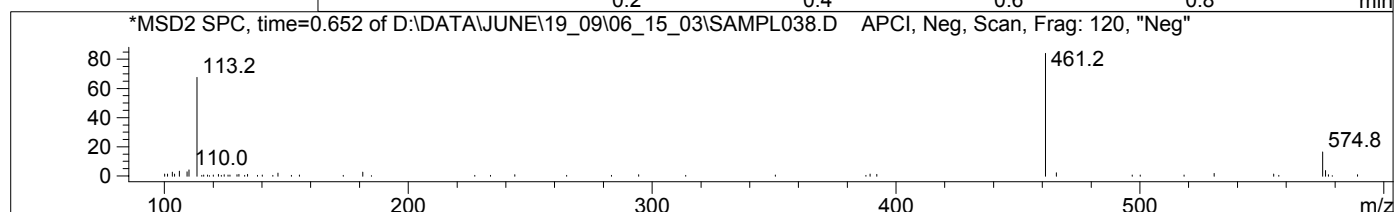

Supplement: Source data 2. [file elife-70700-data2.zip › Supplementary Material_source_data/Figure 1-figure supplement 1 & Supplementary1a-source/Z2.PDF]

MaxPeak: 91.15%  
Ret\_Time: 1.176 min

5355026

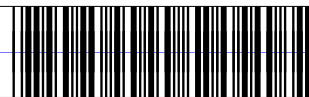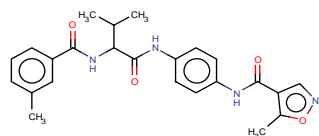

Mol Wt 434.488  
Exact Mass 434.22

| # | Time  | Area% |
|---|-------|-------|
| 1 | 0.742 | 3.83  |
| 2 | 1.039 | 5.02  |
| 3 | 1.176 | 91.15 |

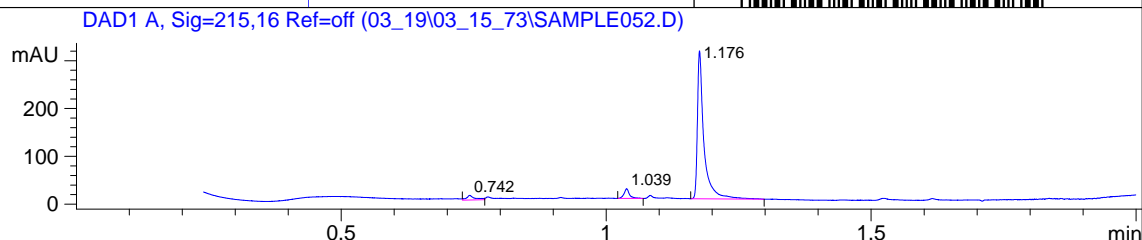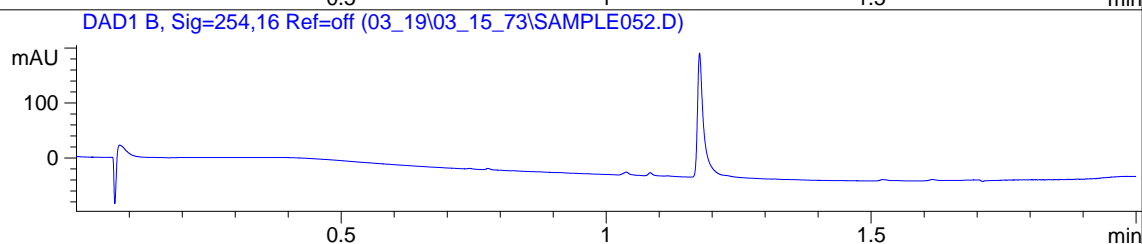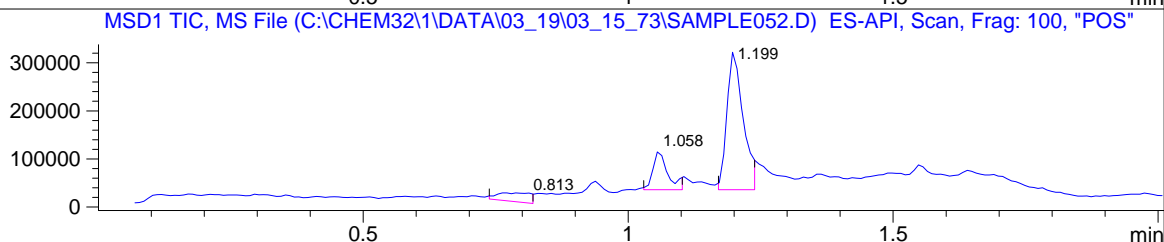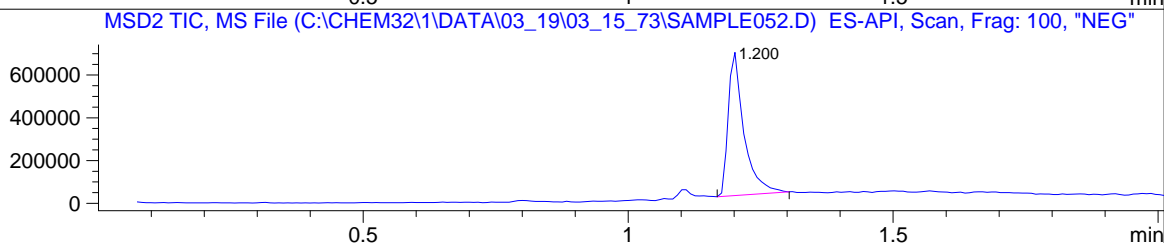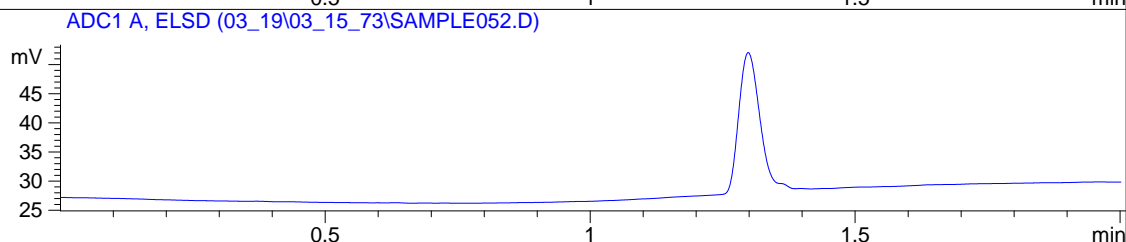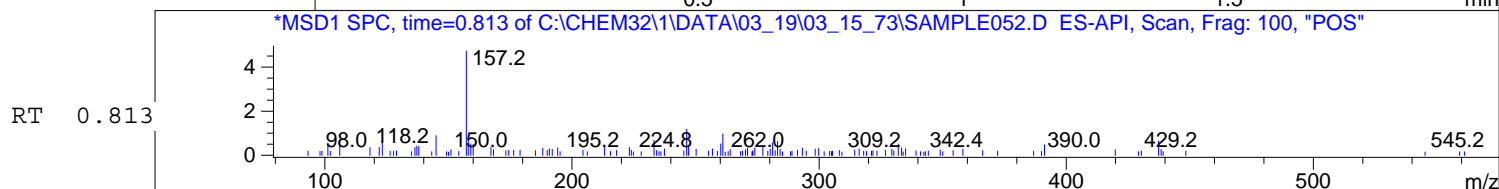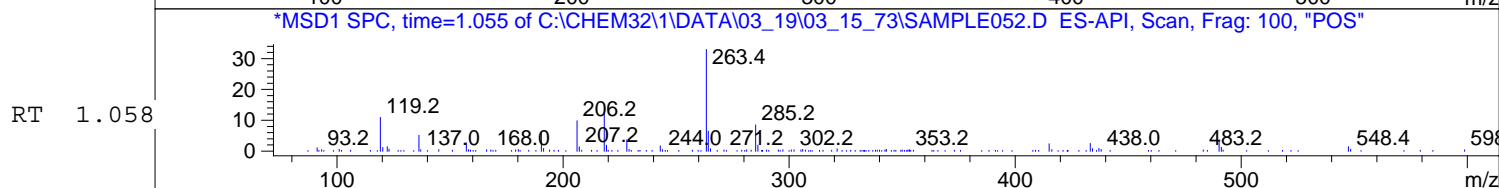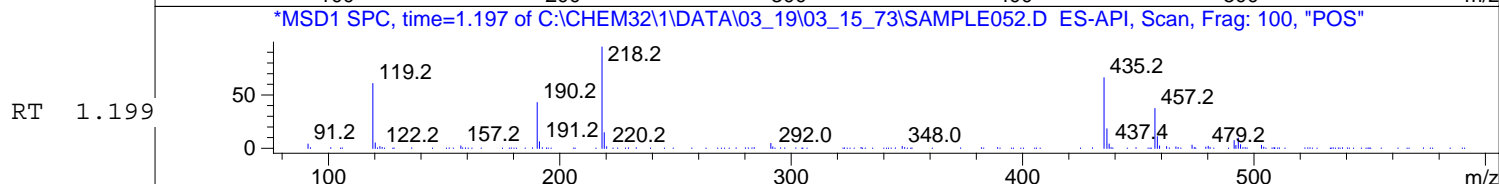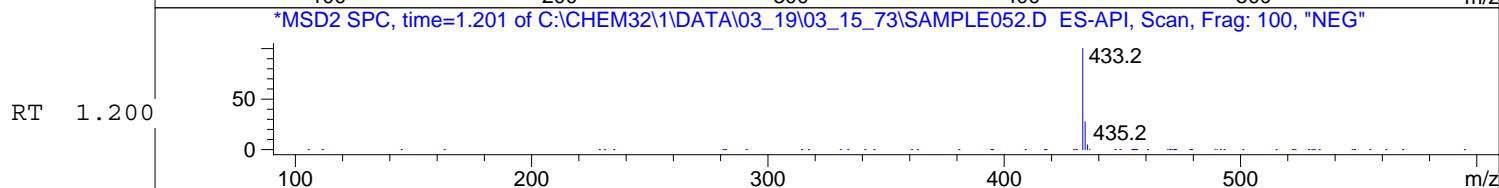

Supplement: Source data 2. [file elife-70700-data2.zip › Supplementary Material_source_data/Figure 1-figure supplement 1 & Supplementary1a-source/Z20.PDF]

MaxPeak: 94.58%  
Ret\_Time: 1.134 min

3083495

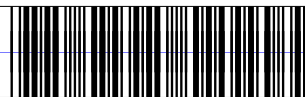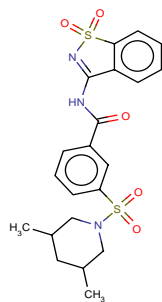

Mol Wt 461.554  
Exact Mass 461.12

| # | Time  | Area% |
|---|-------|-------|
| 1 | 0.303 | 2.67  |
| 2 | 1.053 | 2.74  |
| 3 | 1.134 | 94.58 |

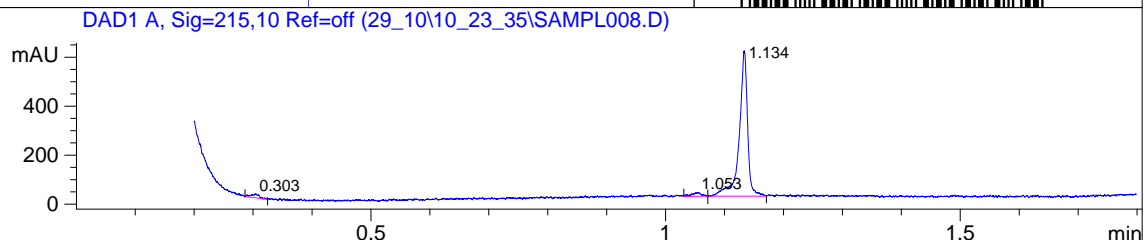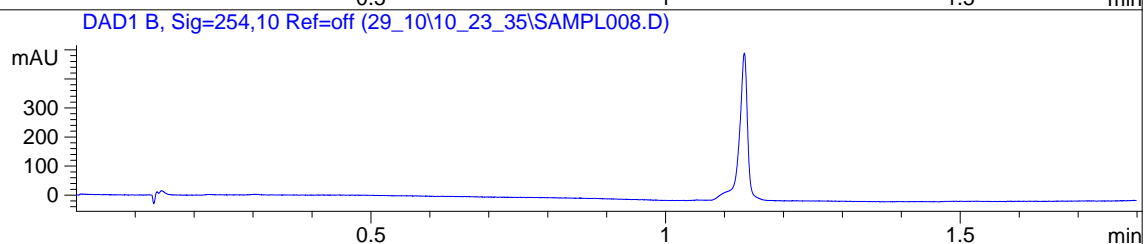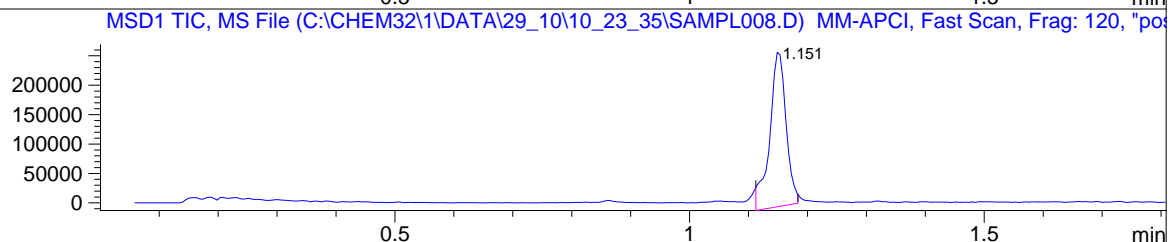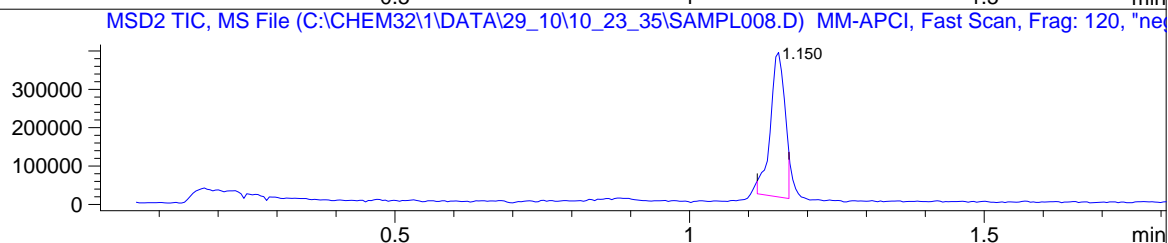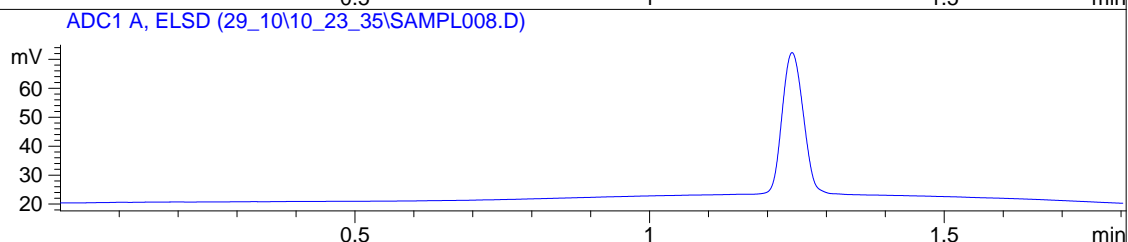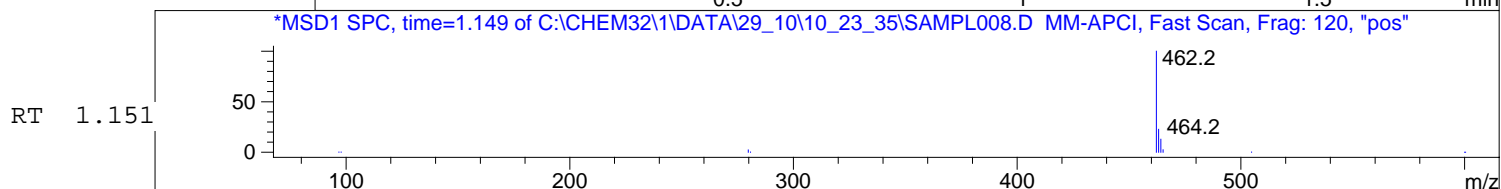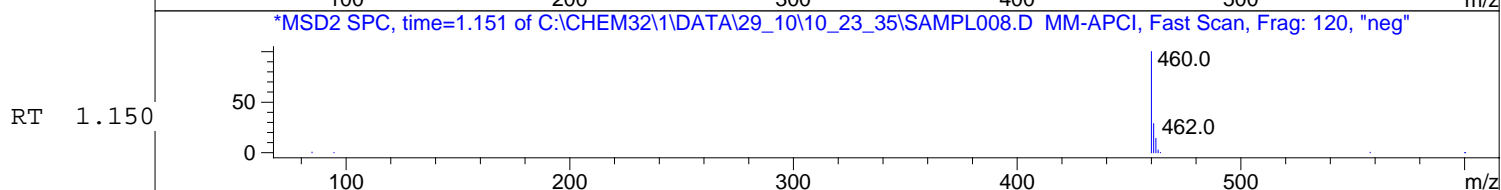

Supplement: Source data 2. [file elife-70700-data2.zip › Supplementary Material_source_data/Figure 1-figure supplement 1 & Supplementary1a-source/Z22.PDF]

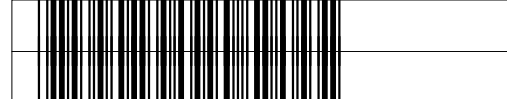

MaxPeak: 100.00%  
Ret\_Time: 0.698 min

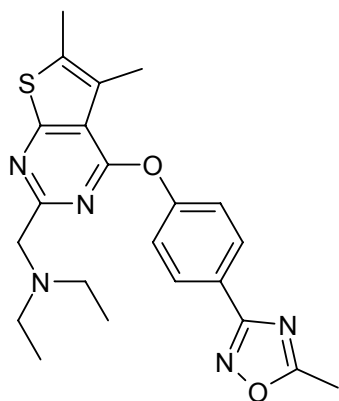

mw = 423,54

| # | Time  | Area%  |
|---|-------|--------|
| 1 | 0.698 | 100.00 |

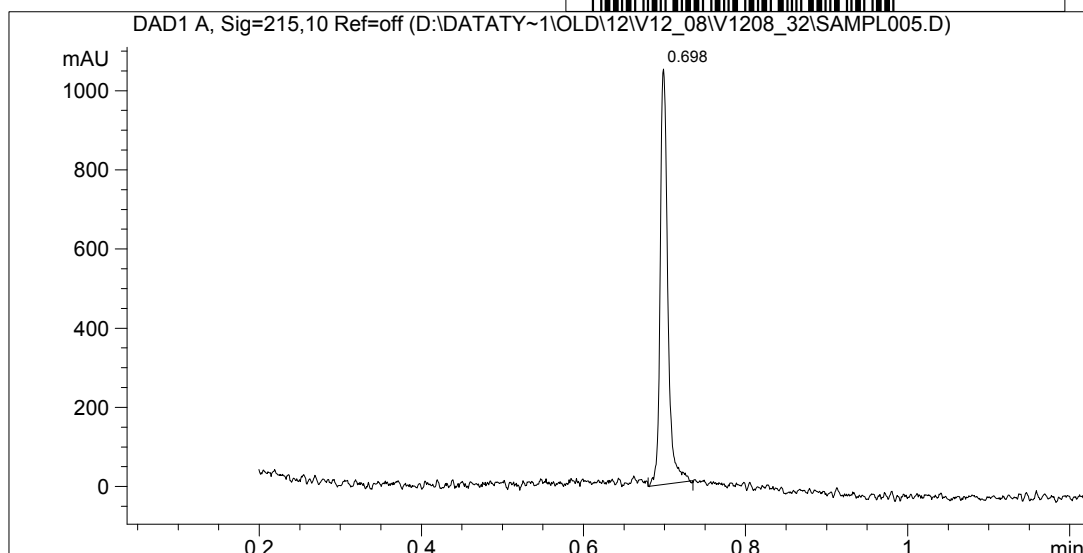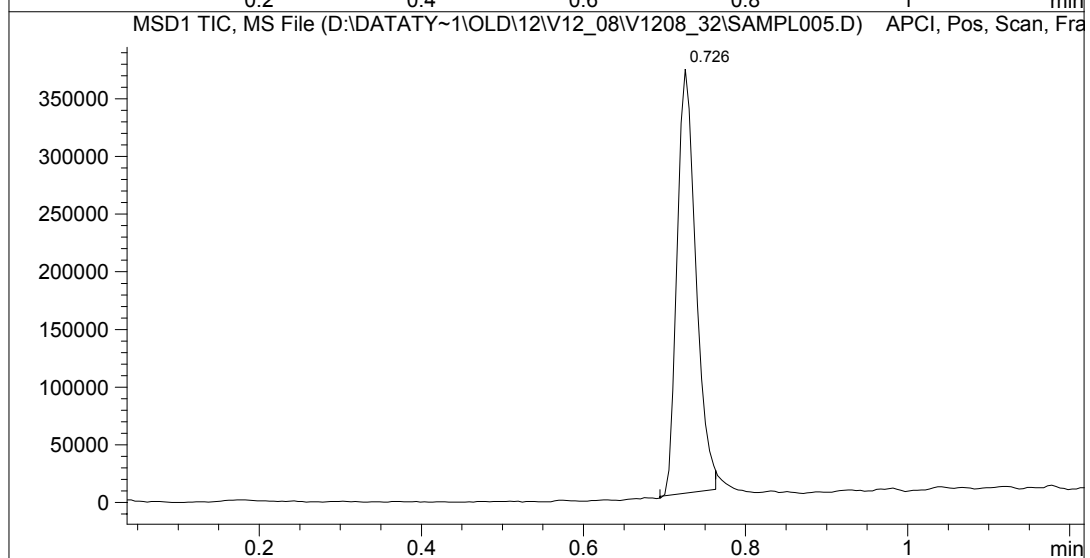

RT 0.726

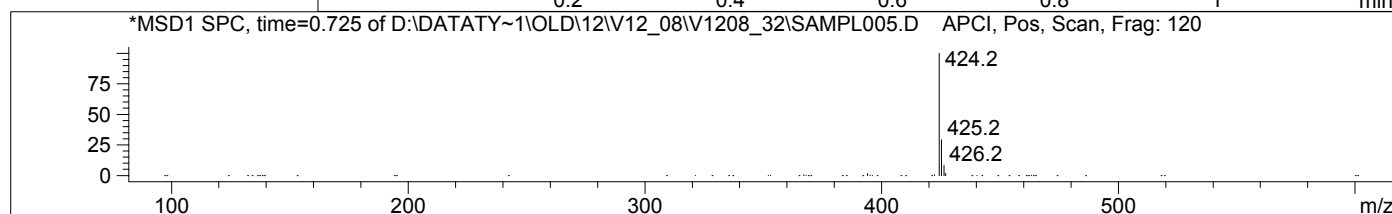

Supplement: Source data 2. [file elife-70700-data2.zip › Supplementary Material_source_data/Figure 1-figure supplement 1 & Supplementary1a-source/Z26.PDF]

Sample Name: 1432396

MaxPeak: 100.00%  
Ret\_Time: 0.760 min

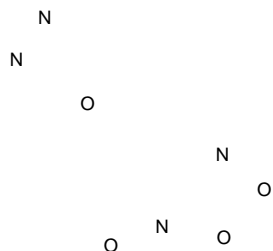

| # | Time  | Area%  |
|---|-------|--------|
| 1 | 0.760 | 100.00 |

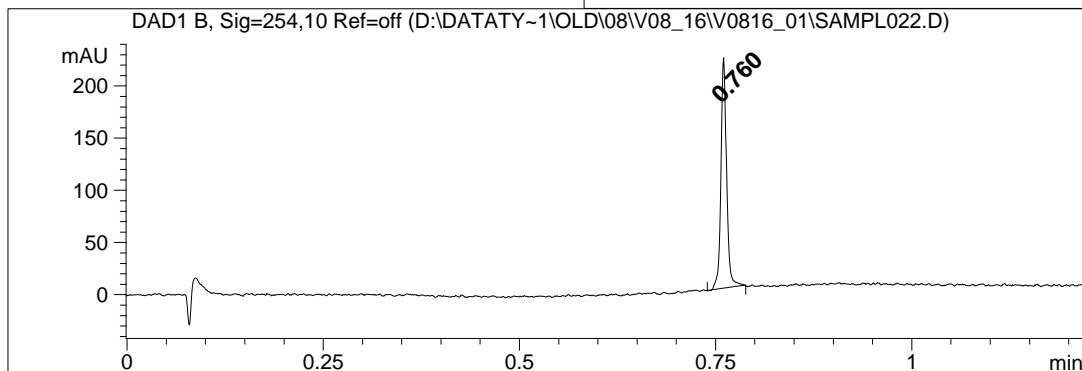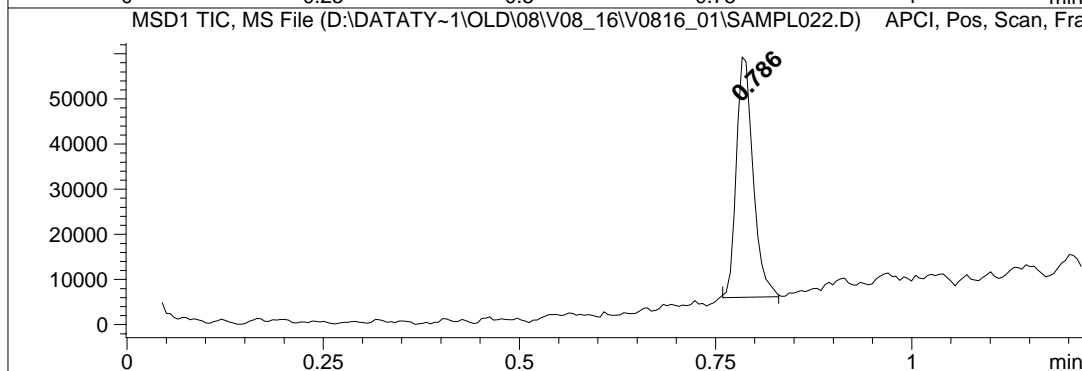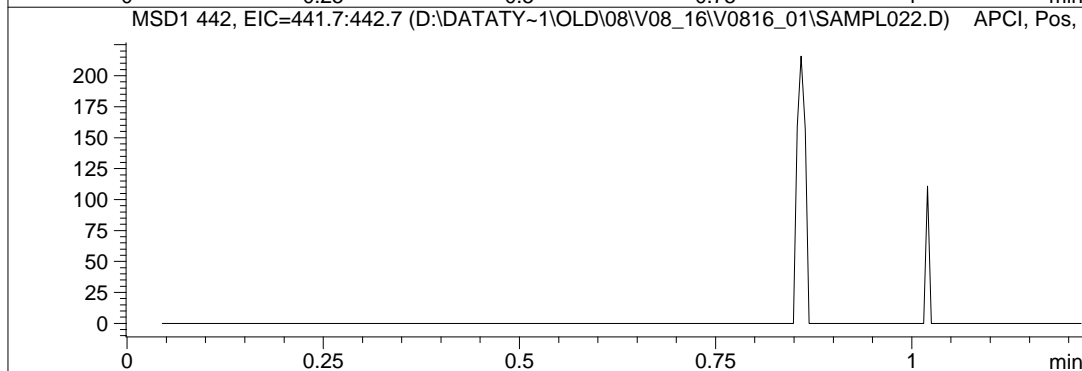

RT 0.786

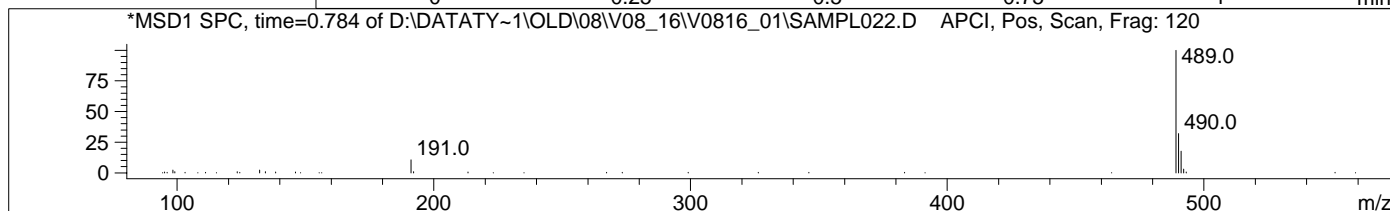

Supplement: Source data 2. [file elife-70700-data2.zip › Supplementary Material_source_data/Figure 1-figure supplement 1 & Supplementary1a-source/Z27.PDF]

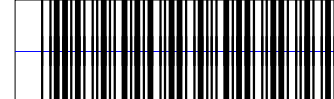

MaxPeak: 92.71%  
Ret\_Time: 0.673 min

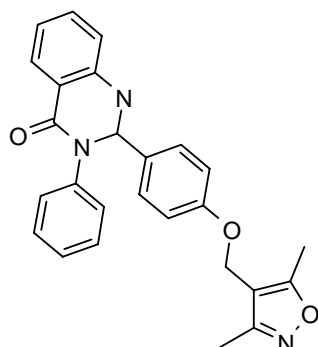

mw = 425,49

| # | Time  | Area% |
|---|-------|-------|
| 1 | 0.619 | 4.45  |
| 2 | 0.673 | 92.71 |
| 3 | 0.698 | 2.84  |

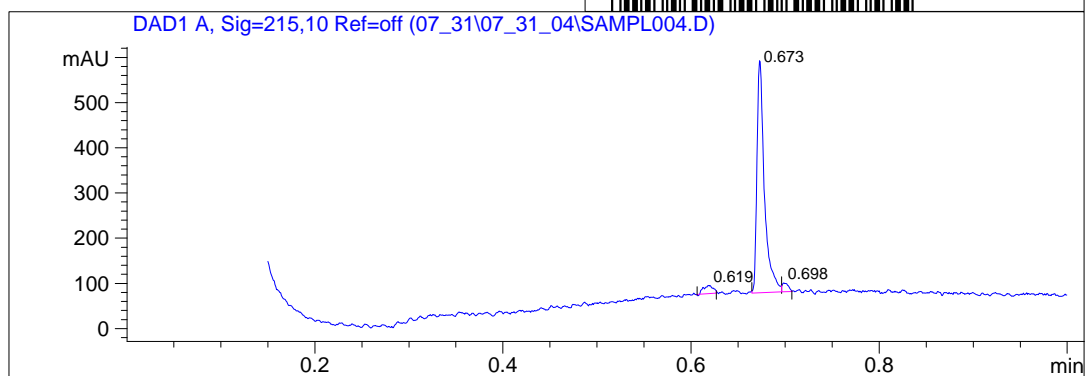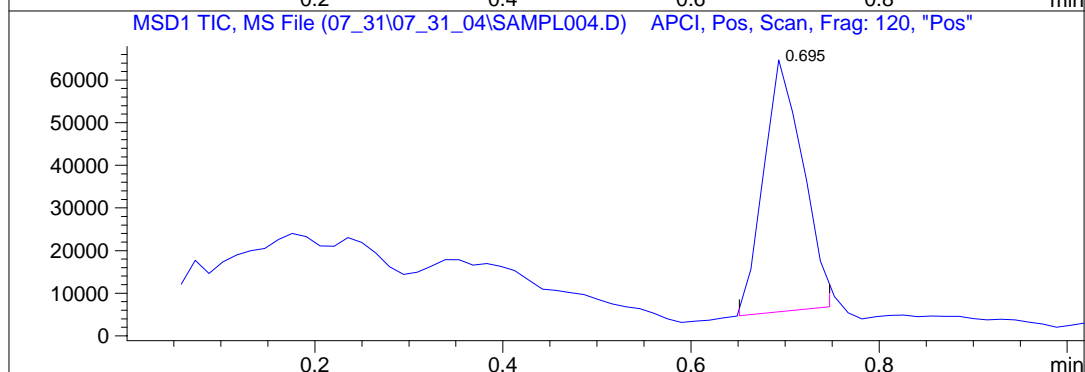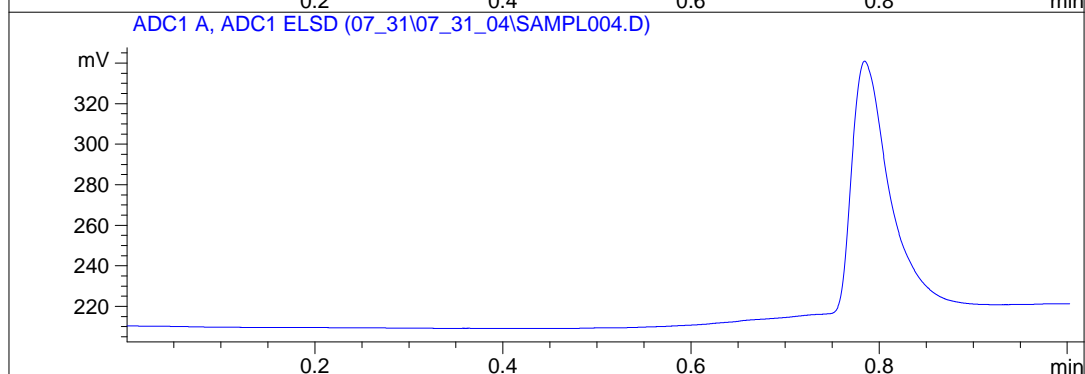

RT 0.695

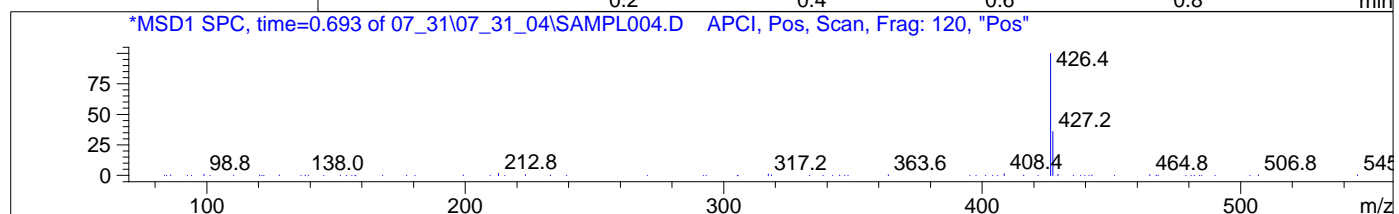

Supplement: Source data 2. [file elife-70700-data2.zip › Supplementary Material_source_data/Figure 1-figure supplement 1 & Supplementary1a-source/Z29.PDF]

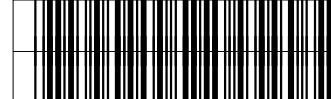

MaxPeak: 93.74%  
Ret\_Time: 0.757 min

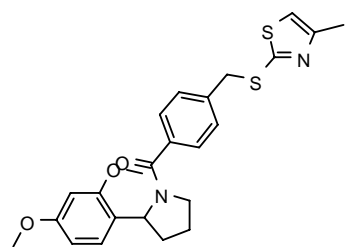

mw = 454,61

| # | Time  | Area% |
|---|-------|-------|
| 1 | 0.656 | 6.26  |
| 2 | 0.757 | 93.74 |

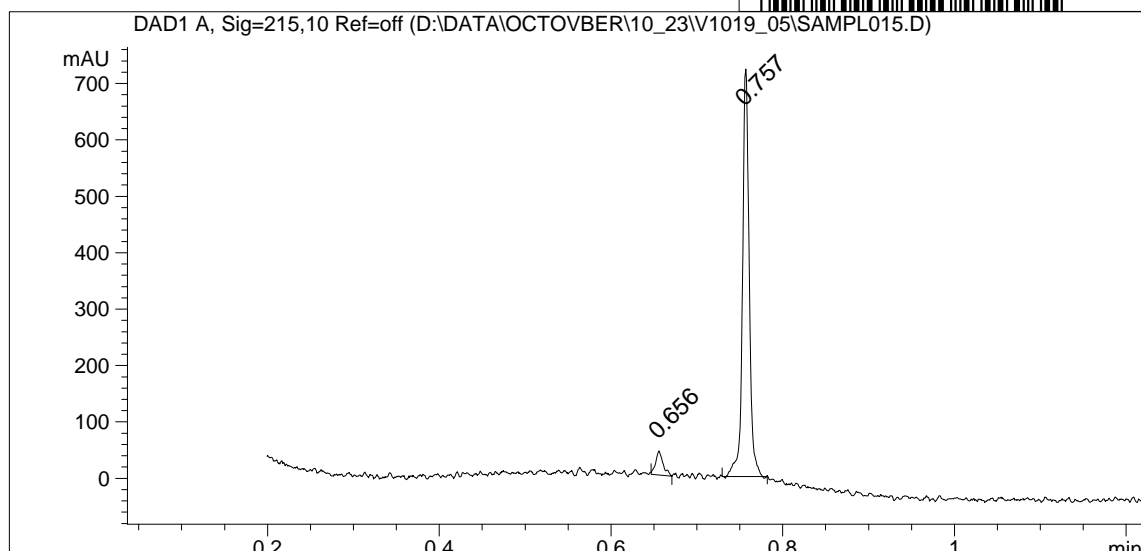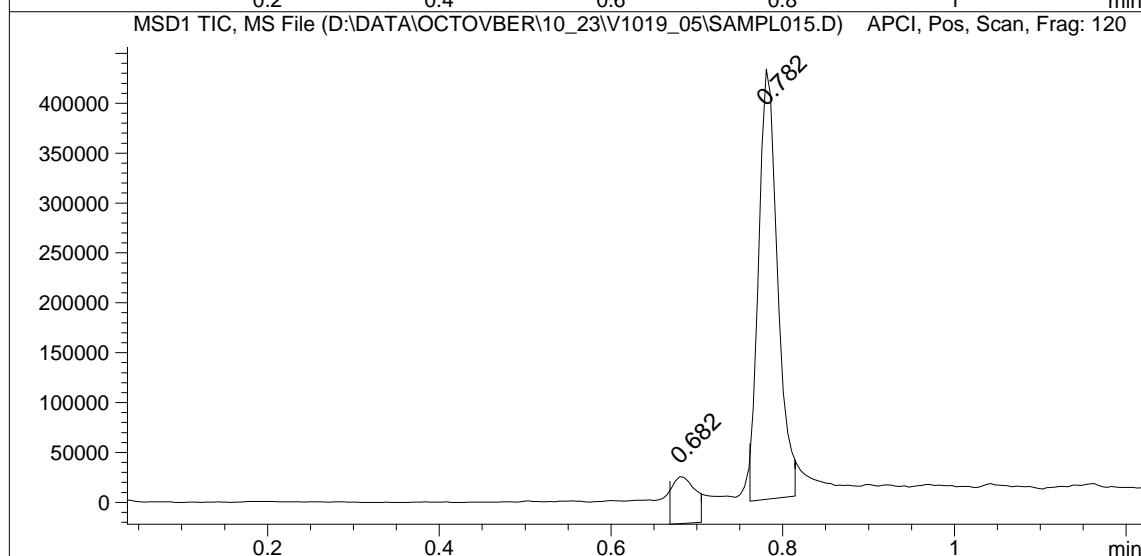

RT 0.682

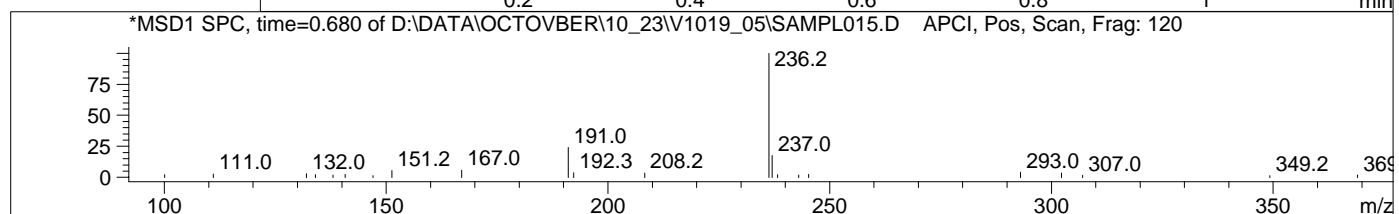

RT 0.782

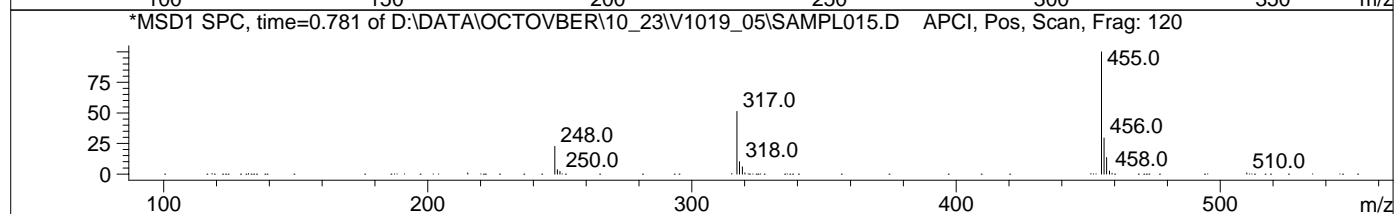

Supplement: Source data 2. [file elife-70700-data2.zip › Supplementary Material_source_data/Figure 1-figure supplement 1 & Supplementary1a-source/Z30.PDF]

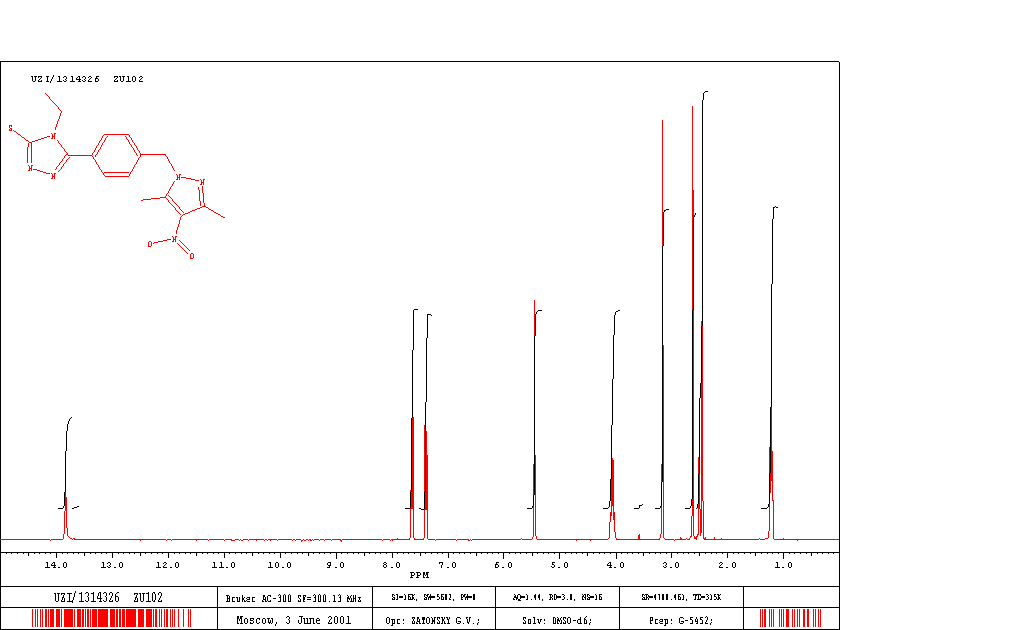

Supplement: Source data 2. [file elife-70700-data2.zip › Supplementary Material_source_data/Figure 1-figure supplement 1 & Supplementary1a-source/Z33.TIF]

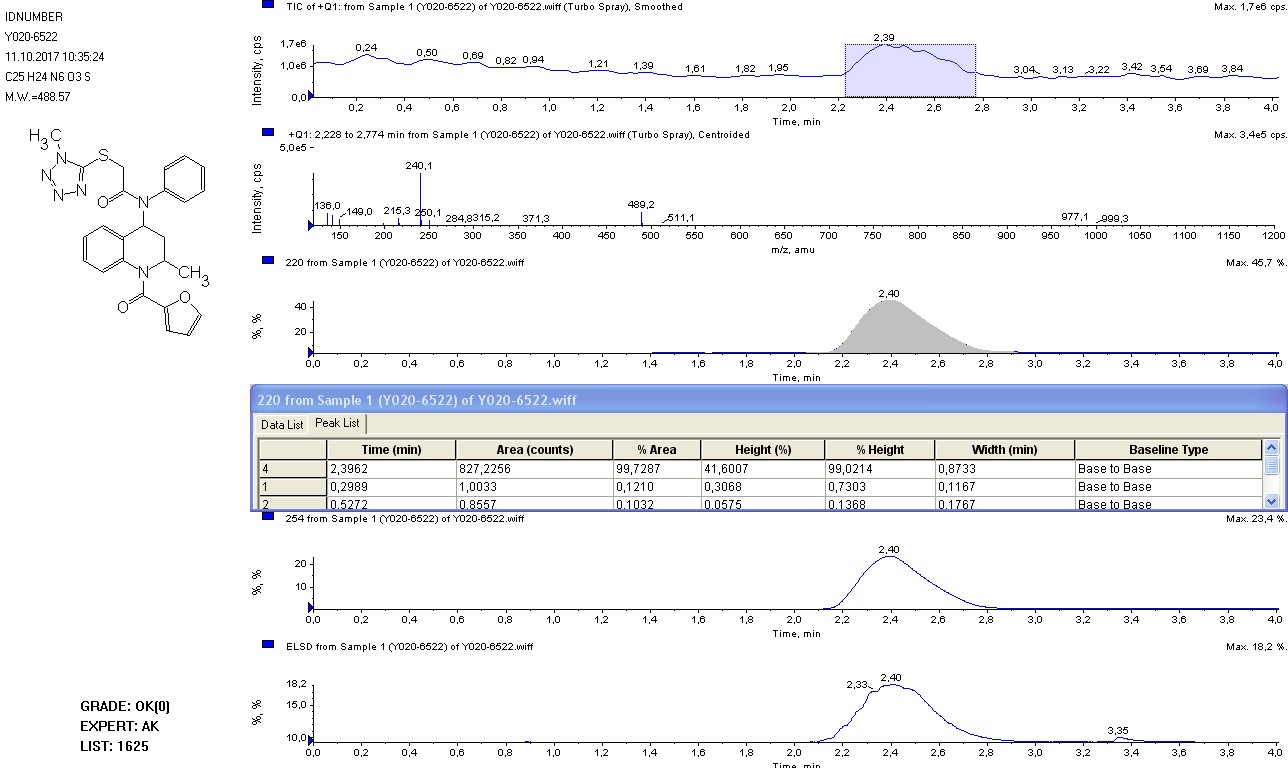

Supplement: Source data 2. [file elife-70700-data2.zip › Supplementary Material_source_data/Figure 1-figure supplement 1 & Supplementary1a-source/Z39.JPG]

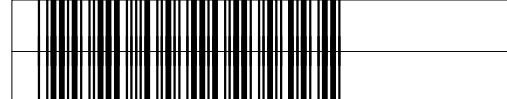

MaxPeak: 94.23%  
Ret\_Time: 0.682 min

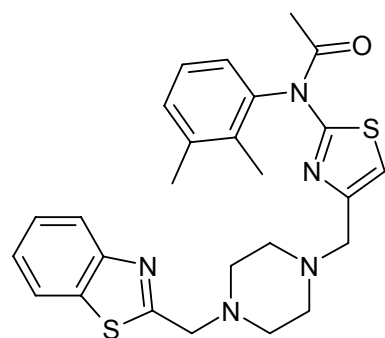

mw = 491,68

| # | Time  | Area% |
|---|-------|-------|
| 1 | 0.682 | 94.23 |
| 2 | 0.769 | 5.77  |

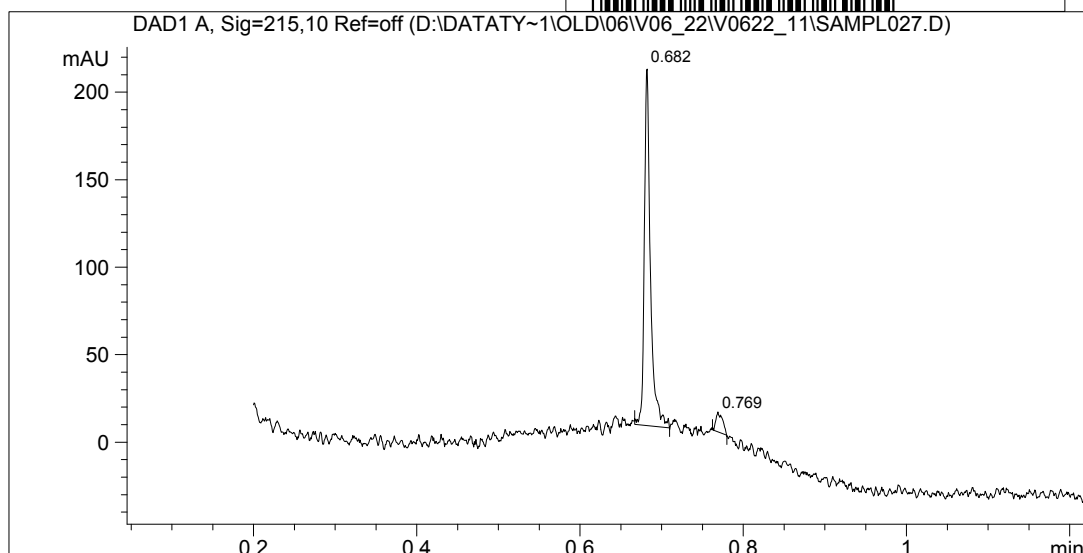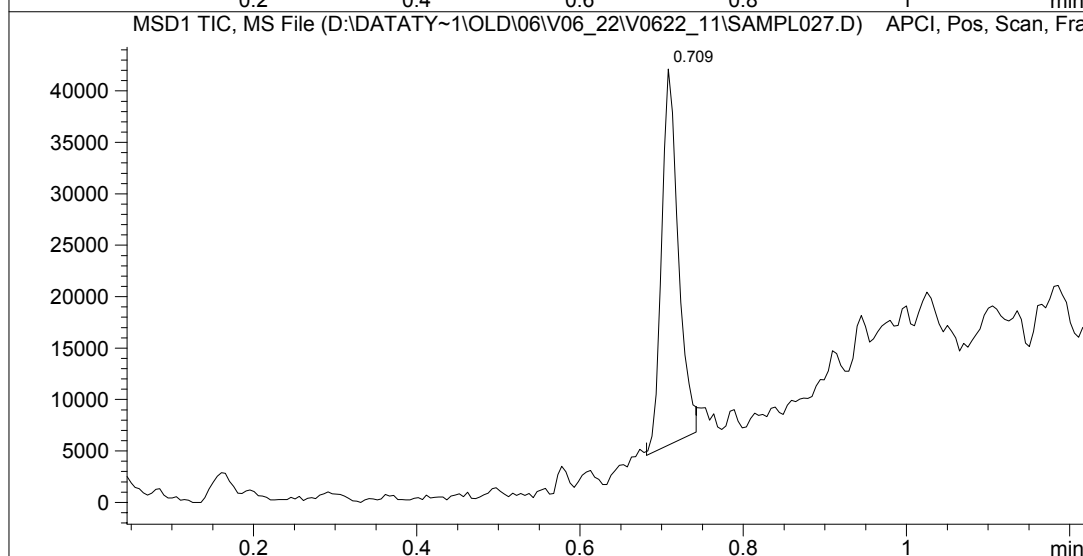

RT 0.709

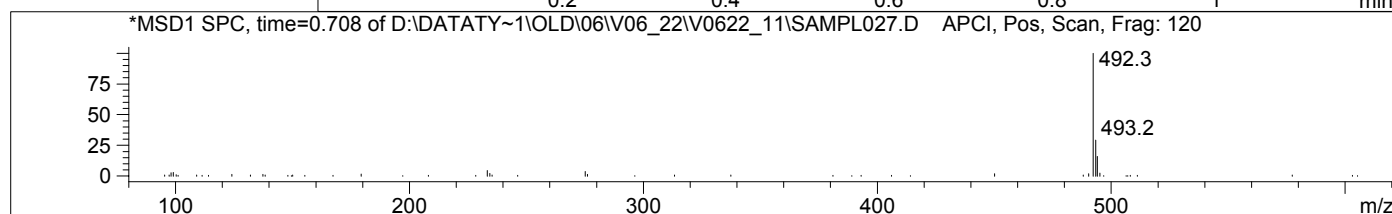

Supplement: Source data 2. [file elife-70700-data2.zip › Supplementary Material_source_data/Figure 1-figure supplement 1 & Supplementary1a-source/Z4.PDF]

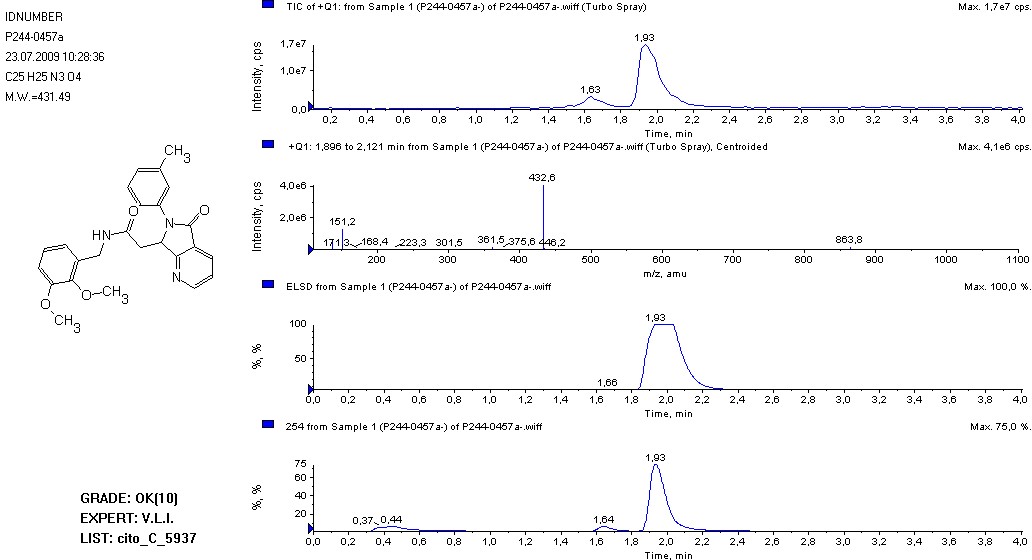

Supplement: Source data 2. [file elife-70700-data2.zip › Supplementary Material_source_data/Figure 1-figure supplement 1 & Supplementary1a-source/Z44.JPG]

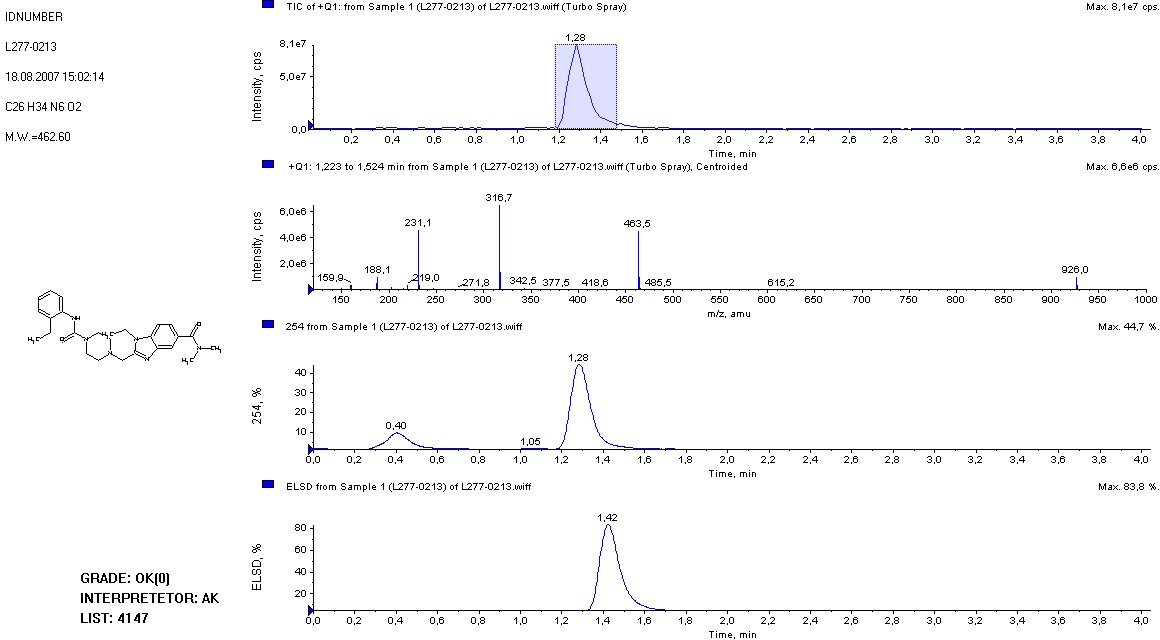

Supplement: Source data 2. [file elife-70700-data2.zip › Supplementary Material_source_data/Figure 1-figure supplement 1 & Supplementary1a-source/Z45-2.JPG]

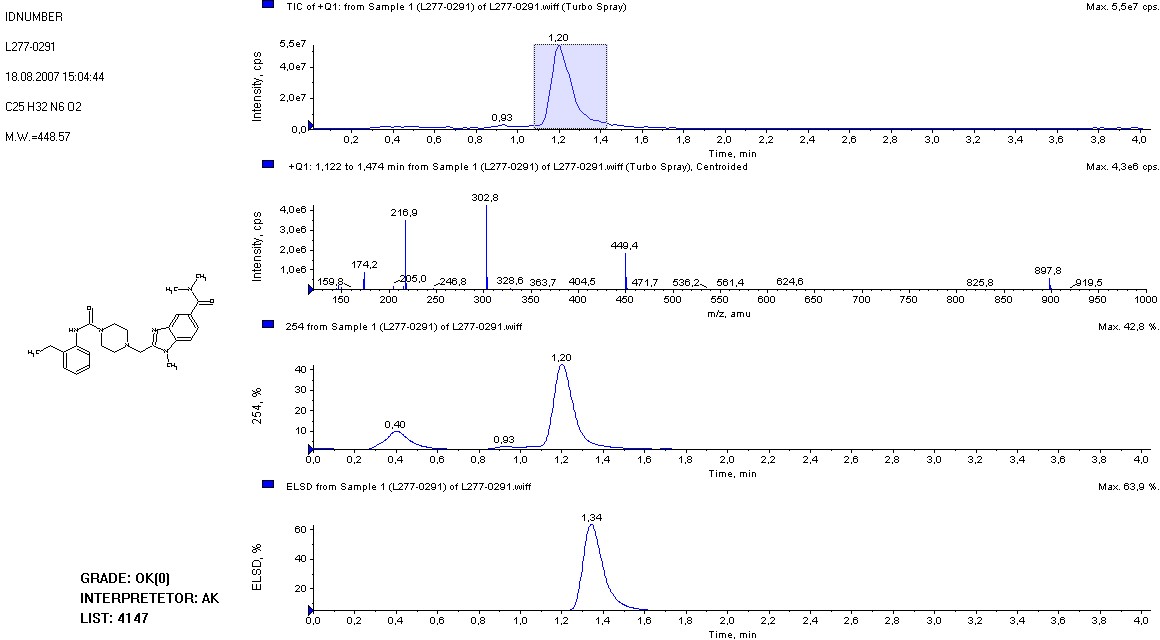

Supplement: Source data 2. [file elife-70700-data2.zip › Supplementary Material_source_data/Figure 1-figure supplement 1 & Supplementary1a-source/Z46-2.JPG]

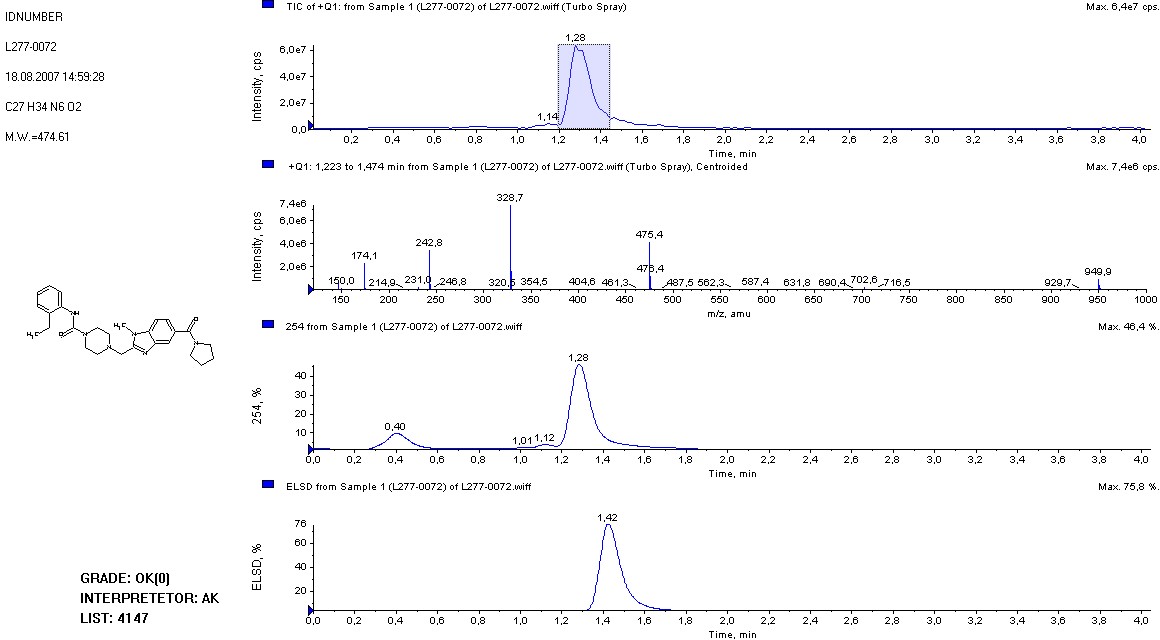

Supplement: Source data 2. [file elife-70700-data2.zip › Supplementary Material_source_data/Figure 1-figure supplement 1 & Supplementary1a-source/Z49-2.JPG]

MaxPeak: 92.22%  
Ret\_Time: 1.193 min

2467071

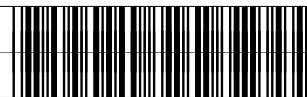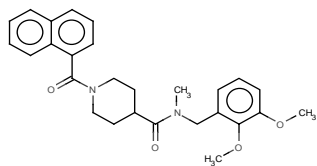

Mol Wt 446.538  
Exact Mass 446.26

| # | Time  | Area% |
|---|-------|-------|
| 1 | 0.849 | 1.26  |
| 2 | 0.894 | 1.31  |
| 3 | 1.156 | 1.60  |
| 4 | 1.193 | 92.22 |
| 5 | 1.215 | 2.08  |
| 6 | 1.250 | 1.54  |

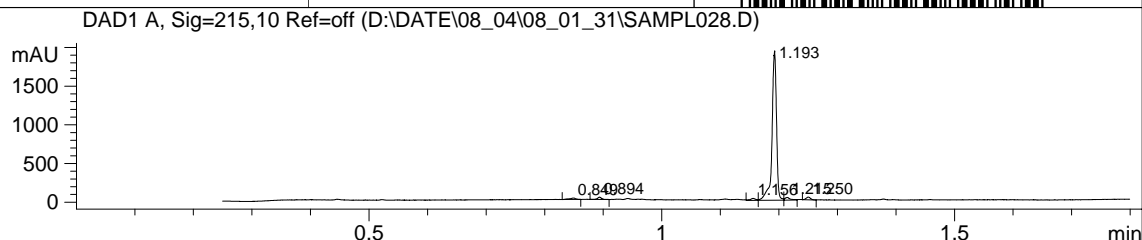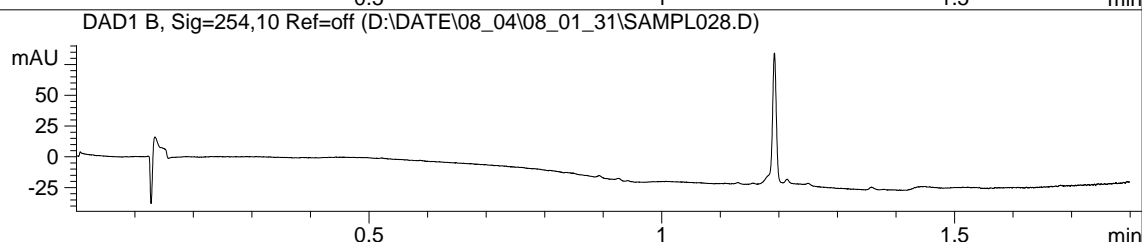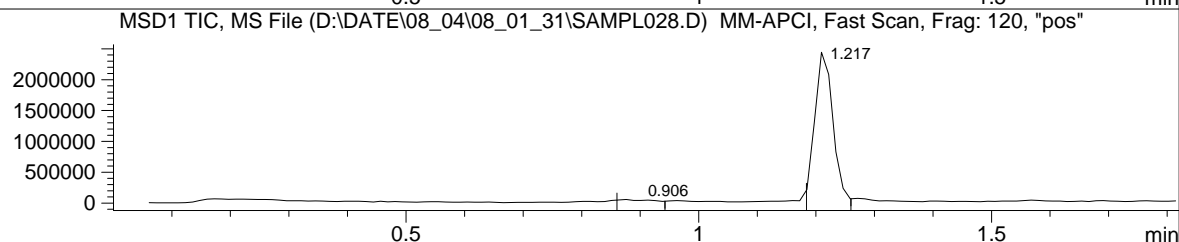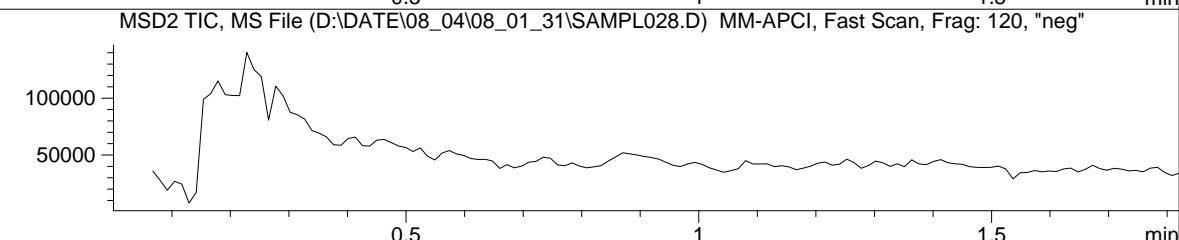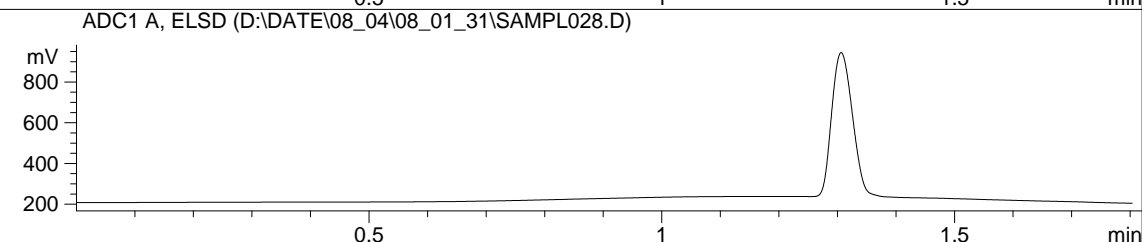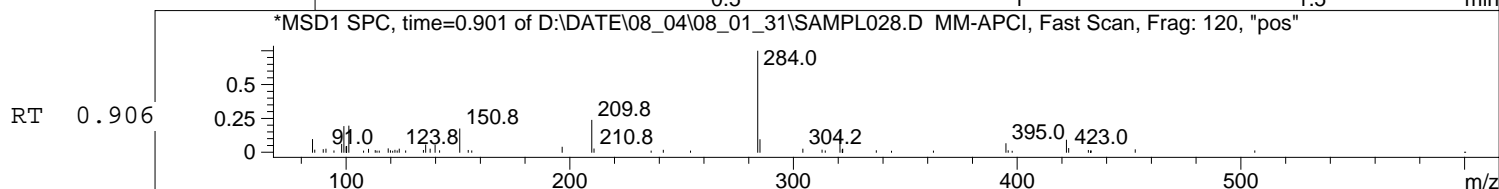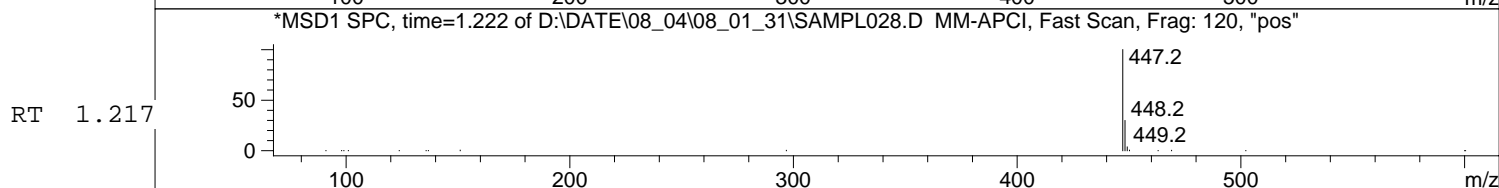

Supplement: Source data 2. [file elife-70700-data2.zip › Supplementary Material_source_data/Figure 1-figure supplement 1 & Supplementary1a-source/Z5.PDF]

MaxPeak: 95.15%  
Ret\_Time: 0.701 min

1831447

OK

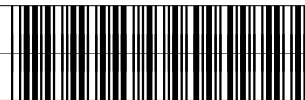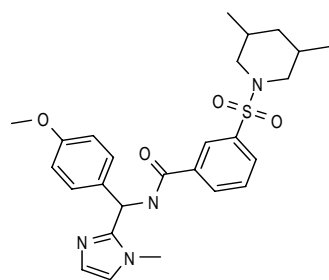

mw = 496.63

| # | Time  | Area% |
|---|-------|-------|
| 1 | 0.701 | 95.15 |
| 2 | 0.804 | 4.85  |

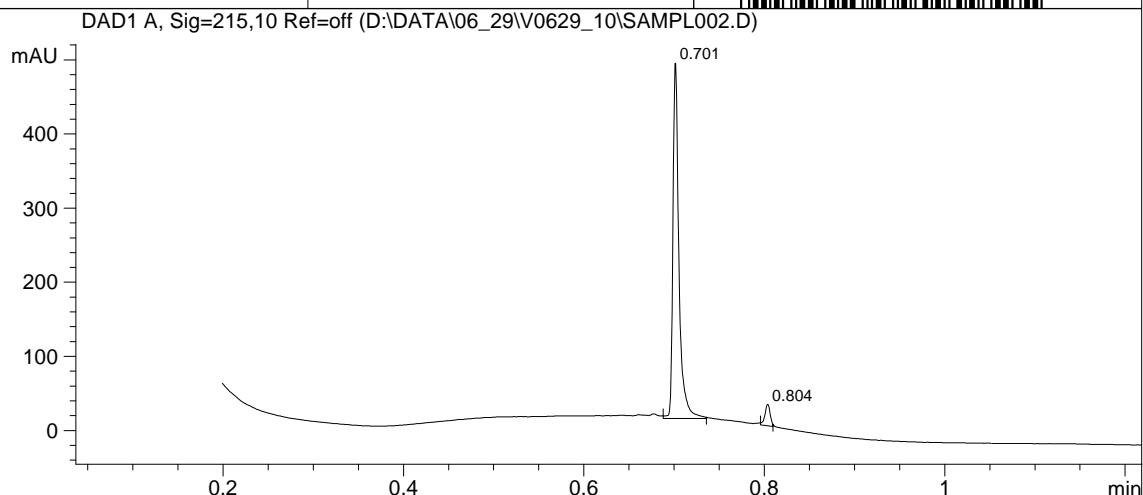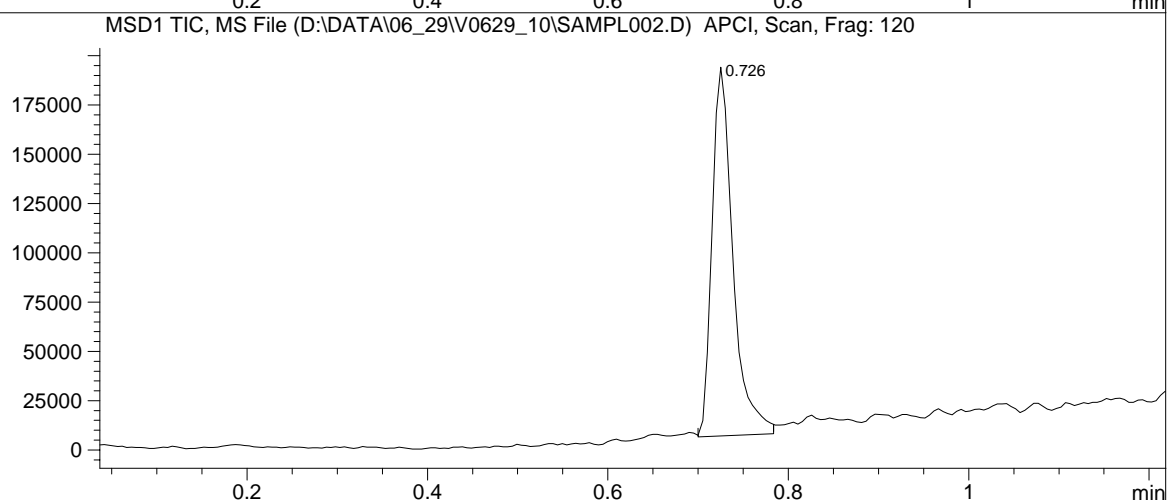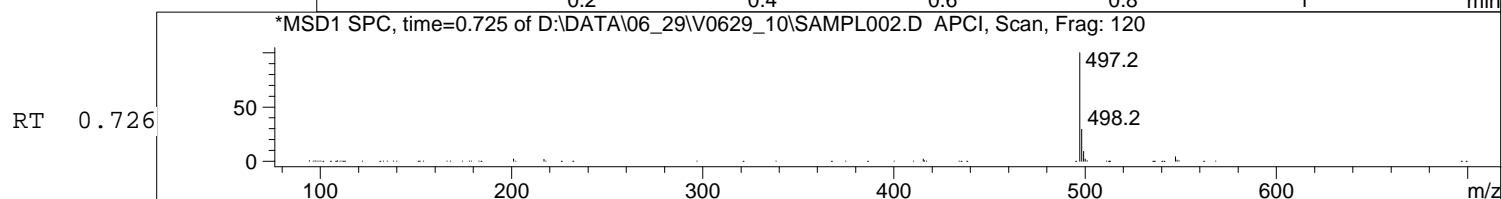

Supplement: Source data 2. [file elife-70700-data2.zip › Supplementary Material_source_data/Figure 1-figure supplement 1 & Supplementary1a-source/Z9.PDF]

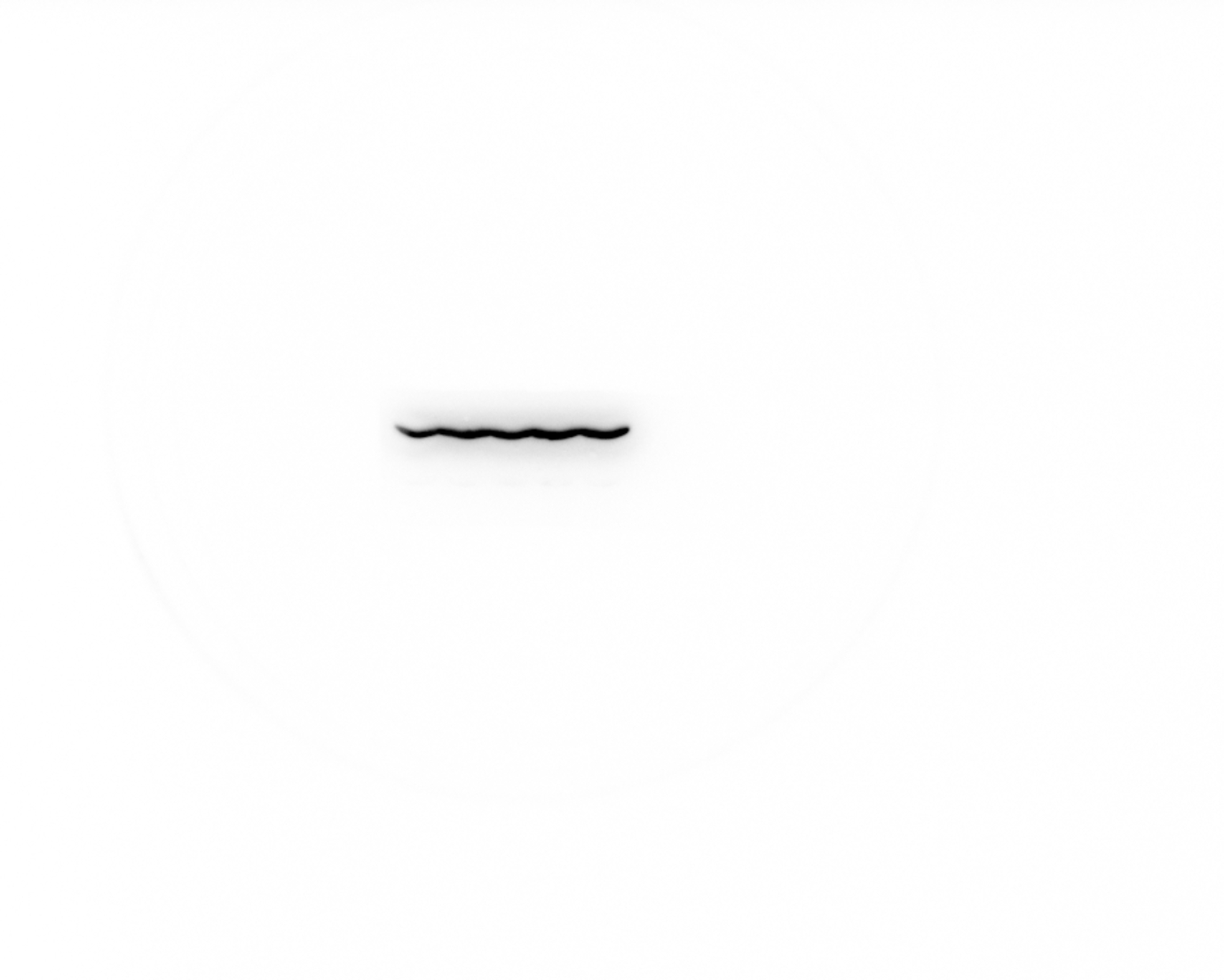

Supplement: Source data 2. [file elife-70700-data2.zip › Supplementary Material_source_data/Figure 2-figure supplement 3-source data 1/Figure 2-figure supplement 3-actin.jpg]

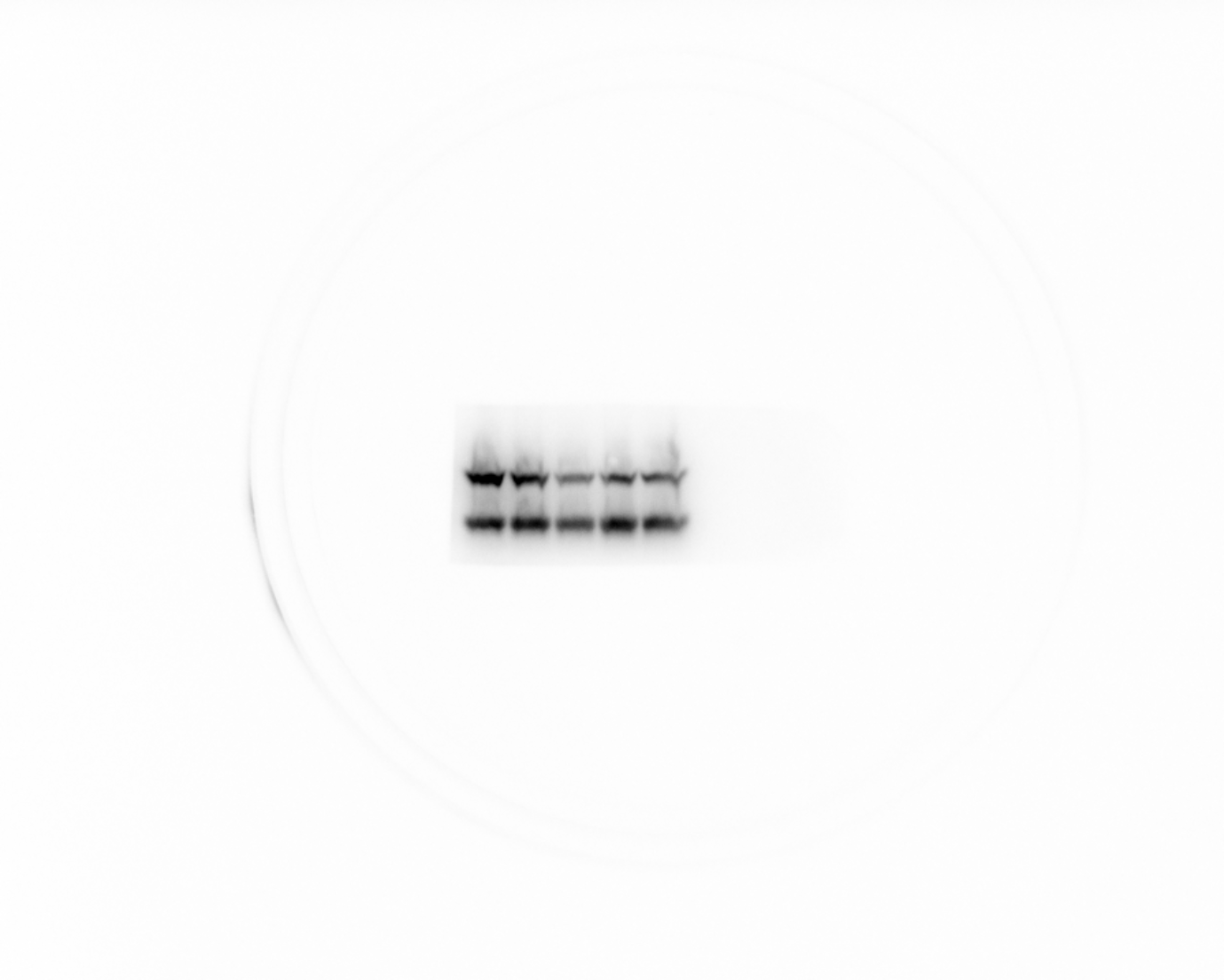

Supplement: Source data 2. [file elife-70700-data2.zip › Supplementary Material_source_data/Figure 2-figure supplement 3-source data 1/Figure 2-figure supplement 3-AR.tif]

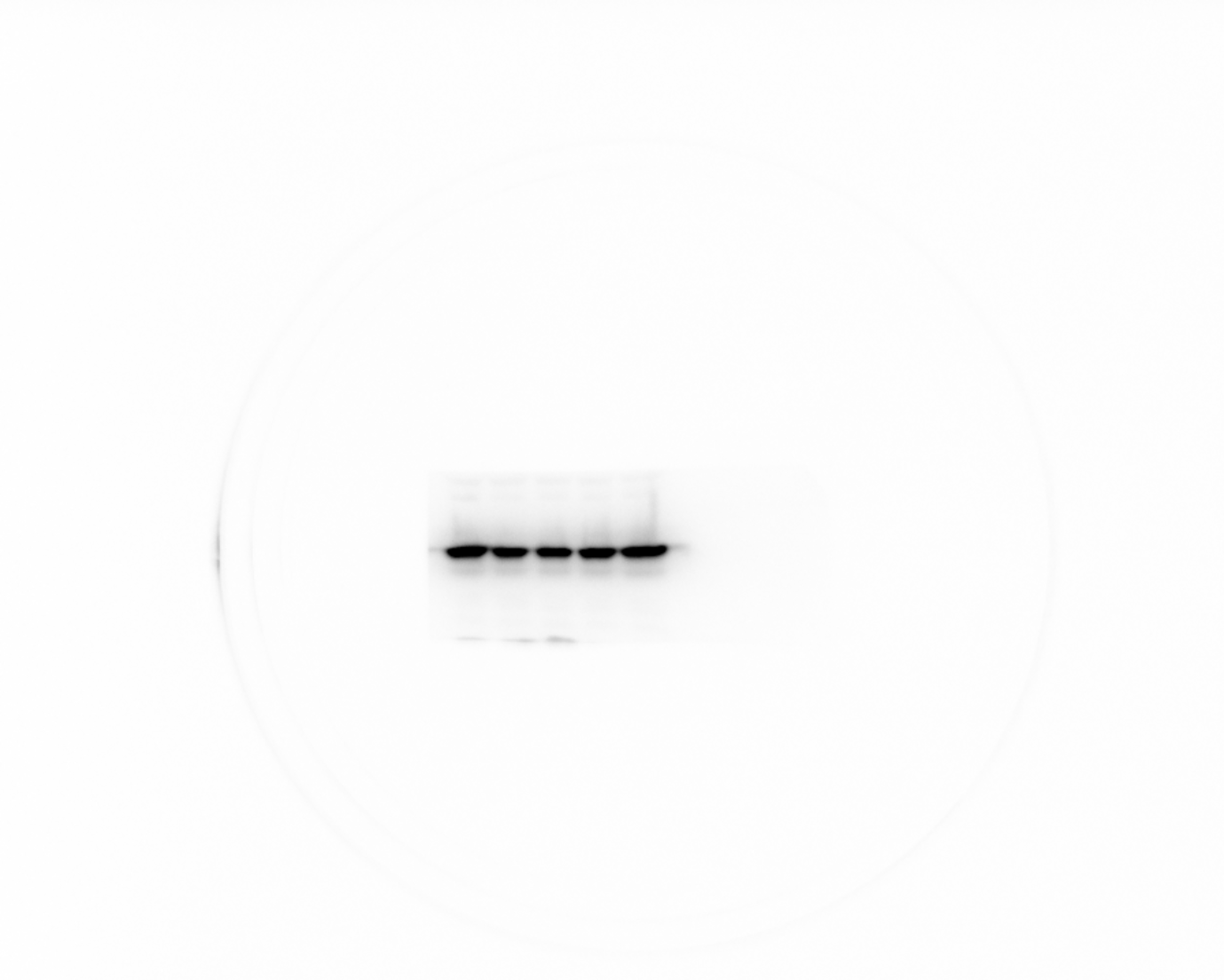

Supplement: Source data 2. [file elife-70700-data2.zip › Supplementary Material_source_data/Figure 2-figure supplement 3-source data 1/Figure 2-figure supplement 3-CDK7.tif]

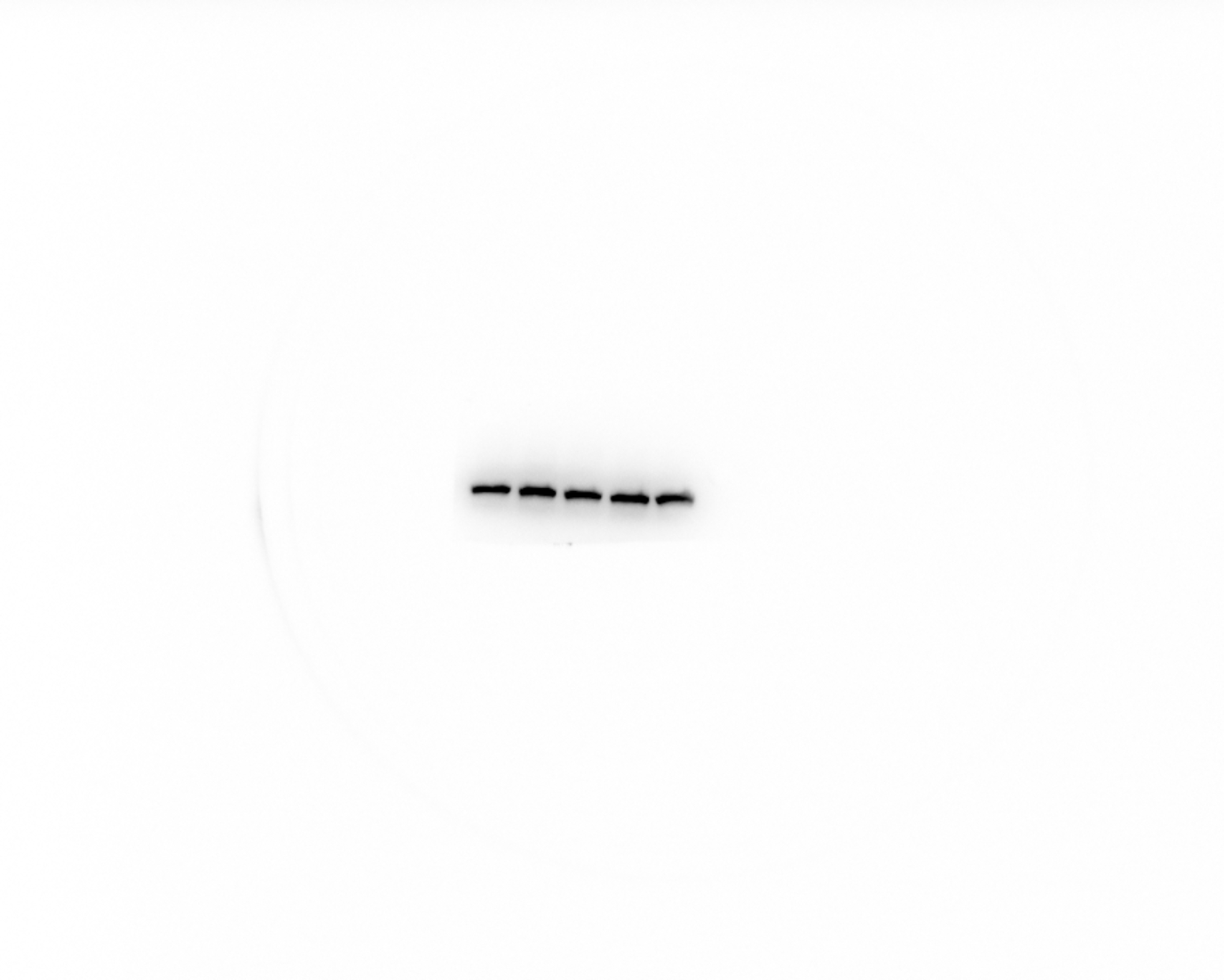

Supplement: Source data 2. [file elife-70700-data2.zip › Supplementary Material_source_data/Figure 2-figure supplement 3-source data 1/Figure 2-figure supplement 3-GR.jpg]

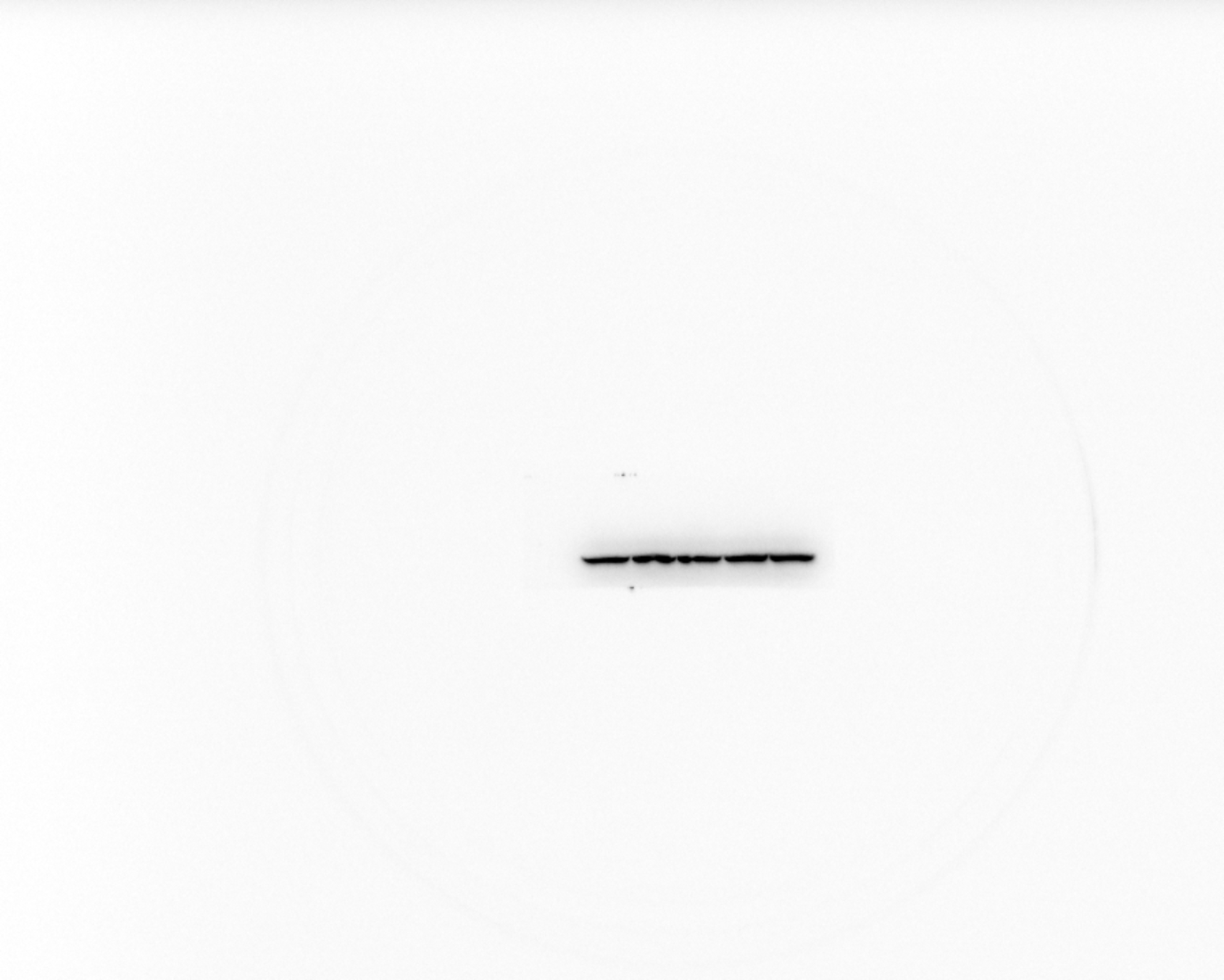

Supplement: Source data 2. [file elife-70700-data2.zip › Supplementary Material_source_data/Figure 2-figure supplement 3-source data 1/Figure 2-figure supplement 3-HSP90.jpg]

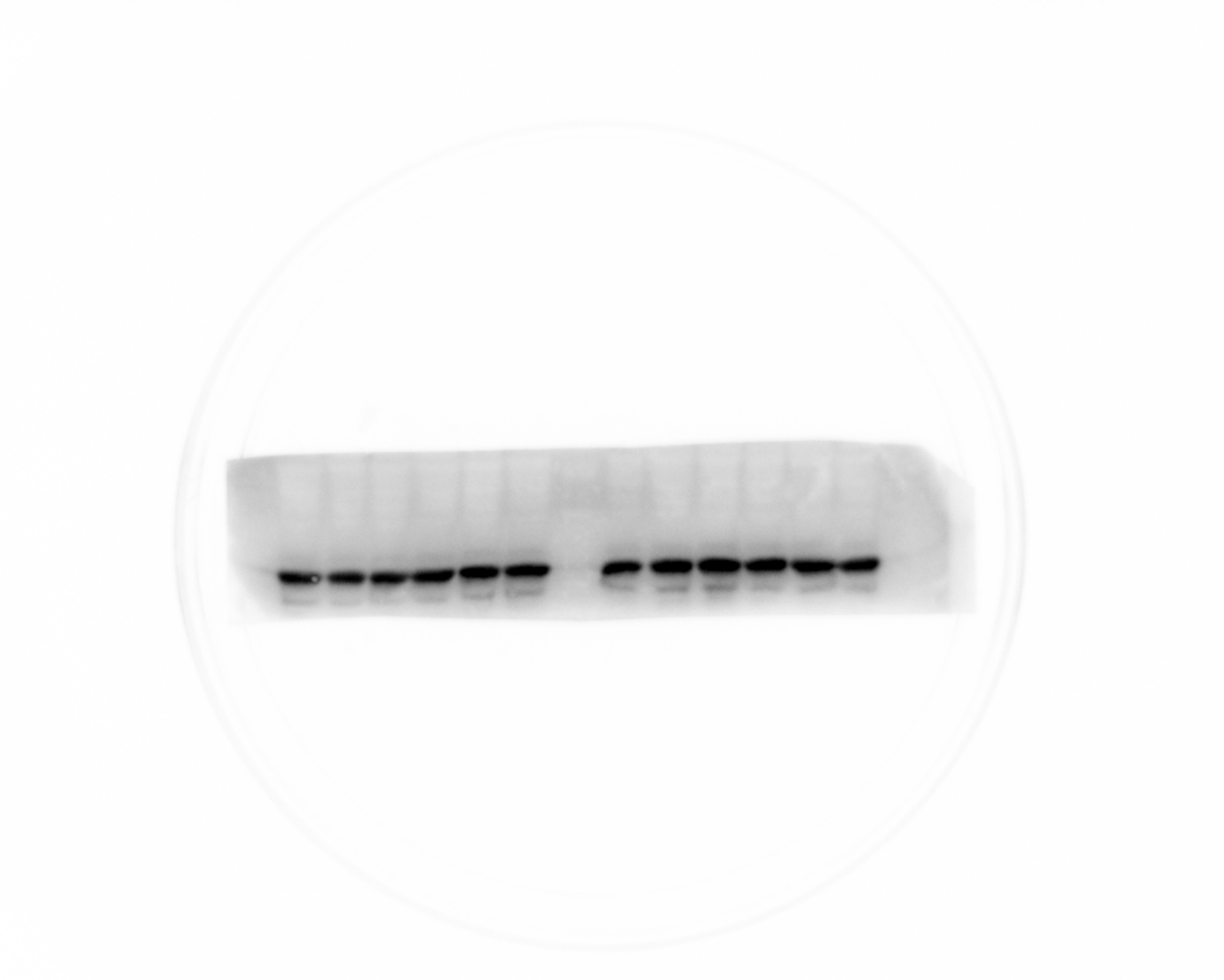

Supplement: Source data 2. [file elife-70700-data2.zip › Supplementary Material_source_data/Figure 4-figure supplement 2-source data 1/Figure 4-figure supplement 2A-actin.tif]

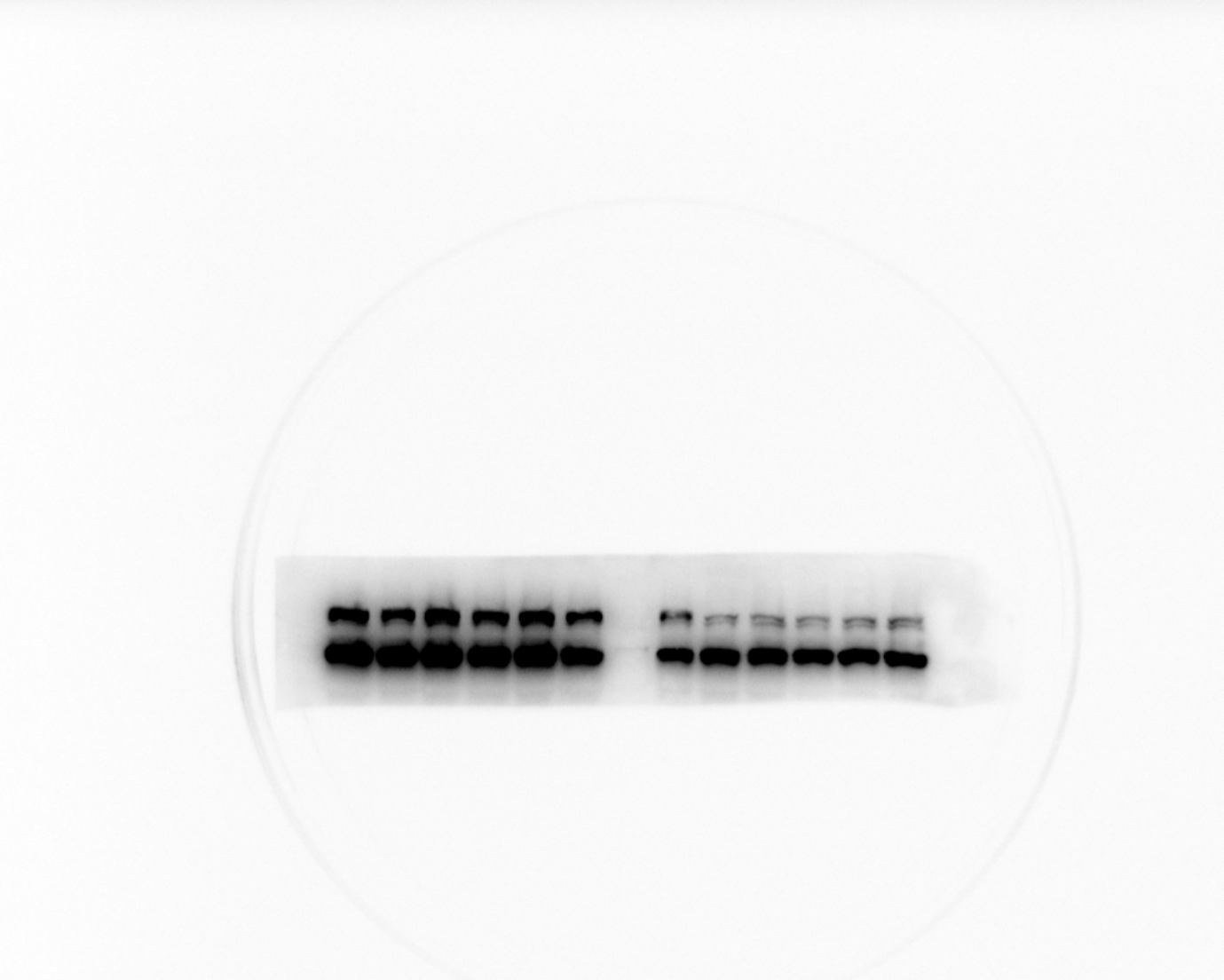

Supplement: Source data 2. [file elife-70700-data2.zip › Supplementary Material_source_data/Figure 4-figure supplement 2-source data 1/Figure 4-figure supplement 2A-AR.tif]

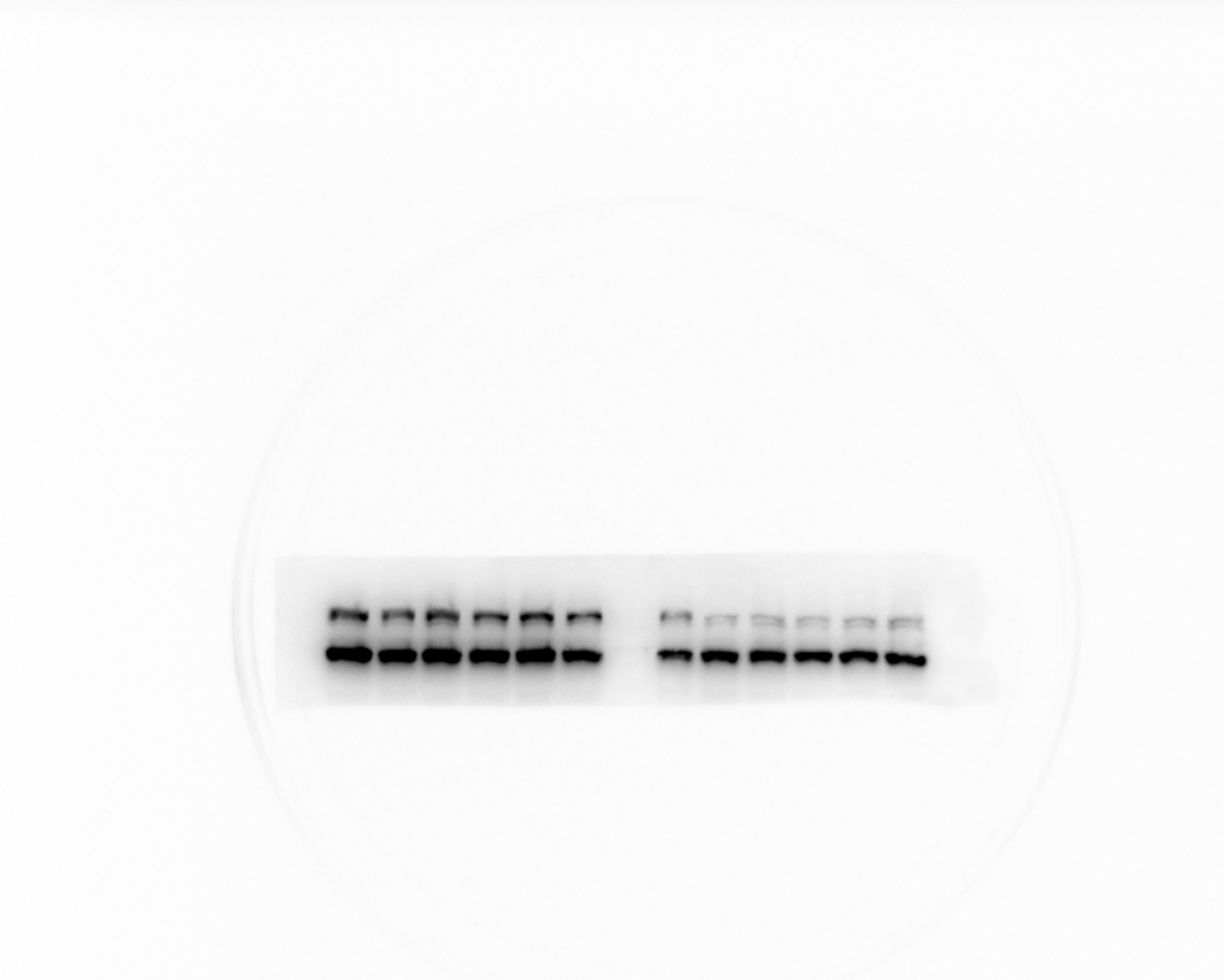

Supplement: Source data 2. [file elife-70700-data2.zip › Supplementary Material_source_data/Figure 4-figure supplement 2-source data 1/Figure 4-figure supplement 2D-AR.tif]

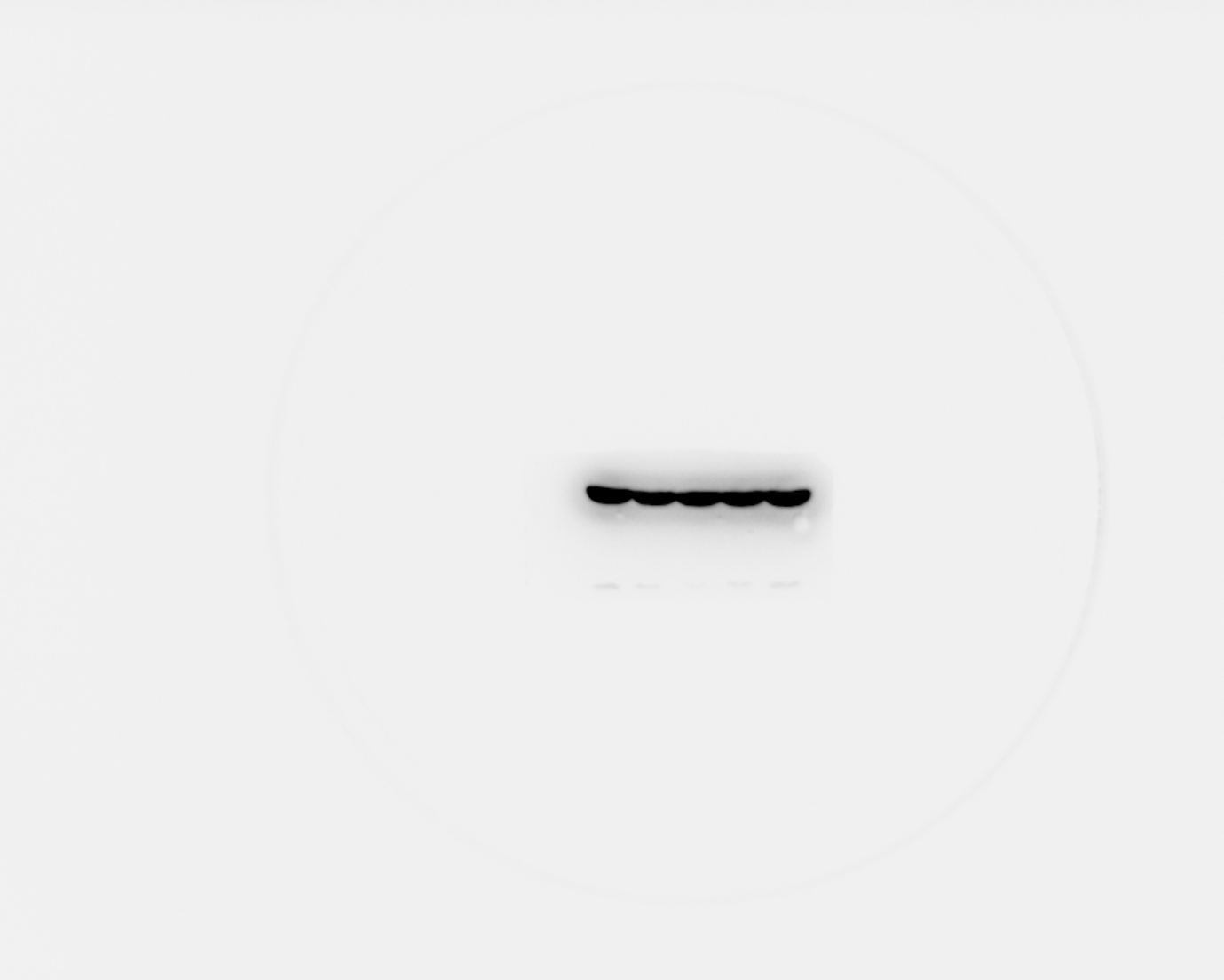

Supplement: Source data 2. [file elife-70700-data2.zip › Supplementary Material_source_data/Figure 6-figure supplement 1-source data 1/Figure 6-figure supplement 1A-actin.tif]

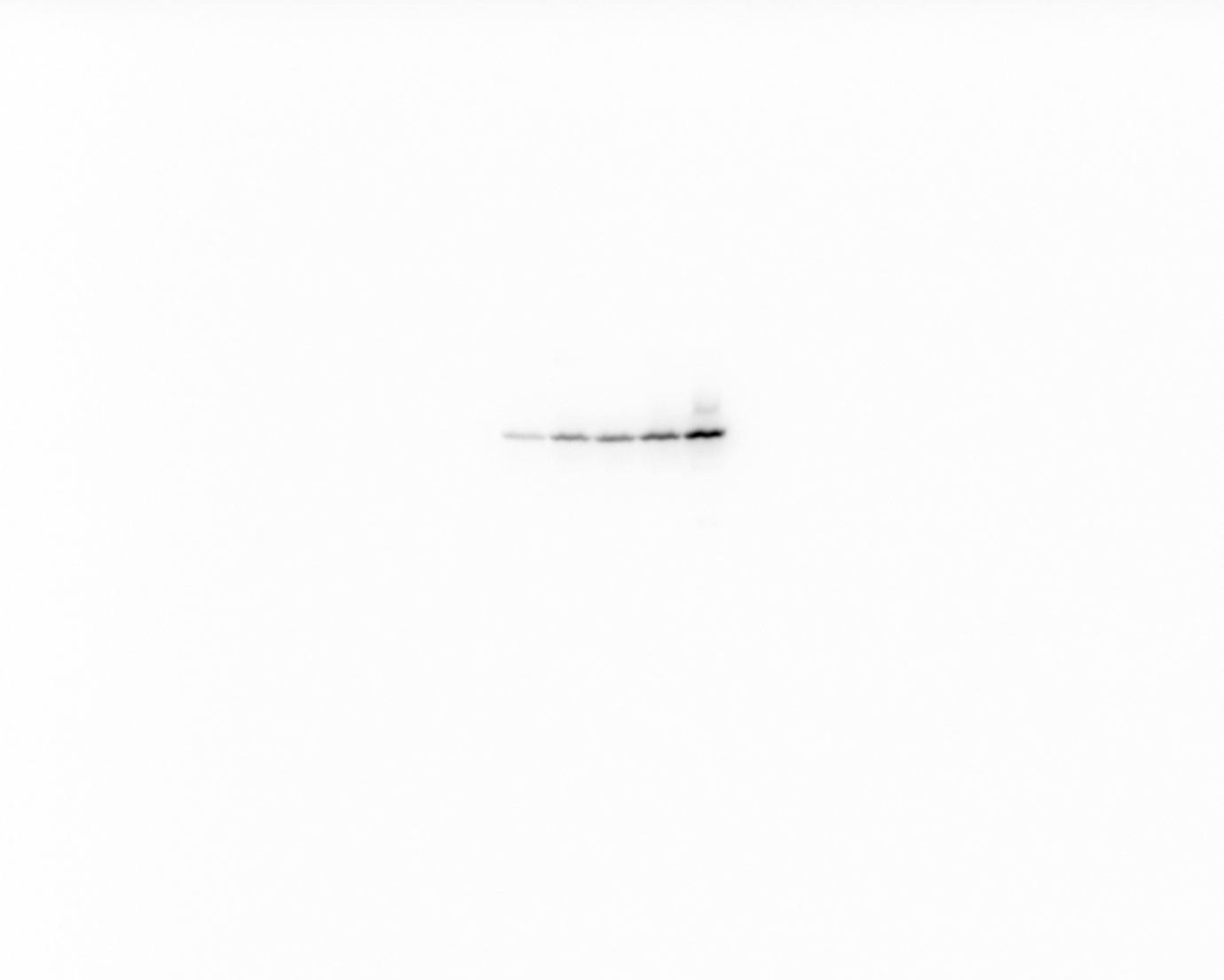

Supplement: Source data 2. [file elife-70700-data2.zip › Supplementary Material_source_data/Figure 6-figure supplement 1-source data 1/Figure 6-figure supplement 1A-Cleaved PARP.tif]

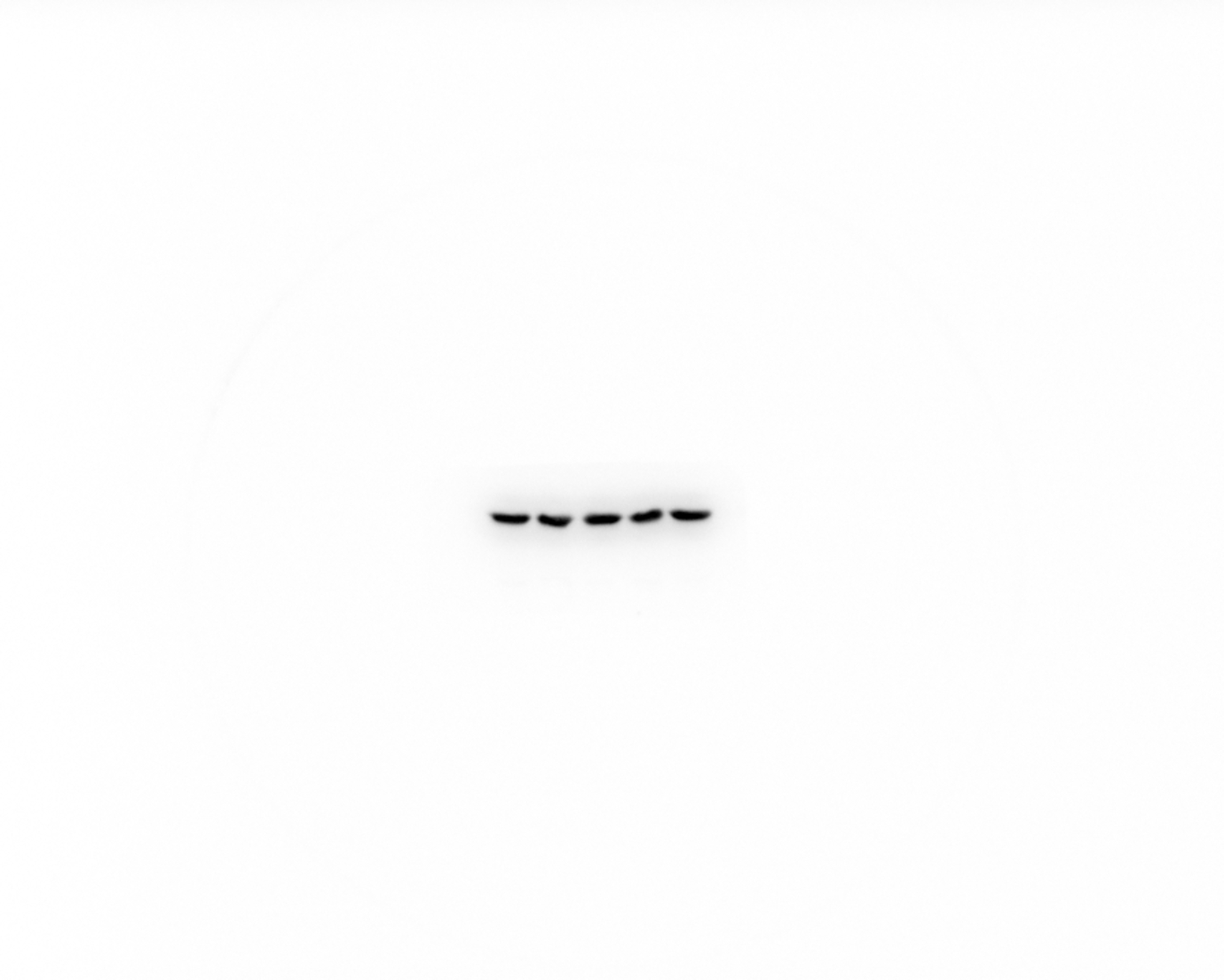

Supplement: Source data 2. [file elife-70700-data2.zip › Supplementary Material_source_data/Figure 6-figure supplement 1-source data 1/Figure 6-figure supplement 1B-actin.jpg]

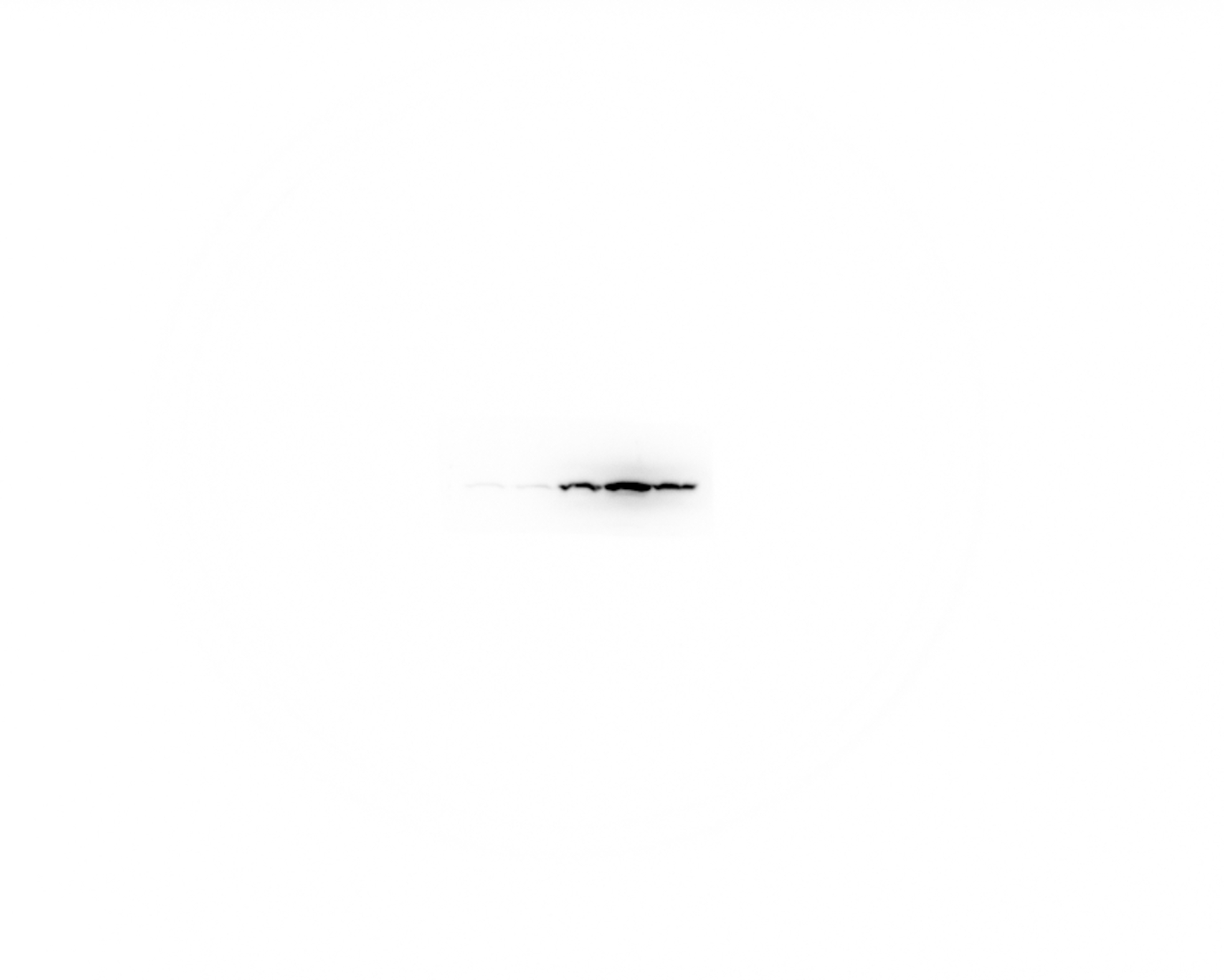

Supplement: Source data 2. [file elife-70700-data2.zip › Supplementary Material_source_data/Figure 6-figure supplement 1-source data 1/Figure 6-figure supplement 1B-Cleaved PARP.jpg]

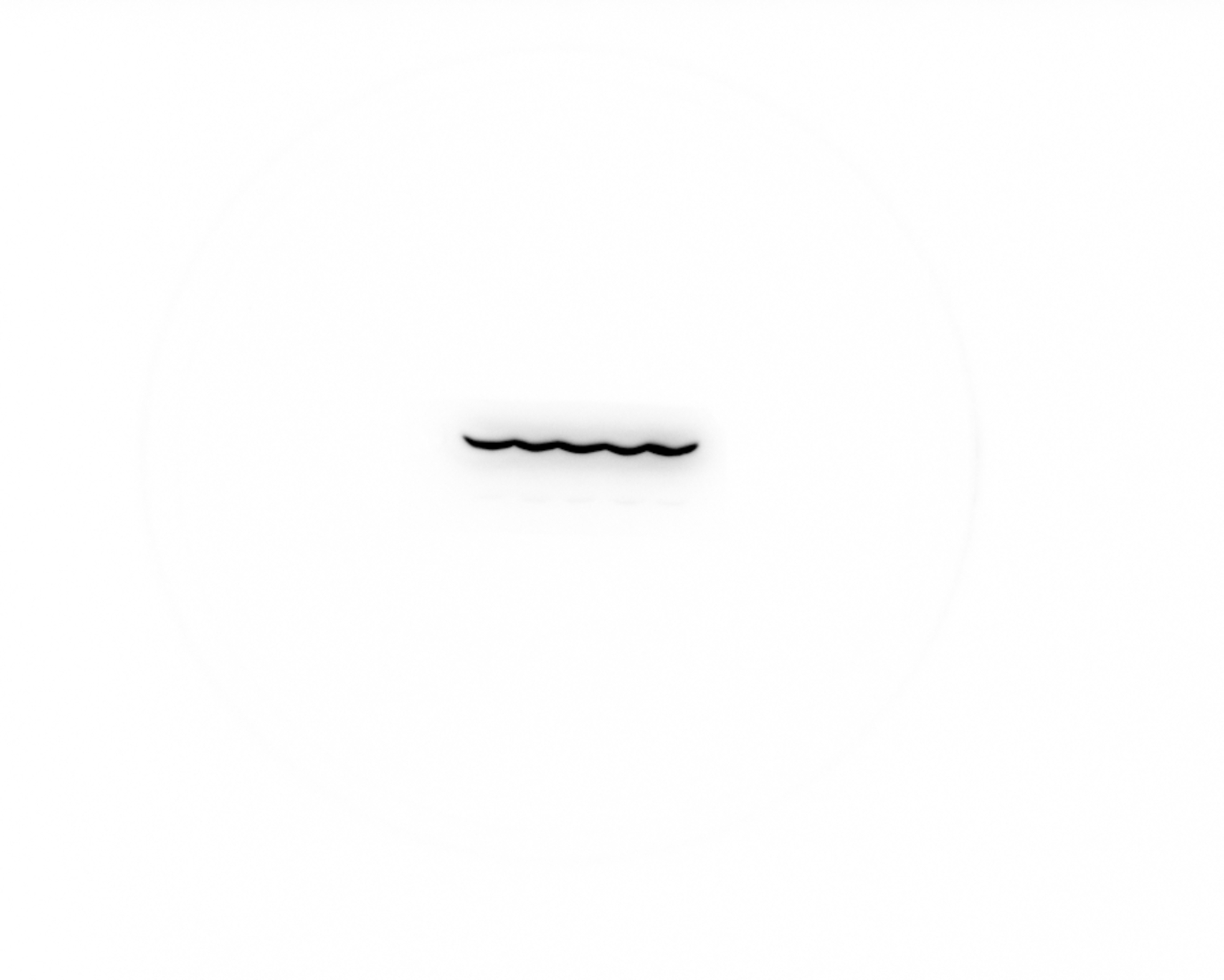

Supplement: Source data 2. [file elife-70700-data2.zip › Supplementary Material_source_data/Figure 6-figure supplement 1-source data 1/Figure 6-figure supplement 1E-actin.jpg]

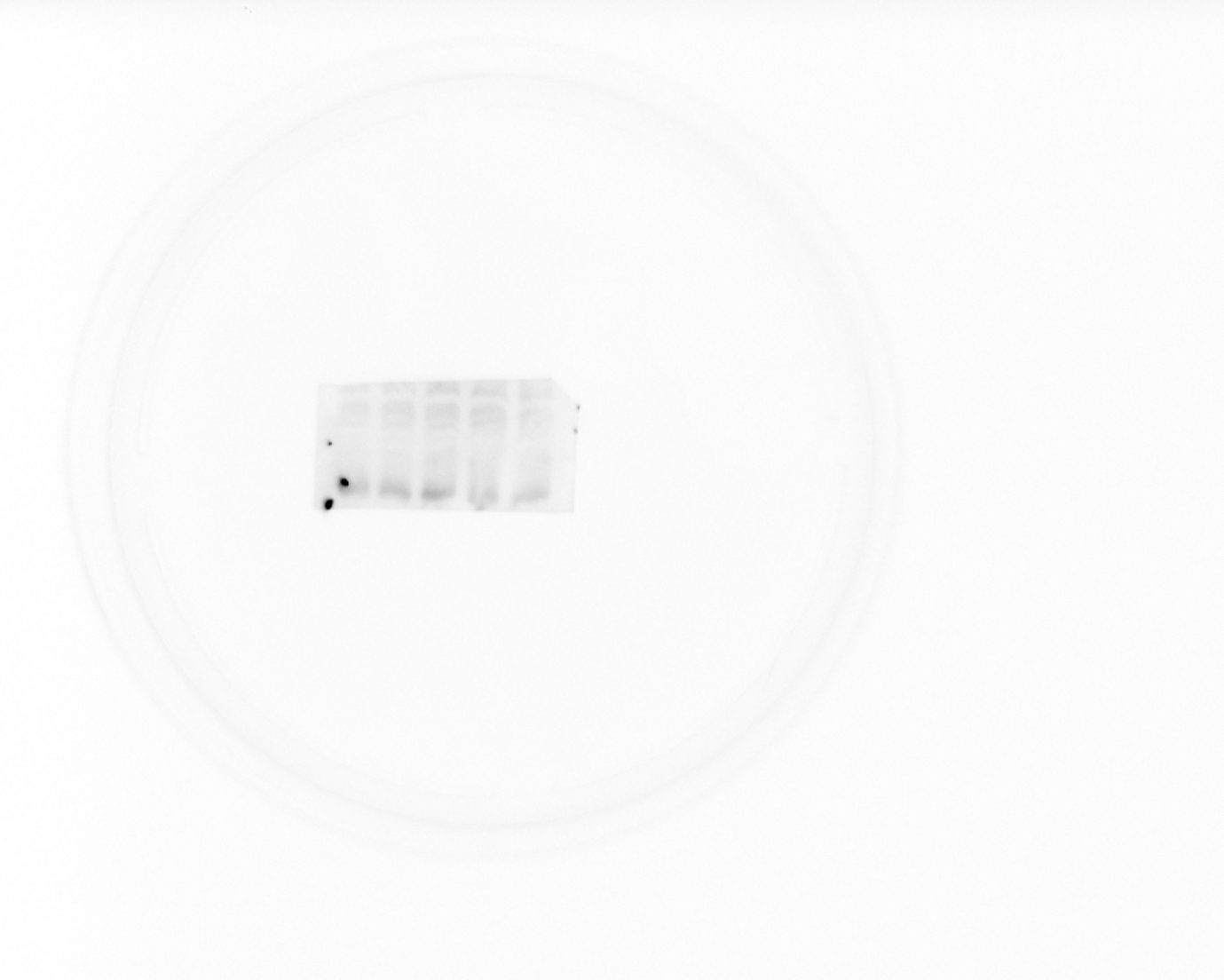

Supplement: Source data 2. [file elife-70700-data2.zip › Supplementary Material_source_data/Figure 6-figure supplement 1-source data 1/Figure 6-figure supplement 1E-Cleaved PARP.jpg]

MaxPeak: 100.00%  
Ret\_Time: 1.388 min

T6783885

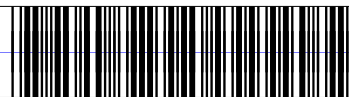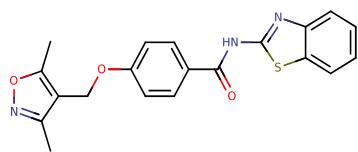

Mol Wt 379.43  
Exact Mass 379.11

| # | Time  | Area%  |
|---|-------|--------|
| 1 | 1.388 | 100.00 |

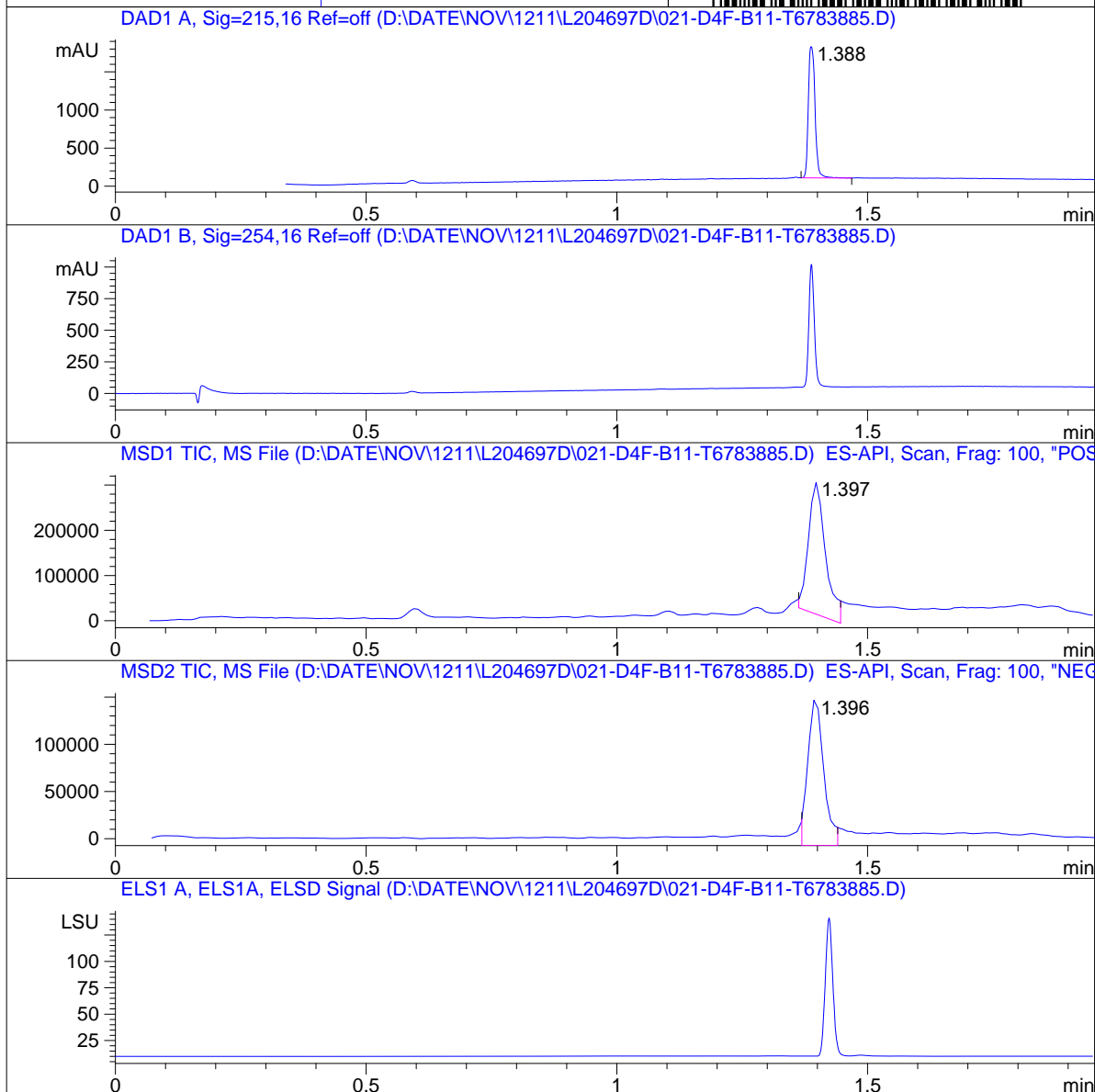

RT 1.397

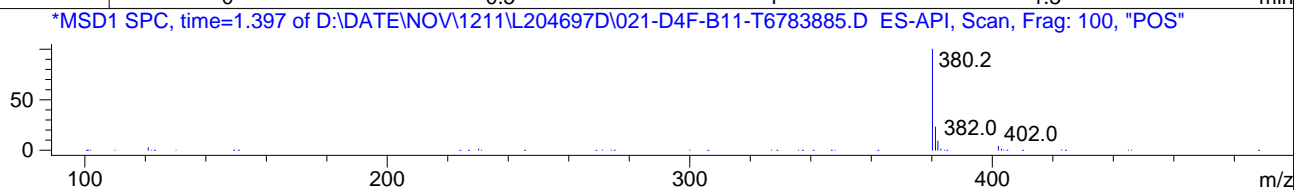

RT 1.396

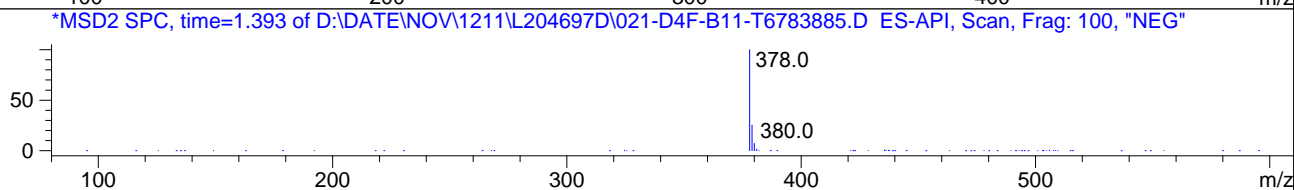

Supplement: Source data 2. [file elife-70700-data2.zip › Supplementary Material_source_data/Figure 8-figure supplement 1 & Supplementary file 1c-source/ZL-4-Z30198298.PDF]

MaxPeak: 100.00%  
Ret\_Time: 1.070 min

2665320

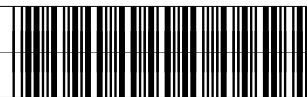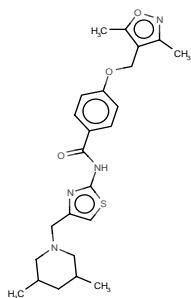

Mol Wt 454.586  
Exact Mass 454.24

| # | Time  | Area%  |
|---|-------|--------|
| 1 | 1.070 | 100.00 |

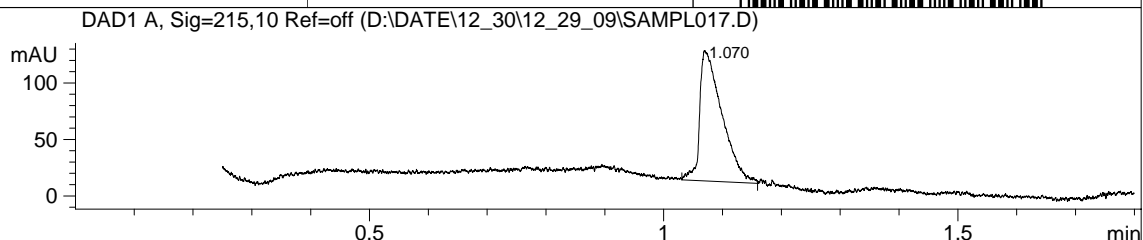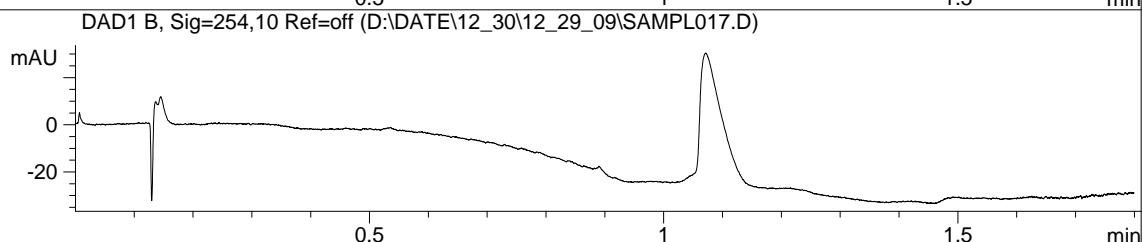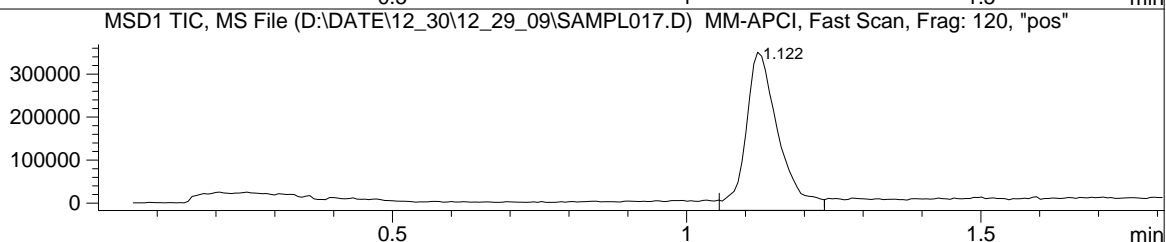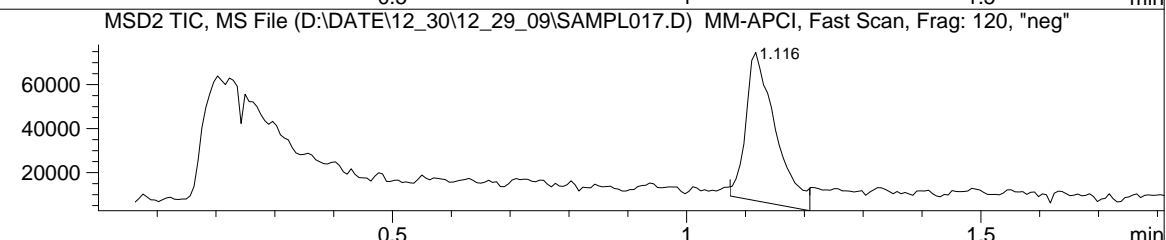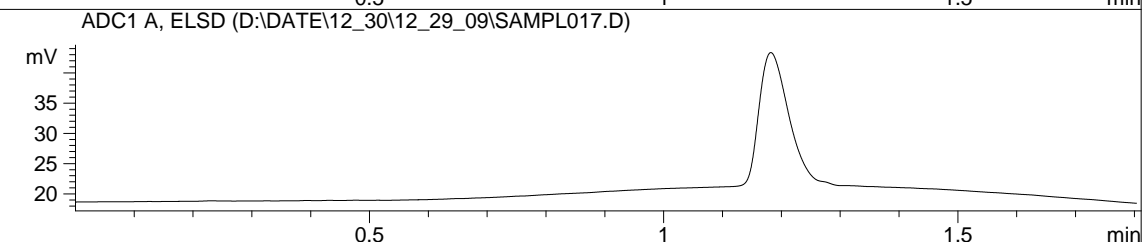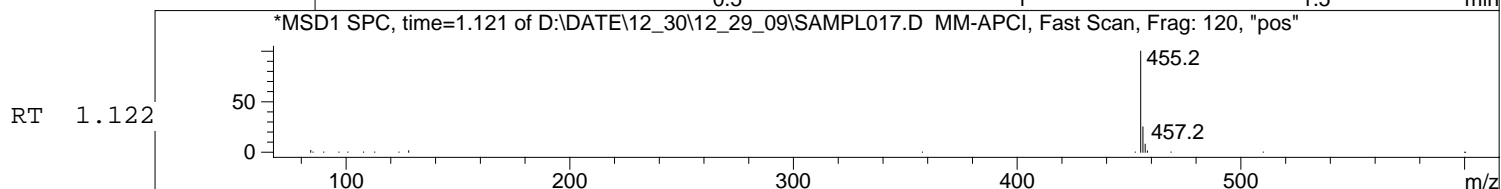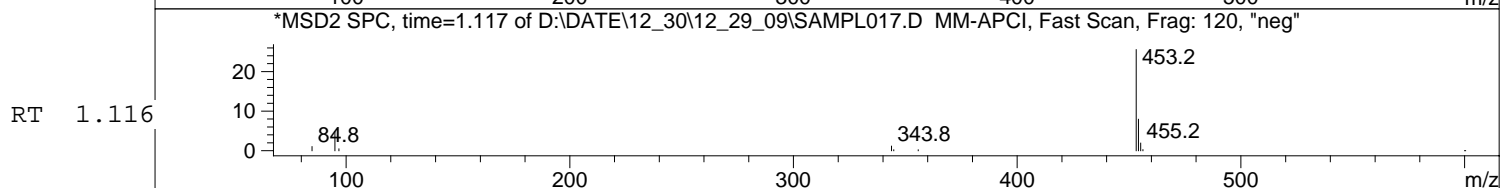

Supplement: Source data 2. [file elife-70700-data2.zip › Supplementary Material_source_data/Figure 8-figure supplement 1 & Supplementary file 1c-source/ZL-6-PB228330606.PDF]

MaxPeak: 95.23%  
Ret\_Time: 1.534 min

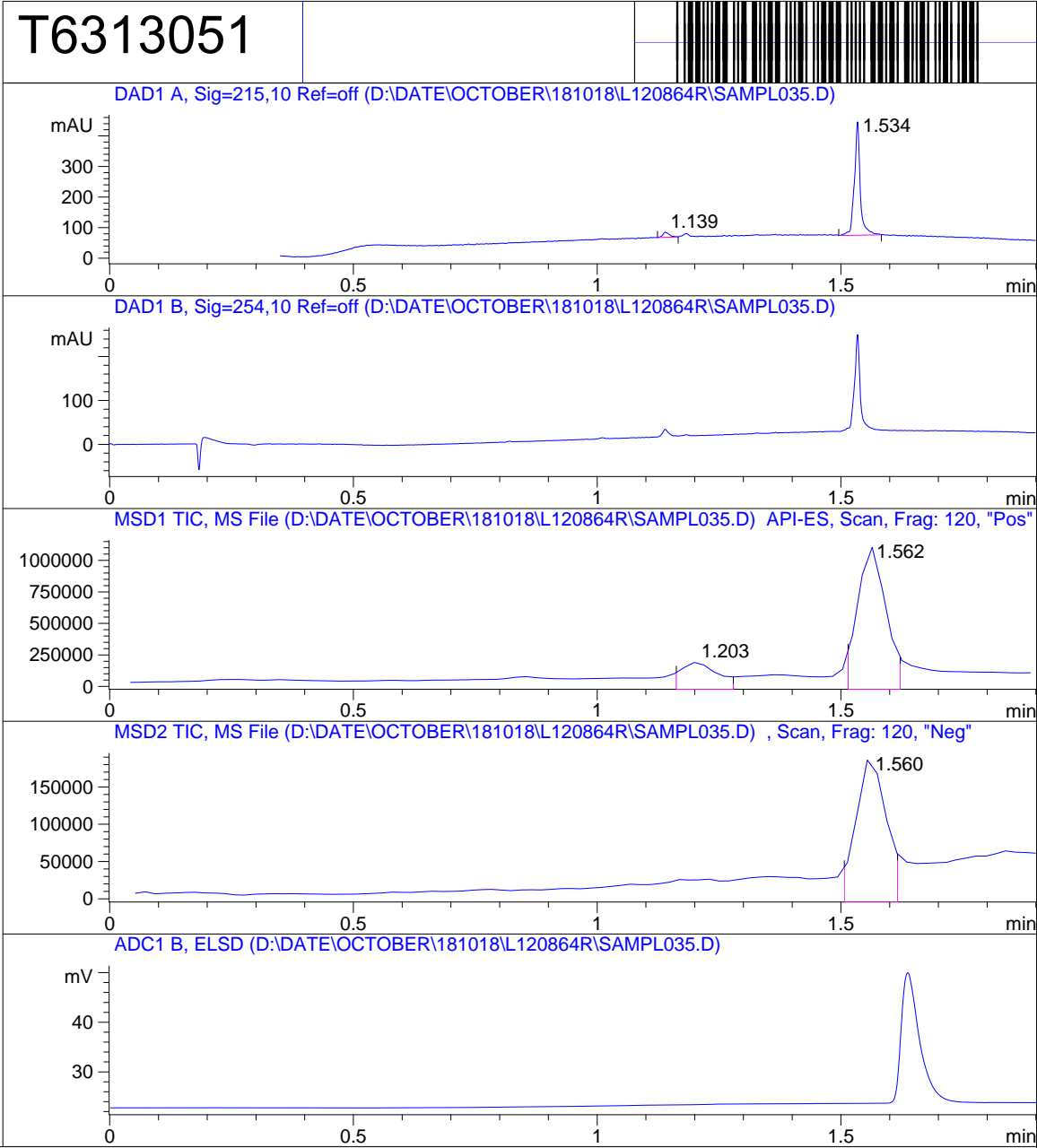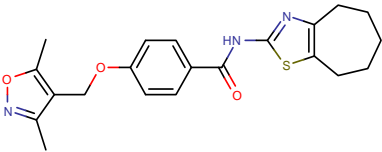

Mol Wt 397.49  
Exact Mass 397.17

| # | Time  | Area% |
|---|-------|-------|
| 1 | 1.139 | 4.77  |
| 2 | 1.534 | 95.23 |

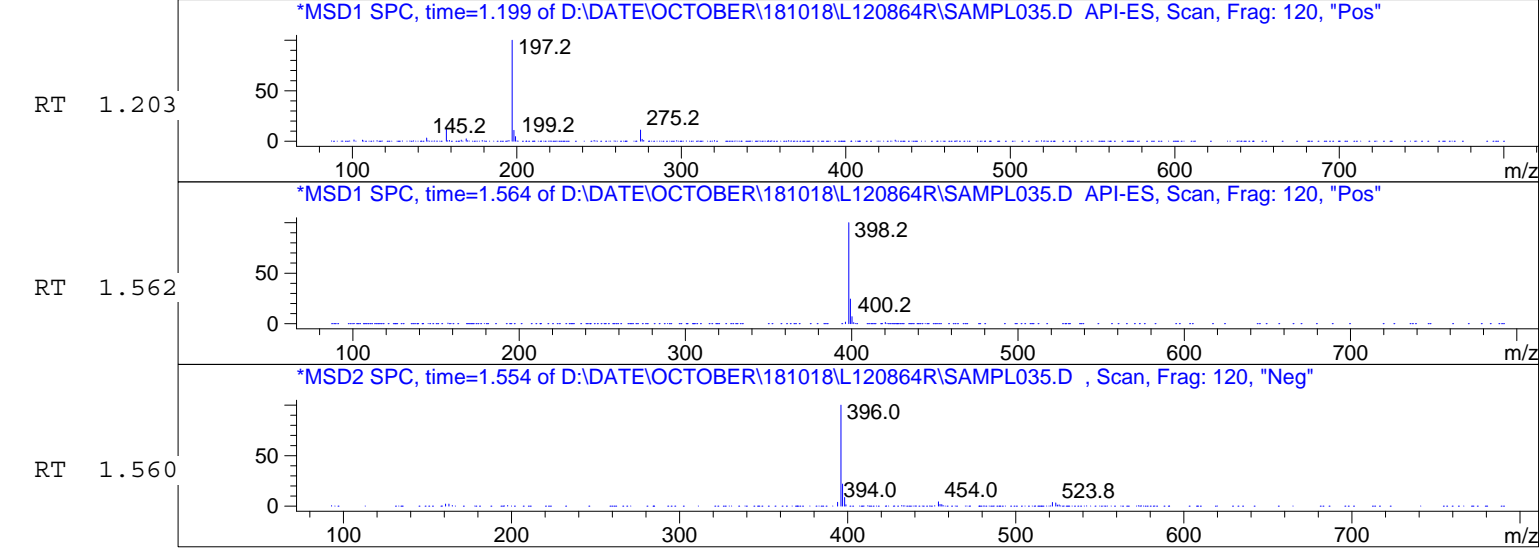

Supplement: Source data 2. [file elife-70700-data2.zip › Supplementary Material_source_data/Figure 8-figure supplement 1 & Supplementary file 1c-source/ZL-7-PB228374000.PDF]

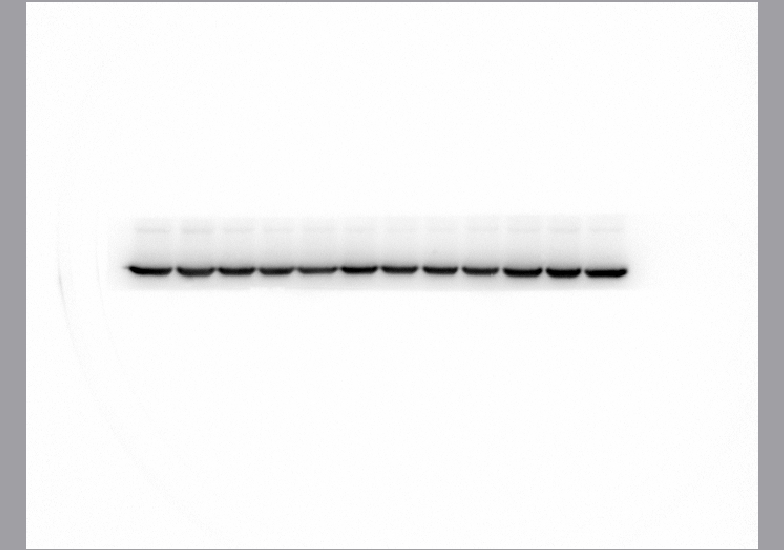

Supplement: Source data 2. [file elife-70700-data2.zip › Supplementary Material_source_data/Figure 8-figure supplement 2-source data 1/Figure 8-figure supplement 2B/Figure 8-figure supplement 2B-actin.jpg]

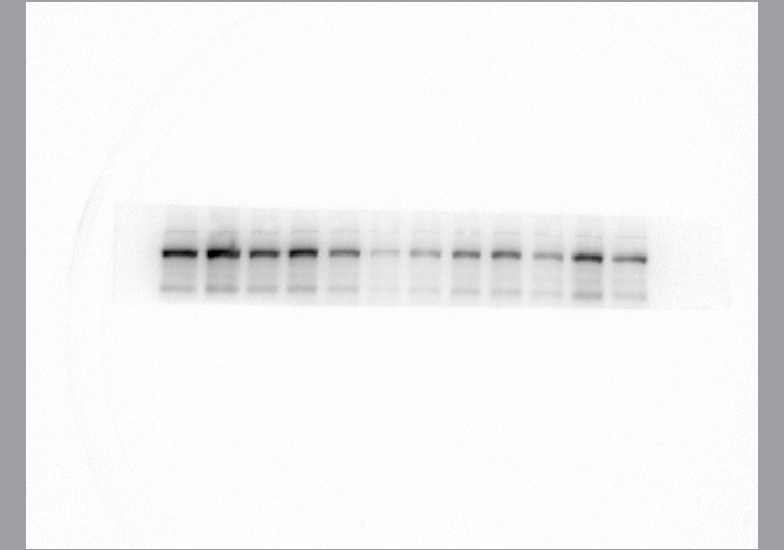

Supplement: Source data 2. [file elife-70700-data2.zip › Supplementary Material_source_data/Figure 8-figure supplement 2-source data 1/Figure 8-figure supplement 2B/Figure 8-figure supplement 2B-AR.jpg]

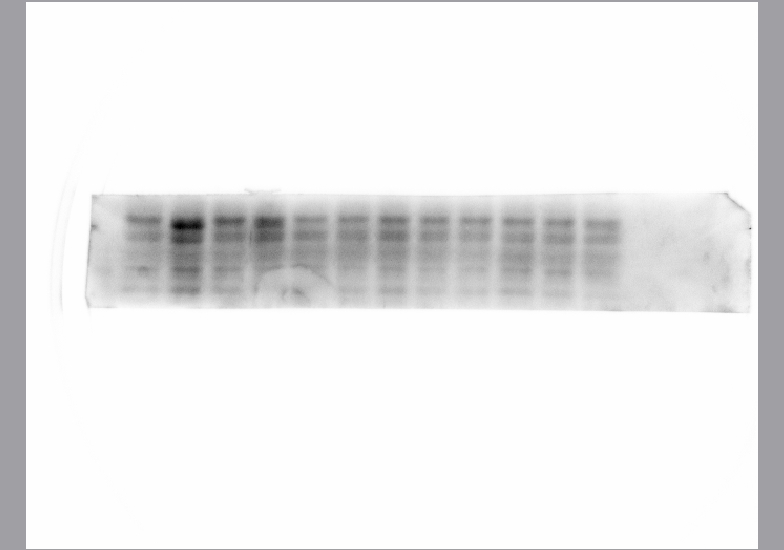

Supplement: Source data 2. [file elife-70700-data2.zip › Supplementary Material_source_data/Figure 8-figure supplement 2-source data 1/Figure 8-figure supplement 2B/Figure 8-figure supplement 2B-PSA.jpg]

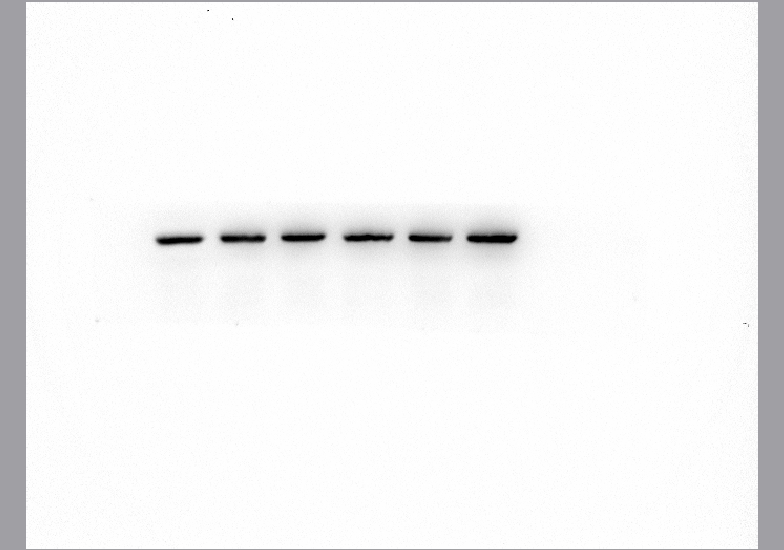

Supplement: Source data 3. [file elife-70700-data3.zip › Figure source data/Figure 2-source data 1/Figure 2D/Figure 2D-actin.jpg]

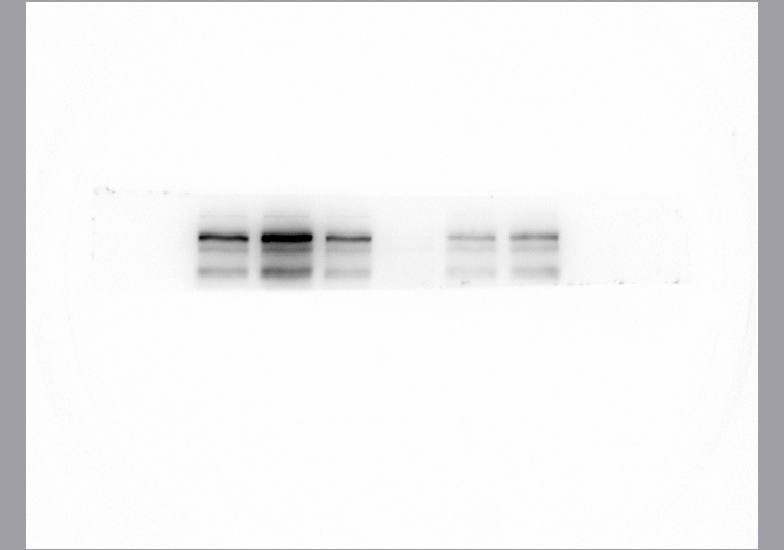

Supplement: Source data 3. [file elife-70700-data3.zip › Figure source data/Figure 2-source data 1/Figure 2D/Figure 2D-AR.jpg]

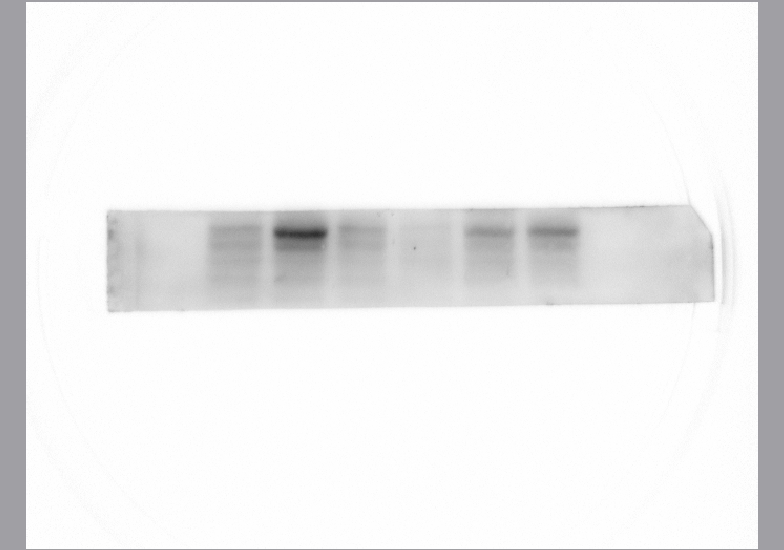

Supplement: Source data 3. [file elife-70700-data3.zip › Figure source data/Figure 2-source data 1/Figure 2D/Figure 2D-PSA.jpg]

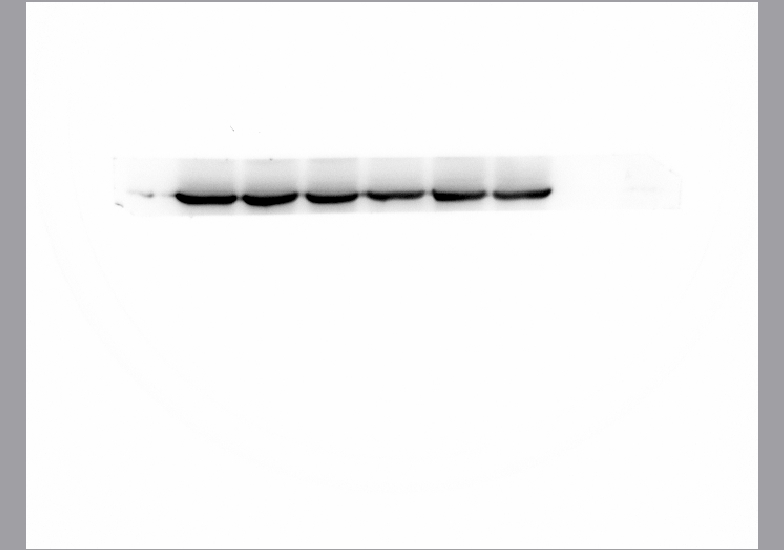

Supplement: Source data 3. [file elife-70700-data3.zip › Figure source data/Figure 2-source data 1/Figure 2E/Figure 2E-actin.jpg]

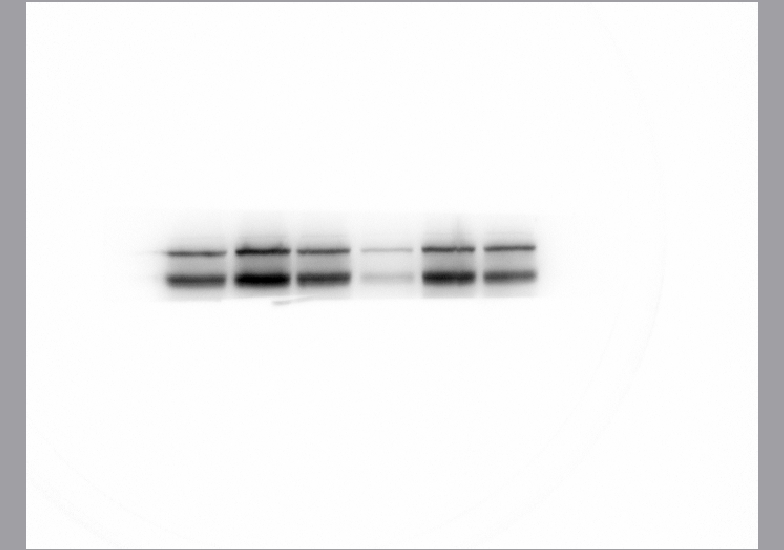

Supplement: Source data 3. [file elife-70700-data3.zip › Figure source data/Figure 2-source data 1/Figure 2E/Figure 2E-AR.jpg]

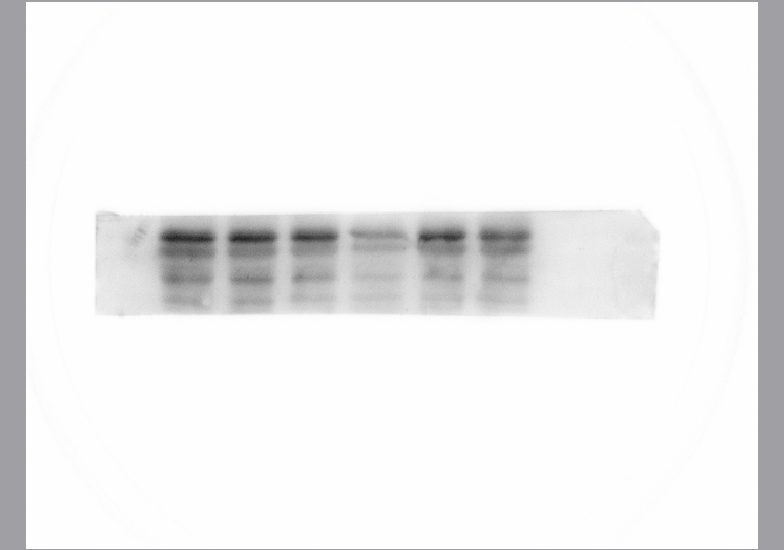

Supplement: Source data 3. [file elife-70700-data3.zip › Figure source data/Figure 2-source data 1/Figure 2E/Figure 2E-PSA.jpg]

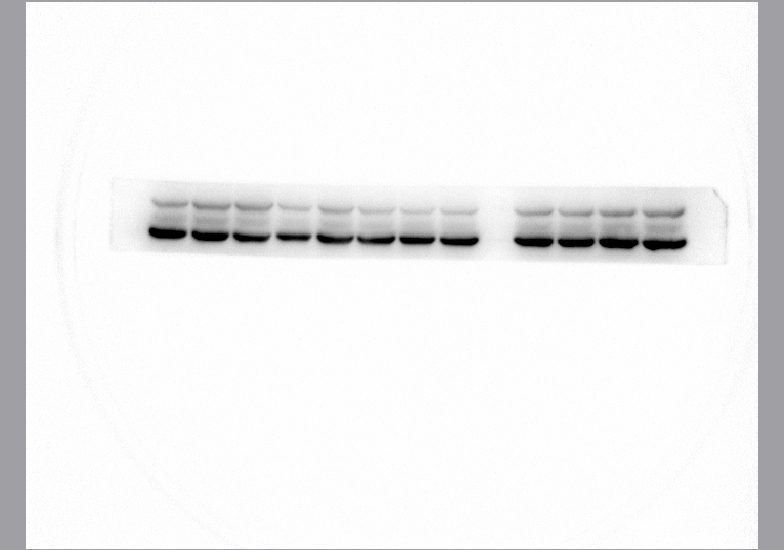

Supplement: Source data 3. [file elife-70700-data3.zip › Figure source data/Figure 2-source data 1/Figure 2F/Figure 2F-actin.jpg]

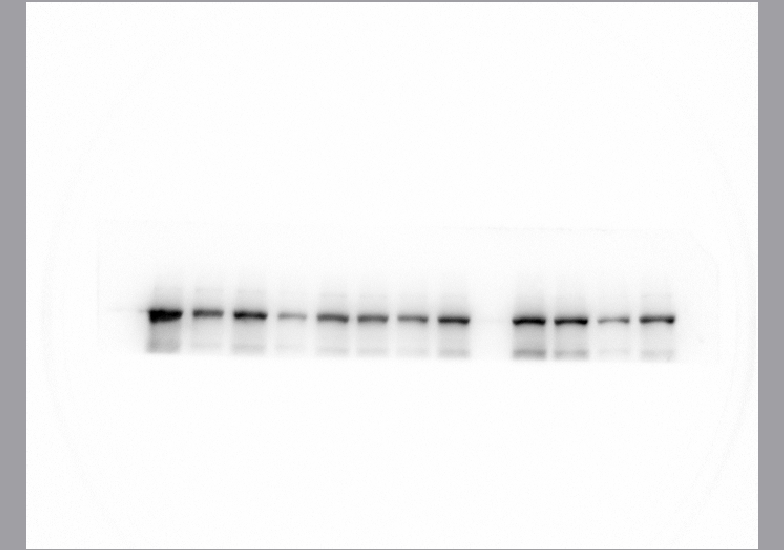

Supplement: Source data 3. [file elife-70700-data3.zip › Figure source data/Figure 2-source data 1/Figure 2F/Figure 2F-AR.jpg]

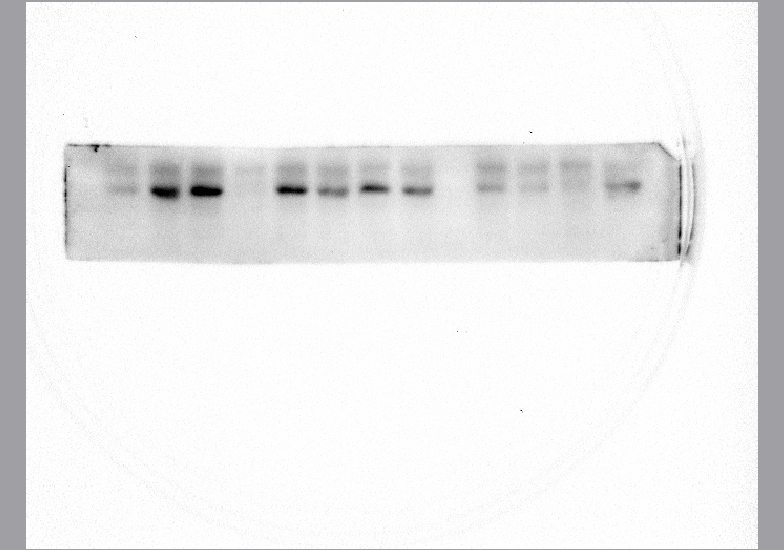

Supplement: Source data 3. [file elife-70700-data3.zip › Figure source data/Figure 2-source data 1/Figure 2F/Figure 2F-PSA.jpg]

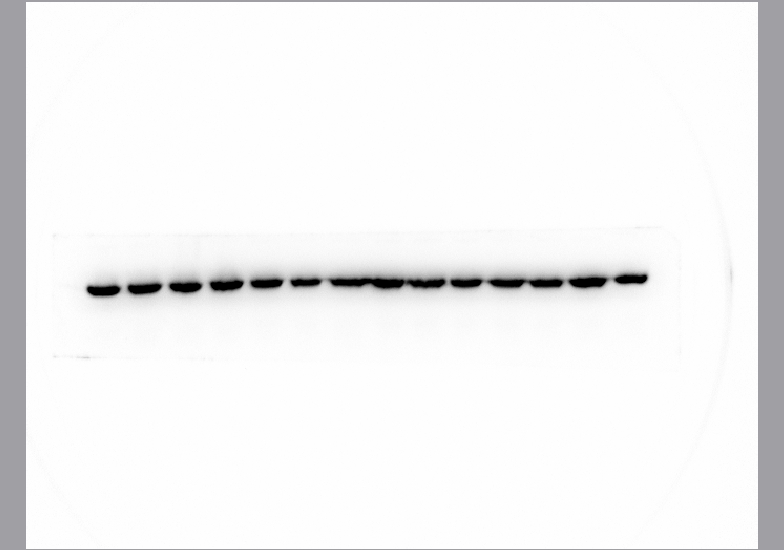

Supplement: Source data 3. [file elife-70700-data3.zip › Figure source data/Figure 2-source data 1/Figure 2G/Figure 2G-actin.jpg]

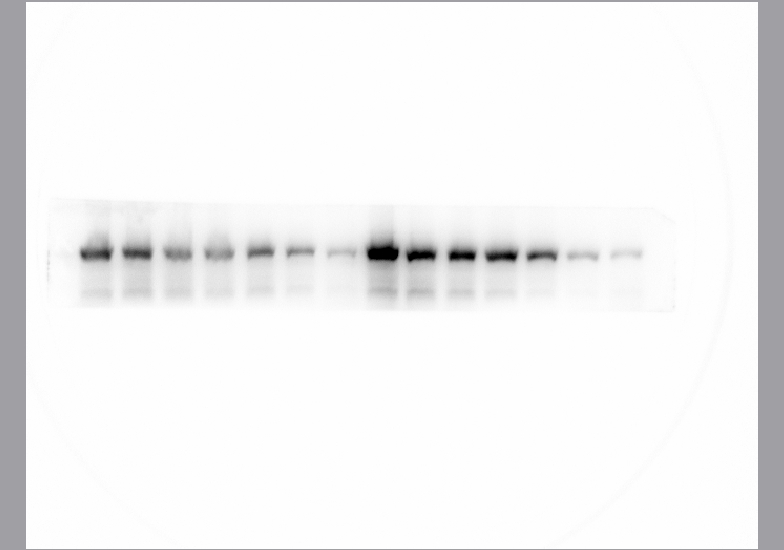

Supplement: Source data 3. [file elife-70700-data3.zip › Figure source data/Figure 2-source data 1/Figure 2G/Figure 2G-AR.jpg]

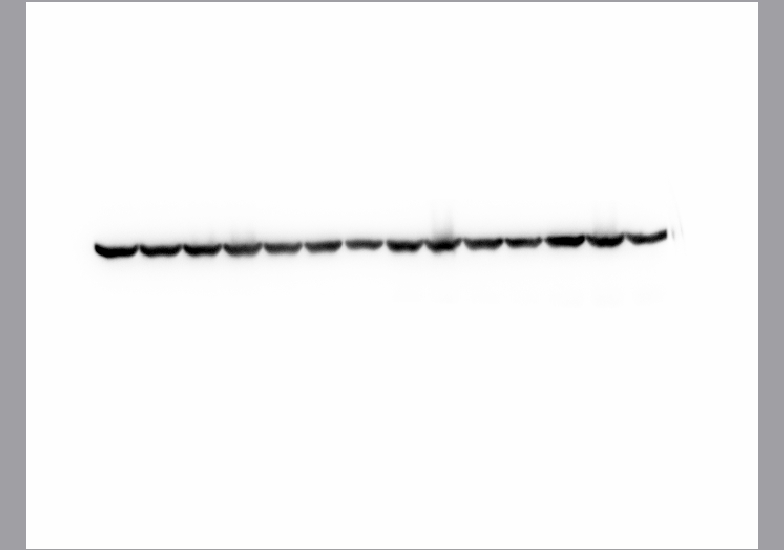

Supplement: Source data 3. [file elife-70700-data3.zip › Figure source data/Figure 2-source data 1/Figure 2H/Figure 2H-actin.jpg]

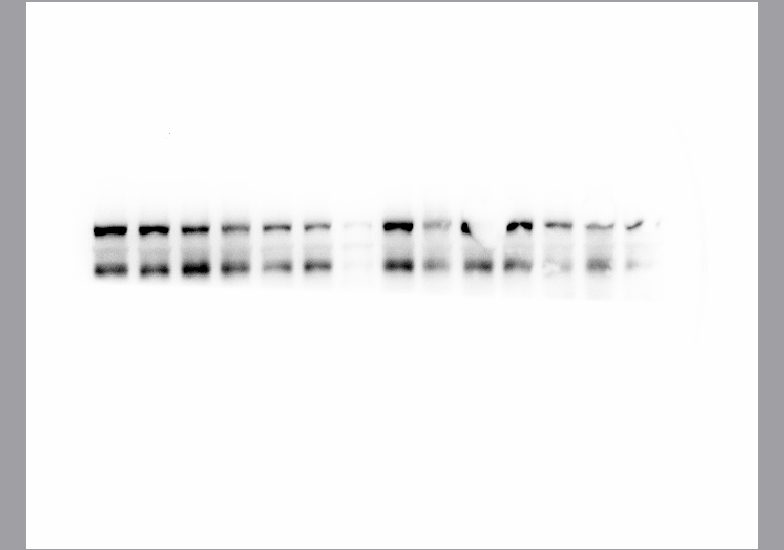

Supplement: Source data 3. [file elife-70700-data3.zip › Figure source data/Figure 2-source data 1/Figure 2H/Figure 2H-AR.jpg]

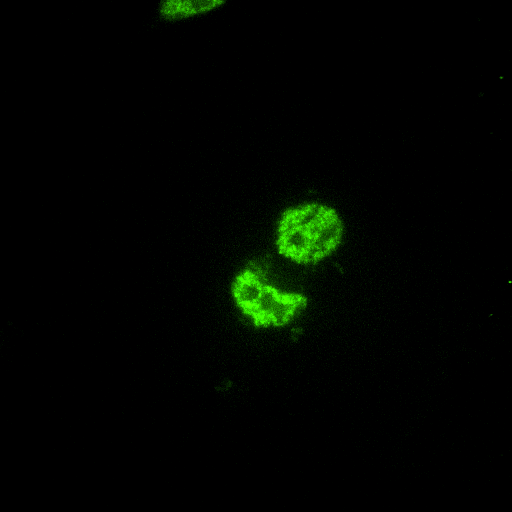

Supplement: Source data 3. [file elife-70700-data3.zip › Figure source data/Figure 3-source data 1/Figure 3-DHT_AR.tif]

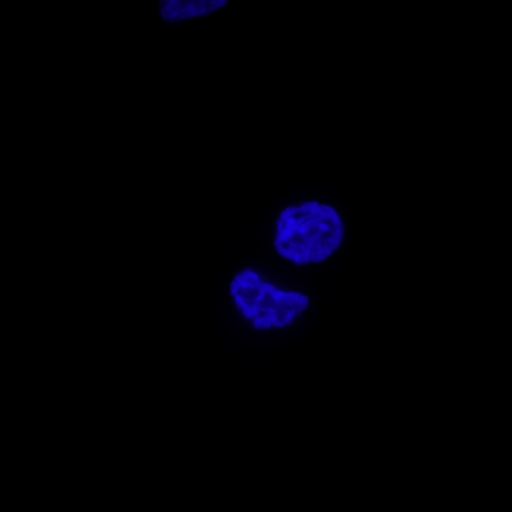

Supplement: Source data 3. [file elife-70700-data3.zip › Figure source data/Figure 3-source data 1/Figure 3-DHT_DAPI.tif]

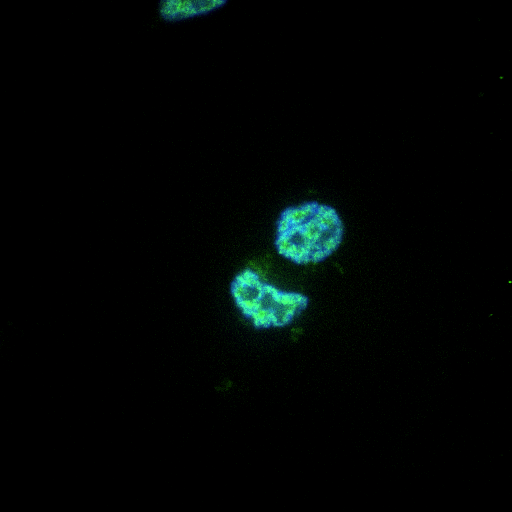

Supplement: Source data 3. [file elife-70700-data3.zip › Figure source data/Figure 3-source data 1/Figure 3-DHT_Merge.tif]

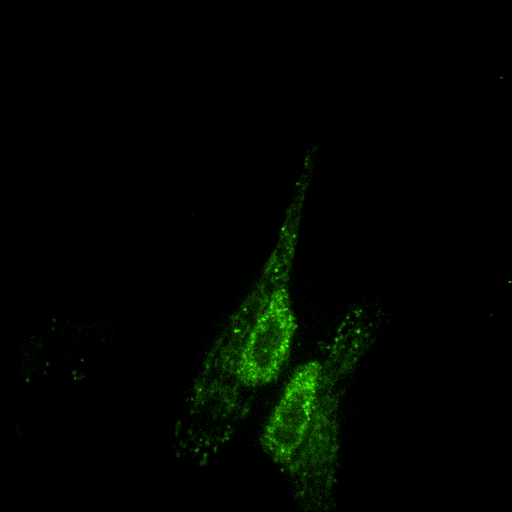

Supplement: Source data 3. [file elife-70700-data3.zip › Figure source data/Figure 3-source data 1/Figure 3-DMSO_AR.tif]

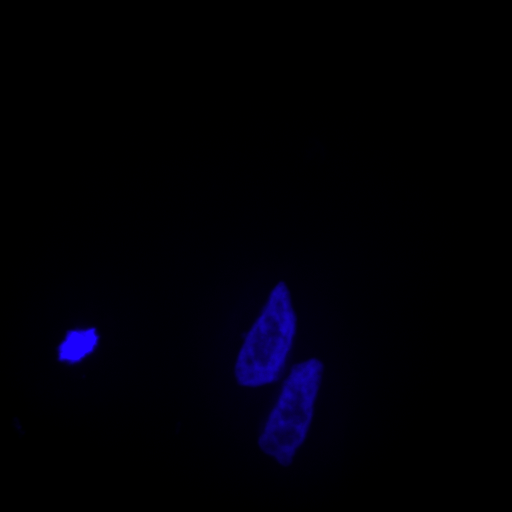

Supplement: Source data 3. [file elife-70700-data3.zip › Figure source data/Figure 3-source data 1/Figure 3-DMSO_DAPI.tif]

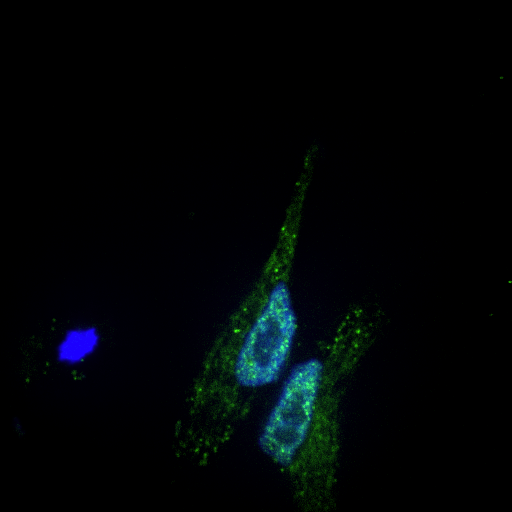

Supplement: Source data 3. [file elife-70700-data3.zip › Figure source data/Figure 3-source data 1/Figure 3-DMSO_Merge.tif]

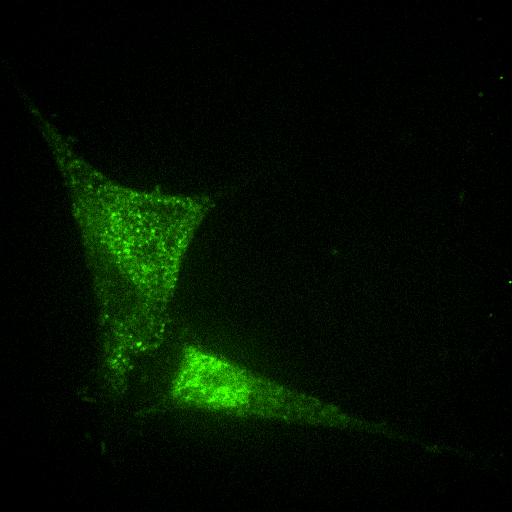

Supplement: Source data 3. [file elife-70700-data3.zip › Figure source data/Figure 3-source data 1/Figure 3-ENZa_AR.tif]

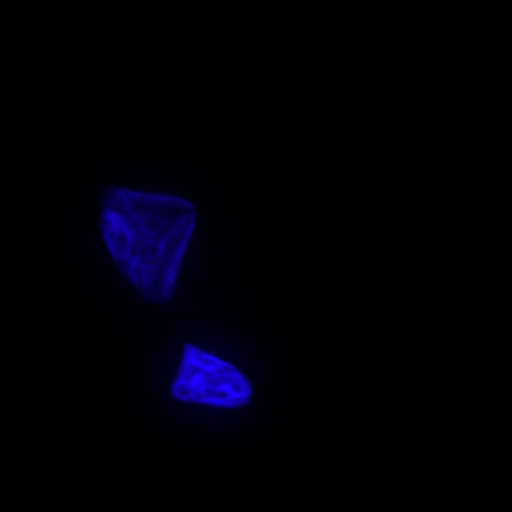

Supplement: Source data 3. [file elife-70700-data3.zip › Figure source data/Figure 3-source data 1/Figure 3-ENZa_DAPI.tif]

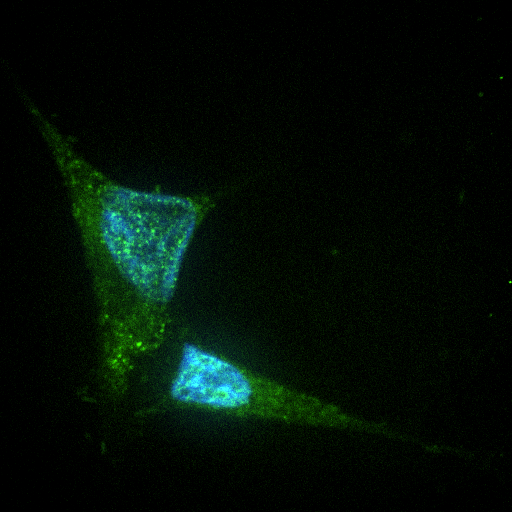

Supplement: Source data 3. [file elife-70700-data3.zip › Figure source data/Figure 3-source data 1/Figure 3-ENZa_Merge.tif]

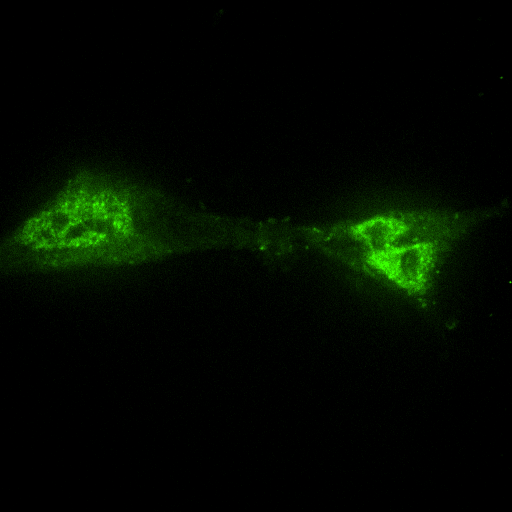

Supplement: Source data 3. [file elife-70700-data3.zip › Figure source data/Figure 3-source data 1/Figure 3-Z15_AR.tif]

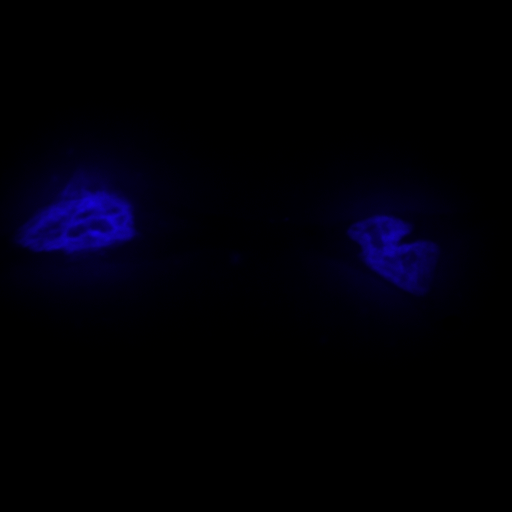

Supplement: Source data 3. [file elife-70700-data3.zip › Figure source data/Figure 3-source data 1/Figure 3-Z15_DAPI.tif]

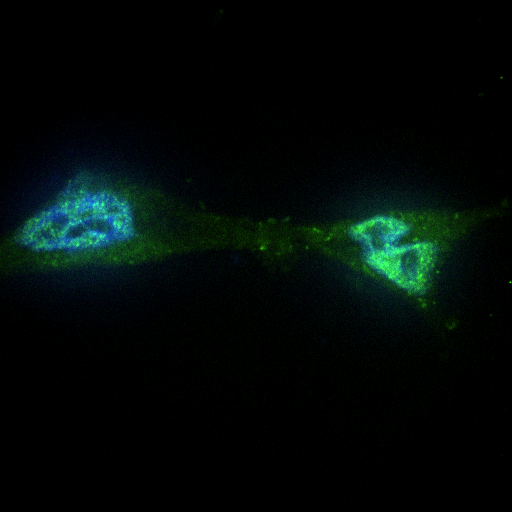

Supplement: Source data 3. [file elife-70700-data3.zip › Figure source data/Figure 3-source data 1/Figure 3-Z15_Merge.tif]

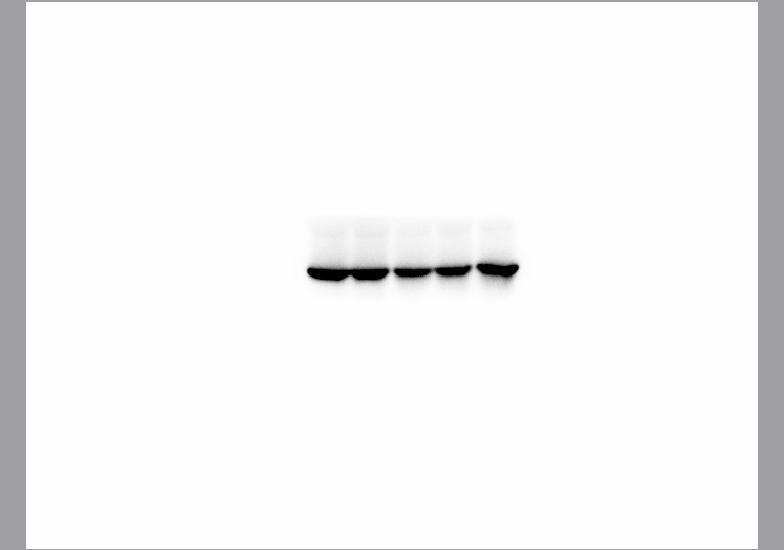

Supplement: Source data 3. [file elife-70700-data3.zip › Figure source data/Figure 5-source data 1/Figure 5A/Figure 5A-actin.jpg]

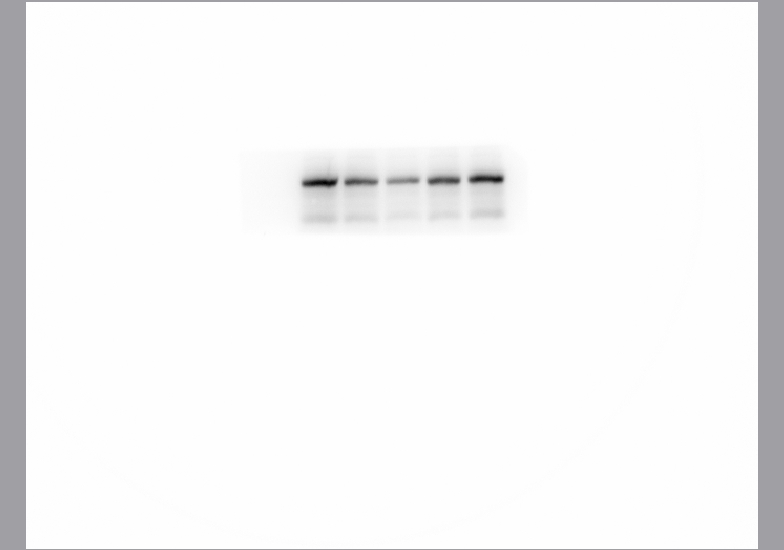

Supplement: Source data 3. [file elife-70700-data3.zip › Figure source data/Figure 5-source data 1/Figure 5A/Figure 5A-AR.jpg]

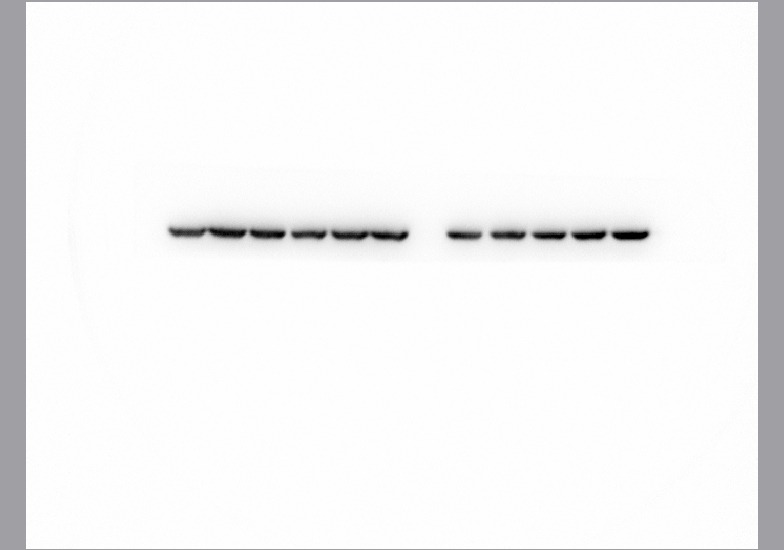

Supplement: Source data 3. [file elife-70700-data3.zip › Figure source data/Figure 5-source data 1/Figure 5B/Figure 5B-actin.jpg]

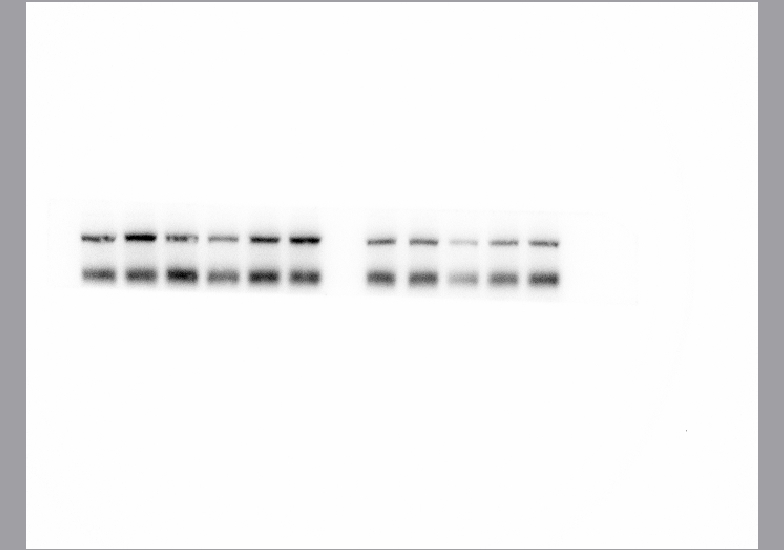

Supplement: Source data 3. [file elife-70700-data3.zip › Figure source data/Figure 5-source data 1/Figure 5B/Figure 5B-AR.jpg]

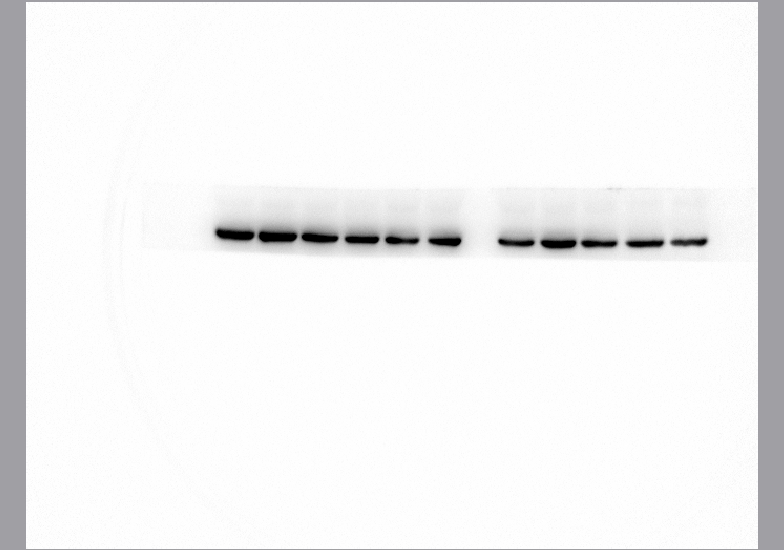

Supplement: Source data 3. [file elife-70700-data3.zip › Figure source data/Figure 5-source data 1/Figure 5C/Figure 5C-actin.jpg]

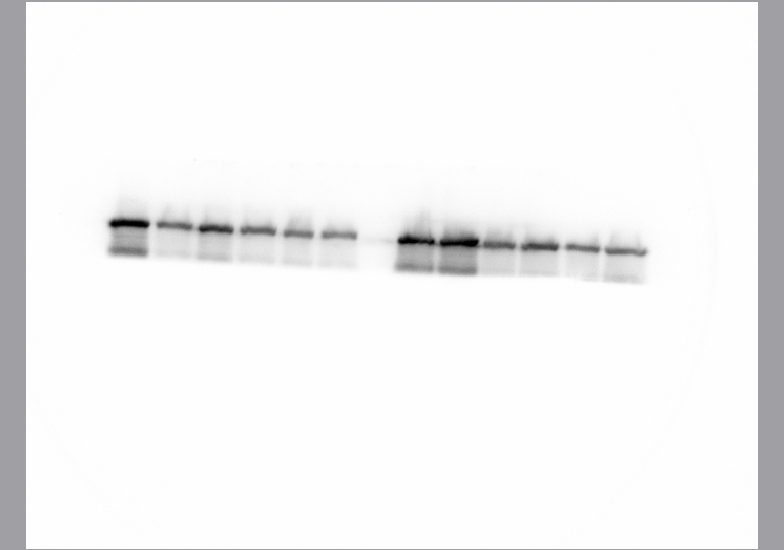

Supplement: Source data 3. [file elife-70700-data3.zip › Figure source data/Figure 5-source data 1/Figure 5C/Figure 5C-AR.jpg]

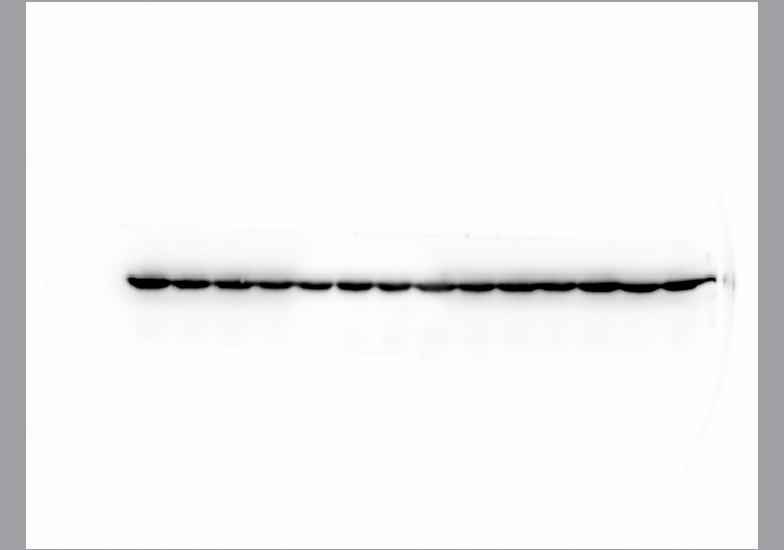

Supplement: Source data 3. [file elife-70700-data3.zip › Figure source data/Figure 5-source data 1/Figure 5D/Figure 5D-actin.jpg]

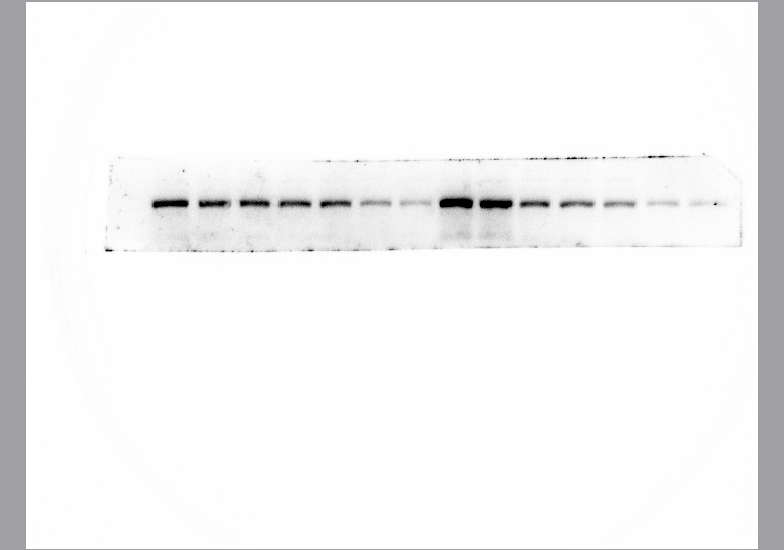

Supplement: Source data 3. [file elife-70700-data3.zip › Figure source data/Figure 5-source data 1/Figure 5D/Figure 5D-AR.jpg]

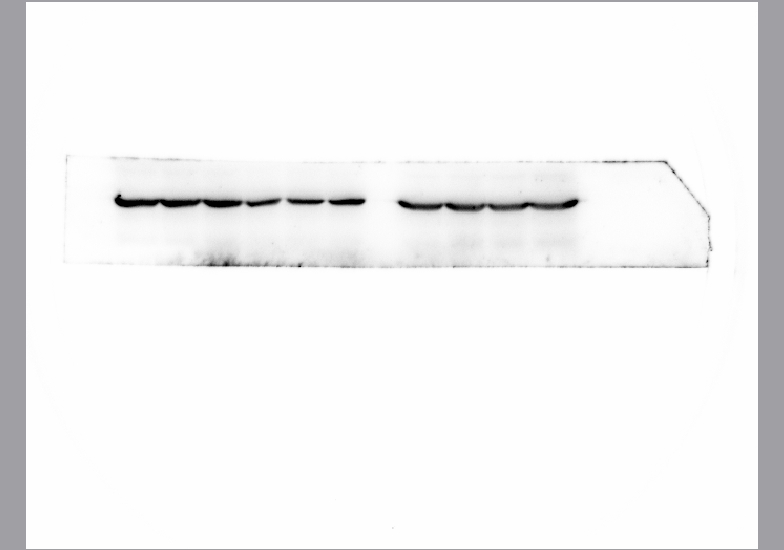

Supplement: Source data 3. [file elife-70700-data3.zip › Figure source data/Figure 5-source data 1/Figure 5E/Figure 5E-actin_LNCaP.jpg]

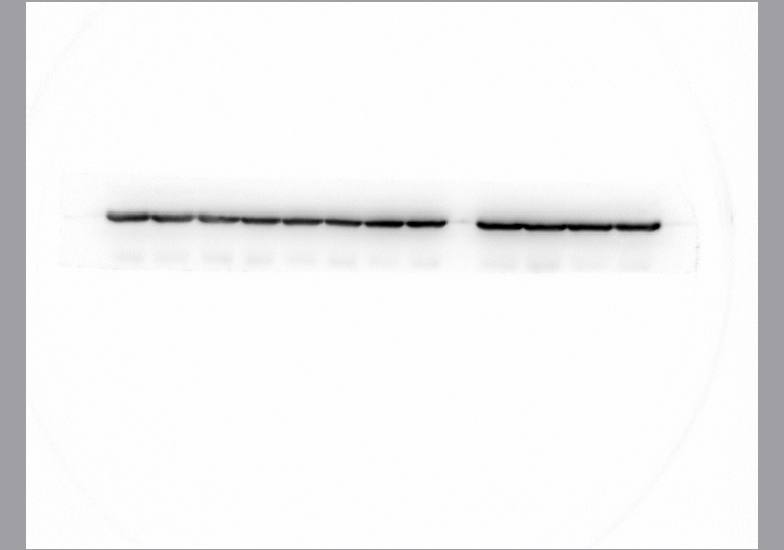

Supplement: Source data 3. [file elife-70700-data3.zip › Figure source data/Figure 5-source data 1/Figure 5E/Figure 5E-actin_VCaP.jpg]

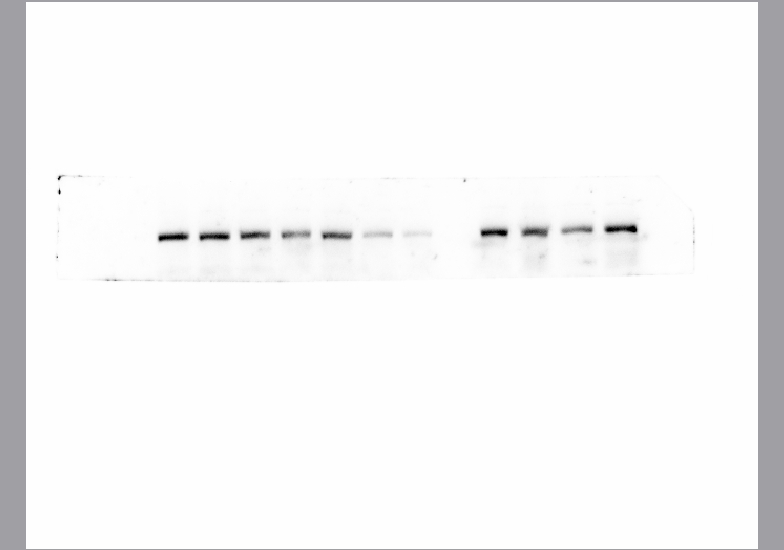

Supplement: Source data 3. [file elife-70700-data3.zip › Figure source data/Figure 5-source data 1/Figure 5E/Figure 5E-AR_LNCaP.jpg]

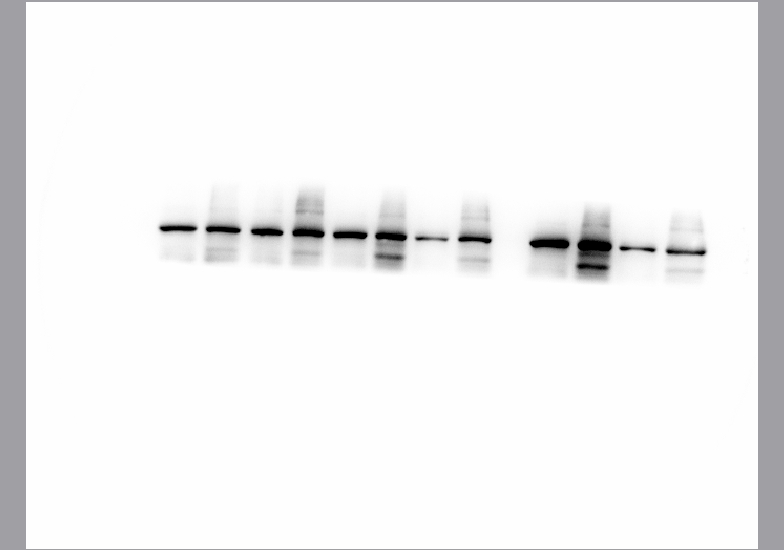

Supplement: Source data 3. [file elife-70700-data3.zip › Figure source data/Figure 5-source data 1/Figure 5E/Figure 5E-AR_VCaP.jpg]

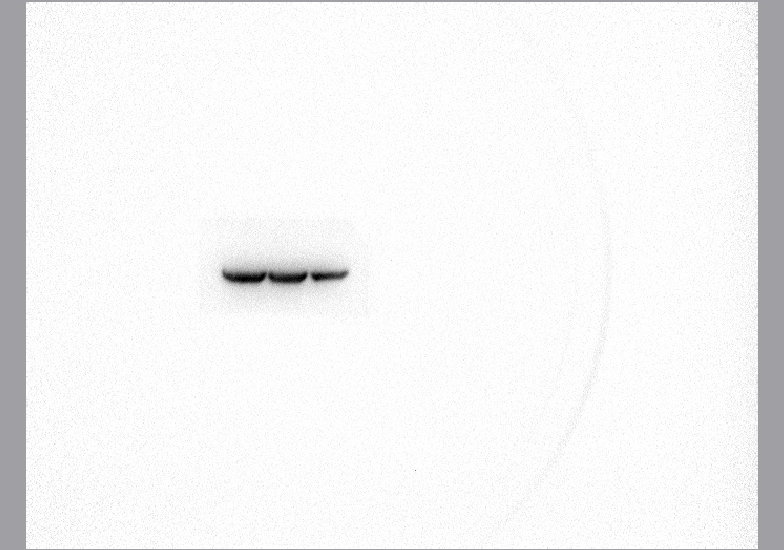

Supplement: Source data 3. [file elife-70700-data3.zip › Figure source data/Figure 5-source data 1/Figure 5F/Figure 5F-actin.jpg]

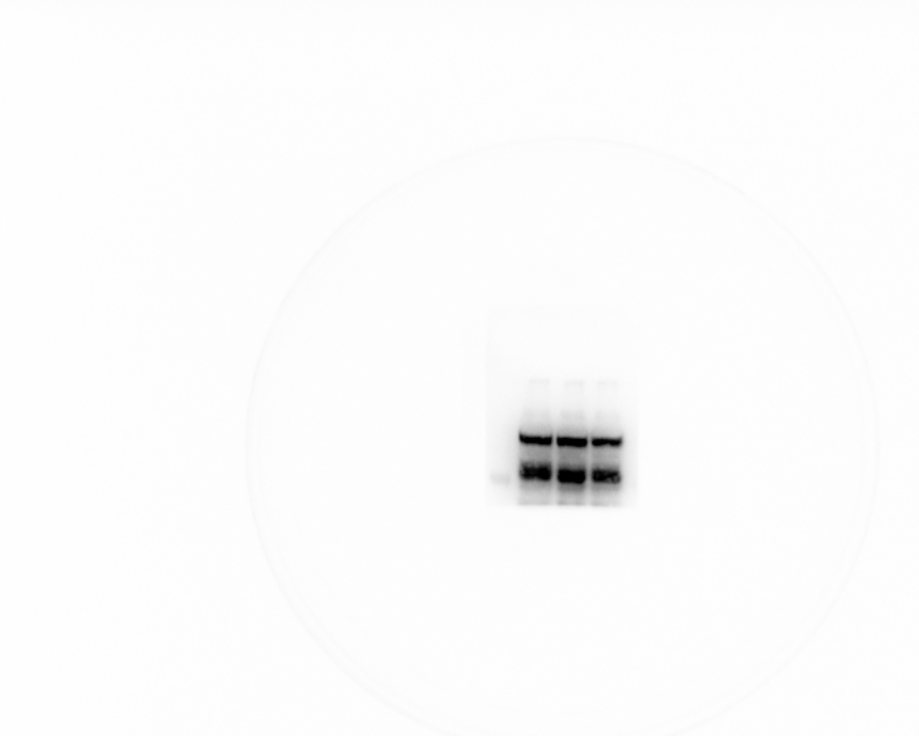

Supplement: Source data 3. [file elife-70700-data3.zip › Figure source data/Figure 5-source data 1/Figure 5F/Figure 5F-AR.tif]

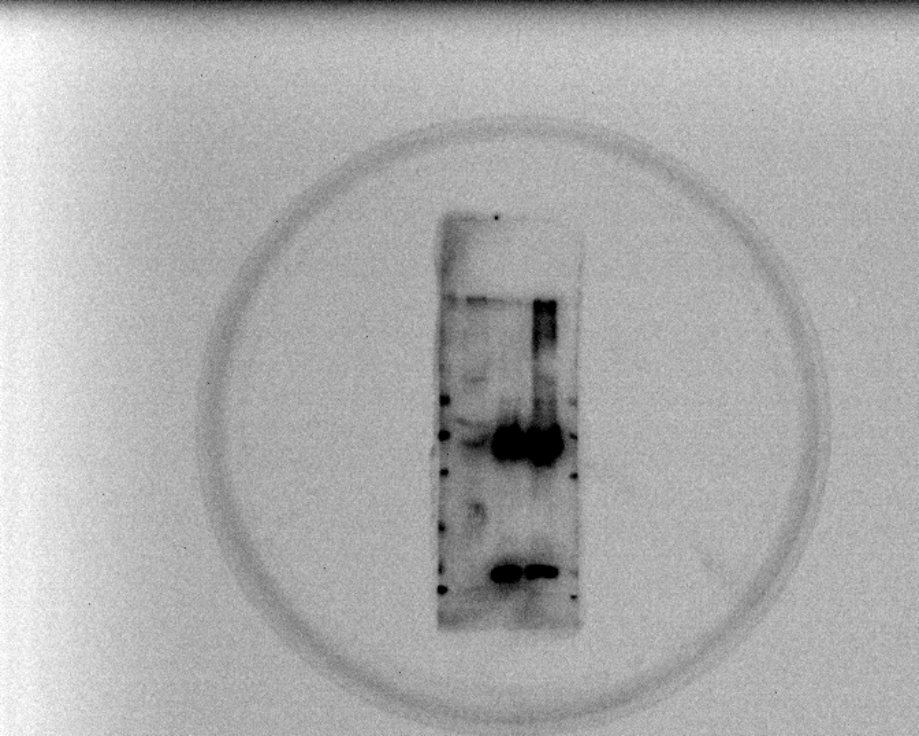

Supplement: Source data 3. [file elife-70700-data3.zip › Figure source data/Figure 5-source data 1/Figure 5F/Figure 5F-UB-MYC.tif]

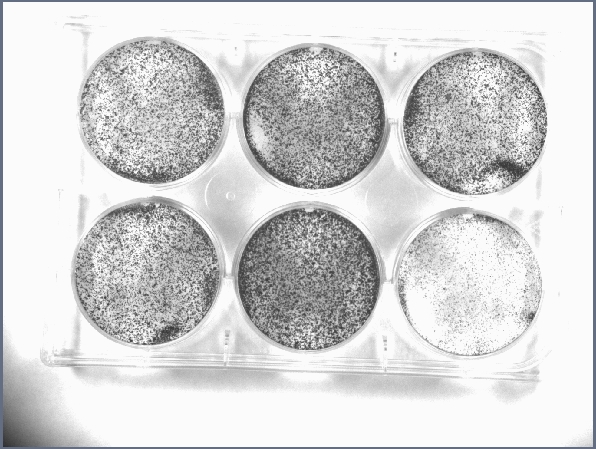

Supplement: Source data 3. [file elife-70700-data3.zip › Figure source data/Figure 6-source data 1/Figure 6B/Figure 6B-22Rv1.jpg]

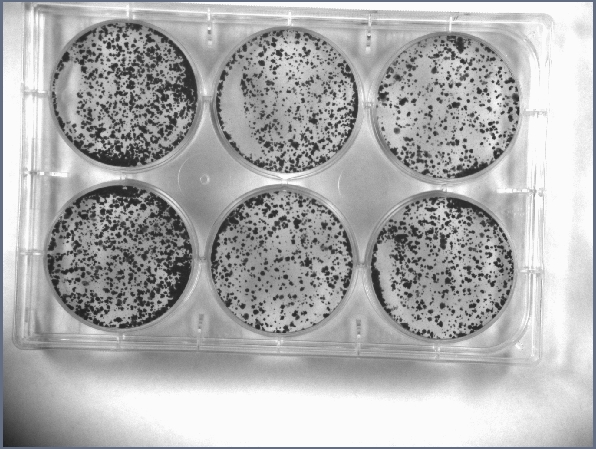

Supplement: Source data 3. [file elife-70700-data3.zip › Figure source data/Figure 6-source data 1/Figure 6B/Figure 6B-PC3.jpg]

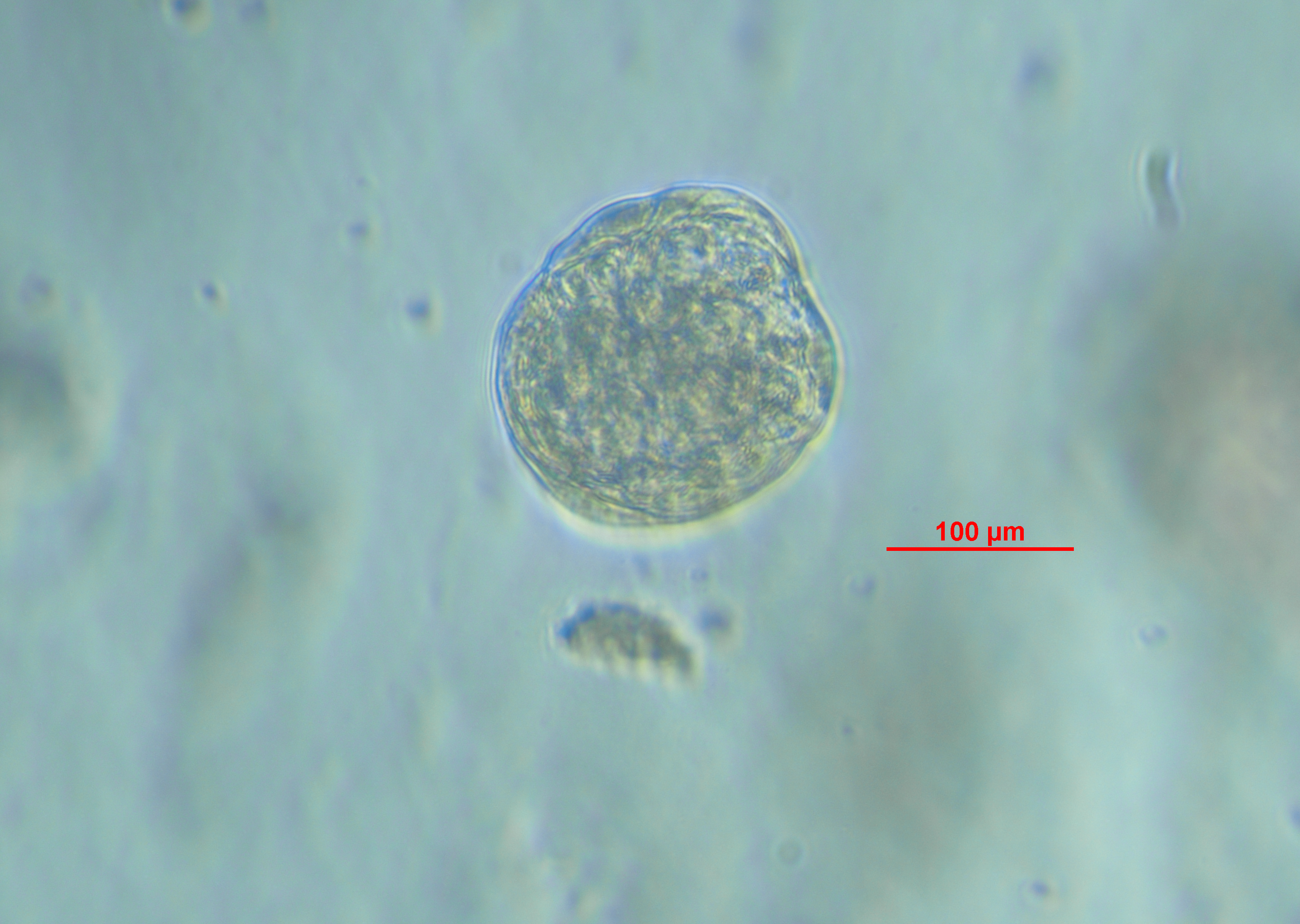

Supplement: Source data 3. [file elife-70700-data3.zip › Figure source data/Figure 6-source data 1/Figure 6C/Figure 6C-DMSO.tif]

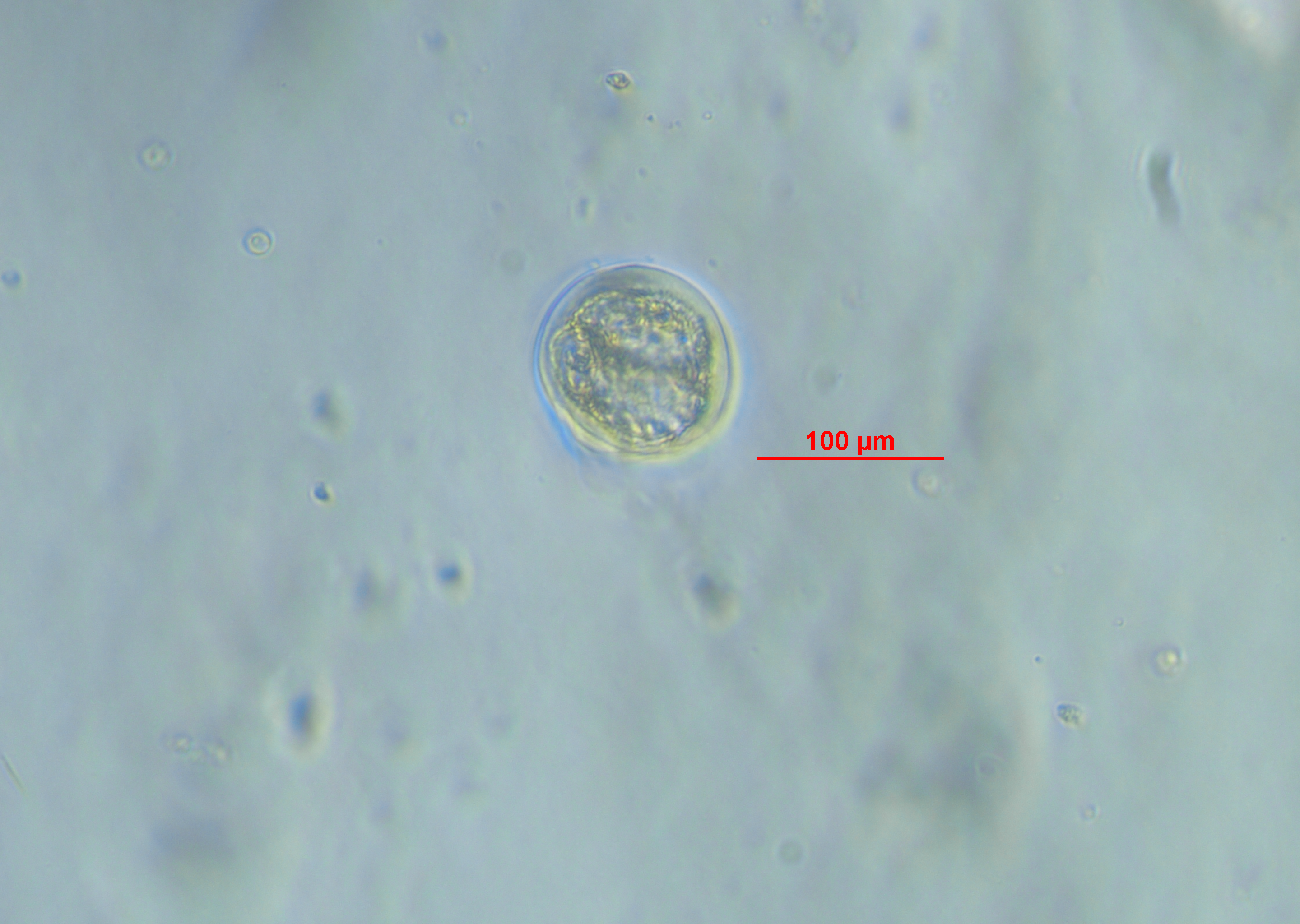

Supplement: Source data 3. [file elife-70700-data3.zip › Figure source data/Figure 6-source data 1/Figure 6C/Figure 6C-Z15.tif]

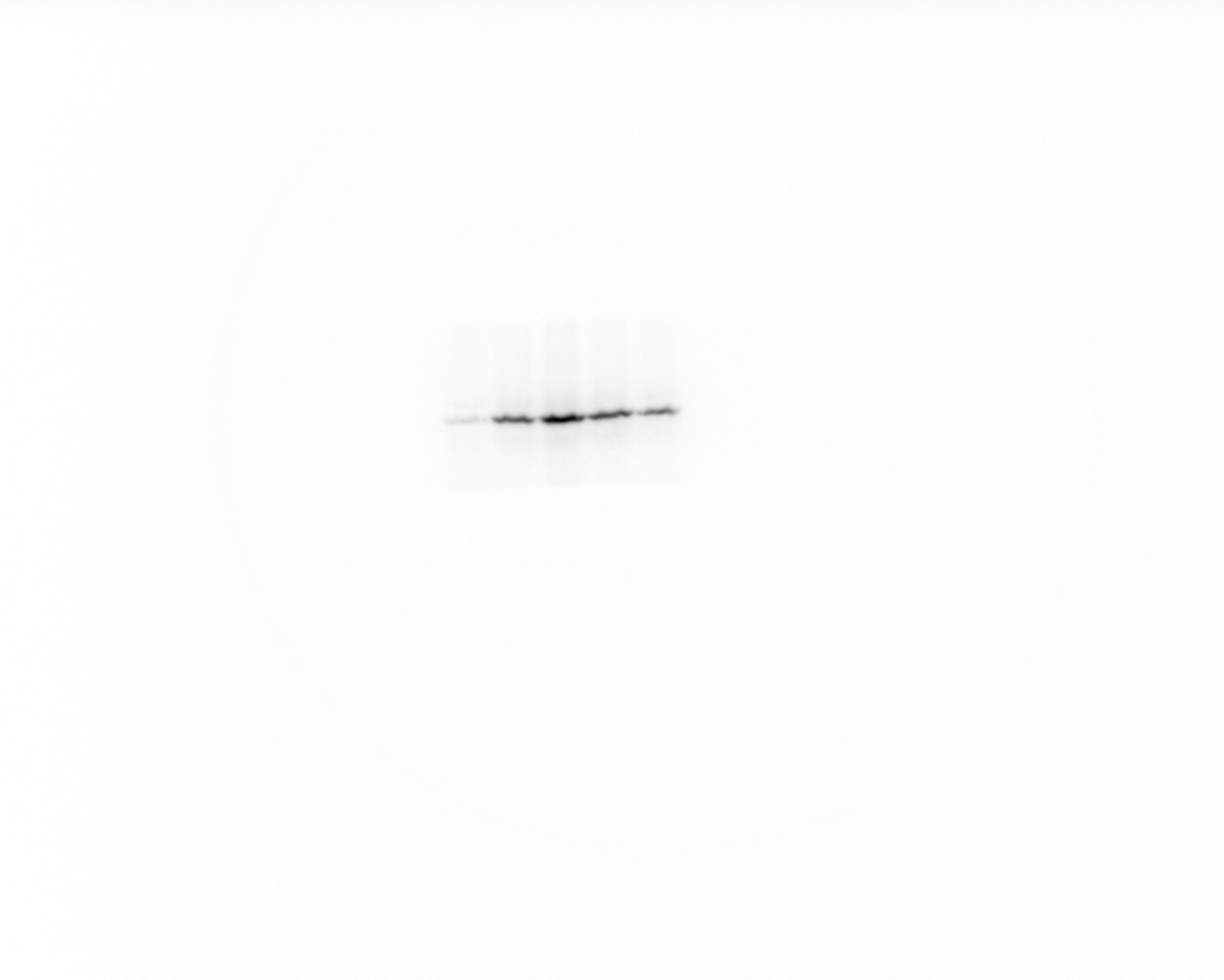

Supplement: Source data 3. [file elife-70700-data3.zip › Figure source data/Figure 6-source data 1/Figure 6D/Figure 6D-actin-.tif]

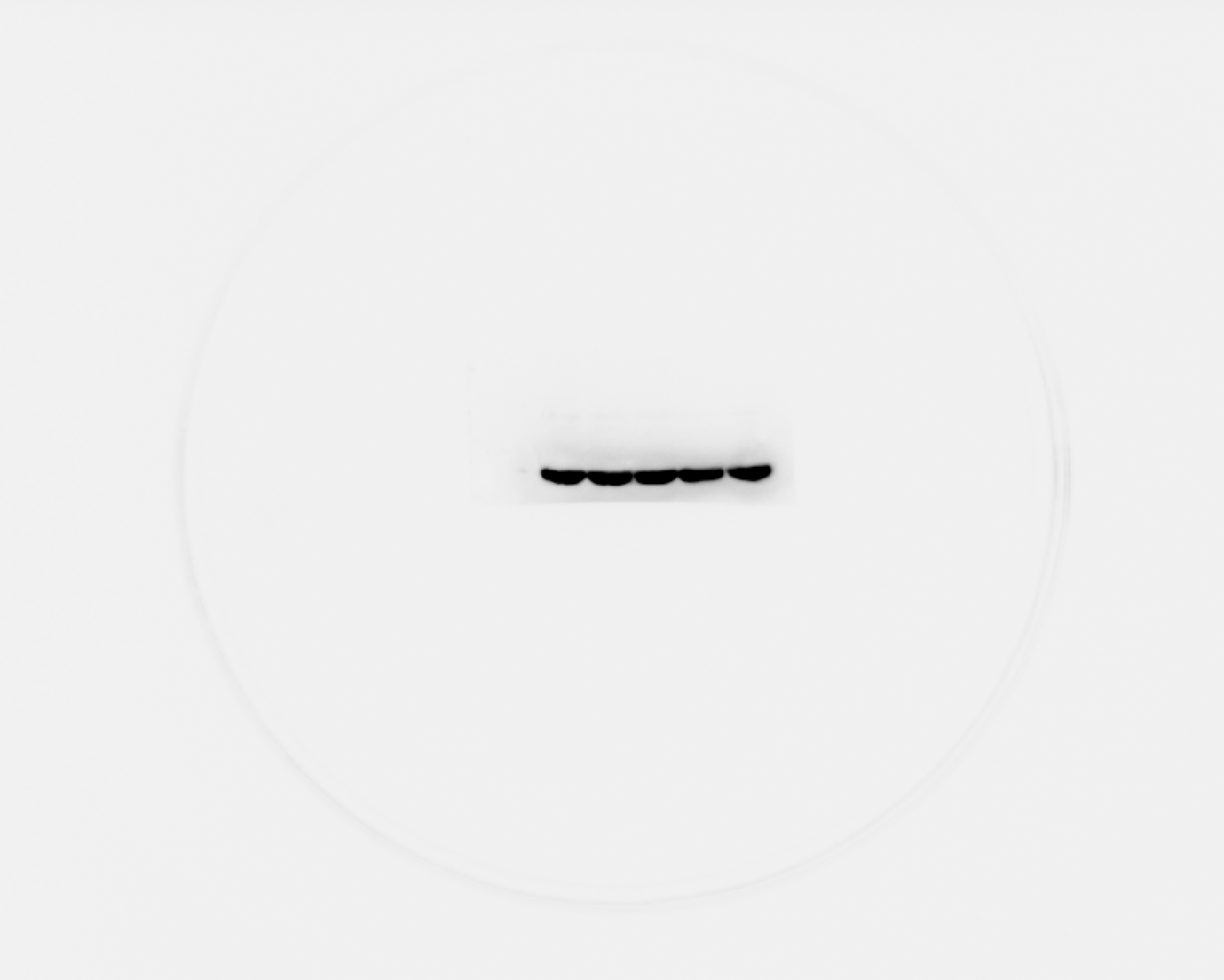

Supplement: Source data 3. [file elife-70700-data3.zip › Figure source data/Figure 6-source data 1/Figure 6D/Figure 6D-Cleaved Parp-.tif]

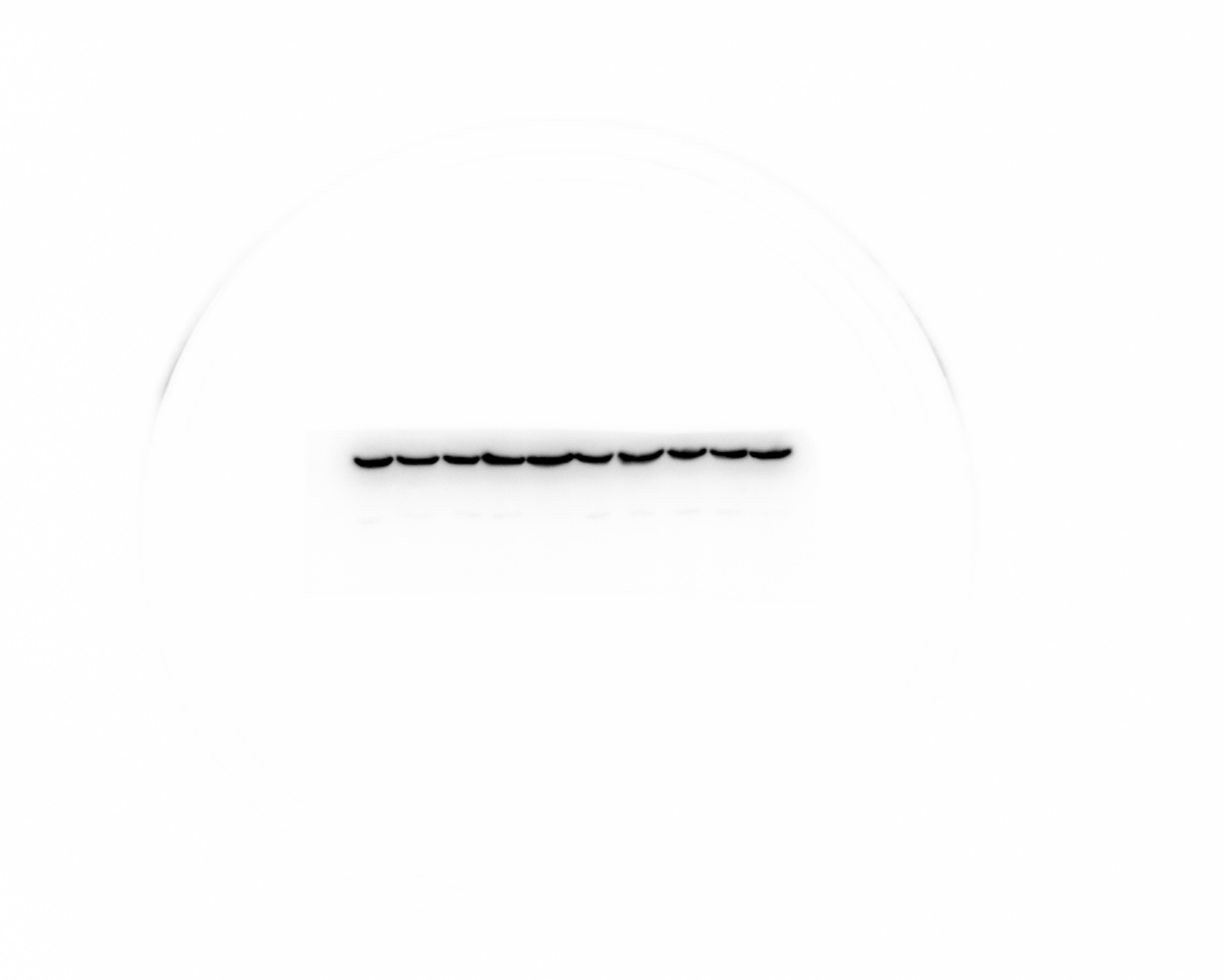

Supplement: Source data 3. [file elife-70700-data3.zip › Figure source data/Figure 6-source data 1/Figure 6E/Figure 6E-actin.tif]

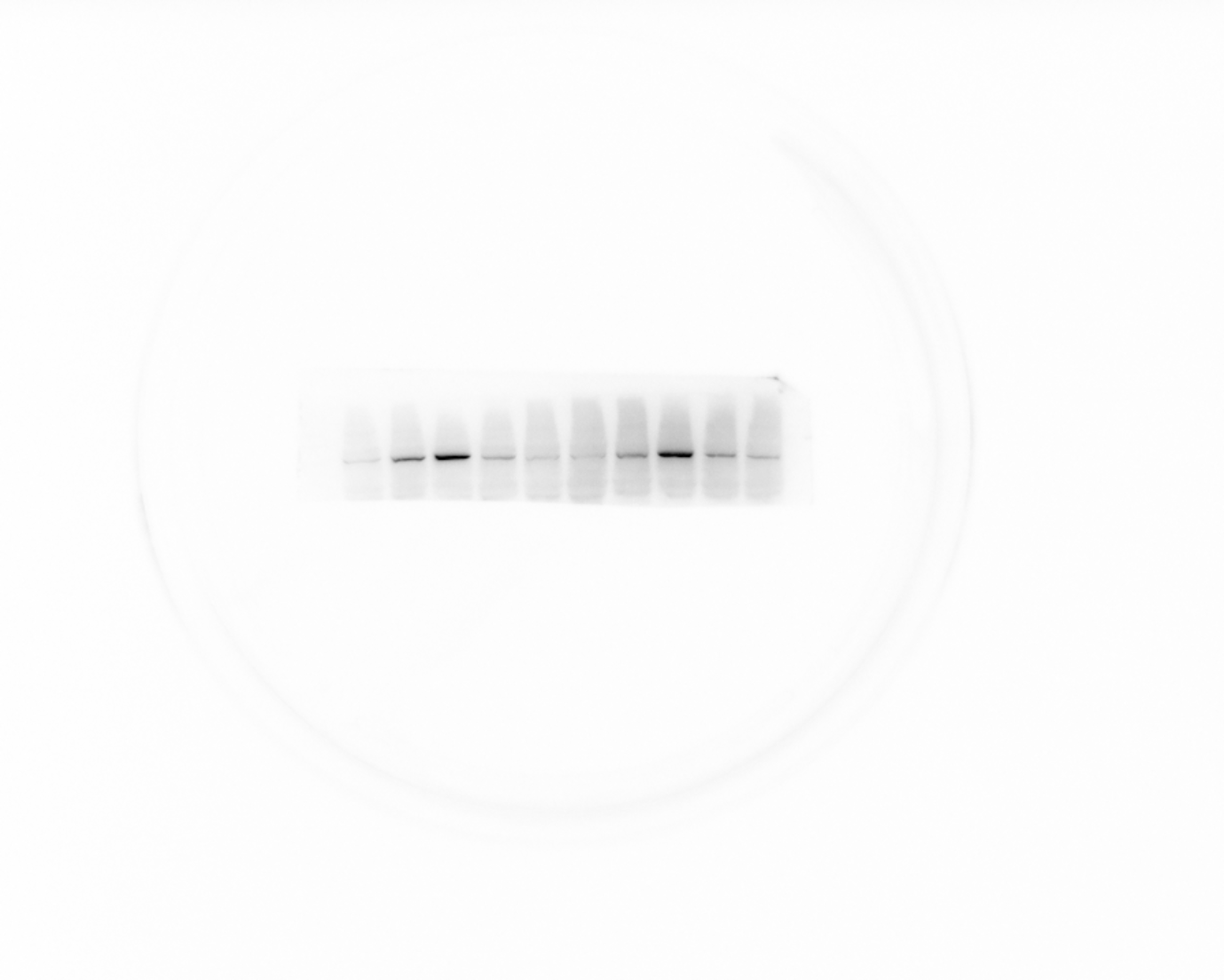

Supplement: Source data 3. [file elife-70700-data3.zip › Figure source data/Figure 6-source data 1/Figure 6E/Figure 6E-Cleaved PARP.tif]

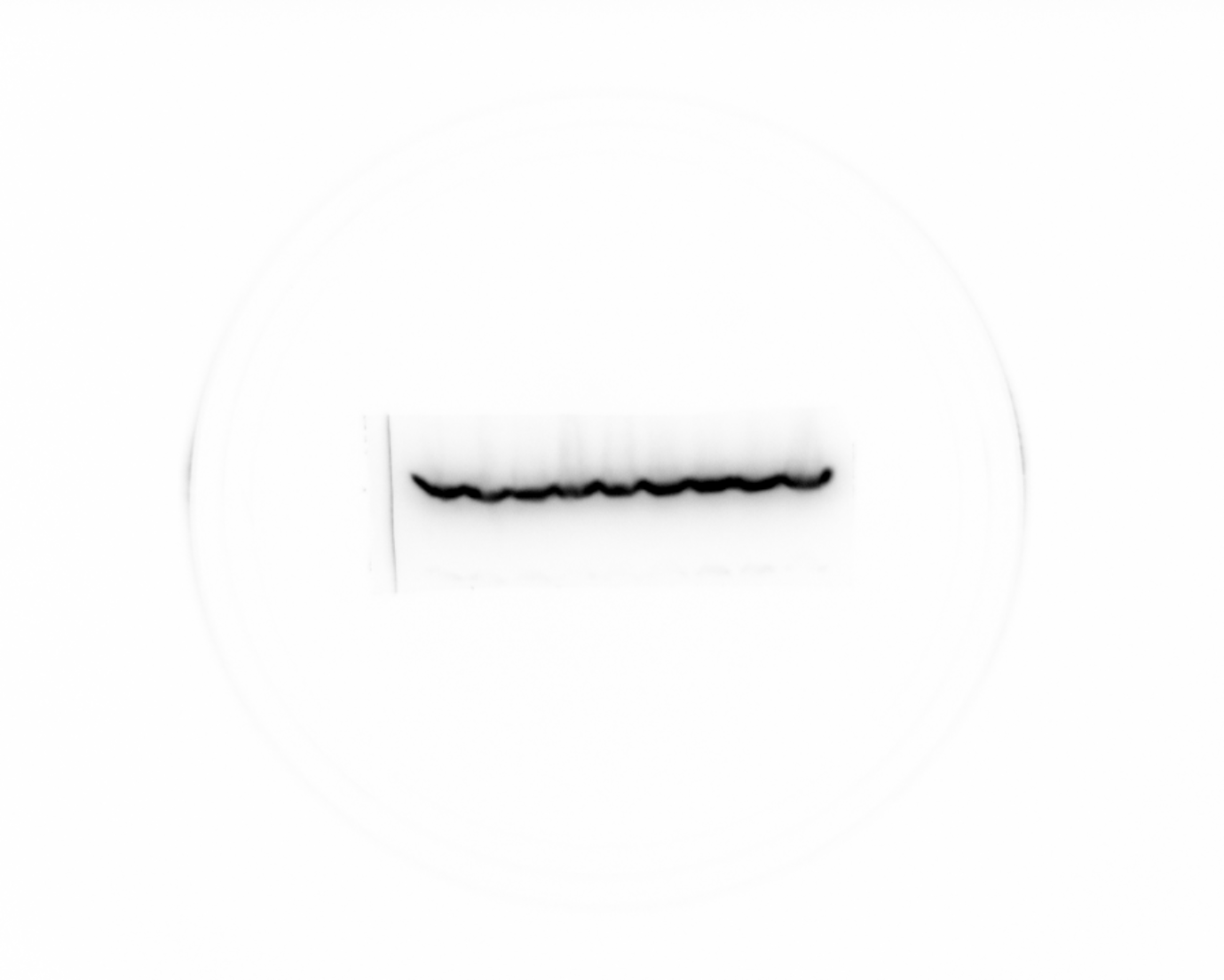

Supplement: Source data 3. [file elife-70700-data3.zip › Figure source data/Figure 7-source data 1/Figure 7D/Figure 7D-actin.tif]

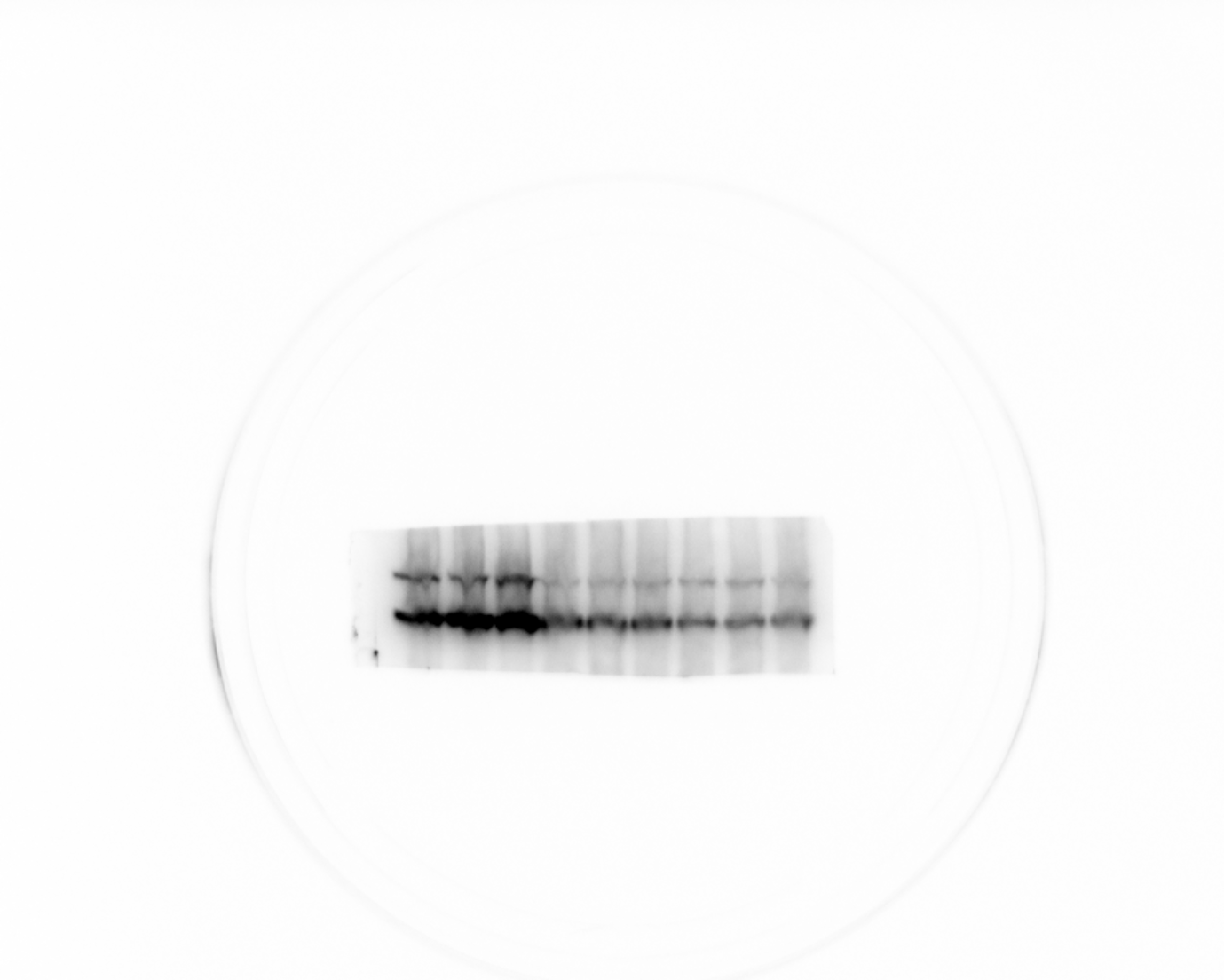

Supplement: Source data 3. [file elife-70700-data3.zip › Figure source data/Figure 7-source data 1/Figure 7D/Figure 7D-AR.tif]

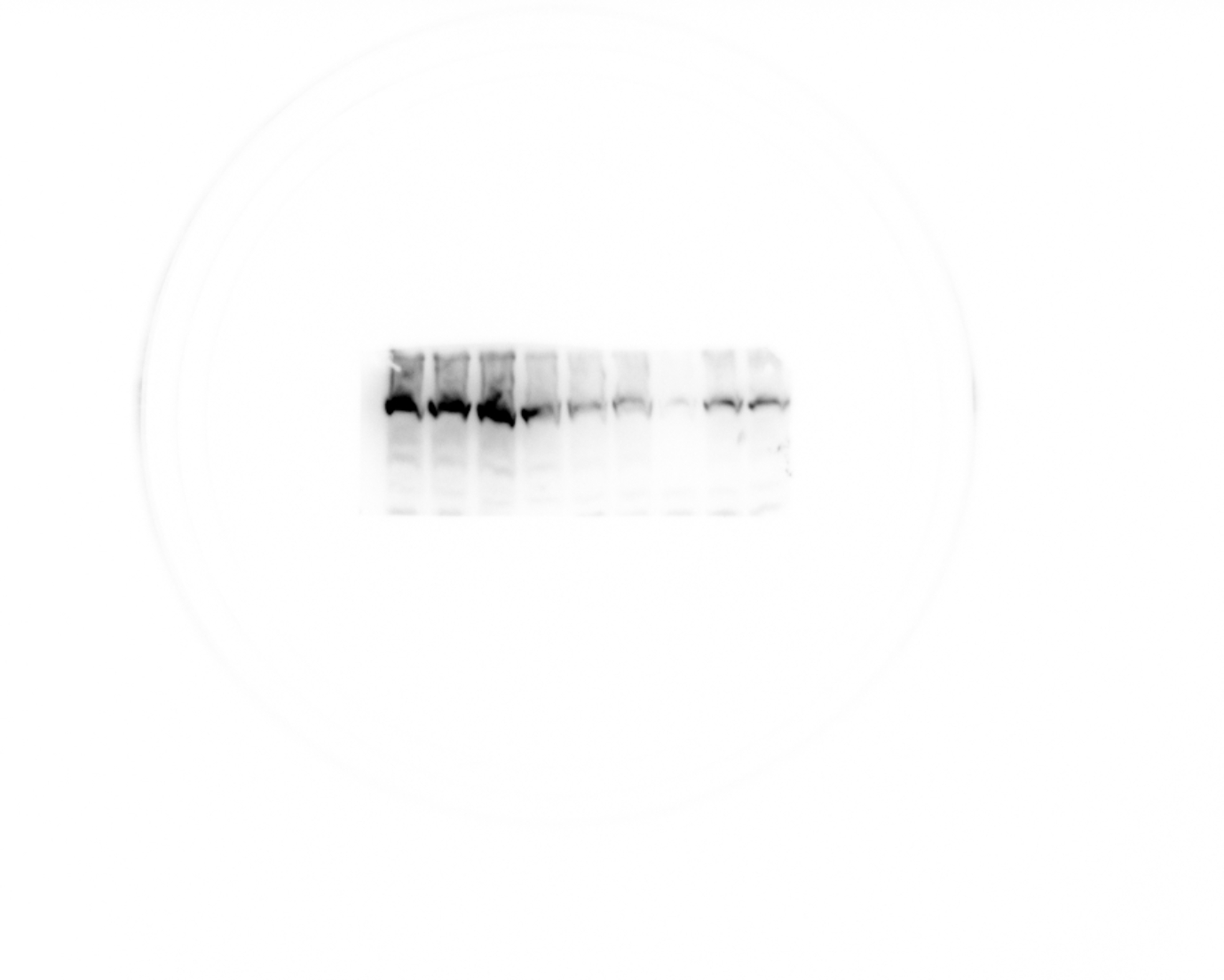

Supplement: Source data 3. [file elife-70700-data3.zip › Figure source data/Figure 7-source data 1/Figure 7D/Figure 7D-PSA.jpg]

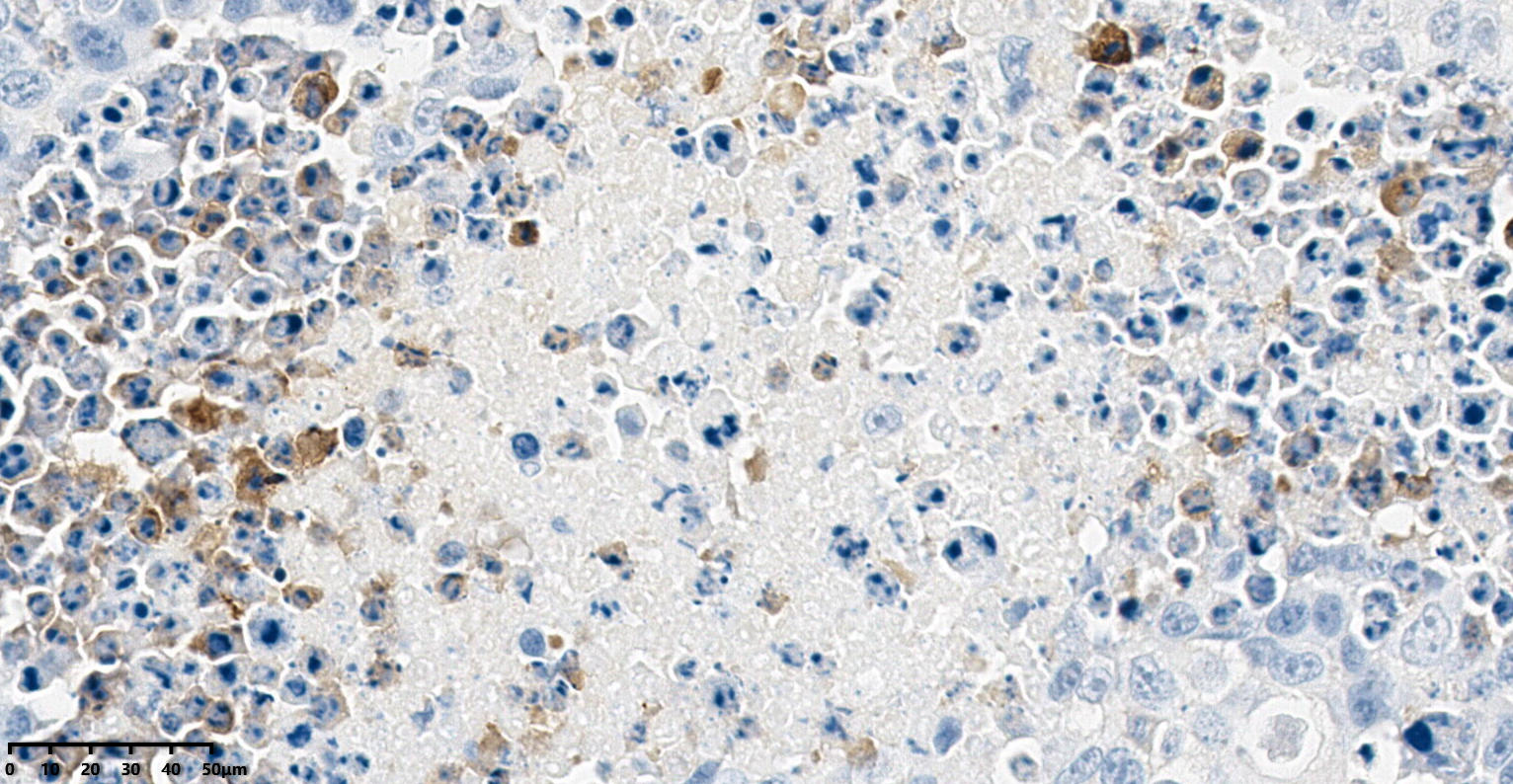

Supplement: Source data 3. [file elife-70700-data3.zip › Figure source data/Figure 7-source data 1/Figure 7E/Figure 7E-Ki-67-Control.tiff]

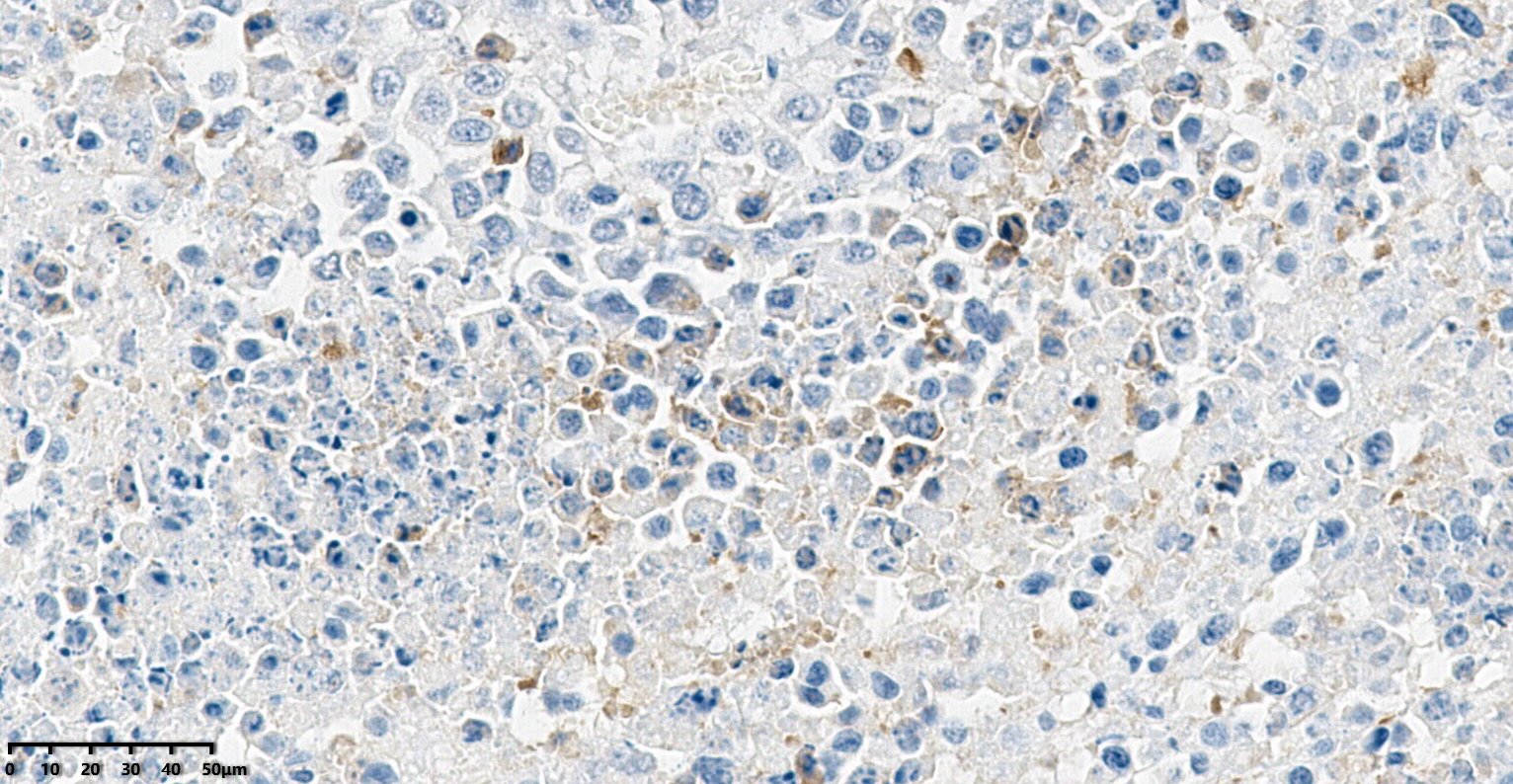

Supplement: Source data 3. [file elife-70700-data3.zip › Figure source data/Figure 7-source data 1/Figure 7E/Figure 7E-Ki-67-Z15 10mg.tiff]

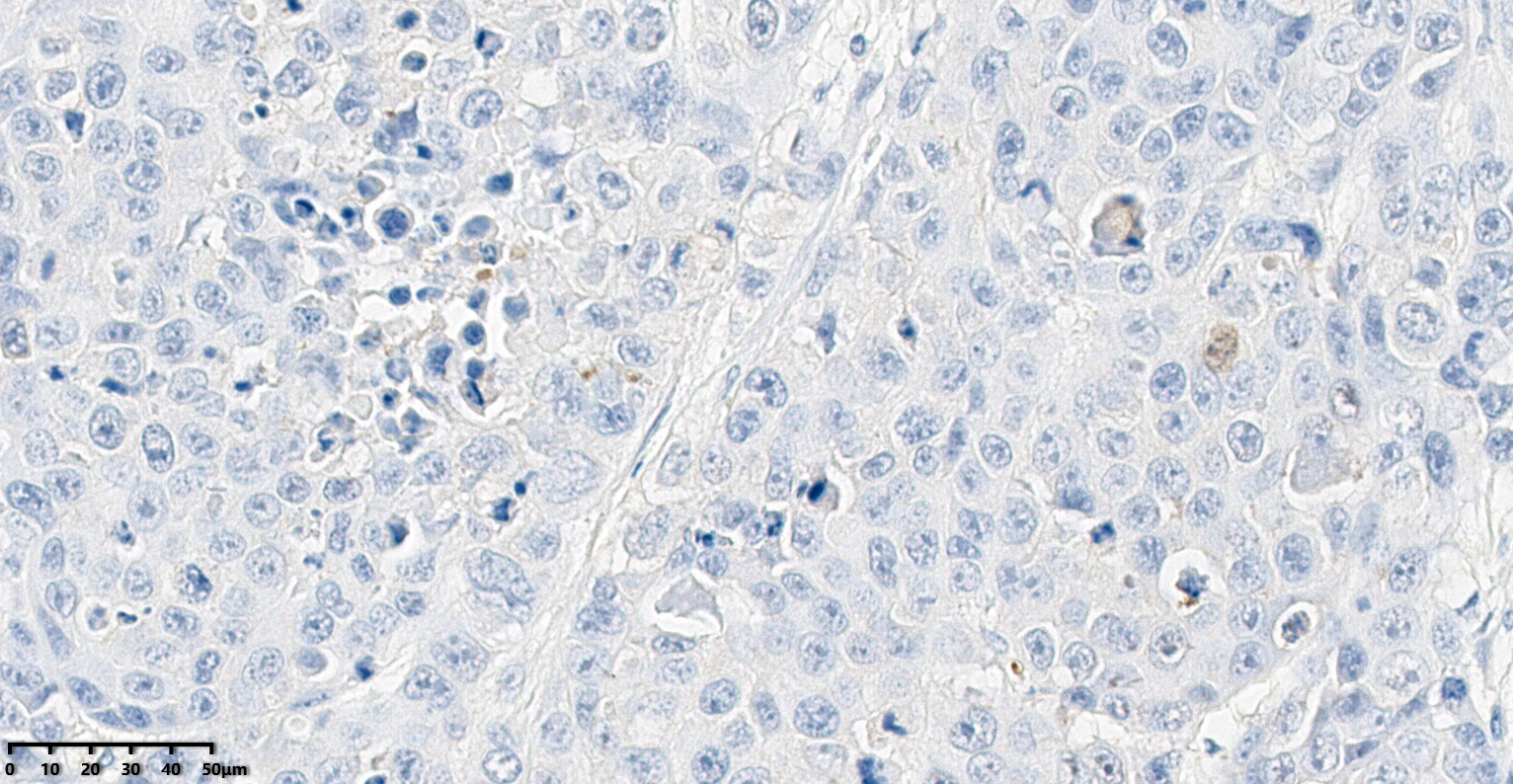

Supplement: Source data 3. [file elife-70700-data3.zip › Figure source data/Figure 7-source data 1/Figure 7E/Figure 7E-Ki-67-Z15 20mg.tiff]

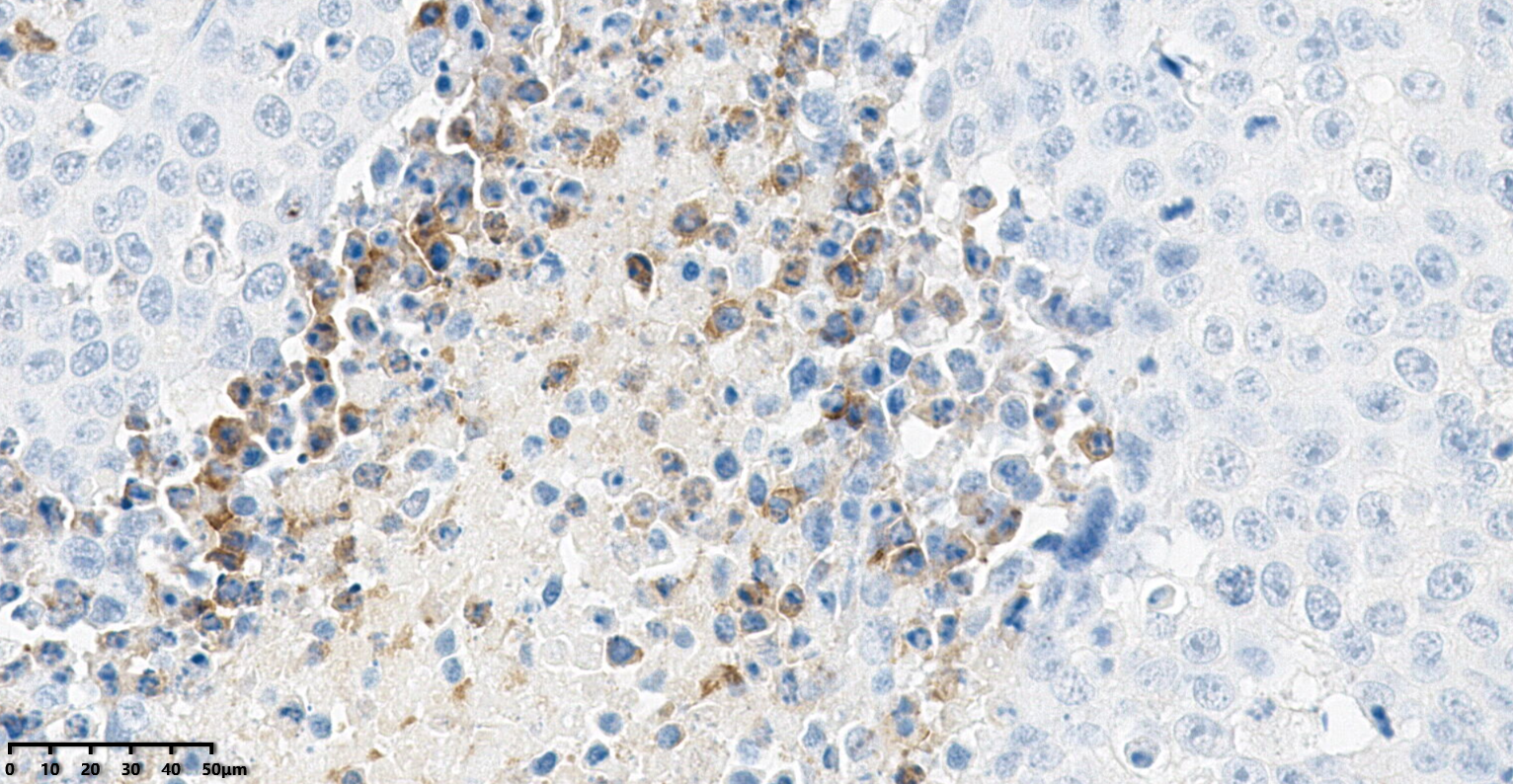

Supplement: Source data 3. [file elife-70700-data3.zip › Figure source data/Figure 7-source data 1/Figure 7E/Figure 7E-PSA-Control.tiff]

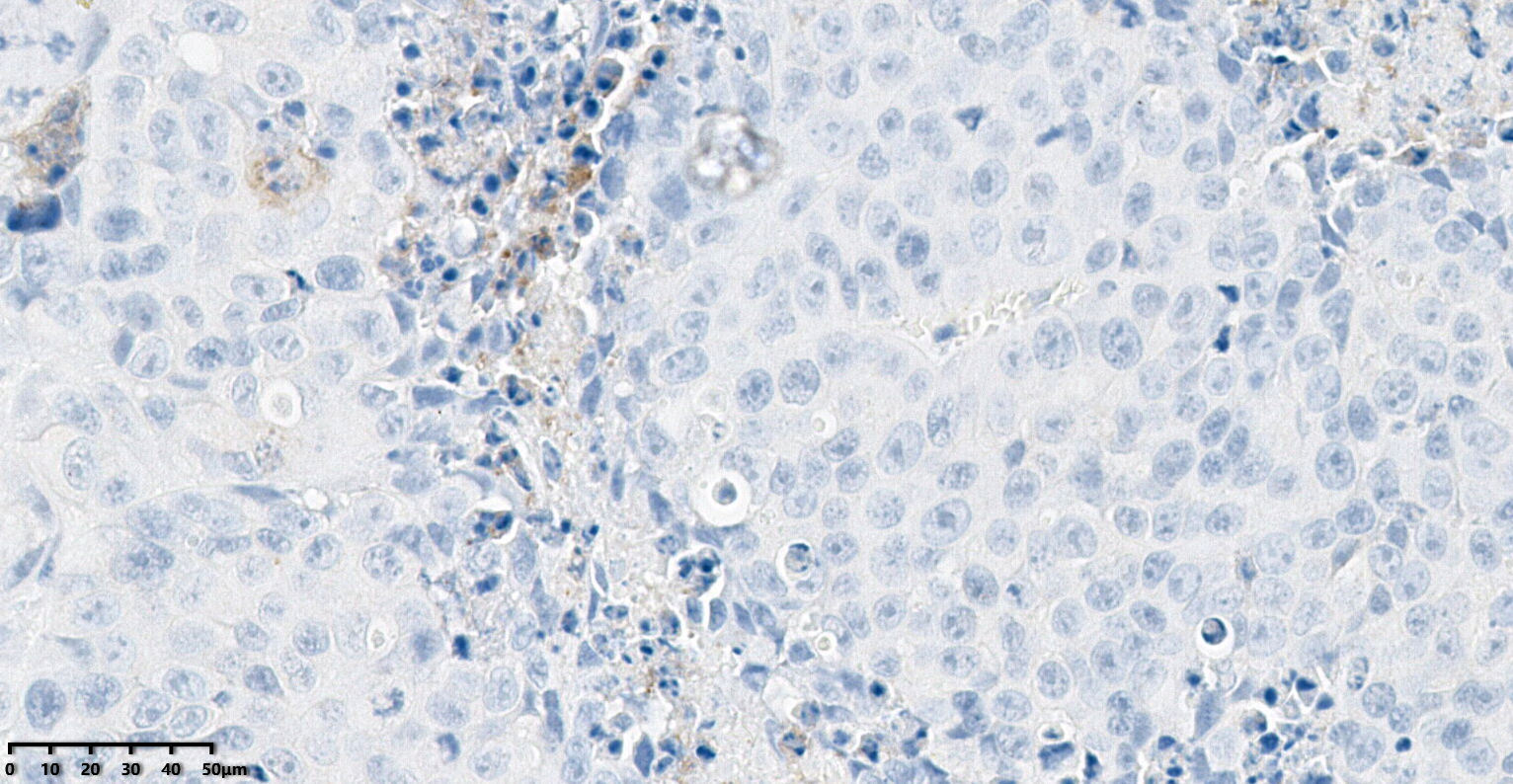

Supplement: Source data 3. [file elife-70700-data3.zip › Figure source data/Figure 7-source data 1/Figure 7E/Figure 7E-PSA-Z15 10mg.tiff]

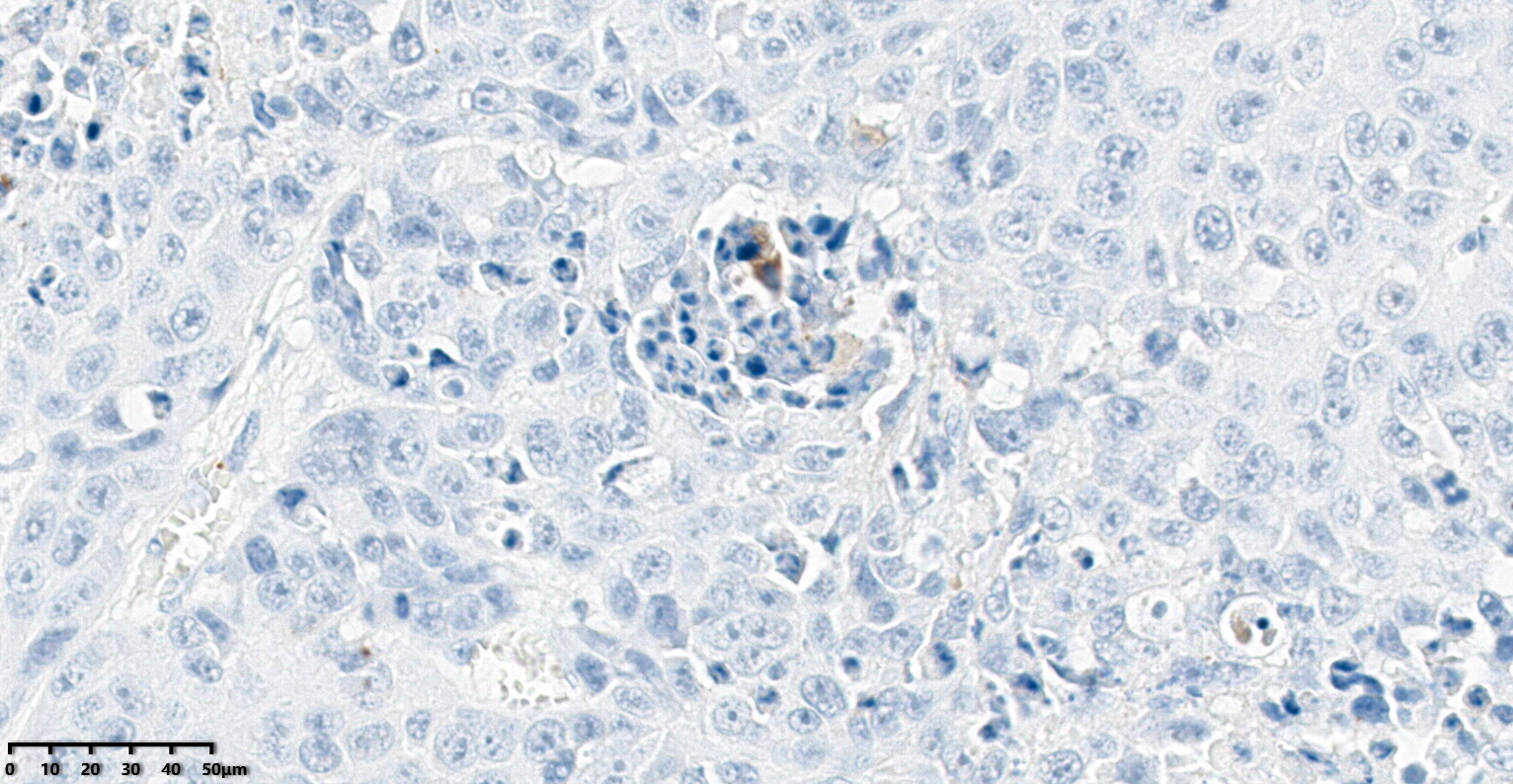

Supplement: Source data 3. [file elife-70700-data3.zip › Figure source data/Figure 7-source data 1/Figure 7E/Figure 7E-PSA-Z15 20mg.tiff]

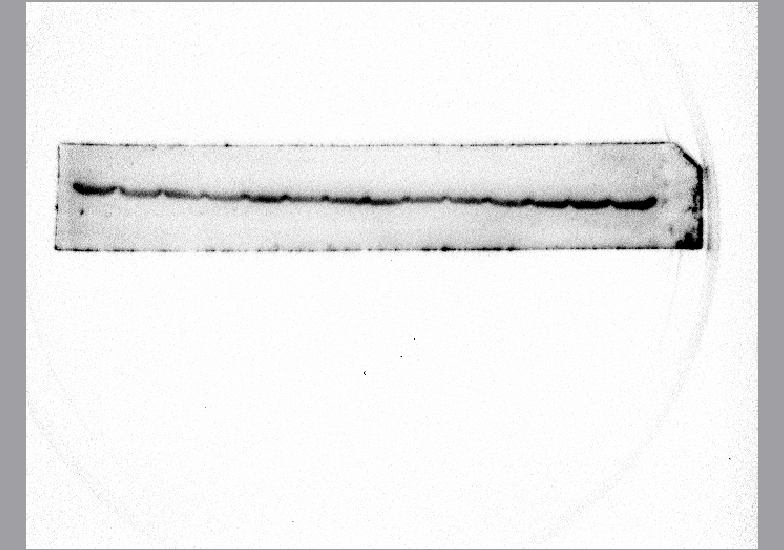

Supplement: Source data 3. [file elife-70700-data3.zip › Figure source data/Figure 8-source data 1/Figure 8C/actin-1.jpg]

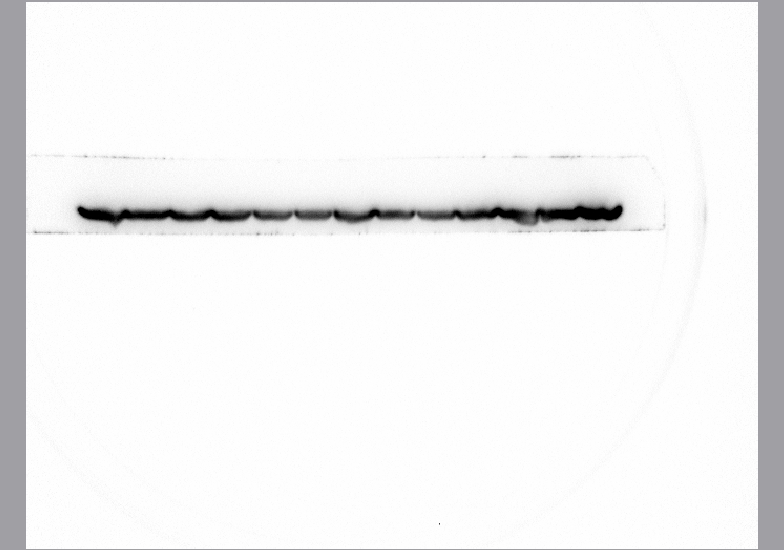

Supplement: Source data 3. [file elife-70700-data3.zip › Figure source data/Figure 8-source data 1/Figure 8C/actin-2.jpg]

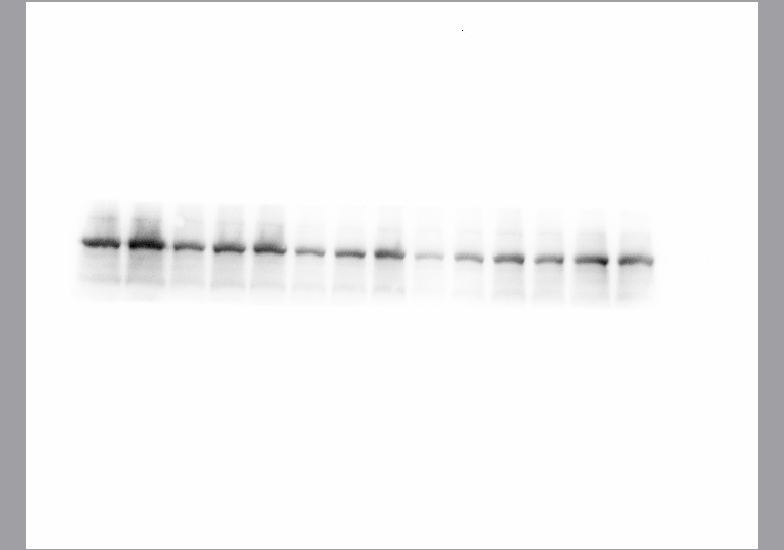

Supplement: Source data 3. [file elife-70700-data3.zip › Figure source data/Figure 8-source data 1/Figure 8C/AR-1.jpg]

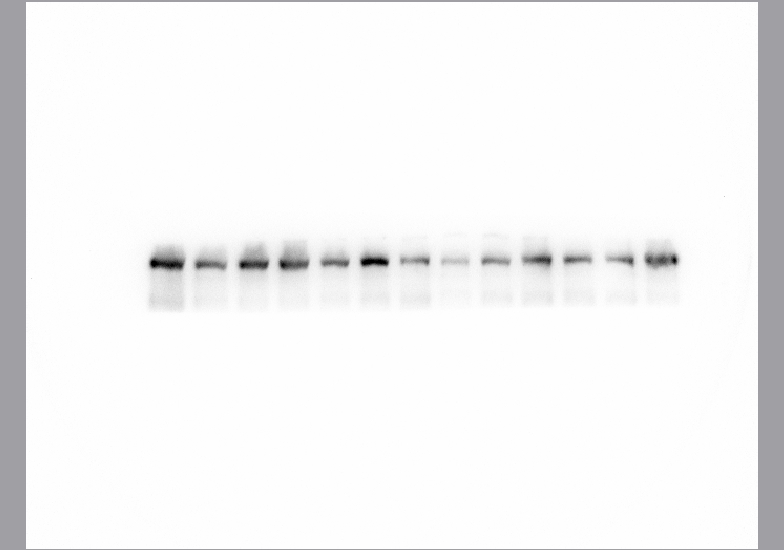

Supplement: Source data 3. [file elife-70700-data3.zip › Figure source data/Figure 8-source data 1/Figure 8C/AR-2.jpg]

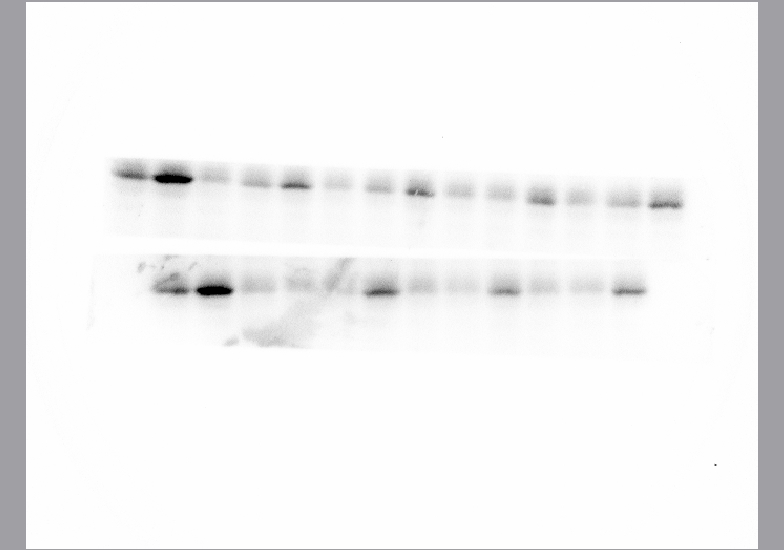

Supplement: Source data 3. [file elife-70700-data3.zip › Figure source data/Figure 8-source data 1/Figure 8C/PSA-1.jpg]
