# Supplementary figures and images for: Selective androgen receptor degrader (SARD) to overcome antiandrogen resistance in castration-resistant prostate cancer (part 2 of 2)
Source: eLife. 2023 Jan 19;12:e70700. doi: 10.7554/eLife.70700 (PMC9901937; doi:10.7554/eLife.70700)

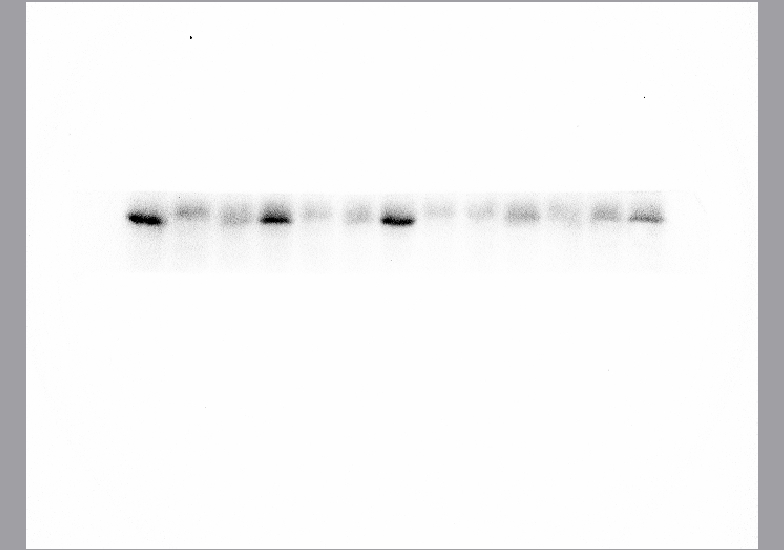

Supplement: Source data 3. [file elife-70700-data3.zip › Figure source data/Figure 8-source data 1/Figure 8C/PSA-2.jpg]
