# Supplementary material for: Selective androgen receptor degrader (SARD) to overcome antiandrogen resistance in castration-resistant prostate cancer
Source: eLife. 2023 Jan 19;12:e70700. doi: 10.7554/eLife.70700 (PMC9901937; doi:10.7554/eLife.70700)
Supplement: Source data 2. [file Z36.PDF › Supplementary Material_source_data/Figure 1-figure supplement 1 & Supplementary1a-source/Z36.PDF]

IVW36517

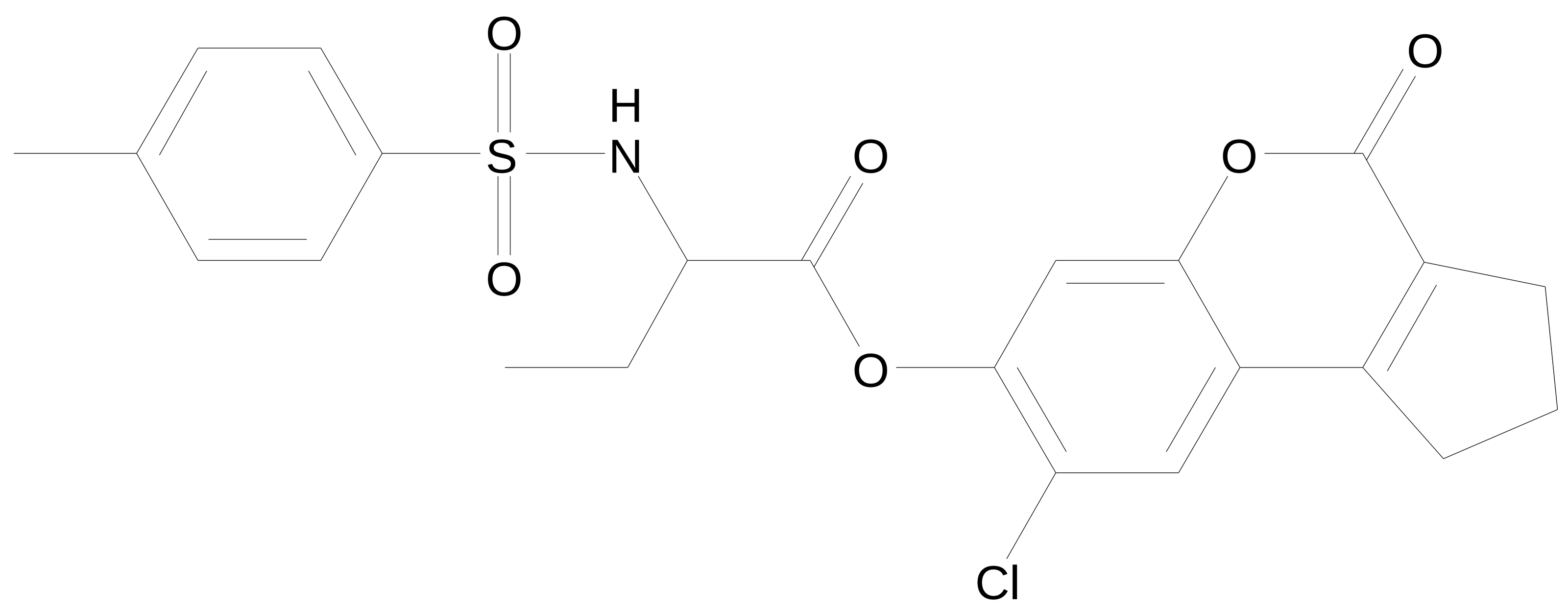

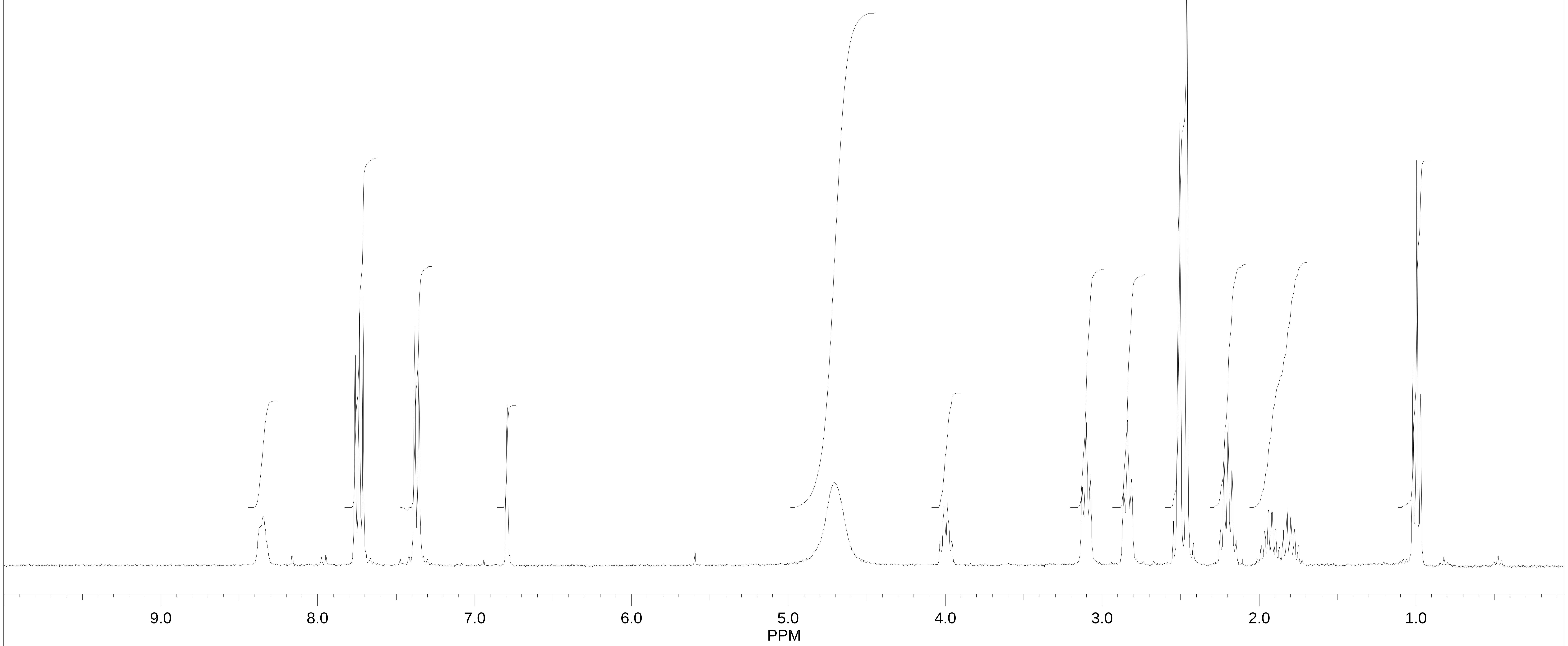

IVW36517	AC-300 SF=300.13 MHz	SI=16K, SW=5376.34, PW=2.5	AQ=1.333, RD=3.00, NS=16	SR=3364.92, TE=318K	
	Moscow, 31 July 2017	Opr: STEPANOV A.V.;	Solv: DMSO+CCl4;	BBB6273-110	
