# Supplementary material for: Selective androgen receptor degrader (SARD) to overcome antiandrogen resistance in castration-resistant prostate cancer
Source: eLife. 2023 Jan 19;12:e70700. doi: 10.7554/eLife.70700 (PMC9901937; doi:10.7554/eLife.70700)
Supplement: Source data 3. [file elife-70700-data3.zip › Figure source data/Figure 2-source data 1/Figure 2A-_RNA-Seq.doc]

**Data availability**

The datasets presented in this study can be found in online repositories. The names of the repository/repositories and accession number(s) can be found below: Genome Sequence Archive for Human (https://ngdc.cncb.ac.cn/gsa-human/browse/HRA000921) (accession: HRA000921)
